# α‐Amination of 1,3‐Dicarbonyl Compounds and Analogous Reactive Enolate‐Precursors Using Ammonia under Oxidative Conditions

**DOI:** 10.1002/anie.202501586

**Published:** 2025-05-06

**Authors:** Christopher Mairhofer, Katharina Röser, Gabriel Burel, Sarah Merzinger, Jean‐François Brière, Roland Obermüller, Mario Waser

**Affiliations:** ^1^ Institute of Organic Chemistry Johannes Kepler University Linz Altenbergerstrasse 69, 4040 Linz Austria; ^2^ CNRS, INSA Rouen Normandie, Univ Rouen Normandie, Normandie Univ CARMeN UMR 6064, INC3 M FR 3038, F‐76000 Rouen France; ^3^ Process Safety Laboratory Thermo Fisher Scientific Linz St.‐Peter‐Straße 25, 4020 Linz Austria

**Keywords:** Ammonia, Hypochlorites, Oxidative catalysis, Phase‐transfer catalysis, α‐Amination

## Abstract

We, herein, introduce a method for the direct α‐amination of different carbonyl compounds by employing aqueous ammonia as the *N*‐source. Upon using NH_3_ in combination with hypochlorites as simple oxidants under phase‐transfer catalytic conditions it is possible to carry out the direct α‐amination of reactive enolate‐precursors such as cyclic β‐ketoesters, oxindoles, as well as malonitriles and malonates with good to excellent yields, while simple ketone precursors being out of the scope thus far. Furthermore, a first proof‐of‐concept for an asymmetric variant by employing a chiral quaternary ammonium salt is reported.

## Graphical abstract

Using ammonia in combination with hypochlorites as simple oxidants under phase‐transfer conditions allows for the direct oxidative α‐amination of reactive enolate precursors such as cyclic β‐ketoesters, oxindoles, as well as malonitriles and malonates with good to excellent yields.

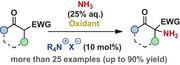

## Introduction

**Scheme 1:**
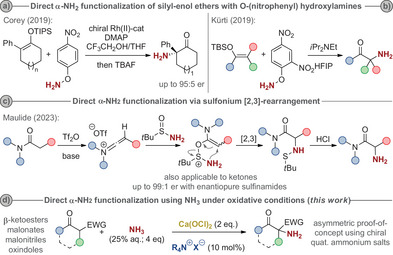
Recently reported approaches for direct α‐NH_2_ functionalizations of silyl enol ethers with O‐(nitrophenyl) hydroxylamines (a, b) and amides and ketones via a sulfonium [2,3]‐rearrangement (c) and the herein reported direct α‐amination using ammonia under oxidative conditions (d).

The importance of nitrogen‐containing compounds like naturally‐occurring primary and secondary metabolites, such as amino acids/proteins, RNA/DNA, or alkaloids,^[^ ^1^, ^2^, ^3^, ^4^ ^]^ as well as the value of (synthetically accessed) *N*‐containing small molecule‐based pharmaceutically active ingredients (APIs) cannot be over‐estimated.^[^ ^5^, ^6^, ^7^ ^]^ As a consequence, the development of efficient synthesis methods to install amino groups represents one of the most important targets in synthetic organic chemistry. Numerous approaches to introduce *N*‐containing functionalities within existing carbon‐skeletons have been reported.^[^ ^8^, ^9^, ^10^, ^11^, ^12^, ^13^, ^14^, ^15^ ^]^ Among them, those methods that allow for the direct installation of a primary unprotected amino group (─NH_2_) in the α‐position of a carbonyl compound are the most appealing, but often also most challenging ones.^[^ ^13^, ^14^, ^15^, ^16^, ^17^, ^18^, ^19^, ^20^, ^21^, ^22^, ^23^ ^]^ In general, α‐nitrogenations of carbonyl groups can be achieved by a variety of conceptually different strategies.^[^ ^24^, ^25^, ^26^, ^27^, ^28^, ^29^, ^30^, ^31^, ^32^, ^33^, ^34^, ^35^, ^36^, ^37^, ^38^, ^39^, ^40^, ^41^, ^42^, ^43^ ^]^ Approaches relying on the use of nucleophilic *N*‐sources can be carried out via a carbonyl α‐functionalization (e.g., halogenation, oxygenation, or diazotation) at first, followed by addition or substitution with a suited *N*‐nucleophile.^[^ ^24^, ^25^, ^26^, ^27^, ^28^, ^29^, ^30^ ^]^ Alternatively, nucleophilic *N*‐containing reagents have also been successfully engaged for direct α‐aminations of carbonyl derivatives under oxidative conditions.^[^ ^31^, ^32^, ^33^, ^34^ ^]^ On the other hand, (stereoselective) α‐aminations using suited electrophilic *N*‐transfer reagents have also been well‐established, i.e., employing asymmetric catalysis.^[^ ^38^, ^39^, ^40^, ^41^, ^42^, ^43^ ^]^ However, usually more complex reagents and/or subsequent functional group manipulations (e.g., deprotections or reductions) to access a free NH_2_‐functionality are necessary. Accordingly, direct free NH_2_‐transfer approaches are still difficult.^[^ ^20^, ^21^, ^22^, ^23^, ^29^, ^30^ ^]^ Recently, the groups of Corey,^[^ ^20^ ^]^ Kürti^[^ ^21^ ^]^ and Maulide^[^ ^22^, ^23^ ^]^ contributed significantly to the advancement of the field. They introduced strategies to facilitate the direct (asymmetric) α‐NH_2_‐functionalization of a) silyl enol ethers using O‐(nitrophenyl) hydroxylamines^[^ ^44^, ^45^ ^]^ (Corey^[^ ^20^ ^]^ and Kürti^[^ ^21^ ^]^; Scheme 1a/b) and b) amides or ketones as keteniminium precursors via a sulfonium [2,3]‐sigmatropic rearrangement process (Maulide^[^ ^22^, ^23^ ^]^; Scheme 1c).

Over the last years our group became engaged in the development of (asymmetric) heterofunctionalization reactions using simple inorganic heteroatom transfer reagents under oxidative conditions.^[^ ^46^, ^47^, ^48^, ^49^ ^]^ Based on the experience gathered therein and the general interest in direct α‐NH_2_‐functionalizations we now started a program exploring the suitability of aqueous ammonia, which is without doubt the cheapest and most readily available *N*‐source,^[^ ^29^, ^30^ ^]^ for the direct free α‐NH_2_‐functionalization of different enolate precursors under oxidative conditions. Importantly, it is known that NH_3_ is easily oxidized to highly reactive monochloramine (NH_2_Cl) with hypochlorites.^[^ ^50^, ^51^, ^52^ ^]^ NH_2_Cl can then be utilized as an electrophilic NH_2_‐source for *N*‐aminations under phase transfer conditions.^[^ ^53^ ^]^ However, to the best of our knowledge, such an operationally simple strategy that employs easily available and cheap reagents and catalysts has so far not been applied to the direct synthesis of α‐NH_2_‐carbonyl derivatives via a C─N bond formation. Due to the generally biphasic nature of the herein targeted transformations (organic starting materials, aqueous ammonia, inorganic oxidants) and capitalizing on the impressive recent developments in using quaternary ammonium halide catalysts (Quatsalts, R_4_N^+^X^−^) under oxidative conditions,^[^ ^54^, ^55^, ^56^, ^57^, ^58^ ^]^ we focused on the use of simple achiral Quatsalts as (phase‐transfer) catalysts for the herein targeted NH_3_‐based oxidative α‐aminations.

Very pleasingly, as outlined in this contribution, this is indeed possible by employing inorganic hypochlorites (Ca(OCl)_2_ or NaOCl) as readily available oxidants in combination with aqueous NH_3_ as the *N*‐source. Overall, this allows for a straightforward protocol for direct α‐NH_2_‐functionalizations of pronucleophiles like β‐ketoesters, malonates, and malonitriles, as well as oxindoles (Scheme 1d). In contrast to other strategies, this approach avoids any protecting group chemistry or further functional group manipulations to access the free NH_2_‐motif.

## Results and Discussion

### Optimization of Reaction Conditions

We started our investigations by testing the α‐amination of the cyclic β‐ketoester **1a** (Table 1 gives a condensed overview of the most significant results obtained in a detailed screening of different reagents and conditions^[^ ^59^ ^]^). Inspired by recent developments using Quatsalts containing an easily oxidizable counter anion (*i.e*., I^−^ or Br^−^, which can form catalytically competent higher oxidation state species like IO^−^ or Br_3_^−^ in situ under oxidative conditions),^[^ ^54^, ^55^, ^56^, ^57^, ^58^ ^]^ we used *n*Bu_4_NI as a catalyst in combination with Ca(OCl)_2_ as an oxidant and 4 eq. of aqueous ammonia at first. Gratifyingly, our initial tests showed that this system indeed allows for the formation of the targeted NH_2_‐functionalized **2a** (entries 1–4). Noteworthy, conversion of **1a** was high in most of the initially tested solvents,^[^ ^59^ ^]^ but the reactions were accompanied by the formation of unidentified side‐products as well as the corresponding α‐chlorinated β‐ketoester **3a** (which was formed in a ratio up to 50% depending on the solvent). Promising yields of around 50% **2a** were only achieved when aromatic or chlorinated solvents were used (entries 1 and 4). We tested whether **3a** may be an intermediate/precursor *en route* to **2a**. By reacting isolated **3a** with NH_3_ (4 eq.) in the presence of 20 mol% Bu_4_NI in toluene (r.t., 17 h) no formation of **2a** was observed hereby. This result clearly rules out an α‐chlorination—nucleophilic substitution reaction sequence leading to the α‐aminated target **2a**. Instead, the α‐chlorination represents a competing pathway and the formed amount of side‐product **3a** usually made up for most of the remaining mass balance (aside from **1a** and **2a**) during the optimization of the α‐amination (**3a** can very easily be removed by simple silica gel column chromatography).

**Table 1:** Optimization of the α‐amination of β‐ketoester **1a** using aqueous ammonia under oxidative phase‐transfer catalysis conditions.^a)^

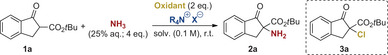						
Entry	R_4_N^+^X^−^ (mol %)	Oxidant	Solvent	t [h]	Conv. [%]^b)^	**2a** (%)^c)^
1	*n*Bu_4_NI (20)	Ca(OCl)_2_	toluene	17	>99	52
2	*n*Bu_4_NI (20)	Ca(OCl)_2_	CH_3_CN	17	>99	9
3	*n*Bu_4_NI (20)	Ca(OCl)_2_	THF	17	70	28
4	*n*Bu_4_NI (20)	Ca(OCl)_2_	CH_2_Cl_2_	17	>99	49
5	–	Ca(OCl)_2_	toluene	17	70	25
6	*n*Bu_4_NCl (20)	Ca(OCl)_2_	toluene	17	>99	65
7	*n*Bu_4_NHSO_4_ (20)	Ca(OCl)_2_	toluene	17	>99	64
8	*n*Bu_4_NClO_4_ (20)	Ca(OCl)_2_	toluene	17	>99	37
9	*n*Bu_4_NBr_3_ (20)	Ca(OCl)_2_	toluene	17	95	18
10	Aliquat 336^d)^ (20)	Ca(OCl)_2_	toluene	17	>99	76
11	Aliquat 336^d)^ (10)	Ca(OCl)_2_	toluene	17	>99	78
12	Aliquat 336^d)^ (10)	Ca(OCl)_2_	toluene	1	>99	82
13	Aliquat 336^d)^ (10)	H_2_O_2_ (35%)^e)^	toluene	1	25	–
14	Aliquat 336^d)^ (10)	tBuOOH^e)^	toluene	1	>99	–
15	Aliquat 336^d)^ (10)	Oxone	toluene	1	10	–
16	Aliquat 336^d)^ (10)	mCPBA	toluene	1	>99	–
17	Aliquat 336^d)^ (10)	NBS	toluene	1	40	–
18	Aliquat 336^d)^ (10)	Ca(OCl)_2_	PhCl	1	>99	85
19	Aliquat 336^d)^ (10)	NaOCl.5H_2_O^e)^	PhCl	1	>99	64
20	Aliquat 336^d)^ (10)	NaOCl (8% aq.)	PhCl	20	>99	79
21	Aliquat 336^d)^ (10)	Ca(OCl)_2_ ^f)^	PhCl	1	>99	91 (74)

^a)^ Unless otherwise noted, all reactions were carried out by mixing **1a** (0.1 mmol), the R_4_N^+^X^−^ catalyst and aqueous NH_3_ (25wt%, 0.4 mmol) in the indicated solvent (1.0 mL) at r.t. first, followed by addition of the oxidant (0.2 mmol) in one portion under vigorous stirring (1200 rpm).

^b)^ Conversion of **1a** determined by ^1^H NMR of the crude product

^c)^ Yield of **2a** determined by ^1^H NMR using CH_3_NO_2_ as an internal standard. Value in parenthesis gives the isolated yield on 1 mmol scale.

^d)^ [63 393–96–4]^[^ ^60^ ^]^

^e)^ 4 eq.

^f)^ Charged in three portions over 5 min.

The beneficial effect of the Quatsalt on this biphasic reaction was clearly demonstrated when carrying out the amination in the absence of any Quatsalt, which resulted in incomplete conversion and little product formation only (entry 5). Screening different Quatsalts next gave unexpected results, as we found that ammonium salts with counter anions that are less easily oxidized were catalytically active as well (entries 6 and 7), while higher oxidation state halogen‐based anions (entries 8 and 9) were less‐well suited. These results actually suggest that, contrary to our initial hypothesis, ammonium salts with (in situ formed) higher oxidation state counter anions (like (hypo)iodites or tribromides) are not relevant for the herein introduced oxidative α‐amination. Interestingly when using well‐established Aliquat 336^[^ ^60^ ^]^ next, product **2a** was obtained with an even higher yield and in a cleaner manner as compared to the afore tested tetrabutylammonium salts (entry 10). This illustrates that the Quatsalt is really primarily acting as a phase‐transfer catalyst that transfers inorganic anionic species, i.e., OH^−^, from the aqueous phase into the organic layer, and may also help solubilizing the hypochlorite. Noteworthy, catalyst loading and reaction time could be reduced without compromising yield and conversion (entries 11 and 12). Other oxidants were investigated next (entries 13–17) but none of them allowed for any product formation, thus illustrating the crucial role of the hypochlorite for this process. Aliquat 336 was also tested in other solvents, which allowed us to identify chlorobenzene as the best‐suited one (entry 18).^[^ ^59^ ^]^ Noteworthy, the amount of side product **3a** could be reduced to around 10% under these conditions. Furthermore, NaOCl was found to be a suitable oxidant as well (entries 19 and 20), but the use of aqueous NaOCl (8%) required longer reaction times to achieve full conversion (entry 20). Finally, the yield could be slightly increased by adding the oxidant in three portions (entry 21). Under these conditions, the amount of **3a** was reduced to less than 10%, thus giving product **2a** in >90% NMR yield and in 74% isolated yield on 1 mmol scale (it should be emphasized that proper mixing/stirring and sequential addition of the oxidant is becoming even more important with increasing reaction scale in order to avoid formation of somewhat larger amounts of the α‐chlorinated side‐product **3a**).

### Application Scope

**Scheme 2:**
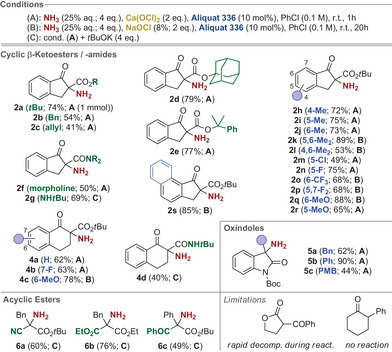
Application scope (isolated yields on 0.1 mmol scale except for **2a**).

With suited conditions for the α‐amination of **1a** at hand, we next investigated the application scope for different pronucleophiles. As outlined in Scheme 2, a broad variety of differently substituted cyclic indanone‐based α‐aminated β‐ketoesters and β‐ketoamides **2** could be straightforwardly accessed. In general, the nature of the ester‐group has an influence on the reaction, with bulky esters allowing for higher yields and cleaner transformations (compare the results for products **2a**–**e**). Furthermore, also β‐ketoamides can be successfully employed, as outlined for products **2f** and **2g**. Interestingly, the use of *sec*.‐amides requires the addition of an external stronger base, such as *t*BuOK to achieve good conversion (cond. C; less than 10% conversion under cond. A). Interestingly, depending on the substitution pattern of the starting materials (see the results obtained for products **2h**–**s**) we found it beneficial to either use the “standard” conditions with solid Ca(OCl)_2_ (cond. A; compare with entry 21, Table 1), or to employ the “slower” NaOCl cond. (cond. B; entry 20, Table 1). Here, it should be said that in principle all these products could be accessed with Ca(OCl)_2_. However, in some cases the amount of formed α‐chlorinated side product **3** was rather pronounced under conditions A (up to 30%–40%) and in those cases the use of aqueous NaOCl slowed down the overall reaction and reduced the α‐chlorination side reaction significantly (remaining amounts of around 10%–20% of side‐products **3** can in general easily be removed by silica gel column chromatography). Gratifyingly, the tetralone‐based products **4a**–**d** could also be obtained by using either cond. A, B, or C. Furthermore, we also succeeded in accessing the α‐amino‐oxindoles **5** under the standard conditions too, although the PMB‐substituent present in **5c** reduced its reactivity noteworthy. Finally, we also tested the less acidic acyclic esters **6**. Here, the addition of an external stronger base (*t*BuOK, cond. C) was again beneficial (only trace amounts of products were obtained under cond. A). Unfortunately, the methodology so far comes to its limits when using less reactive pronucleophiles like simple ketones, which do not react at all, or lactones, which rapidly underwent ring‐opening and decomposition under the reaction conditions.

### Mechanism and Safety Considerations

**Scheme 3:**
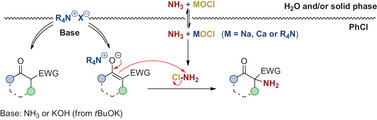
Proposed mechanistic scenario.

It is well documented that the reaction of NH_3_ with hypochlorites leads to the formation of monochloramine (NH_2_Cl), which can then act as an electrophilic NH_2_‐transfer reagent.^[^ ^50^, ^51^, ^52^, ^53^ ^]^ In addition, we carried out a control experiment where we first charged the Ca(OCl)_2_ and NH_3_ to the solvent and stirred for 10 min (formation of NH_2_Cl) followed by addition of **1a** and the Quatsalt. Hereby, we also observed product **2a** formation, but only in lower amounts of less than 30%. Furthermore, **3a** and side products were formed too. This outcome can be rationalized by the rapid formation of unstable NH_2_Cl, which, in the initial absence of any pronucleophile (first 10 min), partially decomposes under these concentrated conditions (vide infra). This result also explains why the stepwise addition of Ca(OCl)_2_ as well as the use of diluted NaOCl is beneficial (Table 1, entries 20, 21). Control experiments performed in the presence of radical traps like BHT (di‐*tert‐*butylhydroxytoluene), PBN (*N*‐*tert*‐butyl‐α‐phenylnitrone), or DPE (1,1‐diphenylethene) did not suppress product formation, thus ruling out a mechanistic scenario involving radical intermediates.^[^ ^59^ ^]^ Accordingly, the herein reported α‐amination is supposed to proceed via a rather simple phase‐transfer catalyzed two‐step procedure: i) formation of monochloramine; ii) α‐amination by means of an S_N_2‐type reaction between NH_2_Cl and the in situ formed enolate species, which is obtained upon deprotonation of the pronucleophile either by ammonia (NH_3_) or by the additional base such as *t*BuOK (respectively formed OH^−^) (Scheme 3).

Considering the well‐document instability and potentially hazardous behavior of monochloramine (which can decompose violently or form NCl_3_ under acidic conditions)^[^ ^50^, ^51^, ^52^, ^53^, ^61^, ^62^ ^]^ we have also undertaken first, rather basic, safety considerations in collaboration with the Process Safety Lab of Thermo Fisher Scientific in Linz, Austria. Carrying out the reaction under optimized conditions at 1 mmol scale no significant increase in temperature or any other violent behavior were observed, thus speaking for a rather rapid and controlled consumption of the formed NH_2_Cl. However, when just mixing aqueous NH_3_ and Ca(OCl)_2_ (or NaOCl) in an equimolar amount in the absence of any pronucleophile under isoperibolic conditions a rapid increase in temperature of around 20–30 K when using around 3 mL of NH_3_ (25%) was observed. Such a scenario, which can for example become very likely in case the addition of the pronucleophile was forgotten and/or no proper mixing and temperature control is implemented, is not yet supposed to be a serious safety concern when carrying out the reaction on small scale only. However, this eventual rapid rise in temperature together with the well‐described exothermic decomposition behavior of NH_2_Cl under various conditions, i.e., at elevated temperature,^[^ ^50^, ^51^, ^52^, ^61^, ^62^ ^]^ means that proper safety investigations and precautions have to be made when this reaction is supposed to be run on larger scale (please be aware that even in the presence of the pronucleophile an excess of NH_2_Cl will be present). Along this line, DSC‐testing on the final reaction mixture showed a medium severity and a low probability for a thermal runaway at adiabatic conditions only and DSC‐testing on the separate organic and aqueous layers does not show any significant exothermic events until 300 °C, thus showing that the NH_2_Cl is not accumulating over the progress of the reaction.^[^ ^59^ ^]^

Based on all these observations and literature reports some first suggestions would thus be: i) efficient temperature control and cooling has to be ensured, maybe even by opting for continuous flow setups;^[^ ^63^ ^]^ ii) any accumulation of NH_2_Cl has to be avoided (proper mixing and presence of nucleophile have to be ensured); iii) slightly basic conditions where decomposition of NH_2_Cl is more controlled should be maintained. *(Please be aware that this is far from being a complete list and we strongly recommend proper safety investigations and considerations when running such reactions on larger scales!!)*.

### Asymmetric Proof‐of‐Concept

**Scheme 4:**
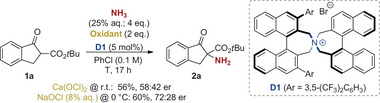
Proof‐of‐concept for the asymmetric α‐amination of **1a** using Maruoka's chiral ammonium salt catalyst **D1**.

Finally, we also tested whether these α‐aminations can be rendered enantioselective by using chiral ammonium salt catalysts that are capable of forming chiral ion pairs with in situ formed enolates.^[^ ^64^, ^65^, ^66^, ^67^, ^68^ ^]^ A variety of different catalyst scaffolds that have been well‐established for asymmetric α‐functionalizations were tested under various conditions.^[^ ^59^ ^]^ So far, we were able to identify Maruoka's spiro ammonium salt catalyst **D1** ^[^ ^69^ ^]^ as the best suited one (Scheme 4). This nowadays commercially available privileged catalyst allowed for measurable but low asymmetric induction under the standard conditions using Ca(OCl)_2_ as the oxidant (noteworthy longer reaction times as compared to the racemic approach were necessary). By using aqueous NaOCl instead allowed for higher enantioselectivities up to 72:28 er, thus representing a promising first proof‐of‐concept (which, however, will require an extensive future optimization and fine‐tuning of the catalyst scaffold).

## Conclusion

We, herein, report an operationally simple protocol for the installation of a free NH_2_‐group in the α‐position of different carbonyl compounds by using aqueous ammonia as the nitrogen‐source. Key to success is a carefully optimized phase‐transfer catalysis approach using hypochlorites in combination with quaternary ammonium salt catalysts. This allows for the efficient direct α‐amination of activated pronucleophiles such as cyclic β‐ketoesters and ‐amides, oxindoles, and acyclic esters (limitations arise when less reactive enolate‐precursors are employed). We also succeeded in obtaining a first proof‐of‐concept for an asymmetric protocol which, despite being far from perfect, in our opinion represents a promising basis for further catalyst variations and development.

## Supporting Information

The authors have cited additional references within the Supporting Information.^[^ ^70^, ^71^, ^72^, ^73^, ^74^, ^75^, ^76^, ^77^, ^78^, ^79^, ^80^, ^81^, ^82^, ^83^ ^]^

## Conflict of Interests

The authors declare no conflict of interest.

## Supporting information



Supporting information

## Data Availability

The data that support the findings of this study are available in the supplementary material of this article.
