# Supplementary material for: α‐Amination of 1,3‐Dicarbonyl Compounds and Analogous Reactive Enolate‐Precursors Using Ammonia under Oxidative Conditions
Source: Angew Chem Int Ed Engl. 2025 May 6;64(25):e202501586. doi: 10.1002/anie.202501586 (PMC12171386; doi:10.1002/anie.202501586)
Supplement: Supplementary file 1 — Supporting information [file ANIE-64-e202501586-s001.pdf]

SUPPORTING INFORMATION

α-Amination of Carbonyl Compounds Using Ammonia under Oxidative Conditions

Christopher Mairhofer,[a] Katharina Röser,[a] Gabriel Burel,[a,b] Sarah Merzinger,[a] Jean-François Brière,[b] Roland Obermüller,[c] and Mario Waser*[a]

[a] Dr. Christopher Mairhofer, Dr.in Katharina Röser, Gabriel Burel, Sarah Merzinger, Prof. Dr. Mario Waser
Institute of Organic Chemistry, Johannes Kepler University Linz
Altenbergerstrasse 69, 4040 Linz, Austria
* E-mail: mario.waser@jku.at
www.jku.at/orc/waser

[b] Gabriel Burel, Prof. Dr. Jean-François Brière
CNRS, INSA Rouen Normandie, Univ Rouen Normandie, Normandie Univ, CARMeN UMR 6064, INC3M FR 3038,
F-76000 Rouen, France

[c] Dr. Roland Obermüller
Process Safety Laboratory, Thermo Fisher Scientific Linz, St.-Peter-Straße 25,
4020 Linz, Austria

Table of Contents

1. General Information 2
2. Optimization of Reaction Conditions and Additional Information 3
3. Oxidative α-Amination Reaction 8
3.1 General Procedures 8
3.2 Characterization of α-Amination Products 10
3.3 Asymmetric α-Amination of 1a (Proof-of-Concept) 21
4. NMR Spectra 22
5. IR Spectra 53
6. HRMS Data 67
7. Differential Scanning Calorimetry 75

1. General Information

Infrared (IR) spectra were recorded on a Bruker Alpha II FTIR spectrometer with diamond ATR-module using OPUS software package and are reported in terms of frequency of absorption (cm^{-1}). Nuclear magnetic resonance (NMR) spectra were recorded on a Bruker Avance III 300 MHz spectrometer with a broad band observe probe and a sample changer for 16 samples, which is property of the Austro Czech NMR Research Center "RERI uasb". All NMR spectra were referenced on the solvent residual peak ($CDCl_3$: δ 7.26 ppm for ^1H NMR and δ 77.16 ppm for ^{13}C NMR; DMSO-d_6: 39.52 ppm for ^{13}C). Chemical shifts (δ) are given in parts per million (ppm), coupling constants (*J*) are given in Hertz (Hz). ^1H NMR spectra are reported as follows: chemical shift (δ ppm) (multiplicity, coupling constants, number of protons). Peak multiplicities are denoted as: s = singlet, d = doublet, t = triplet, q = quartet, m = multiplet, br = broad, dd = doublet of doublet, etc. High resolution mass spectra (HRMS) were recorded on an Agilent QTOF 6520 with ESI source. Low resolution (LR) mass spectrometry (MS) was performed on a Shimadzu LCMS-2020 (single quadrupole) spectrometer with ESI source. Melting points (MP) are reported in degrees Celsius (°C), using a Büchi M-560 apparatus and are reported uncorrected. Thin layer chromatography (TLC) was performed on Macherey-Nagel pre-coated TLC plates (silica gel, 60 F_{254}, 0.20 mm, ALUGRAM® Xtra SIL). Preparative column chromatography was carried out using Davisil LC 60A 70–200 MICRON silica gel or high purity grade 9385 silica gel (230–400 mesh, Supelco 40/63). Enantiomeric ratios (e.r.) were determined by HPLC analysis using a Dionex Summit HPLC system with a CHIRALPAK AD-H (4.6 × 250 mm, 5 μm) chiral stationary phase. Compound names are those generated by ChemDraw® Professional (21.0) software (PerkinElmer, CBD/cambridgesoft), following IUPAC nomenclature. The term *in vacuo* refers to removal of solvents by rotary evaporation (40 °C, 950–20 mbar) followed by drying at high vacuum.

All α-amination starting materials are known compounds and were prepared by following the literature procedures (β-Ketoesters[1] - Oxindoles[2]).

Anhydrous solvents were obtained using a purification column composed of activated alumina and were stored over activated 3 Å molecular sieves. Aqueous sodium hypochlorite solution (8% - determined by titration) and solid calcium hypochlorite (70%) were purchased commercially. The chiral Maruoka catalysts (**D1** and **D2**) were purchased from FUJIFILM Wako Pure Chemical Industries, Ltd. and used without further purification. All other chemicals were purchased from commercial suppliers and used without further purification unless otherwise stated.

---

[1] a) T. A. Moss, D. R. Fenwick, D. J. Dixon, *J. Am. Chem. Soc.* **2008**, *130*, 10076–10077; b) J. Christoffers, N. Önal, *Eur. J. Org. Chem.* **2000**, 1633–1635; c) P. Baumhof, R. Mazitschek, A. Giannis, *Angew. Chem. Int. Ed.* **2001**, *40*, 3672–3674; d) M. Lian, Z. Li, J. Du, Q. Meng, Z. Gao, *Eur. J. Org. Chem.* **2010**, *34*, 6525–6530; e) A. Pericas, A. Shafir, A. Vallribera, *Tetrahedron* **2008**, *64,* 9258–9263; f) R. A. Craig, S. A. Loskot, J. T. Mohr, D. C. Behenna, A. M. Harned, B. M. Stoltz, *Org. Lett.* **2015**, *17*, 5160–5163

[2] a) Y. Hamashima, T. Suzuki, H. Takano, Y. Shimura, M. Sodeoka, *J. Am. Chem. Soc.* **2005**, *127*, 10164–10165; b) J.-T. Xia, X.-P. Hu, *Org. Lett.* **2020**, *22*, 1102–1107; c) E. Badiola, B. Fiser, E. Gómez-Bengoa, A. Mielgo, I. Olaizola, I. Urruzuno, J. M. García, J. M. Odriozola, J. Razkin, M. Oiarbide, C. Palomo, *J. Am. Chem. Soc.* **2014**, *136*, 17869–17881.

Dynamic DSC-thermograms were recorded on a Mettler-Toledo DSC-3 calorimeter. Sealed gold-plated high-pressure crucibles type S50 (content 50 µl) were used (purchased from Bächler Feintech AG, Switzerland). The investigated temperature range was 0°C to 300°C (or 400°C) with a heating rate of 5 K/min. Typical sample mass: approx. 5 – 6 mg. Sample handling under air. Sealing of the DSC-crucibles under air.

2. Optimization of Reaction Conditions and Additional Information

Table S1. Preliminary Results and Catalyst Screening

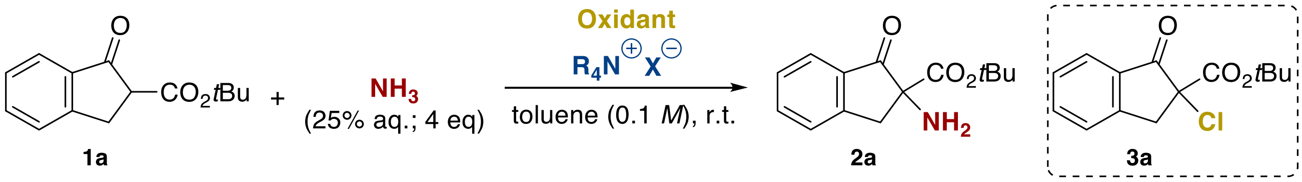

entry[a]	oxidant[b] (equiv)	cat (mol%)	time (h)	**1a**, conv. (%)[c]	yield (%)[c]
1	aq. NaOCl (2)	Bu_4NI (20)	14	46	29
2	$Ca(OCl)_2$ (2)	Bu_4NI (20)	17	>99	52
3	$Ca(OCl)_2$ (2)	—	17	70	25
4	—	—	17	*no reaction*	
5	$Ca(OCl)_2$ (1)	Bu_4NI (20)	17	51	16
6	$Ca(OCl)_2$ (3)	Bu_4NI (20)	17	>99	46
7	$Ca(OCl)_2$ (4)	Bu_4NI (20)	17	>99	29
8	$Ca(OCl)_2$ (5)	Bu_4NI (20)	17	>99	0[d]
9	$Ca(OCl)_2$ (2)	Bu_4NBr (20)	17	87	43
10	$Ca(OCl)_2$ (2)	Bu_4NCl (20)	17	>99	65
11	$Ca(OCl)_2$ (2)	Bu_4NF (20)	17	>99	65
12	$Ca(OCl)_2$ (2)	Bu_4NHSO_4 (20)	17	>99	64
13	$Ca(OCl)_2$ (2)	Bu_4NOTf (20)	17	91	39
14	$Ca(OCl)_2$ (2)	Bu_4NNO_3 (20)	17	96	63
15	$Ca(OCl)_2$ (2)	Bu_4NClO_4 (20)	17	>99	37
16	$Ca(OCl)_2$ (2)	Bu_4NBr_3 (20)	17	93	18
17	$Ca(OCl)_2$ (2)	$BnNEt_3Br$ (20)	17	75	28
18	$Ca(OCl)_2$ (2)	Aliquat® 336 (20)	17	>99	76
19	$Ca(OCl)_2$ (2)	Aliquat® 336 (10)	17	>99	78
**20**	**$Ca(OCl)_2$ (2)**	**Aliquat® 336 (10)**	**1**	**>99**	**82**
21	$Ca(OCl)_2$ (2)	Bu_3NMeCl (10)	1	>99	80
22	$Ca(OCl)_2$ (2)	*n*-Oct_2NMe_2Cl (10)	17	>99	55
23	$Ca(OCl)_2$ (2)	*n*-Oct_4NBr (10)	17	>99	66
24	$Ca(OCl)_2$ (2)	$C_{16}H_{33}NMe_3Br$ (10)	17	>99	52

[a] Unless otherwise noted, to a solution of **1a** (0.1 mmol), catalyst (0.01 or 0.02 mmol) and aq NH_3 (25wt% in water) in toluene (1 mL) was added oxidant in one portion under vigorous stirring (1200 rpm). [b] aq. NaOCl (8%). [c] Determined by ^1H NMR using CH_3NO_2 as external standard. [d] α-Chlorinated product (**3a**) or other side products formed.

Table S2. Oxidant and Preliminary Solvent Screening

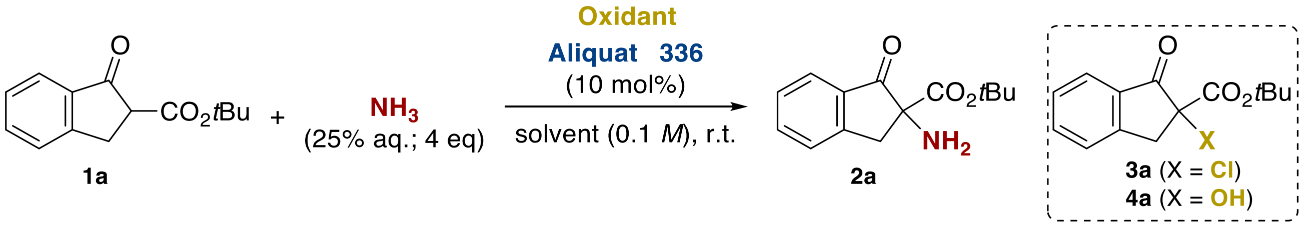

entry[a]	oxidant[b] (equiv)	solvent	time (h)	**1a**, conv. (%)[c]	yield (%)[c]
1	aq. H_2O_2 (4)	PhMe	2	25	0[d]
2	UHP (4)	PhMe	2	32	0[d]
3	$Na_2CO_3 \cdot 1.5H_2O_2$ (2)	PhMe	2	11	0[d]
4	CHP (4)	PhMe	2	>99	0[d]
5	*t*-BuOOH (4)	PhMe	2	81	0[d]
6	*t*-BuOO*t*-Bu (2)	PhMe	2	*no reaction*	
7	*t*-BuOOBz (2)	PhMe	2	*no reaction*	
8	$K_2S_2O_8$ (2)	PhMe	2	*no reaction*	
9	IBX (2)	PhMe	2	*no reaction*	
10	*t*-BuOOAc (2)	PhMe	2	33	0[d]
11	MMPP (2)	PhMe	2	>99	0[d]
12	BPO (2)	PhMe	2	76	0[d]
13	*m*-CPBA (2)	PhMe	2	>99	0[d]
14	Oxone (2)	PhMe	2	9	0[d]
15	$PhI(OAc)_2$ (2)	PhMe	2	>99	0[d]
16	$PhI(OTFA)_2$ (2)	PhMe	2	>99	0[d]
17	LPO (2)	PhMe	2	>99	0[d]
18	NCS (2)	PhMe	2	>99	0[d]
19	NBS (2)	PhMe	2	41	0[d]
20	NIS (2)	PhMe	2	>99	0[d]
21	$NaOCl_2$ (2)	PhMe	2	*no reaction*	
22	NaOCl $5H_2O$ (2)	PhCl	1	78	57
23	NaOCl $5H_2O$ (4)	PhCl	1	>99	64
24	aq. NaOCl (2)	PhCl	1	>99	79
25	$Ca(OCl)_2$ (2)	PhMe	1	>99	82
**26**	**$Ca(OCl)_2$ (2)**	**PhCl**	**1**	**>99**	**85**

[a] Unless otherwise noted, to a mixture of **1a** (0.1 mmol), Aliquat® 336 (0.01 mmol) and aq NH_3 (25wt% in water, 0.4 mmol) in solvent (1.0 mL) was added oxidant (0.2 or 0.4 mmol) in one portion under vigorous stirring (1200 rpm). [b] aq. H_2O_2: 35wt% H_2O_2 aq.; UHP: urea hydrogen peroxide; CHP: cumene hydroperoxide (contains ca. 20% aromatic hydrocarbon); IBX: 2-iodoxybenzoic acid; MMPP: Magnesium monoperoxyphthalate · $6H_2O$; BPO: dibenzoyl peroxide; *m*-CPBA: *m*-chloro peroxybenzoic acid (≤77%); LPO: lauroyl peroxide (Luperox); NCS: *N*-chlorosuccinimide; NBS: *N*-bromosuccinimide; NIS: *N*-iodosuccinimide; aq. NaOCl (12%). [c] Determined by ^1H NMR using CH_3NO_2 as external standard. [d] α-Hydroxylated product (**4a**) or other side products formed.

Table S3. Solvent Screening Results

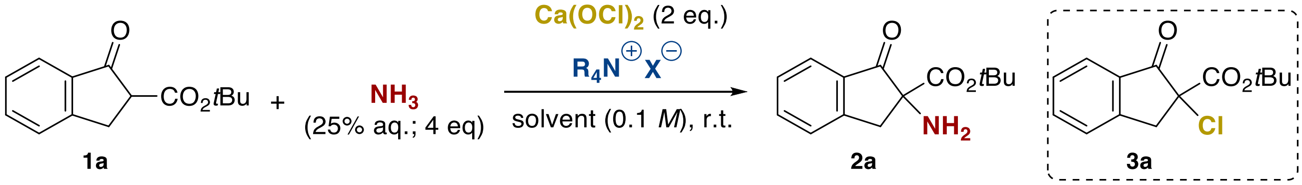

entry[a]	cat (mol%)	solvent[b]	T (°C)	time (h)	**1a**, conv. (%)[c]	yield (%)[c]
1	Bu_4NI (20)	DMF	25	17	*decomposition*	
2	Bu_4NI (20)	HFIP	25	17	87	0[e]
3	Bu_4NI (20)	acetone	25	17	21	4
4	Bu_4NI (20)	CH_3CN	25	17	>99	9
5	Bu_4NI (20)	Et_2O	25	17	70	7
6	Bu_4NI (20)	*t*-BuOMe	25	17	97	13
7	Bu_4NI (20)	CPME	25	17	>99	23
8	Bu_4NI (20)	THF	25	17	69	28
9	Bu_4NI (20)	EtOAc	25	17	>99	37
10	Bu_4NI (20)	CH_2Cl_2	25	17	>99	49
11	Bu_4NI (20)	PhMe	25	17	>99	52
12	Bu_4NI (20)	PhCl	25	17	>99	56
13	Aliquat® 336 (10)	CH_3NO_2[f]	25	17	14	0[d]
14	Aliquat® 336 (10)	*n*-hexane	25	17	>99	44
15	Aliquat® 336 (10)	$CHCl_3$	25	17	>99	61
16	Aliquat® 336 (10)	CH_2Cl_2	25	17	>99	67
17	Aliquat® 336 (10)	DCE	25	17	>99	73
18	Aliquat® 336 (10)	1,2-DFB	25	17	>99	60
19	Aliquat® 336 (10)	C_6F_6	25	17	89	62
20	Aliquat® 336 (10)	$PhCF_3$	25	17	>99	68
21	Aliquat® 336 (10)	*o*-xylol	25	17	>99	70
22	Aliquat® 336 (10)	mesitylene	25	17	>99	72
23	Aliquat® 336 (10)	C_6H_6	25	17	>99	78
24	Aliquat® 336 (10)	C_6H_5F	25	17	>99	79
25	Aliquat® 336 (10)	PhMe	25	1	>99	82
26	Aliquat® 336 (10)	PhCl	25	1	>99	85
27	Aliquat® 336 (10)	PhCl	0	17	>99	77
28	Aliquat® 336 (10)	PhCl	50	1	>99	73
**29**	**Aliquat® 336 (10)**	**PhCl**	**25**	**1**	**>99**	**91 (74)[e]**

[a] Unless otherwise noted, to a mixture of **1a** (0.1 mmol), catalyst (0.01 or 0.02 mmol) and NH_3-source (0.4 mmol) in solvent (1.0 mL) was added $Ca(OCl)_2$ (0.2 mmol) in one portion under vigorous stirring (1200 rpm). [b] DMF: *N*,*N*-dimethylformamide; HFIP: 1,1,1,3,3,3-hexafluoro-2-propanol. CPME: cyclopentyl methyl ether; DCE: 1,2-dichloroethane; 1,2-DFB: 1,2-difluorobenzene. [c] Determined by ^1H NMR using CH_3NO_2 as external standard (isolated yield on 1 mmol scale given in parenthesis). [d] α-Chlorinated product (**3a**) or other side products formed. [e] $Ca(OCl)_2$ was added in three portions (3 × 0.667 equiv) every 2 min (1200 rpm). [f] To allow for an accurate determination of conversion and yield with CH_3NO_2 as external NMR standard the crude product was dried on the Schlenk line for 3 h in this case to ensure that no residual reaction solvent is present before the CH_3NO_2 standard was added.

Scheme S1. Influence of Agitation[a]

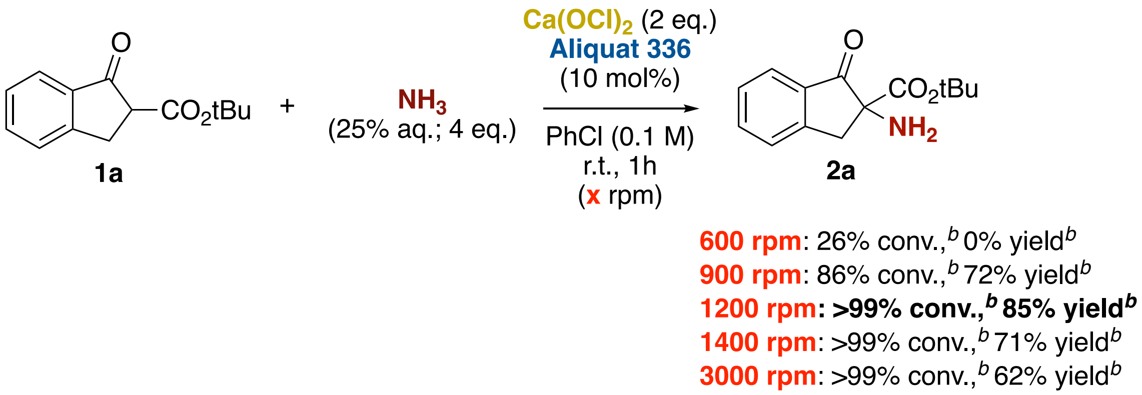

[a] Unless otherwise noted, to a mixture of **1a** (0.1 mmol), Aliquat® 336 (0.01 mmol) and aq NH_3 (25wt%, 0.4 mmol) in PhCl (1.0 mL) was added $Ca(OCl)_2$ (0.2 mmol) in one portion while stirring at ***x*** rpm. [b] Determined by ^{1}H NMR using CH_3NO_2 as external standard.

Scheme S2. Radical-Trapping Experiments[a]

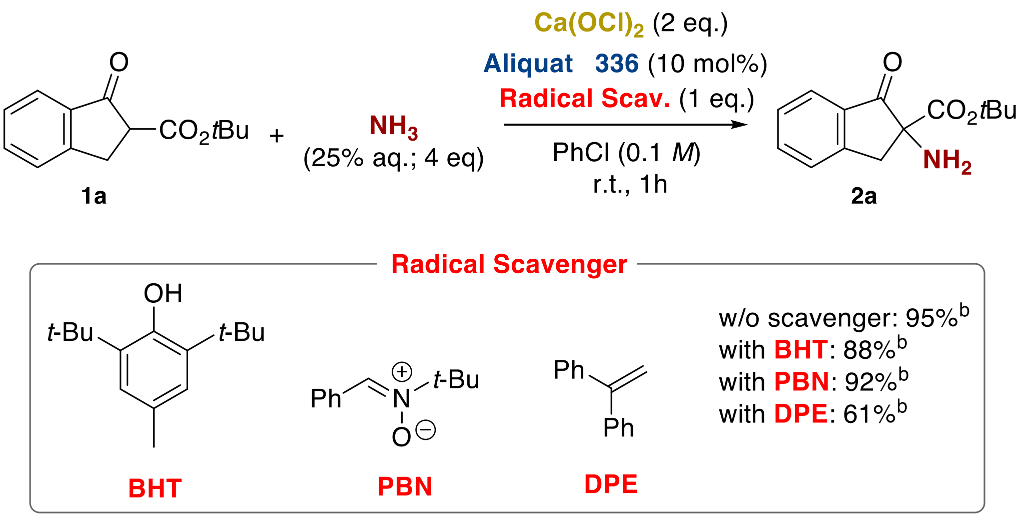

[a] Unless otherwise noted, to a mixture of **1a** (0.1 mmol), radical scavenger (0.1 mmol), Aliquat® 336 (0.01 mmol) and aq NH_3 (25wt%, 0.4 mmol) in PhCl (1.0 mL) was added $Ca(OCl)_2$ (0.2 mmol) in three portions every 2 min under vigorous stirring (1200 rpm). [b] NMR yield determined by ^{1}H NMR using CH_3NO_2 as external standard.

NOTE: When using an excess of 5 equiv. of the scavengers the results for BHT (59% **2a** + an increased amount of 30% **3a**) and DPE (61% **2a**) did not change significantly thus ruling out a radical pathway. When adding 5 equiv. PBN a lot of sideproducts and some chlorinated **3a** were formed but no product **2a**. This is however not entirely surprising considering the well-documented antioxidant properties of nitrones and their generally high reactivity towards various nucleophiles,[3] suggesting that this excess of PBN consumes the reagents (also a control experiment where PBN was reacted with $Ca(OCl)_2$ and NH_3 in the presence of Aliquat® 336 showed some unspecified decomposition of PBN).

[3] a) G. Scott, K. V. Smith, *Eur. Polym. J.* **1978**, *14*, 39–43; b) S.-I. Murahashi, Y. Imada, *Chem. Rev.* **2019**, *119*, 4684-4716.

Structures of Chiral Catalysts

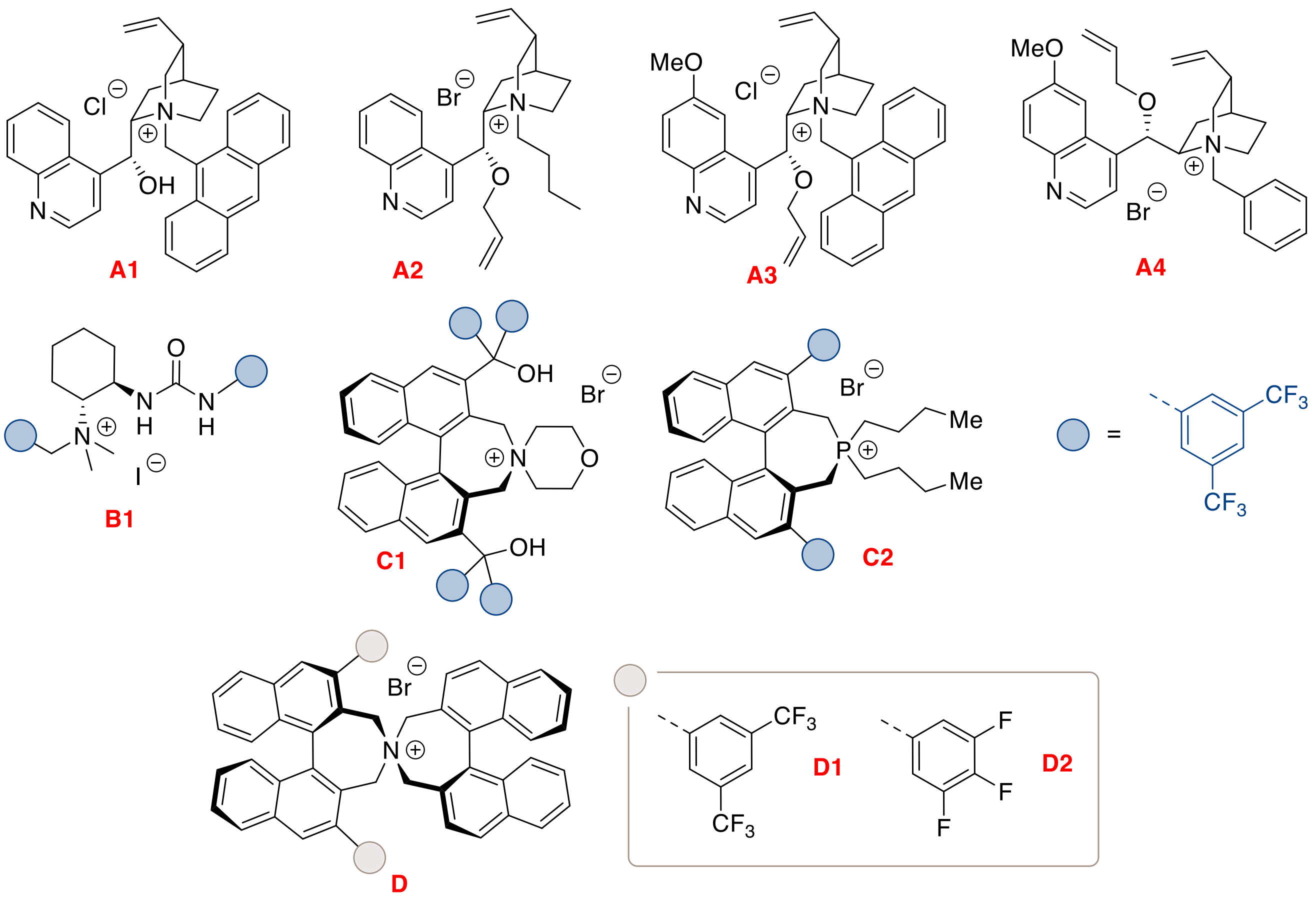

Scheme S3. Investigation of Enantioselective α-Amination of **1a**

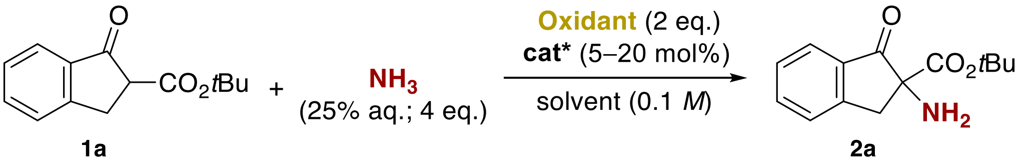

entry[a]	oxidant	cat* (mol%)	solvent	T (°C)	time (h)	2a, yield (%)[b]	e.r.[c]
1	$Ca(OCl)_2$	**A1** (20)	PhMe	25	17	40	50 : 50
2	$Ca(OCl)_2$	**A2** (20)	PhMe	25	17	74	52 : 48
3	$Ca(OCl)_2$	**A3** (20)	PhMe	25	17	70	51 : 49
4	$Ca(OCl)_2$	**A4** (20)	PhMe	25	17	52	50 : 50
5	$Ca(OCl)_2$	**B1** (5)	PhMe	25	17	66	51 : 49
7	$Ca(OCl)_2$	**C1** (5)	PhMe	25	17	56	50 : 50
8	$Ca(OCl)_2$	**C2** (5)	PhMe	25	17	72	50 : 50
9	$Ca(OCl)_2$	**D1** (5)	PhMe	25	17	56	42 : 58
10	$Ca(OCl)_2$	**D2** (5)	PhMe	25	17	56	48 : 52
11	aq. NaOCl	**D1** (5)	PhMe	25	17	58	32 : 68
12	aq. NaOCl	**D1** (5)	PhCl	25	17	82	31 : 69
13	aq. NaOCl	**D1** (5)	PhCl	0	17	60	28 : 72

[a] Unless otherwise noted, to a mixture of **1a** (0.1 mmol), chiral catalyst (2.5–20 mol%) and aq NH_3 (25wt% in water, 0.4 mmol) in solvent (1.0 mL) was added oxidant (0.2 mmol) in one portion under vigorous stirring (1200 rpm). [b] Determined by ^{1}H NMR using CH_3NO_2 as external standard. [c] Enantiomeric ratios were determined by chiral HPLC (CHIRALPAK AD-H column, *n*-hexane/2-propanol = 94/6, flow rate = 1.0 mL/min, 10 °C).

3. Oxidative α-Amination Reaction

3.1 General Procedures

Conditions A:

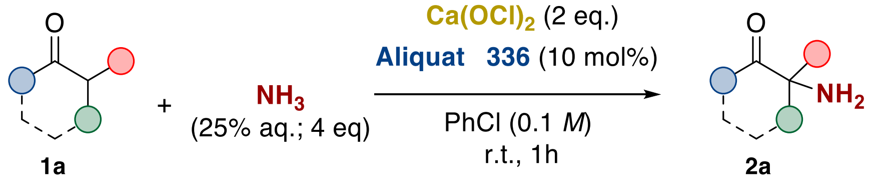

To a stirring mixture (300 rpm) of starting material (0.100 mmol) in 0.9 mL chlorobenzene was added a solution of Aliquat® 336 (100.0 µL, 0.010 mmol, 0.1 *M* in chlorobenzene) followed by addition of aqueous NH_3 (25wt% in H_2O, 29.9 µL, 0.40 mmol) within 20 seconds. Under rapid stirring (1200 rpm) $Ca(OCl)_2$ (70% purity) was added in three portions (3 × 13.6 mg, total: 40.9 mg, 0.200 mmol) every 2 min and the suspension was stirred for 1 h at 25 °C. The reaction mixture was diluted with 3 mL CH_2Cl_2 and filtered over a short pad of anhydrous Na_2SO_4/silica (diameter 2 cm, height Na_2SO_4 = 3 cm, silica = 1 cm) and washed with additional 35 mL CH_2Cl_2 and 0.5 mL MeOH. The solvents were removed *in vacuo* and the crude product was analyzed by qNMR using $MeNO_2$ as external standard (133.3 µL, 0.667 equiv., 0.5 *M* in $CDCl_3$; Note: *cf.*: for **2a**, normalization of the nitromethane CH_3 integral to 200 directly gave the NMR yield (%) for one of the diastereotopic doublet-integrals of **2a**). Isolated yields are reported after purification by silica gel column chromatography.

Conditions B:

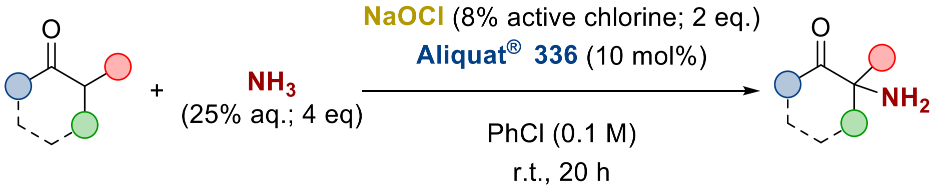

To a stirring mixture (300 rpm) of starting material (0.100 mmol) in 0.9 mL chlorobenzene was added a solution of Aliquat® 336 (100.0 µL, 0.010 mmol, 0.1 *M* in chlorobenzene) followed by addition of aqueous NH_3 (25wt% in H_2O, 29.9 µL, 0.40 mmol) within 20 seconds. Under rapid stirring (1200 rpm) NaOCl (8% active chlorine; 168 µL, 0.20 mmol) was added and the reaction mixture was stirred for 20 h at 25 °C. The reaction mixture was diluted with 3 mL CH_2Cl_2 and filtered over a short pad of anhydrous Na_2SO_4/silica (diameter 2 cm, height Na_2SO_4 = 3 cm, silica = 1 cm) and washed with additional 35 mL CH_2Cl_2 and 0.5 mL MeOH. The solvents were removed *in vacuo* and the crude product was analyzed by qNMR using $MeNO_2$ as external standard (133.3 µL, 0.667 equiv., 0.5 *M* in $CDCl_3$; Note: *cf.*: for **2a**, normalization of the nitromethane CH_3 integral to 200 directly gave the NMR yield (%) for one of the diastereotopic doublet-integrals of **2a**). Isolated yields are reported after purification by silica gel column chromatography.

Conditions C:

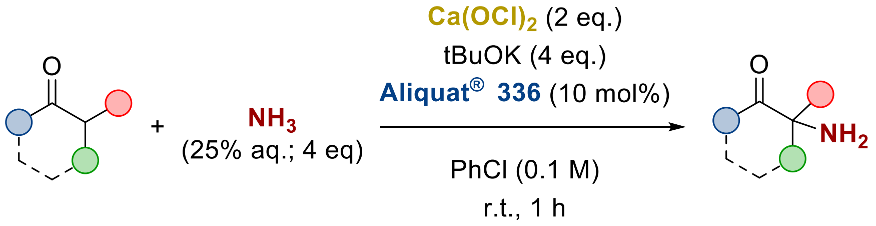

To a stirring mixture (300 rpm) of starting material (0.100 mmol) in 0.9 mL chlorobenzene was added a solution of Aliquat® 336 (100.0 µL, 0.010 mmol, 0.1 *M* in chlorobenzene) followed by addition of tBuOK (4 eq., 44.9 mg, 0.40 mmol) and aqueous NH_3 (25wt% in H_2O, 29.9 µL, 0.40 mmol) within 20 seconds. Under rapid stirring (1200 rpm) $Ca(OCl)_2$ (70% purity) was added in three portions (3 × 13.6 mg, total: 40.9 mg, 0.200 mmol) every 2 min and the suspension was stirred for 1 h at 25 °C. The reaction mixture was diluted with 3 mL CH_2Cl_2 and filtered over a short pad of anhydrous Na_2SO_4/silica (diameter 2 cm, height Na_2SO_4 = 3 cm, silica = 1 cm) and washed with additional 35 mL CH_2Cl_2 and 0.5 mL MeOH. The solvents were removed *in vacuo* and the crude product was analyzed by qNMR using $MeNO_2$ as external standard (133.3 µL, 0.667 equiv., 0.5 *M* in $CDCl_3$; Note: *cf.*: for **2a**, normalization of the nitromethane CH_3 integral to 200 directly gave the NMR yield (%) for one of the diastereotopic doublet-integrals of **2a**). Isolated yields are reported after purification by silica gel column chromatography.

3.2 Characterization of α-Amination Products

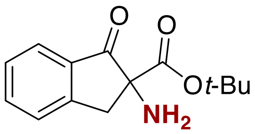

***tert*-Butyl 2-amino-1-oxo-2,3-dihydro-1*H*-indene-2-carboxylate (2a):**[4] obtained in 74% yield (0.183 g, 0.74 mmol) using **conditions A**; purified by column chromatography on silica gel (Supelco Art. 9385 silica 30/64; eluent: CH_2Cl_2–MeOH = 80:1 to 40:1). White solid; **MP**: 61.1–62.4 °C; **TLC**, R_f = 0.49 (CH_2Cl_2–MeOH = 40:1, ninhydrin); **IR** (neat) 3382 (NH_2), 3317 (NH_2), 2978, 1730, 1712, 1605 (NH_2), 1147, 843, 745 cm^{-1}; **^{1}H NMR** (300 MHz, $CDCl_3$, 298 K): δ 7.76 (d, *J* = 7.4 Hz, 1H), 7.61 (td, *J* = 7.5, 1.2 Hz, 1H), 7.49–7.31 (m, 2H), 3.62 (d, *J* = 17.0 Hz, 1H), 3.03 (d, *J* = 17.0 Hz, 1H), 2.10 (s, 2H), 1.32 (s, 9H); **^{13}C NMR** (75 MHz, $CDCl_3$, 298 K): δ 202.9, 171.5, 152.7, 135.6, 134.5, 127.9, 126.4, 125.1, 82.6, 67.8, 41.5, 27.8; **HRMS** (ESI+) *m/z* calcd for $[C_{14}H_{17}NO_3+H]^+$ 248.1281, found 248.1281; also detected *m/z* calcd for $[C_{14}H_{17}NO_3+Na]^+$ 270.1101, found 270.1104 (+1.3 ppm).

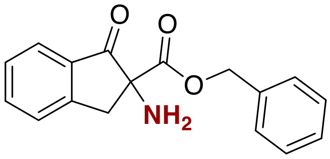

Benzyl 2-amino-1-oxo-2,3-dihydro-1*H*-indene-2-carboxylate (2b): obtained in 54% yield (0.0152 g, 0.054 mmol) using **conditions A**; purified by column chromatography on silica gel (Supelco Art. 9385 silica 30/64; eluent: CH_2Cl_2–MeOH = 80:1 to 40:1). Brown oil; **TLC**, R_f = 0.23 (CH_2Cl_2–MeOH = 40:1, ninhydrin); **IR** (neat) 3373 (NH_2), 3311 (NH_2), 2930, 1741, 1714, 1655, 1606 (NH_2), 1464, 1455, 1211, 1177, 935, 744, 695 cm^{-1}; **^{1}H NMR** (300 MHz, $CDCl_3$, 298 K): δ 7.89 (d, *J* = 8.2 Hz, 1H), 7.73 (td, *J* = 7.5, 1.3 Hz, 1H), 7.56–7.46 (m, 2H), 7.39–7.31 (m, 3H), 7.28–7.20 (m, 2H), 5.26 (d, *J* = 12.5 Hz, 1H), 5.16 (d, *J* = 12.5 Hz, 1H), 3.77 (d, *J* = 17.1 Hz, 1H), 3.17 (d, *J* = 17.1 Hz, 1H), 2.27 (s, 2H); **^{13}C NMR** (75 MHz, $CDCl_3$, 298 K): δ 202.1, 172.4, 152.6, 135.9, 135.4, 134.2, 128.6, 128.4, 128.2, 127.8, 126.6, 125.4, 67.5, 67.5, 41.3; **HRMS** (ESI+) *m/z* calcd for $[C_{17}H_{15}NO_3+H]^+$ 282.1125, found 282.1124 (−0.2 ppm).

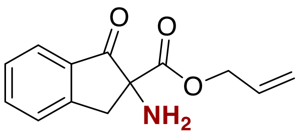

Allyl 2-amino-1-oxo-2,3-dihydro-1*H*-indene-2-carboxylate (2c): obtained in 41% yield (0.0249 g, 0.041 mmol) using **conditions A**; purified by column chromatography on silica gel (Supelco Art. 9385 silica 30/64; eluent: CH_2Cl_2–MeOH = 80:1 to 40:1). Brown oil; **TLC**, R_f = 0.28 (CH_2Cl_2–MeOH = 40:1, ninhydrin); **IR** (neat) 3369 (NH_2), 3299 (NH_2), 2928, 1741, 1715, 1649, 1606 (NH_2), 1465, 1211, 933, 749 cm^{-1}; **^{1}H NMR** (300 MHz, $CDCl_3$, 298 K): δ 7.81 (d, *J* = 7.7 Hz, 1H), 7.72–7.58 (m, 1H), 7.52–

[4] K. Shibatomi, Y. Soga, A. Narayama, I. Fujisawa,S. Iwasa, *J. Am. Chem. Soc.* **2012**, *134*, 9836–9839.

7.38 (m, 2H), 5.90–5.68 (m, 1H), 5.22–5.03 (m, 2H), 4.67–4.49 (m, 2H), 3.72 (d, J = 17.1 Hz, 1H), 3.10 (d, J = 17.1 Hz, 1H), 2.29 (s, 2H); **^{13}C NMR** (75 MHz, $CDCl_3$, 298 K): δ 202.2, 172.3, 152.6, 136.0, 134.2, 131.4, 128.2, 126.6, 125.4, 118.5, 67.4, 66.4, 41.4; **HRMS** (ESI+) *m/z* calcd for $[C_{13}H_{13}NO_3+H]^+$ 232.0968, found 232.0969 (+0.4 ppm).

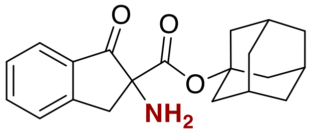

Adamantan-1-yl 2-amino-1-oxo-2,3-dihydro-1*H*-indene-2-carboxylate (2d): obtained in 79% yield (0.0257 g, 0.079 mmol) using **conditions A**; purified by column chromatography on silica gel (Supelco Art. 9385 silica 30/64; eluent: CH_2Cl_2–MeOH = 80:1 to 40:1). Yellow oil; **TLC**, R_f = 0.37 (CH_2Cl_2–MeOH = 40:1, ninhydrin); **IR** (neat) 3377 (NH_2), 3316 (NH_2), 2912, 2853, 1716, 1605 (NH_2), 1425, 1355, 1215, 1203, 1066, 1051, 934, 913, 858, 743, 725, 698 cm^{-1}; **^{1}H NMR** (300 MHz, $CDCl_3$, 298 K): δ 7.78 (d, J = 7.7 Hz, 1H), 7.62 (td, J = 7.5, 1.3 Hz, 1H), 7.49–7.33 (m, 2H), 3.64 (d, J = 17.0 Hz, 1H), 3.04 (d, J = 17.0 Hz, 1H), 2.10 (s, 5H), 1.97–1.90 (m, 6H), 1.61–1.54 (m, 6H); **^{13}C NMR** (75 MHz, $CDCl_3$, 298 K): δ 203.0, 171.2, 152.8, 135.6, 134.6, 127.9, 126.4, 125.1, 82.6, 67.9, 41.7, 41.0, 36.1, 30.9; **HRMS** (ESI+) *m/z* calcd for $[C_{20}H_{23}NO_3+H]^+$ 326.1751, found 326.1750 (−0.3 ppm).

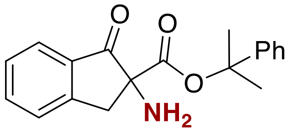

2-Phenylpropan-2-yl 2-amino-1-oxo-2,3-dihydro-1*H*-indene-2-carboxylate (2e): obtained in 77% yield (0.0238 g, 0.077 mmol) using **conditions A**; purified by column chromatography on silica gel (Supelco Art. 9385 silica 30/64; eluent: CH_2Cl_2–MeOH = 80:1 to 40:1). Colorless oil; **TLC**, R_f = 0.37 (CH_2Cl_2–MeOH = 40:1, ninhydrin); **IR** (neat) 3375 (NH_2), 3304 (NH_2), 2981, 2931, 1740, 1713, 1606 (NH_2), 1587, 1211, 1194, 1134, 1101, 909, 752, 698 cm^{-1}; **^{1}H NMR** (300 MHz, $CDCl_3$, 298 K): δ 7.86 (d, J = 7.7 Hz, 1H), 7.68 (td, J = 7.5, 1.3 Hz, 1H), 7.57–7.41 (m, 2H), 7.32–7.21 (m, 3H), 7.18–7.10 (m, 2H), 3.70 (d, J = 17.0 Hz, 1H), 3.11 (d, J = 17.0 Hz, 1H), 2.14 (s, 2H), 1.68 (s, 3H), 1.67 (s, 3H); **^{13}C NMR** (75 MHz, $CDCl_3$, 298 K): δ 202.6, 171.0, 152.6, 145.1, 135.8, 134.7, 128.4, 128.1, 127.3, 126.6, 125.3, 124.1, 83.9, 67.8, 41.5, 28.3, 28.2; **HRMS** (ESI+) *m/z* calcd for $[C_{19}H_{19}NO_3+Na]^+$ 332.1257, found 332.1255 (−0.6 ppm).

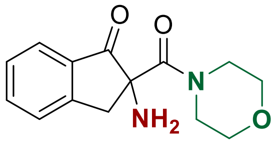

2-Amino-2-(morpholine-4-carbonyl)-2,3-dihydro-1*H*-inden-1-one (2f): obtained in 50% yield (0.0136 g, 0.050 mmol) using **conditions A**; purified by column chromatography on silica gel (Supelco Art. 9385 silica 30/64; eluent: CH_2Cl_2–MeOH = 80:1 to 40:1). Yellowish oil; **TLC**, *R*f = 0.25 (CH_2Cl_2–

MeOH = 40:1, ninhydrin); **IR** (neat) 3357 (NH_2), 3292 (NH_2), 1716, 1626, 1607 (NH_2), 1428, 1271, 1239, 1112, 1067, 1025, 910, 728 cm^{-1}; **^{1}H NMR** (300 MHz, $CDCl_3$, 298 K): δ 7.84 (d, *J* = 7.64 Hz, 1H), 7.68-7.62 (m, 1H), 7.48-7.42 (m, 2H), 3.64-3.41 (m, 9H), 3.09 (d, *J* = 17.39 Hz, 1H), 2.06 (br s, 2H); **^{13}C NMR** (75 MHz, $CDCl_3$, 298 K): δ 202.8, 171.2, 150.6, 136.0, 134.3, 128.6, 127.1, 125.6, 66.9, 66.6, 45.9, 41.4; **HRMS** (ESI+) *m/z* calcd for $[C_{14}H_{16}N_2O_3+H]^+$ 261.1234, found 261.1235 (+0.4 ppm).

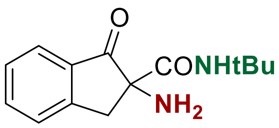

2-Amino-*N*-(*tert*-butyl)-1-oxo-2,3-dihydro-1*H*-indene-2-carboxamide (2g): obtained in 69% yield (0.0170 g, 0.069 mmol) using **conditions C**; purified by column chromatography on silica gel (Supelco Art. 9385 silica 30/64; eluent: CH_2Cl_2–MeOH = 80:1 to 40:1). Light-yellow solid, **MP**: 109.5-110.6 °C; **TLC**, *R*f = 0.44 (CH_2Cl_2–MeOH = 40:1, ninhydrin); **IR** (neat) 3386 (NH_2), 3338 (NH_2), 3312 (NH_2), 2965, 2926, 2854, 1709, 1658, 1602 (NH_2), 1515, 1467, 1210, 844, 743, 694 cm^{-1}; **^{1}H NMR** (300 MHz, $CDCl_3$) δ 7.78 (d, *J* = 7.7 Hz, 1H), 7.61 (td, *J* = 7.5, 1.3 Hz, 1H), 7.52 – 7.33 (m, 3H), 3.79 (d, *J* = 17.1 Hz, 1H), 2.86 (d, *J* = 17.1 Hz, 1H), 1.91 (s, 2H), 1.35 (s, 9H); **^{13}C NMR** (75 MHz, $CDCl_3$) δ 206.2, 171.2, 152.7, 135.7, 134.9, 128.0, 126.5, 125.0, 67.1, 51.1, 42.3, 28.7; **HRMS** (ESI+) *m/z* calcd for $[C_{14}H_{18}N_2O_2+H]^+$ 247.1441, found 247.1434 (−2.8 ppm).

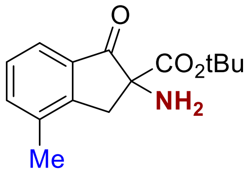

***tert*-Butyl 2-amino-4-methyl-1-oxo-2,3-dihydro-1*H*-indene-2-carboxylate (2h):** obtained in 72% yield (0.0188 g, 0.072 mmol) using **conditions A**; purified by column chromatography on silica gel (Supelco Art. 9385 silica 30/64; eluent: CH_2Cl_2–MeOH = 80:1 to 40:1). Brown oil; **TLC**, *R*f = 0.29 (CH_2Cl_2–MeOH = 40:1, ninhydrin); **IR** (neat) 3374 (NH_2), 3306 (NH_2), 2978, 2931, 1736, 1712, 1605 (NH_2), 1591, 1369, 1251, 1148, 844, 766, 743 cm^{-1}; **^{1}H NMR** (300 MHz, $CDCl_3$) δ 7.62 (d, *J* = 7.5 Hz, 1H), 7.43 (d, *J* = 7. 3 Hz, 1H), 7.36 – 7.27 (m, 1H), 3.52 (d, *J* = 16.9 Hz, 1H), 2.92 (d, *J* = 16.9 Hz, 1H), 2.34 (s, 3H), 2.03 (s, 2H), 1.35 (s, 9H); **^{13}C NMR** (75 MHz, $CDCl_3$) δ 203.1, 171.6, 151.6, 136.0, 135.6, 134.1, 128.1, 122.5, 82.5, 67.6, 40.3, 27.7, 17.8; **HRMS** (ESI+) *m/z* calcd for $[C_{15}H_{19}NO_3+Na]^+$ 284.1257, found 284.1243 (−4.9 ppm).

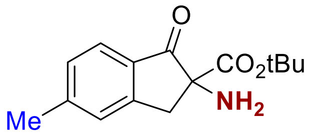

***tert*-Butyl 2-amino-5-methyl-1-oxo-2,3-dihydro-1*H*-indene-2-carboxylate (2i):** obtained in 75% yield (0.0205 g, 0.075 mmol) using **conditions A**; purified by column chromatography on silica gel

(Supelco Art. 9385 silica 30/64; eluent: CH_2Cl_2–MeOH = 80:1 to 40:1). Brown oil; **TLC**, *R*f = 0.81 (CH_2Cl_2–MeOH = 9:1, ninhydrin); **IR** (neat) 3378 (NH_2), 3314 (NH_2), 2973, 2927, 1735, 1709, 1609 (NH_2), 1589, 1150, 940, 846, 782, 749 cm^{-1}; **^{1}H NMR** (300 MHz, $CDCl_3$, 298 K): δ 7.67 (d, *J* = 7.87 Hz, 1H), 7.25 (s, 1H), 7.20 (d, *J* = 7.98 Hz, 1H), 3.59 (d, *J* = 16.99 Hz, 1H), 3.01 (d, *J* = 16.99 Hz, 1H), 2.45 (s, 3H), 2.27 (br s, 2 H), 1.34 (s, 9H); **^{13}C NMR** (75 MHz, $CDCl_3$, 298 K): δ 202.2, 171.6, 153.3, 147.1, 132.1, 129.2, 126.8, 125.0, 82.6, 68.0, 41.3, 27.8, 22.3; **HRMS** (ESI+) *m/z* calcd for $[C_{15}H_{19}NO_3+H]^+$ 262.1438, found 262.1440 (+0.8 ppm).

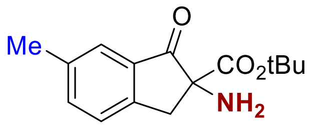

***tert*-Butyl 2-amino-6-methyl-1-oxo-2,3-dihydro-1*H*-indene-2-carboxylate (2j):** obtained in 73% yield (0.0196 g, 0.073 mmol) using **conditions A**; purified by column chromatography on silica gel (Supelco Art. 9385 silica 30/64; eluent: CH_2Cl_2–MeOH = 80:1 to 40:1). Light-brown oil; **TLC**, *R*f = 0.75 (CH_2Cl_2–MeOH = 9:1, ninhydrin); **IR** (neat) 3383 (NH_2), 3318 (NH_2), 2979, 2925, 2856, 1737, 1715, 1614 (NH_2), 1369, 1252, 1221, 1149, 1114, 958, 843, 789, 778, 504 cm^{-1}; **^{1}H NMR** (300 MHz, $CDCl_3$, 298 K): δ 7.58 (s, 1H), 7.44 (dd, *J* = 7.87 Hz, 1.30 Hz, 1H), 7.34 (d, *J* = 7.84 Hz, 1H), 3.59 (d, *J* = 16.86 Hz, 1H), 3.03 (d, *J* = 16.86 Hz, 1H), 2.41 (s, 3H), 2.29 (br s, 2H), 1.34 (s, 9H); **^{13}C NMR** (75 MHz, $CDCl_3$, 298 K): δ 202.7, 171.4, 150.2, 138.0, 137.0, 134.6, 126.1, 125.1, 82.7, 68.1, 41.0, 27.9, 21.2; **HRMS** (ESI+) *m/z* calcd for $[C_{15}H_{19}NO_3+H]^+$ 262.1438, found 262.1435 (-1.1 ppm).

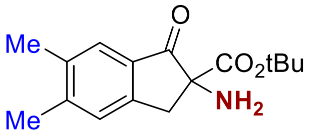

***tert*-Butyl 2-amino-5,6-dimethyl-1-oxo-2,3-dihydro-1*H*-indene-2-carboxylate (2k):** obtained in 89% yield (0.0245 g, 0.089 mmol) using **conditions B**; purified by column chromatography on silica gel (Supelco Art. 9385 silica 30/64; eluent: CH_2Cl_2–MeOH = 80:1 to 40:1). Light-brown oil; **TLC**, *R*f = 0.44 (CH_2Cl_2–MeOH = 40:1, ninhydrin); **IR** (neat) 3401 (NH_2), 3332 (NH_2), 2976, 2928, 2866, 1723, 1702, 1615 (NH_2), 1579, 1364, 1262 1217, 1153, 1126, 848, 746 cm^{-1}; **^{1}H NMR** (300 MHz, $CDCl_3$, 298 K): δ 7.52 (s, 1H), 7.21 (s, 1H), 3.55 (d, *J* = 16.84 Hz, 1H), 2.96 (d, *J* = 16.84 Hz, 1H), 2.34 (s, 3H), 2.29 (s, 3H), 2.26 (br s, 2H), 1.33 (s, 9H); **^{13}C NMR** (75 MHz, $CDCl_3$, 298 K): δ 202.4, 171.7, 151.0, 146.1, 136.9, 132.5, 127.2, 125.4, 82.5, 68.0, 41.0, 27.8, 21.0, 19.9; **HRMS** (ESI+) *m/z* calcd for $[C_{16}H_{21}NO_3+H]^+$ 276.1594, found 276.1593 (-0.4 ppm).

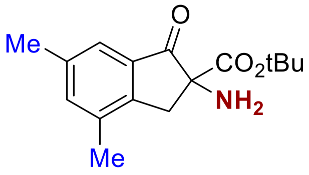

***tert*-Butyl 2-amino-4,6-dimethyl-1-oxo-2,3-dihydro-1*H*-indene-2-carboxylate (2l):** obtained in 53% yield (0.0146 g, 0.053 mmol) using **conditions B**; purified by column chromatography on silica gel (Supelco Art. 9385 silica 30/64; eluent: CH_2Cl_2–MeOH = 80:1 to 40:1). Brown oil; **TLC**, *Rf* = 0.34 (CH_2Cl_2–MeOH = 40:1, ninhydrin); **IR** (neat) 3374 (NH_2), 3366 (NH_2), 3313 (NH_2), 2975, 2925, 2858, 1734, 1711, 1617 (NH_2), 1591, 1530, 1393, 1253, 1152, 843 cm^{-1}; **^{1}H NMR** (300 MHz, $CDCl_3$) δ 7.41 (s, 1H), 7.26 (s, 1H), 3.47 (d, *J* = 16.86 Hz, 2H), 2.88 (d, *J* = 16.86 Hz, 2H), 2.36 (s, 3H), 2.30 (s, 5H), 1.35 (s, 9H); **^{13}C NMR** (75 MHz, $CDCl_3$) δ 203.0, 171.7, 149.2, 138.1, 137.5, 135.3, 134.3, 122.5, 82.6, 68.0, 40.0, 27.9, 21.1, 17.8; **HRMS** (ESI+) *m/z* calcd for $[C_{16}H_{21}NO_3+H]^+$ 276.1594, found 276.1595 (+0.4 ppm).

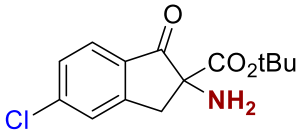

***tert*-Butyl 2-amino-5-chloro-1-oxo-2,3-dihydro-1*H*-indene-2-carboxylate (2m):** obtained in 49% yield (0.0138 g, 0.049 mmol) using **conditions A**; purified by column chromatography on silica gel (Supelco Art. 9385 silica 30/64; eluent: CH_2Cl_2–MeOH = 80:1 to 40:1). White solid, **MP**: 87.9-90.2 °C; **TLC**, *Rf* = 0.27 (CH_2Cl_2–MeOH = 40:1, ninhydrin); **IR** (neat) 3399 (NH_2), 3369 (NH_2), 3329 (NH_2), 2976, 2925, 1732, 1704, 1599 (NH_2), 1574, 1513, 1369, 1243, 1213, 1148, 844 cm^{-1}; **^{1}H NMR** (300 MHz, $CDCl_3$) δ 7.71 (d, *J* = 8.2 Hz, 1H), 7.46 (s, 1H), 7.38 (d, *J* = 9.1 Hz, 1H), 3.60 (d, *J* = 16.9 Hz, 1H), 3.03 (d, *J* = 17.1 Hz, 1H), 2.11 (s, 2H), 1.34 (s, 9H); **^{13}C NMR** (75 MHz, $CDCl_3$) δ 201.4, 171.2, 154.2, 142.2, 133.0, 128.8, 126.7, 126.2, 83.0, 68.0, 41.2, 27.8; **HRMS** (ESI+) *m/z* calcd for $[C_{14}H_{16}ClNO_3+Na]^+$ 304.0711, found 304.0707 (−1.31 ppm).

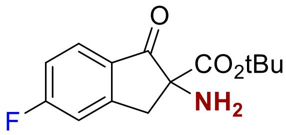

***tert*-Butyl 2-amino-5-fluoro-1-oxo-2,3-dihydro-1*H*-indene-2-carboxylate (2n):** obtained in 75% yield (0.0199 g, 0.075 mmol) using **conditions A**; purified by column chromatography on silica gel (Supelco Art. 9385 silica 30/64; eluent: CH_2Cl_2–MeOH = 80:1 to 40:1). Colorless oil; **TLC**, *Rf* = 0.23 (CH_2Cl_2–MeOH = 40:1, ninhydrin); **IR** (neat) 3375 (NH_2), 3314 (NH_2), 2978, 2931, 1738, 1717, 1652, 1615 (NH_2), 1593, 1481, 1369, 1252, 1150, 943, 842 cm^{-1}; **^{1}H NMR** (300 MHz, $CDCl_3$) δ 7.79 (dd, *J* = 8.3, 5.4 Hz, 1H), 7.18 – 7.03 (m, 2H), 3.61 (d, *J* = 17.2 Hz, 1H), 3.04 (d, *J* = 17.2 Hz, 1H), 1.98 (s, 2H), 1.34 (s, 9H); **^{13}C NMR** (75 MHz, $CDCl_3$) δ 200.9, 171.3, 167.6 (d, *J* = 257.3 Hz), 155.6 (d, *J* =

10.4 Hz), 130.9 (d, J = 2.0 Hz), 127.4 (d, J = 10.4 Hz), 116.2 (d, J = 23.7 Hz), 113.1 (d, J = 22.5 Hz), 82.9, 68.2, 41.4, 27.8; **^{19}F NMR** (282 MHz, $CDCl_3$) δ -101.08 (1F); **HRMS** (ESI+) *m/z* calcd for $[C_{14}H_{16}FNO_3+H]^+$ 266.1187, found 266.1191 (−1.5 ppm).

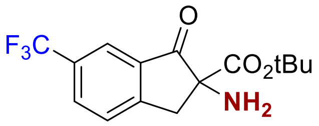

***tert*-Butyl 2-amino-1-oxo-6-(trifluoromethyl)-2,3-dihydro-1*H*-indene-2-carboxylate (2o):** obtained in 68% yield (0.0214 g, 0.068 mmol) using **conditions B**; purified by column chromatography on silica gel (Supelco Art. 9385 silica 30/64; eluent: CH_2Cl_2–MeOH = 80:1 to 40:1). Beige solid, **MP**: 57.9-60.1 °C; **TLC**, *Rf* = 0.50 (CH_2Cl_2–MeOH = 40:1, ninhydrin); **IR** (neat) 3371 (NH_2), 3341 (NH_2), 2985, 2969, 2935, 2925, 1642, 1602 (NH_2), 1480, 1409, 1367, 1319, 1255, 1166, 1119, 833, 786, 630 cm^{-1}; **^{1}H NMR** (300 MHz, $CDCl_3$) δ 8.06 (s, 1H), 7.88 (dd, J = 8.05; 1,26 Hz, 1H), 7.61 (d, J = 8.04 Hz, 1H), 3.70 (d, J = 17.41 Hz, 2H), 3.13 (d, J = 17.41 Hz, 2H), 2.14 (s, 2H), 1.35 (s, 9H); **^{13}C NMR** (75 MHz, $CDCl_3$) δ 201.6, 170.8, 155.9, 135.0, 132.1 (q, J = 3.4 Hz), 130.9 (q, J = 33.3 Hz), 127.2, 123.5 (q, J = 272.3 Hz), 122.4 (q, J = 4.1 Hz), 121.9, 83.3, 68.1, 41.5, 27.8; **^{19}F NMR** (282 MHz, $CDCl_3$) δ -62.55 (s, 3F); **HRMS** (ESI+) *m/z* calcd for $[C_{15}H_{16}F_3NO_3+H]^+$ 316.1155, found 316.1155 (+/- 0 ppm).

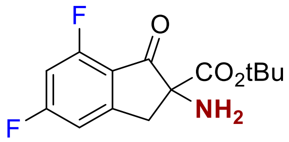

***tert*-Butyl 2-amino-5,7-difluoro-1-oxo-2,3-dihydro-1*H*-indene-2-carboxylate (2p):** obtained in 68% yield (0.0193 g, 0.068 mmol) using **conditions B**; purified by column chromatography on silica gel (Supelco Art. 9385 silica 30/64; eluent: CH_2Cl_2–MeOH = 80:1 to 40:1). Beige oil; **TLC**, *Rf* = 0.33 (CH_2Cl_2–MeOH = 40:1, ninhydrin); **IR** (neat) 3376 (NH_2), 3310 (NH_2), 3302 (NH_2), 2980, 2933, 1740, 1721, 1619 (NH_2), 1598, 1370, 1250, 1212, 1150, 1121, 842, 735 cm^{-1}; **^{1}H NMR** (300 MHz, $CDCl_3$) δ 6.96 (dd, J = 7.78, 0.88 Hz, 1H), 6.77 (dt, J = 9.05, 1.86 Hz, 1H), 3.62 (d, J = 17.4 Hz, 2H), 3.08 (d, J = 17.4 Hz, 2H), 2.60 (br s, 2H), 1.36 (s, 9H). **^{13}C NMR** (75 MHz, $CDCl_3$) δ 197.1, 170.4, 168.1 (dd, J = 259.3, 11.8 Hz), 160.3 (dd, J = 266.7, 14.3 Hz), 156.5 (dd, J = 11.7, 3.9 Hz), 119.2 (dd, J = 13.5, 2.3 Hz), 109.6 (dd, J = 22.5, 4.3 Hz), 104.2 (dd, J = 27.0, 22.7 Hz), 83.4, 68.6, 41.3, 27.8. **^{19}F NMR** (282 MHz, $CDCl_3$) δ -96.74 (dt, J = 16.8, 6.97 Hz, 1F), -107.84 (dd, J = 13.97, 9.03 Hz, 1F); **HRMS** (ESI+) *m/z* calcd for $[C_{14}H_{15}NF_2O_3+H]^+$ 284.1093, found 284.1094 (+0.4 ppm).

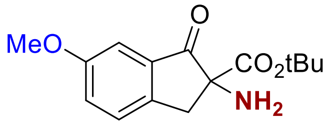

***tert*-Butyl 2-amino-6-methoxy-1-oxo-2,3-dihydro-1*H*-indene-2-carboxylate (2q):** obtained in 88% yield (0.0244 g, 0.088 mmol) using **conditions B**; purified by column chromatography on silica gel (Supelco Art. 9385 silica 30/64; eluent: CH_2Cl_2–MeOH = 80:1 to 40:1). Brown oil; **TLC**, *Rf* = 0.28 (CH_2Cl_2–MeOH = 40:1, ninhydrin); **IR** (neat) 3357 (NH_2), 3300 (NH_2), 2982, 2921, 1735, 1711, 1614 (NH_2), 1491, 1147, 1022, 853 cm^{-1}; **^{1}H NMR** (300 MHz, $CDCl_3$) δ 7.39 – 7.30 (m, 1H), 7.30 – 7.16 (m, 2H), 3.84 (s, 3H), 3.55 (d, *J* = 16.6 Hz, 1H), 2.97 (d, *J* = 16.6 Hz, 1H), 2.03 (s, 2H), 1.34 (s, 9H); **^{13}C NMR** (75 MHz, $CDCl_3$) δ 202.9, 171.6, 159.8, 145.7, 135.6, 127.1, 125.1, 106.2, 82.7, 68.5, 55.7, 40.9, 27.9; **HRMS** (ESI+) *m/z* calcd for $[C_{15}H_{19}NO_4+Na]^+$ 300.1206, found 300.1197 (−3.0 ppm).

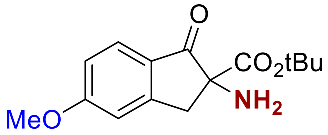

***tert*-Butyl 2-amino-5-methoxy-1-oxo-2,3-dihydro-1*H*-indene-2-carboxylate (2r):** obtained in 65% yield (0.0180 g, 0.065 mmol) using **conditions A**; purified by column chromatography on silica gel (Supelco Art. 9385 silica 30/64; eluent: CH_2Cl_2–MeOH = 80:1 to 40:1). Yellowish oil; **TLC**, *Rf* = 0.28 (CH_2Cl_2–MeOH = 40:1, ninhydrin); **IR** (neat) 3357 (NH_2), 3300 (NH_2), 2920, 1735, 1711, 1614 (NH_2), 1491, 1279, 1251, 1221, 1147, 1022, 853 cm^{-1}; **^{1}H NMR** (300 MHz, $CDCl_3$) δ 7.72 (d, *J* = 8.5 Hz, 1H), 6.99 – 6.85 (m, 2H), 3.90 (s, 3H), 3.59 (d, *J* = 17.0 Hz, 1H), 2.99 (d, *J* = 17.0 Hz, 1H), 2.05 (s, 2H), 1.36 (s, 9H); **^{13}C NMR** (75 MHz, $CDCl_3$) δ 200.9, 171.7, 166.1, 155.9, 127.6, 126.9, 115.9, 109.6, 82.5, 68.2, 55.8, 41.5, 27.9; **HRMS** (ESI+) *m/z* calcd for $[C_{15}H_{19}NO_4+H]^+$ 278.1387, found 278.1384 (−1.1 ppm).

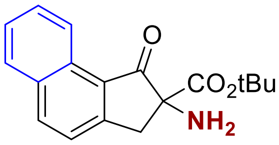

***tert*-Butyl 2-amino-1-oxo-2,3-dihydro-1*H*-cyclopenta[a]naphthalene-2-carboxylate (2s):** obtained in 85% yield (0.0268 g, 0.085 mmol) using **conditions B**; purified by column chromatography on silica gel (Supelco Art. 9385 silica 30/64; eluent: CH_2Cl_2–MeOH = 80:1 to 40:1). Light-yellow solid, **MP**: 99.9-101.3 °C; **TLC**, *Rf* = 0.49 (CH_2Cl_2–MeOH = 40:1, ninhydrin); **IR** (neat) 3378 (NH_2), 3315 (NH_2), 2975, 1724, 1690, 1516, 1367, 1214, 1146, 1086, 847, 819, 795, 766 cm^{-1}; **^{1}H NMR** (300 MHz, $CDCl_3$, 298 K): δ 9.04 (d, *J* = 8.30 Hz, 1H), 8.08 (d, *J* = 8.39 Hz, 1H), 7.90 (d, *J* = 8.13 Hz, 1H), 7.68 (t, *J* = 3.30 Hz, 1H), 7.59-7.50 (m, 2H), 3.74 (d, *J* = 17.33 Hz, 1H), 3.15 (d, *J* = 17.33 Hz, 1H), 2.20 (br s, 2H), 1.34 (s, 9H). **^{13}C NMR** (75 MHz, $CDCl_3$, 298 K): δ 202.9, 171.6, 156.3, 136.9, 133.0, 129.9, 129.3, 128.5,

128.4, 127.0 124.1, 123.7, 82.7, 68.1, 41.7, 27.9; **HRMS** (ESI+) *m/z* calcd for $[C_{18}H_{19}NO_3+H]^+$ 298.1438, found 298.1435 (-1.0 ppm).

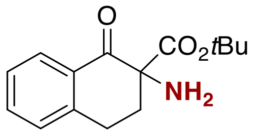

***tert*-Butyl 2-amino-1-oxo-1,2,3,4-tetrahydronaphthalene-2-carboxylate (4a):** obtained in 62% yield (0.0162 g, 0.062 mmol) using **conditions A**; purified by column chromatography on silica gel (Supelco Art. 9385 silica 30/64; eluent: CH_2Cl_2–MeOH = 80:1 to 40:1). Colorless oil; **TLC**, R_f = 0.34 (CH_2Cl_2–MeOH = 40:1, ninhydrin); **IR** (neat) 3379 (NH_2), 3318 (NH_2), 2977, 2909, 1723, 1682, 1598 (NH_2), 1456, 1368, 1284, 1257, 1239, 1154, 1113, 920, 842, 739, 715, 601 cm^{-1}; **^{1}H NMR** (300 MHz, $CDCl_3$, 298 K): δ 8.05 (dd, *J* = 7.8, 1.5 Hz, 1H), 7.48 (td, *J* = 7.5, 1.5 Hz, 1H), 7.32 (t, *J* = 7.6 Hz, 1H), 7.23 (d, *J* = 7.7 Hz, 1H), 3.12–2.93 (m, 2H), 2.64–2.44 (m, 1H), 2.26–2.05 (m, 3H), 1.34 (s, 9H); **^{13}C NMR** (75 MHz, $CDCl_3$, 298 K): δ 195.1, 172.4, 143.4, 133.6, 131.6, 128.8, 128.0, 126.9, 82.8, 65.2, 34.8, 27.9, 26.0; **HRMS** (ESI+) *m/z* calcd for $[C_{15}H_{19}NO_3+H]^+$ 262.1438, found 262.1437 (−0.3 ppm).

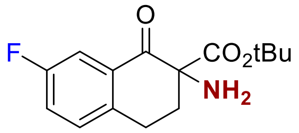

***tert*-Butyl 2-amino-7-fluoro-1-oxo-1,2,3,4-tetrahydronaphthalene-2-carboxylate (4b):** obtained in 63% yield (0.0190 g, 0.063 mmol) using **conditions A**; purified by column chromatography on silica gel (Supelco Art. 9385 silica 30/64; eluent: CH_2Cl_2–MeOH = 80:1 to 40:1). Reddish oil; **TLC**, R_f = 0.65 (CH_2Cl_2–MeOH = 40:1, ninhydrin); **IR** (neat) 3382 (NH_2), 3313 (NH_2), 2978, 2933, 1728, 1690, 1612 (NH_2), 1589, 1492, 1448, 1369, 1241, 1146, 889, 840, 779, 730, 695 cm^{-1}; **^{1}H NMR** (300 MHz, $CDCl_3$, 298 K): δ 7.72-7.69 (m, 1H), 7.25-7.16 (m, 2H), 3.00-2.96 (m, 2H), 2.52 (dt, *J* = 13.58, 4.89 Hz, 1H), 2.18-2.09 (m, 3H), 1.34 (s, 9H). **^{13}C NMR** (75 MHz, $CDCl_3$, 298 K): δ 194.2, 172.2, 161.5 (d, *J* = 247.5 Hz), 139.1 (d, *J* = 3.6 Hz), 133.3 (d, *J* = 6.3 Hz), 130.5 (d, *J* = 7.0 Hz), 120.9 (d, *J* = 22.2 Hz), 113.7 (d, *J* = 21.6 Hz), 83.1, 65.0, 34.9, 27.9, 25.4; **^{19}F NMR** (282 MHz, $CDCl_3$, 298 K): δ -114.92 – -115.00 (m, 1F); HRMS (ESI+) *m/z* calcd for $[C_{15}H_{18}FNO_3+H]^+$ 280.1343, found 280.1344 (+0.4 ppm).

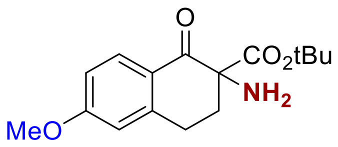

***tert*-Butyl 2-amino-6-methoxy-1-oxo-1,2,3,4-tetrahydronaphthalene-2-carboxylate (4c):** obtained in 78% yield (0.0234 g, 0.078 mmol) using **conditions B**; purified by column chromatography on silica gel (Supelco Art. 9385 silica 30/64; eluent: CH_2Cl_2–MeOH = 80:1 to 40:1). Yellow oil; **TLC**, R_f = 0.58 (CH_2Cl_2–MeOH = 40:1, ninhydrin); **IR** (neat) 3380 (NH_2), 3313 (NH_2), 2976, 2934, 1726, 1675, 1597,

1367, 1247, 1149, 926, 843, 776 cm^{-1}; ^{1}**H NMR** (300 MHz, $CDCl_3$, 298 K): δ 8.02 (d, *J* = 8.77 Hz, 1H), 6.84 (dd, *J* = 8.77, 2.48 Hz, 1H), 6.67 (d, *J* = 2.48 Hz, 1H), 3.85 (s, 3H), 2.99 (dd, *J* = 7.31, 5.12 Hz, 2H), 2.52 (dt, *J* = 13.5, 5.12 Hz, 1H), 2.38 (br s, 2H), 2.13 (dt, *J* = 15.2, 6.78 Hz, 1H), 1.36 (s, 9H); ^{13}**C NMR** (75 MHz, CDC13, 298 K): δ 193.6, 172.4, 163.9, 146.0, 130.6, 125.0, 113.5, 112.6, 82.7, 65.0, 55.6, 34.7, 27.9, 26.4; HRMS (ESI+) *m/z* calcd for $[C_{16}H_{21}NO_4+H]^+$ 292.1543, found 292.1545 (+0.7 ppm).

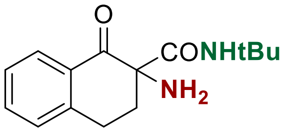

2-Amino-*N*-(*tert*-butyl)-1-oxo-1,2,3,4-tetrahydronaphthalene-2-carboxamide (4d): obtained in 40% yield (0.0104 g, 0.040 mmol) using **conditions C**; purified by column chromatography on silica gel (Supelco Art. 9385 silica 30/64; eluent: CH_2Cl_2–MeOH = 80:1 to 40:1). White solid, **MP**: 99.6-101.7 °C; **TLC**, *Rf* = 0.50 (CH_2Cl_2–MeOH = 40:1, ninhydrin); **IR** (neat) 3332 (NH_2), 2958, 2875, 1678, 1653, 1597, 1537, 1360, 1216, 1186, 1110, 1021, 937, 8887, 757, 731, 659, 570 cm^{-1}; ^{1}**H NMR** (300 MHz, $CDCl_3$) δ 8.06 (dd, *J* = 7.8, 1.4 Hz, 1H), 7.47 (td, *J* = 7.5, 1.5 Hz, 1H), 7.41 (s, 1H), 7.31 (t, *J* = 7.4 Hz, 1H), 7.23 (d, *J* = 7.6 Hz, 1H), 3.38 (ddd, *J* = 17.1, 12.3, 4.8 Hz, 1H), 2.89 (dt, *J* = 16.8, 4.1 Hz, 1H), 2.43 (ddd, *J* = 13.2, 4.8, 3.3 Hz, 1H), 2.23 – 1.84 (br s, 3H), 1.33 (s, 9H); ^{13}**C NMR** (126 MHz, $CDCl_3$) δ 199.6, 171.1, 144.7, 133.7, 132.0, 128.9, 127.8, 126.8, 64.5, 50.9, 35.5, 28.7, 26.6; **HRMS** (ESI+) *m/z* calcd for $[C_{15}H_{20}N_2O_2+H]^+$ 261.1598, found 261.1602 (−1.5 ppm).

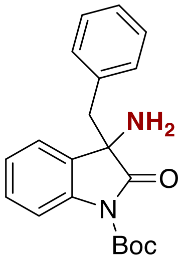

***tert*-Butyl 3-amino-3-benzyl-2-oxoindoline-1-carboxylate (5a):** obtained in 62% yield (0.0210 g, 0.062 mmol) using **conditions A**; purified by column chromatography on silica gel (Supelco Art. 9385 silica 30/64; eluent: CH_2Cl_2–MeOH = 80:1 to 40:1). Yellow oil; **TLC**, R_f = 0.23 (CH_2Cl_2–MeOH = 40:1, ninhydrin); **IR** (neat) 3368 (NH_2), 3306 (NH_2), 2980, 1791, 1765, 1728, 1607 (NH_2), 1288, 1248, 1145, 747, 730, 699 cm^{-1}; ^{1}**H NMR** (300 MHz, $CDCl_3$, 298 K): δ 7.60 (d, *J* = 7.9 Hz, 1H), 7.36–7.23 (m, 2H), 7.22–7.14 (m, 1H), 7.14–7.05 (m, 3H), 6.89–6.79 (m, 2H), 3.13 (d, *J* = 12.80 Hz, 1H), 3.08 (d, *J* = 12.80 Hz, 1H), 2.01 (s, 2H), 1.57 (s, 9H); ^{13}**C NMR** (75 MHz, $CDCl_3$, 298 K): δ 179.4, 148.8, 139.3, 134.2, 130.2, 129.8, 129.4, 127.9, 127.1, 124.6, 124.2, 115.0, 84.3, 62.9, 46.8, 28.1; **HRMS** (ESI+) *m/z* calcd for $[C_{20}H_{22}N_2O_3+CH_3OH+H]^+$ 371.1965, found 371.1964 (−0.35 ppm); also detected *m/z* calcd for $[C_{20}H_{22}N_2O_3+Na]^+$ 361.1523, found 361.1523 (+/- 0 ppm).

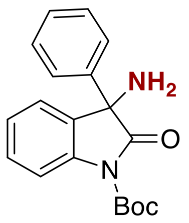

***tert*-butyl 3-amino-2-oxo-3-phenylindoline-1-carboxylate (5b):** obtained in 90% yield (0.0292 g, 0.090 mmol) using **conditions A**; purified by column chromatography on silica gel (Supelco Art. 9385 silica 30/64; eluent: CH_2Cl_2–MeOH = 80:1 to 40:1). Yellow oil; **TLC**, R_f = 0.33 (CH_2Cl_2–MeOH = 40:1, ninhydrin); **IR** (neat) 3371 (NH_2), 3305 (NH_2), 2981, 1790, 1763, 1728, 1605 (NH_2), 1286, 1247, 1144, 727, 696 cm^{-1}; **^{1}H NMR** (300 MHz, $CDCl_3$, 298 K): δ 7.96 (d, J = 8.2 Hz, 1H), 7.43–7.41 (m, 1H), 7.41–7.29 (m, 6H), 7.20 (td, J = 7.5, 1.0 Hz, 1H), 2.16 (s, 2H), 1.67 (s, 9H); **^{13}C NMR** (75 MHz, $CDCl_3$, 298 K): δ 178.6, 149.4, 140.9, 139.3, 132.3, 129.6, 128.8, 128.2, 125.9, 125.3, 125.0, 115.4, 84.8, 64.1, 28.2; **HRMS** (ESI+) *m/z* calcd for $[C_{19}H_{20}N_2O_3+CH_3OH+H]^+$ 357.1809, found 357.1805 (−1.06 ppm); also detected *m/z* calcd for $[C_{19}H_{20}N_2O_3+Na]^+$ 347.1366, found 347.1368 (+0.5 ppm).

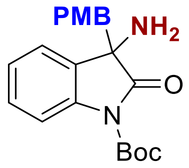

***tert*-Butyl 3-amino-3-(4-methoxybenzyl)-2-oxoindoline-1-carboxylate (5c):** obtained in 44 % yield (0.0162 g, 0.044 mmol) using **conditions A**; purified by column chromatography on silica gel (Supelco Art. 9385 silica 30/64; eluent: CH_2Cl_2–MeOH = 80:1 to 40:1). Yellowish oil; **TLC**, *Rf* = 0.22 (CH_2Cl_2–MeOH = 40:1, ninhydrin); **IR** (neat) 3365 (NH_2), 3294 (NH_2), 2928, 1790, 1766, 1727, 1610 (NH_2), 1512, 1290, 1245, 1145, 839, 823, 753 cm^{-1}; **^{1}H NMR** (300 MHz, $CDCl_3$) δ 7.66 – 7.55 (m, 1H), 7.30 (ddd, J = 8.3, 7.5, 1.5 Hz, 2H), 7.17 (td, J = 7.5, 1.1 Hz, 1H), 6.81 – 6.70 (m, 2H), 6.65 – 6.58 (m, 2H), 3.70 (s, 3H), 3.16 – 2.94 (m, 2H), 1.88 (s, 2H), 1.56 (s, 9H); **^{13}C NMR** (75 MHz, $CDCl_3$) δ 179.6, 158.7, 148.8, 139.3, 131.2, 130.0, 129.4, 126.3, 124.6, 124.2, 115.0, 113.3, 84.2, 62.9, 55.2, 45.9, 28.2; **HRMS** (ESI+) *m/z* calcd for $[C_{21}H_{24}N_2O_4+CH_3OH+H]^+$ 401.2071, 401.2065 found (-1.5 ppm).

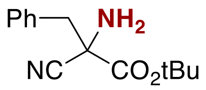

***tert*-Butyl 2-amino-2-cyano-3-phenylpropanoate (6a):** obtained in 60% yield (0.0111 g, 0.045 mmol) using **conditions C**; using KO*t*-Bu (0.0458 g, 0.400 mmol, 4 equiv) as base; purified by column chromatography on silica gel (Supelco Art. 9385 silica 30/64; eluent: CH_2Cl_2–MeOH = 80:1 to 40:1). Colorless oil; **TLC**, R_f = 0.59 (CH_2Cl_2–MeOH = 40:1, ninhydrin); **IR** (neat) 3385 (NH_2), 3317 (NH_2), 2981, 1737, 1604 (NH_2), 1456, 1370, 1253, 1151, 700 cm^{-1}; **^{1}H NMR** (300 MHz, $CDCl_3$, 298 K): δ 7.39–7.30 (m, 5H), 3.29 (d, J = 13.5 Hz, 1H), 3.12 (d, J = 13.5 Hz, 1H), 1.88 (s, 2H), 1.51 (s, 9H); **^{13}C NMR** (75 MHz, $CDCl_3$, 298 K): δ 166.9, 133.1, 130.6, 128.9, 128.3, 119.7, 85.1, 58.2, 44.6, 27.9;

HRMS (ESI+) *m/z* calcd for $[C_{14}H_{18}N_2O_2+H]^+$ 247.1441, found 247.1442 (+0.38 ppm); also detected *m/z* calcd for $[C_{14}H_{18}N_2O_2+Na]^+$ 269.1260, found 269.1259 (−0.6 ppm).

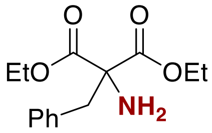

Diethyl 2-amino-2-benzylmalonate (6b):[5] Following a similar procedure using **conditions C**, using KO*t*-Bu (0.0458 g, 0.400 mmol, 4 equiv) as base, 6 h: 76% yield (0.0202 g, 0.076 mmol); purified by column chromatography on silica gel (Supelco Art. 9385 silica 30/64; eluent: CH_2Cl_2–MeOH = 80:1 to 40:1). Pale yellow oil; **TLC**, R_f = 0.47 (CH_2Cl_2–MeOH = 40:1, ninhydrin); **IR** (neat) 3386 (NH_2), 3321 (NH_2), 2981, 1735, 1712, 1603 (NH_2), 1454, 1206, 1185, 1030, 863, 745, 701, 599, 555 cm^{-1}; **^{1}H NMR** (300 MHz, $CDCl_3$, 298 K): δ 7.35–7.24 (m, 3H), 7.22–7.13 (m, 2H), 4.25 (q, *J* = 7.1 Hz, 4H), 3.33 (s, 2H), 1.94 (s, 2H), 1.29 (t, *J* = 7.1 Hz, 6H); **^{13}C NMR** (75 MHz, $CDCl_3$, 298 K): δ 171.2, 135.1, 130.3, 128.6, 127.5, 66.4, 62.1, 41.2, 14.2; **HRMS** (ESI+) *m/z* calcd for $[C_{14}H_{19}NO_4+H]^+$ 266.1387, found 266.1388 (+0.4 ppm).

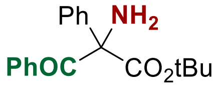

***tert*-Butyl 2-amino-3-oxo-2,3-diphenylpropanoate (6c):** obtained using **conditions C**, using KO*t*-Bu (0.0458 g, 0.400 mmol, 4 equiv) as base; isolated as colorless oil in 49% yield (0.0157 g, 0.049 mmol); purified by column chromatography on silica gel (Supelco Art. 9385 silica 30/64; eluent: CH_2Cl_2–MeOH = 80:1 to 40:1). Colorless oil; **TLC**, R_f = 0.23 (CH_2Cl_2–MeOH = 40:1, ninhydrin); **IR** (neat) 3376 (NH_2), 2981, 2935, 1720, 1646, 1602 (NH_2), 1515, 1483, 1257, 843, 714, 697, 689 cm^{-1}; **^{1}H NMR** (300 MHz, $CDCl_3$, 298 K): δ 7.81-7.78 (m, 2H), 7.55-7.51 (m, 2H), 7.46-7.40 (m, 1H), 7.36-7.28 (m, 5H), 2.27 (br s, 2H), 1.33 (s, 9H); **^{13}C NMR** (75 MHz, $CDCl_3$, 298 K): δ 197.5, 170.3, 139.6, 135.2, 132.7, 130.0, 128.7, 128.3, 128.2, 127.8, 127.3, 126.8, 83.3, 73.3, 28.0, 27.7; **HRMS** (ESI+) *m/z* calcd for $[C_{19}H_{21}NO_3+H]^+$ 312.1594, found 312.1592 (-0.6 ppm).

[5] L.-B. Zhang, D.-X. Wang, L. Zhao, M.-X. Wang, *J. Org. Chem.* **2012**, *77*, 5584–5591.

3.3 Asymmetric α-Amination of 1a (Proof-of-Concept)

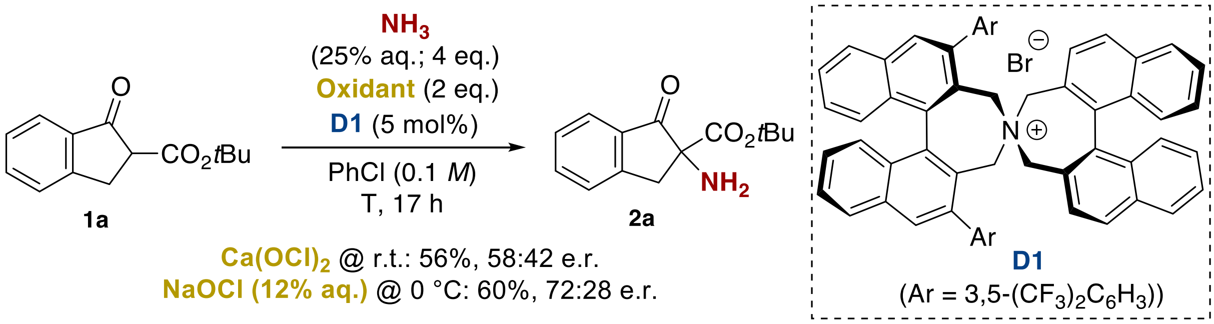

To a stirring mixture (300 rpm) of **1a** (0.0232 g, 0.100 mmol) and **D1** (0.0054 g, 0.005 mmol) in 1.0 mL chlorobenzene was added a solution of aqueous NH_3 (25wt%, 29.94 µL, 0.400 mmol) within 20 seconds at 0 °C. Under rapid stirring (1200 rpm) aqueous NaOCl (12%, 99.3 µL, 0.200 mmol) was added in one portion and the suspension was stirred for 17 h at 0 °C. The reaction mixture was diluted with 3 mL CH_2Cl_2 and filtered over a short pad of anhydrous Na_2SO_4/silica (diameter 2 cm, height Na_2SO_4 = 3 cm, silica = 1 cm) and washed with additional 35 mL CH_2Cl_2 and 0.5 mL MeOH. The solvents were removed *in vacuo* and the crude product was analyzed by qNMR using $MeNO_2$ as external standard (133.33 µL, 0.667 equiv., 0.5 *M* in $CDCl_3$) which gave **2a** in 60% NMR yield. Enantiomeric ratio of **2a** was determined to be 72:28 e.r. by HPLC analysis (CHIRALPAK AD-H column, *n*-hexane/2-propanol = 94/6, flow rate = 1.0 mL/min, 10 °C, t_R= 15.9 min (*minor*), t_R = 18.6 min (*major*); see below chart; left: *rac*-**2a**, right: enantioenriched-**2a**).[6]

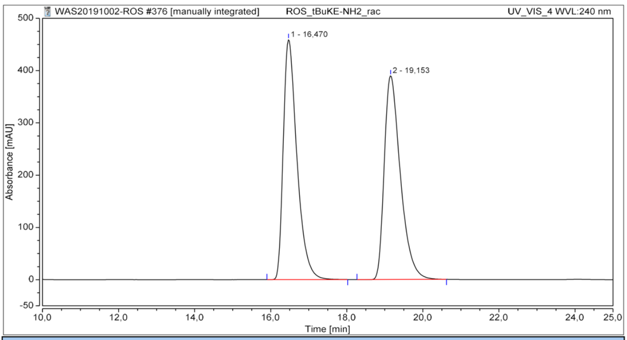

Integration Results

No.	Peak Name	Retention Time min	Area mAU*min	Height mAU	Relative Area %	Relative Height %	Amount n.a.
1		16,470	191,015	459,198	49,96	54,09	n.a.
2		19,153	191,302	389,804	50,04	45,91	n.a.
**Total:**			**382,317**	**849,002**	**100,00**	**100,00**	

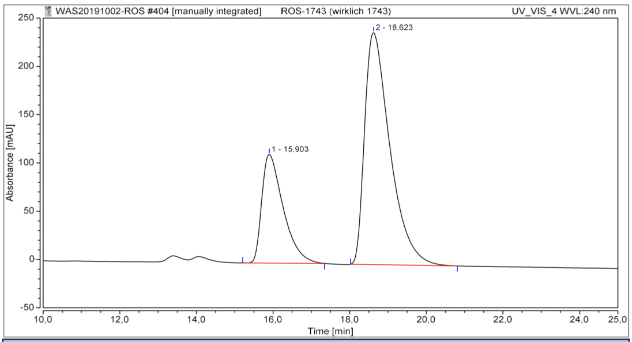

Integration Results

No.	Peak Name	Retention Time min	Area mAU*min	Height mAU	Relative Area %	Relative Height %	Amount n.a.
1		15,903	72,800	112,495	28,35	31,91	n.a.
2		18,623	184,005	240,063	71,65	68,09	n.a.
**Total:**			**256,805**	**352,558**	**100,00**	**100,00**	

[6] B. Maji, M. Baidya, H. Yamamoto, *Chem. Sci.* **2014**, *5*, 3941–3945.

4. NMR Spectra

2a, ^{1}H NMR (700 MHz, CDCl$_3$, 298 K)

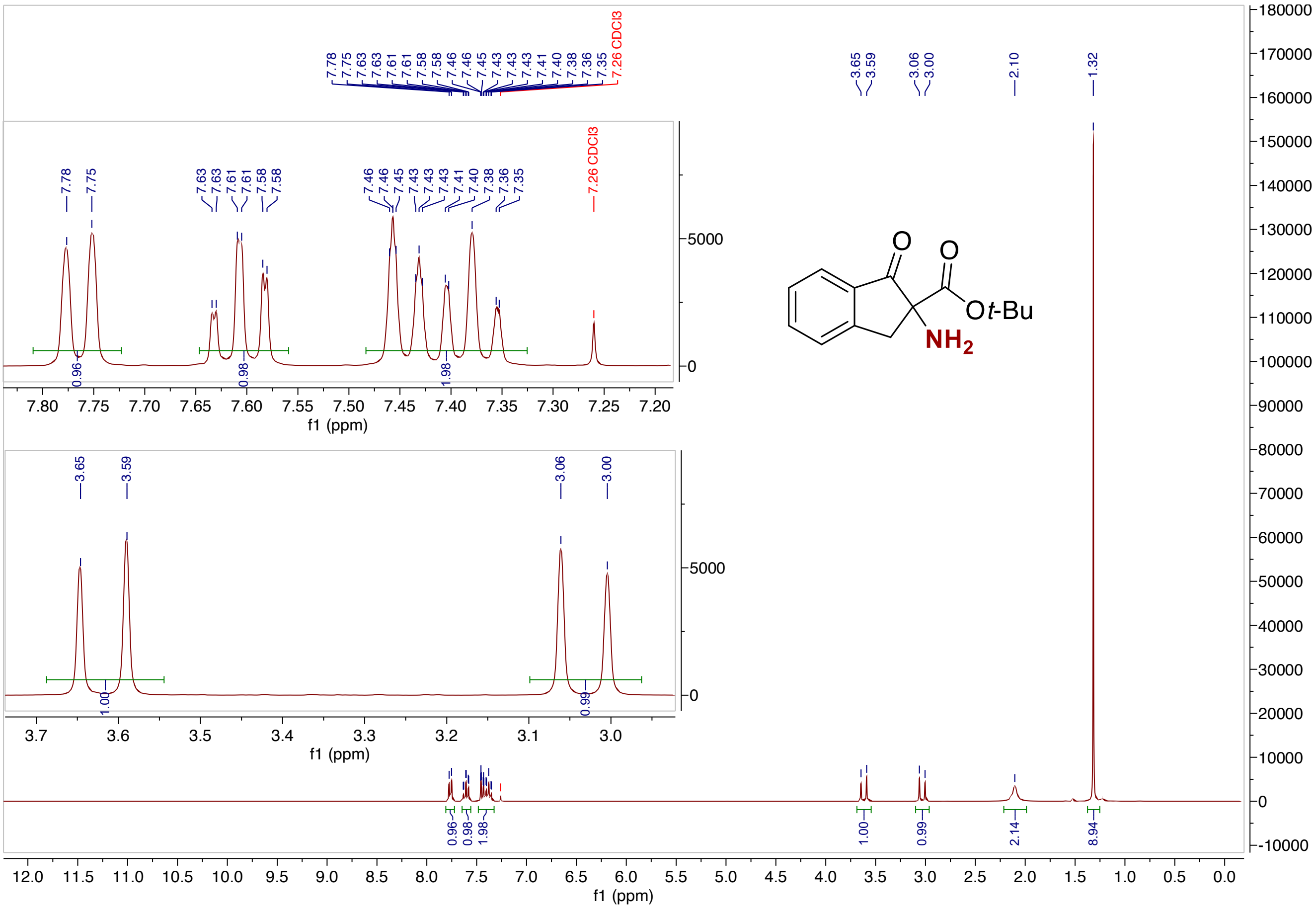

2a, ^{13}C NMR (75 MHz, CDCl$_3$, 298 K)

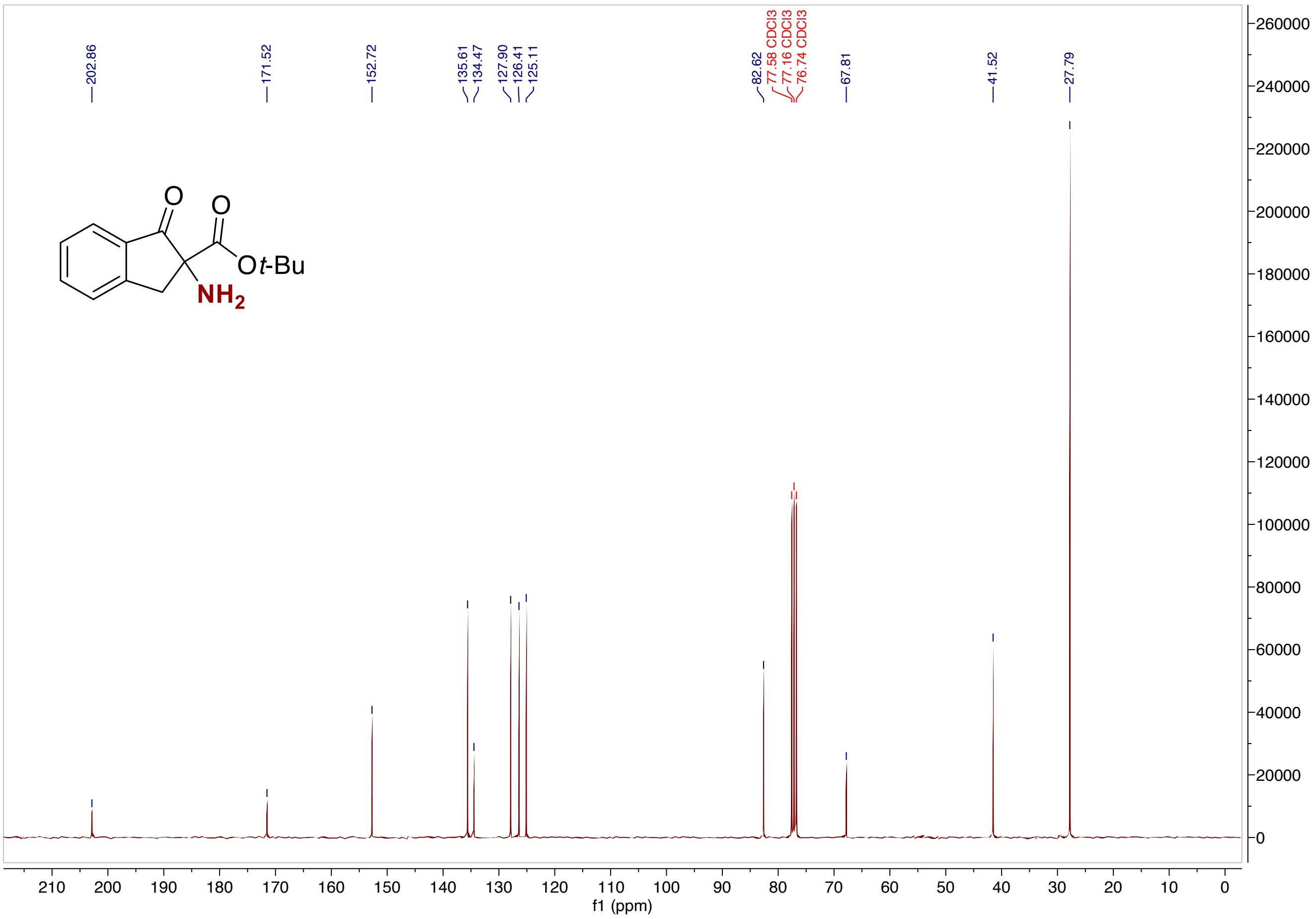

2b, ^{1}H NMR (300 MHz, $CDCl_3$, 298 K)

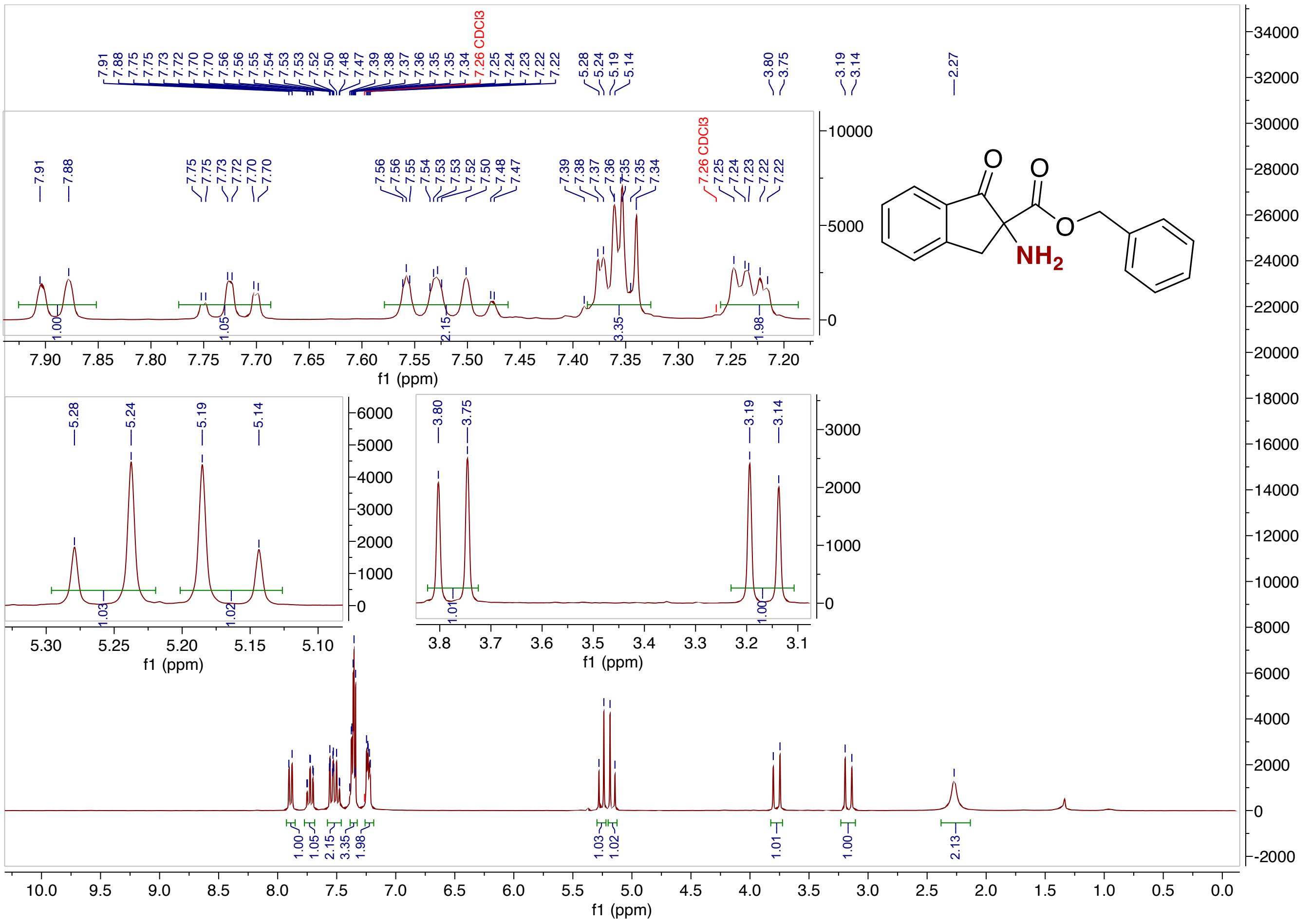

2b, ^{13}C NMR (75 MHz, $CDCl_3$, 298 K)

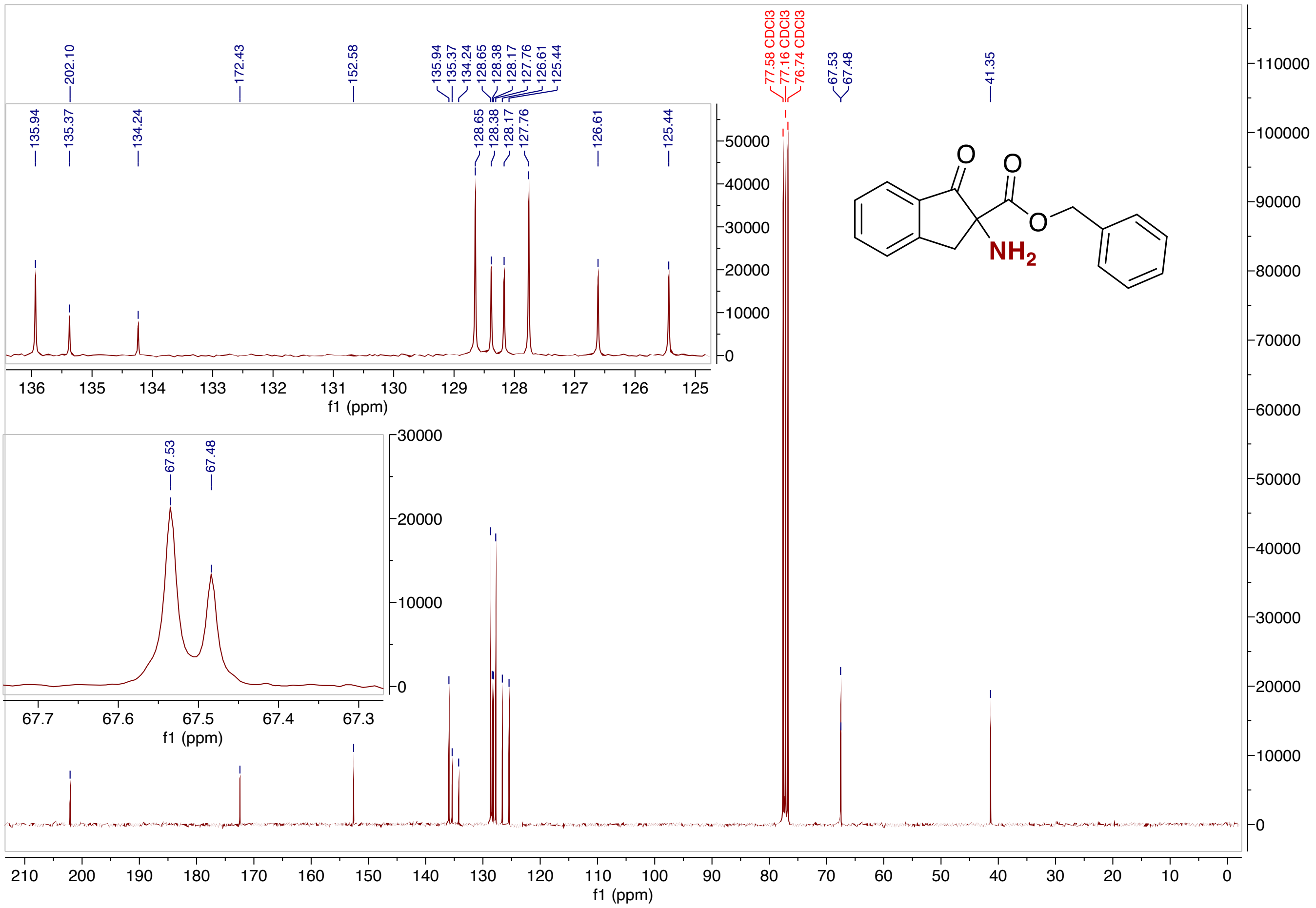

2c, ^{1}H NMR (300 MHz, CDCl$_3$, 298 K)

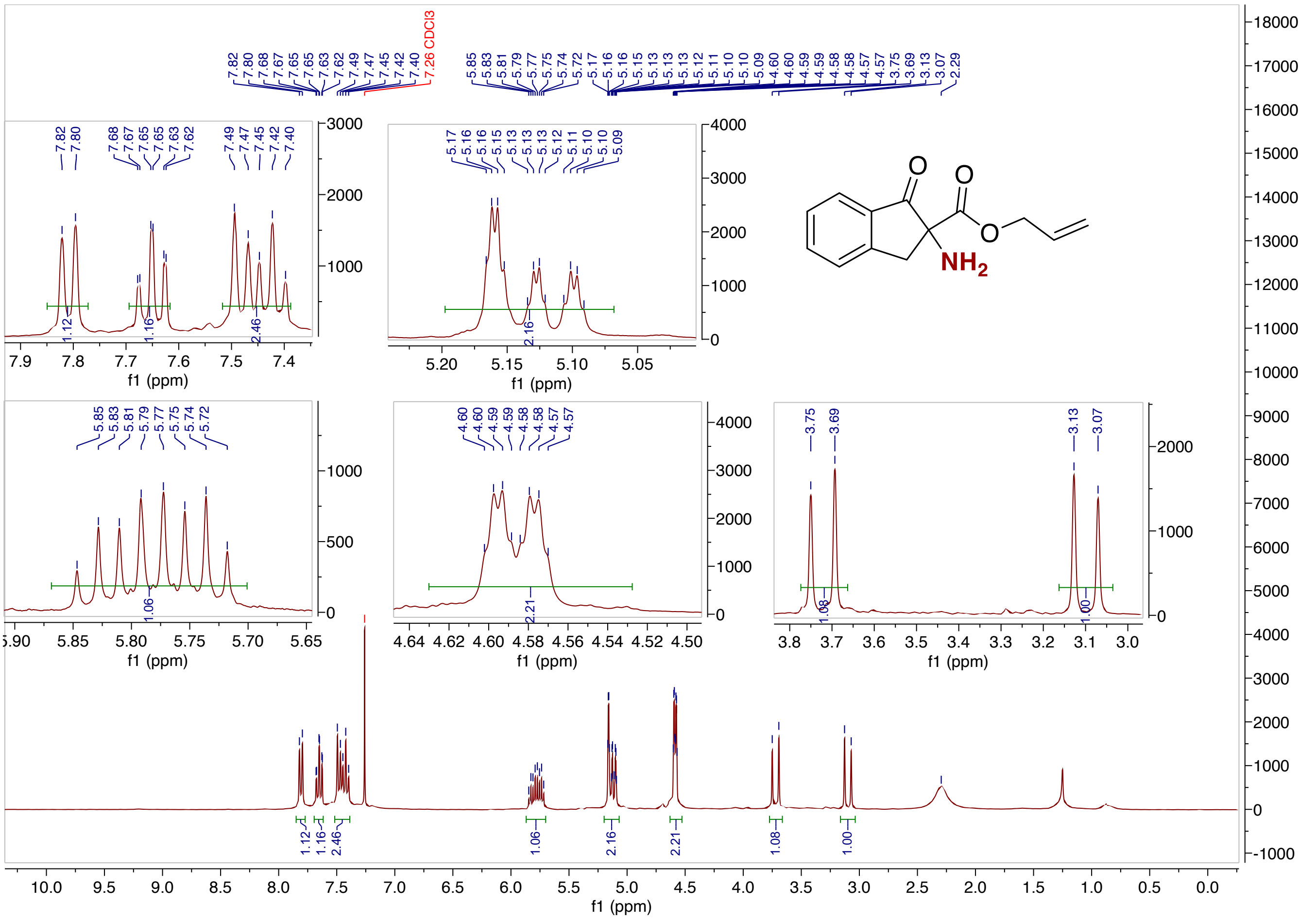

2c, ^{13}C NMR (75 MHz, CDCl$_3$, 298 K)

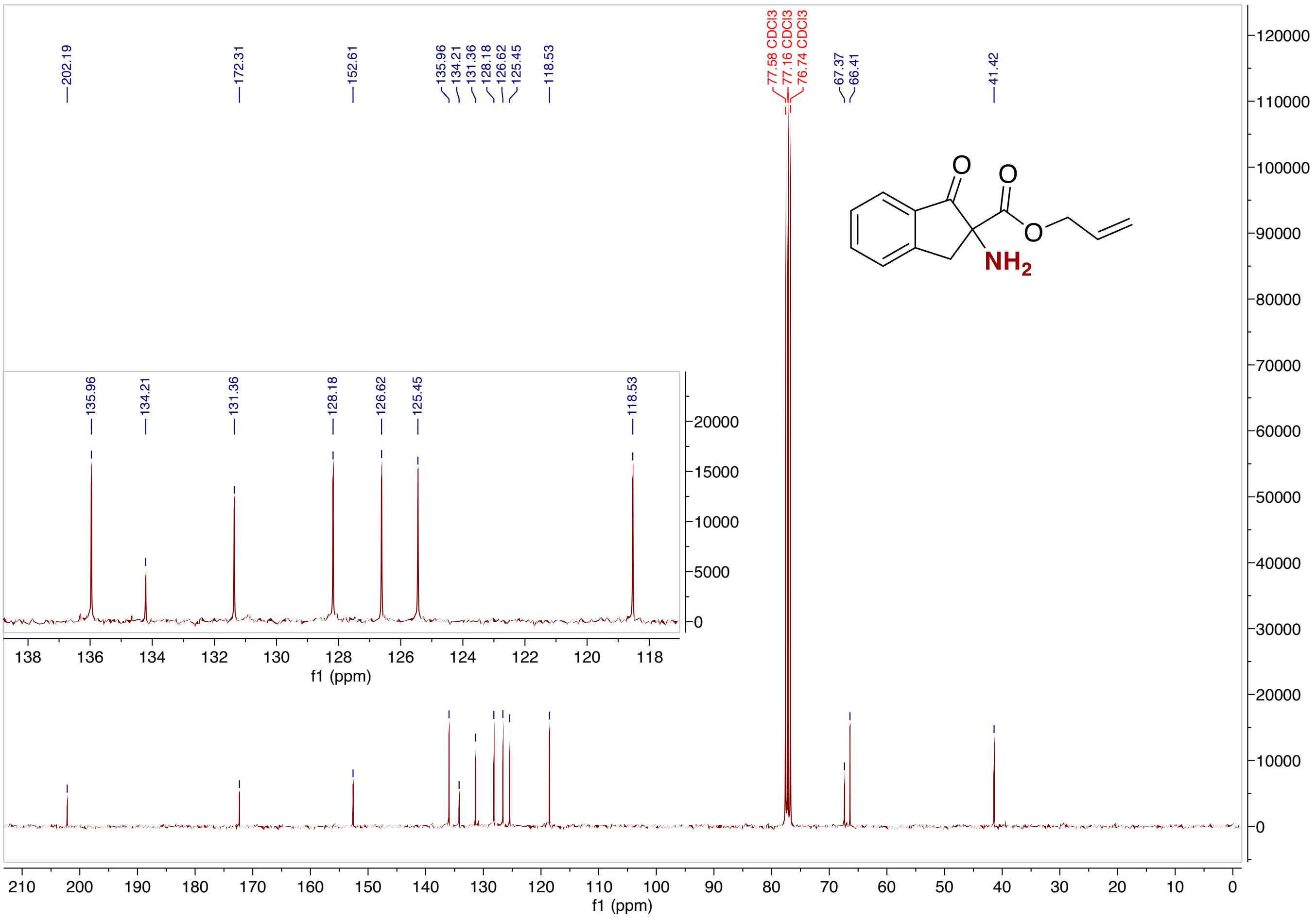

2d, ^{1}H NMR (300 MHz, $CDCl_3$, 298 K)

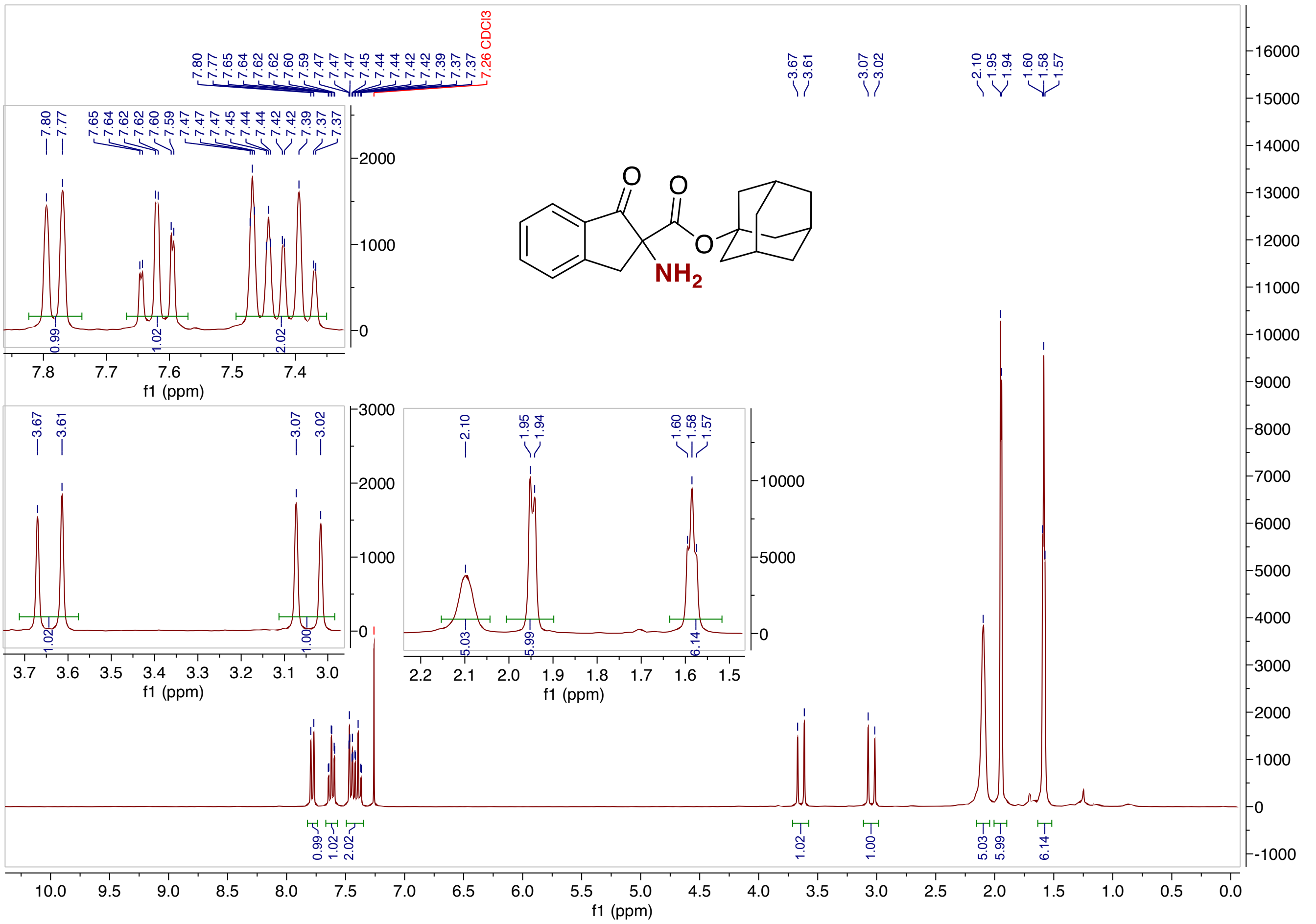

2d, ^{13}C NMR (75 MHz, $CDCl_3$, 298 K)

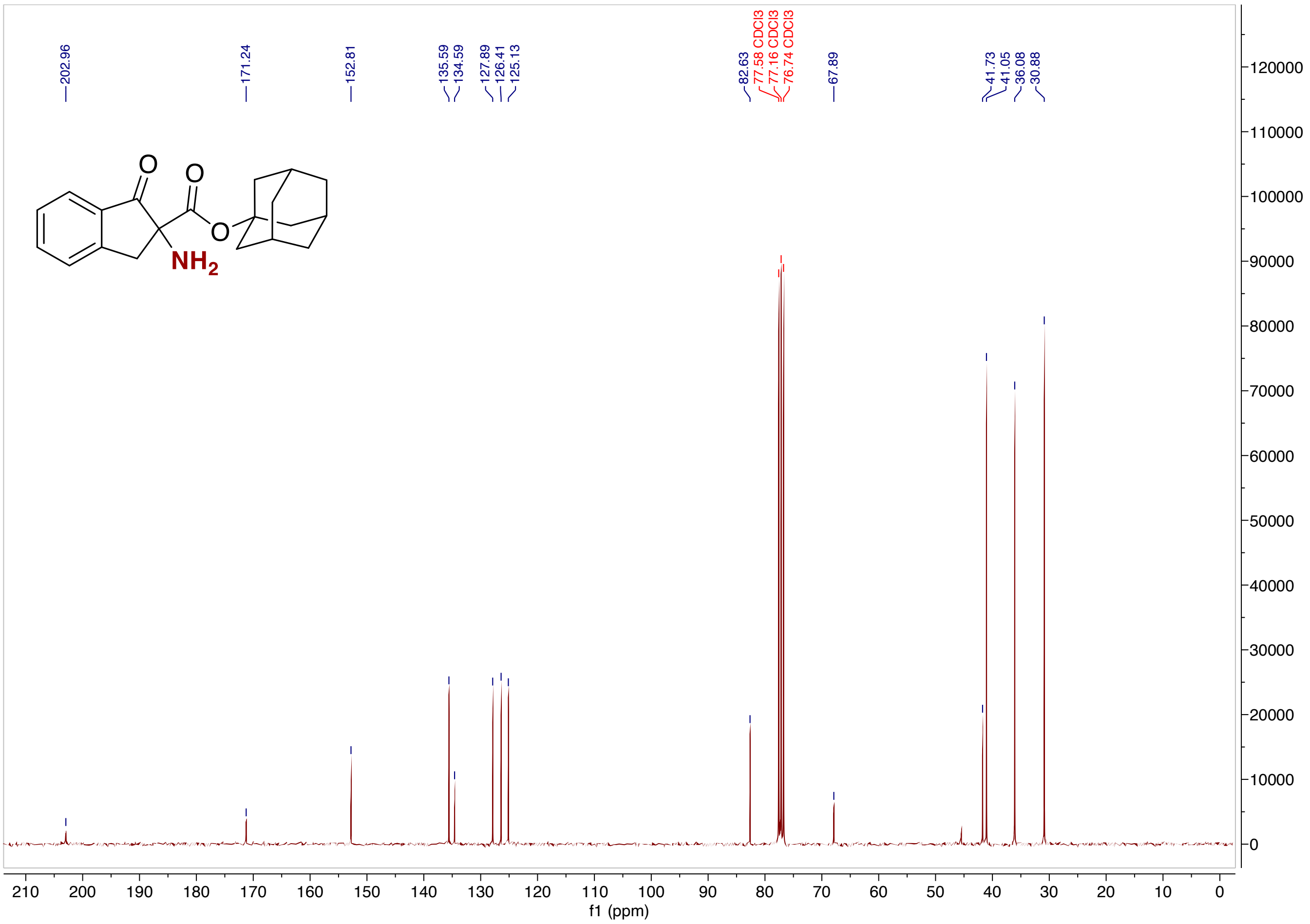

2e, ^{1}H NMR (300 MHz, $CDCl_3$, 298 K)

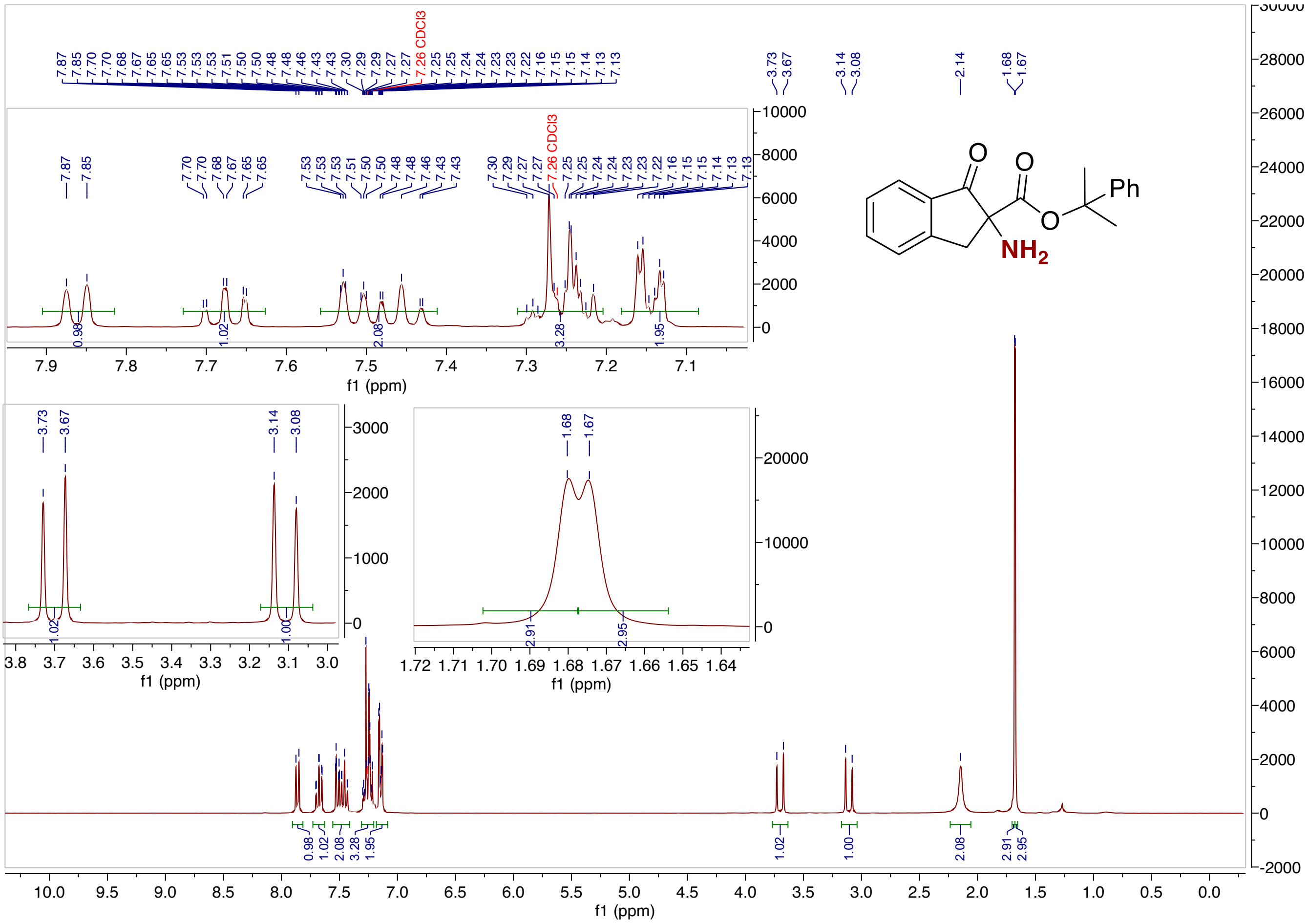

2e, ^{13}C NMR (75 MHz, $CDCl_3$, 298 K)

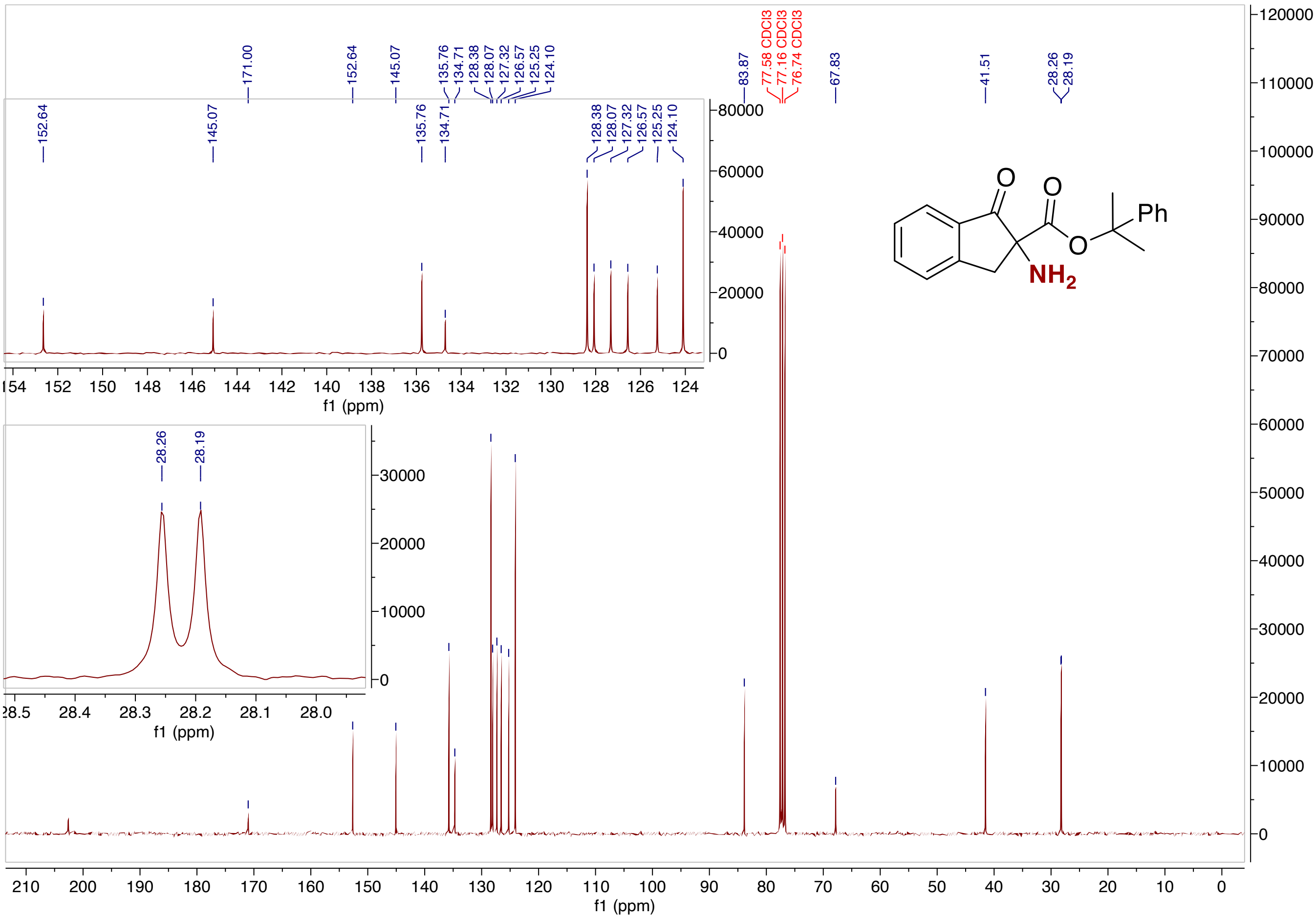

2f, ^{1}H NMR (300 MHz, $CDCl_3$, 298 K)

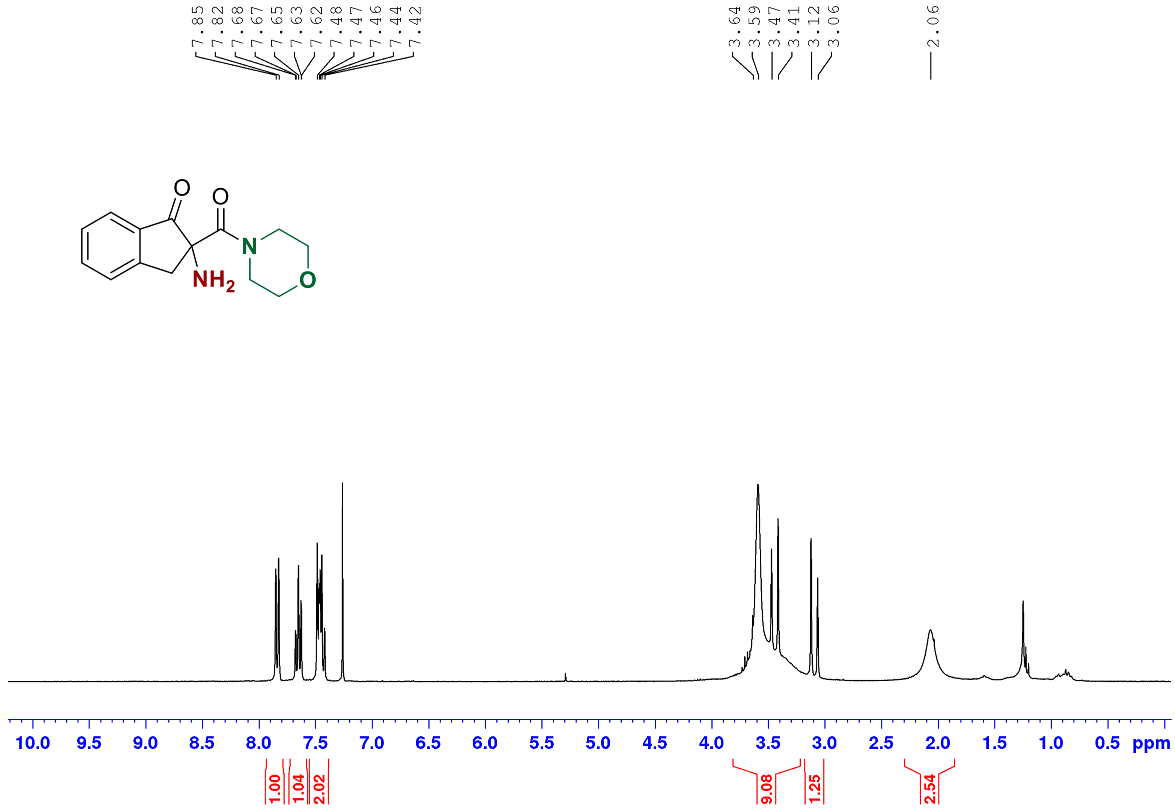

2f, ^{13}C NMR (75 MHz, $CDCl_3$, 298 K)

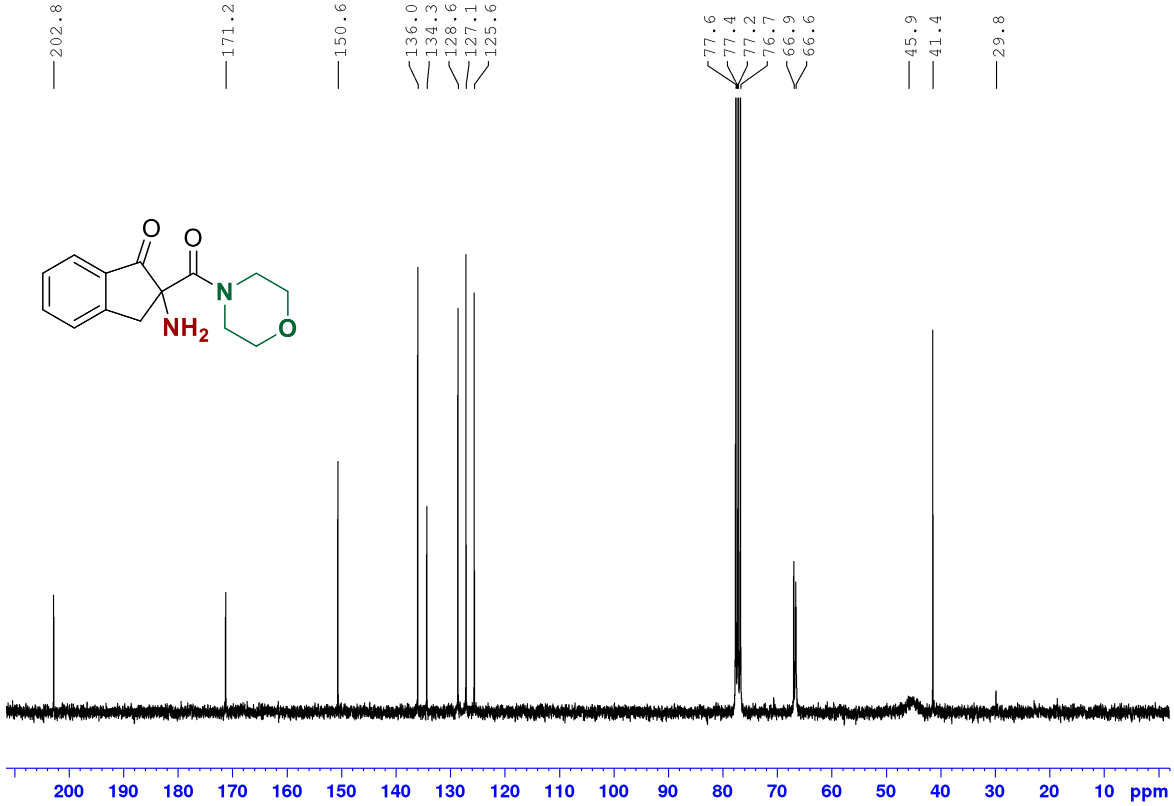

2g, ^{1}H NMR (300 MHz, $CDCl_3$, 298 K)

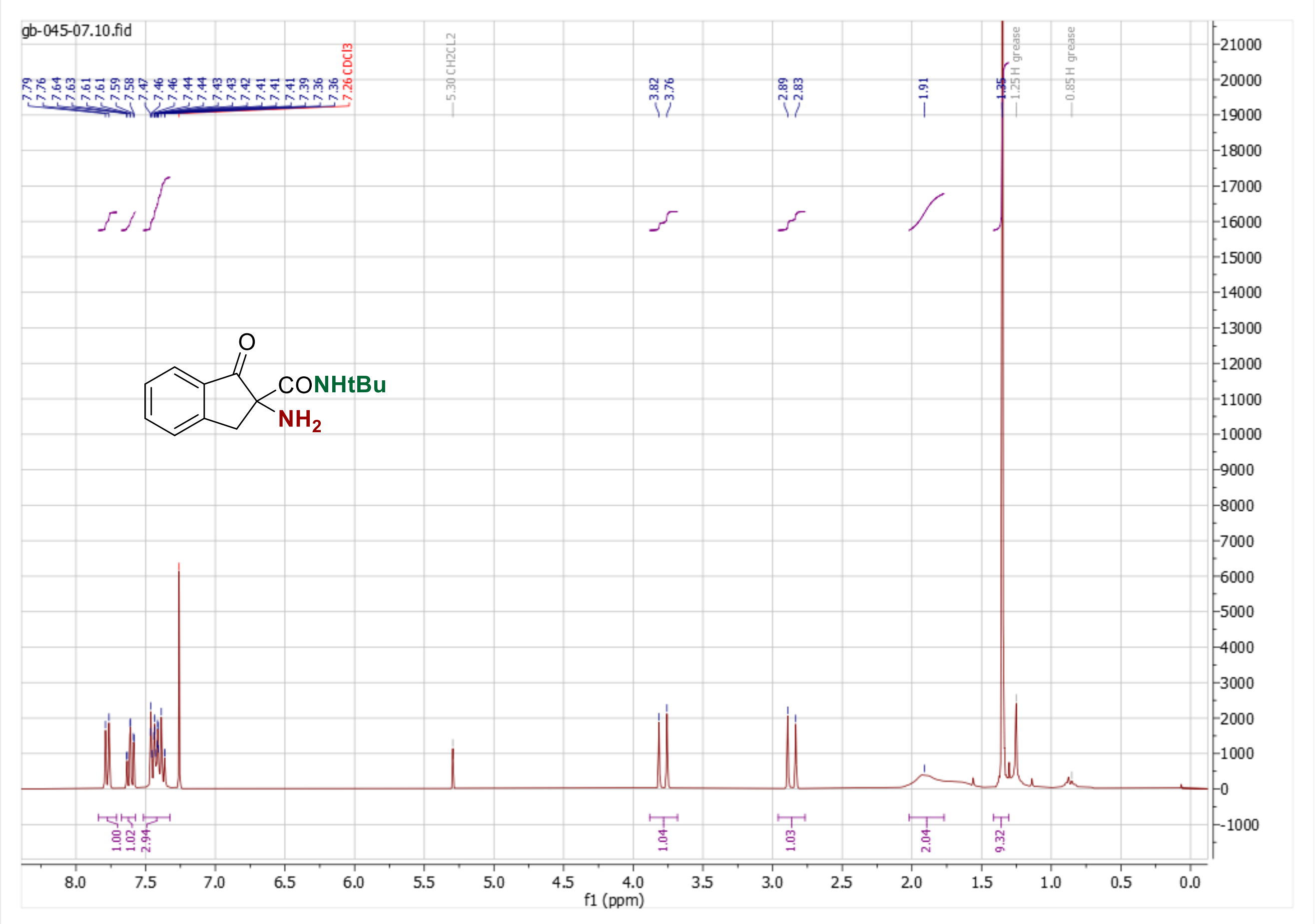

2g, ^{13}C NMR (75 MHz, $CDCl_3$, 298 K)

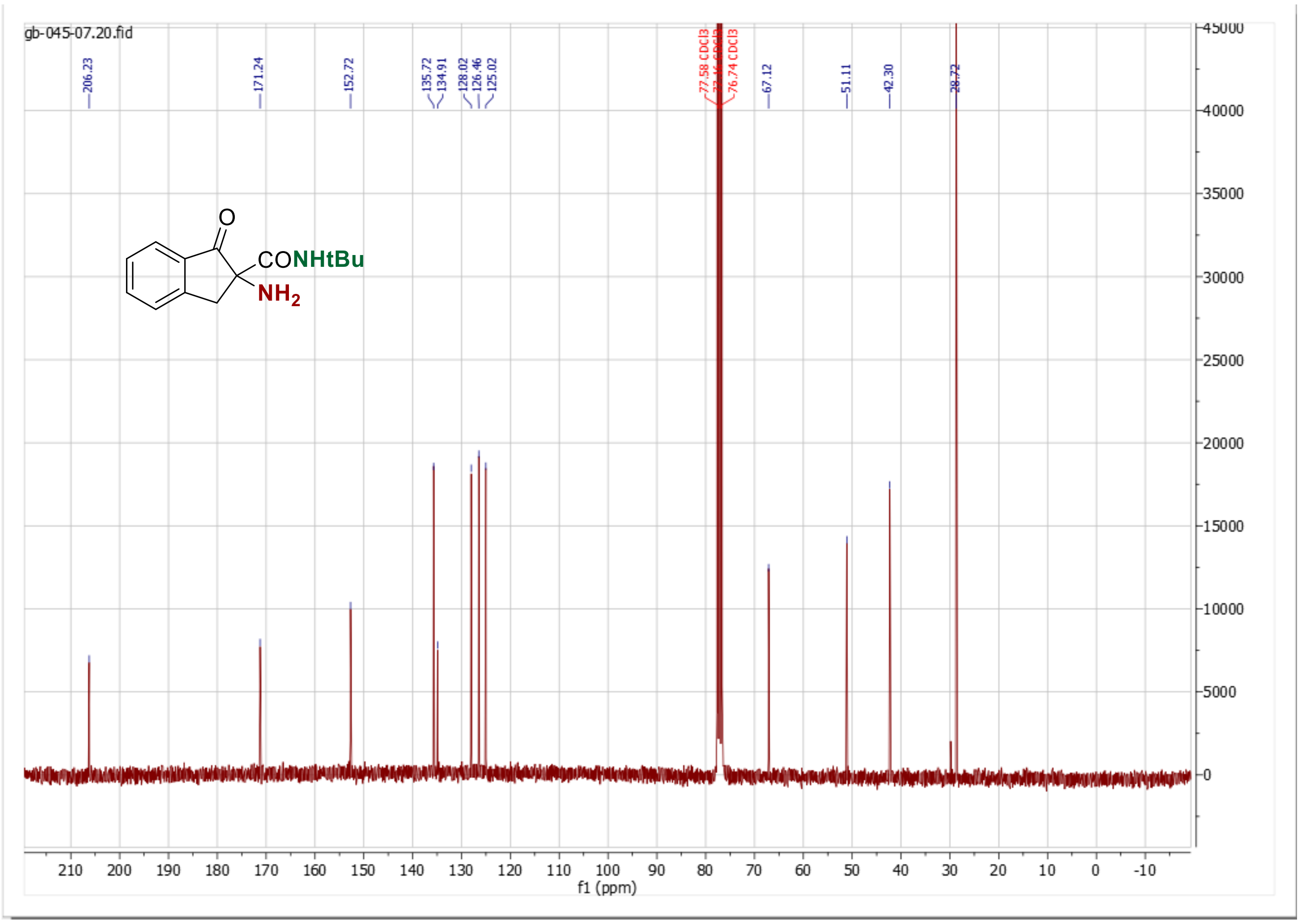

2h, ^{1}H NMR (300 MHz, $CDCl_3$, 298 K)

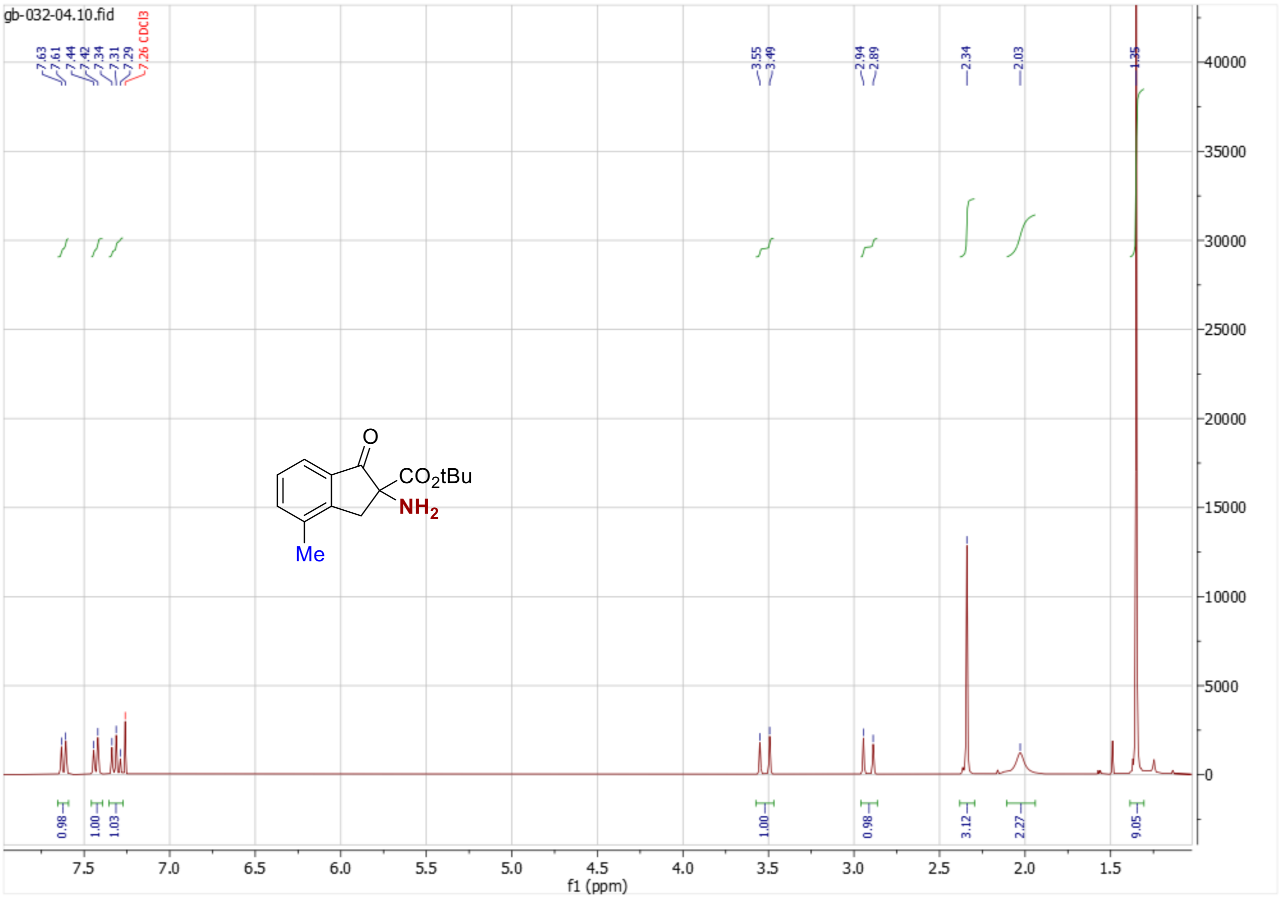

2h, ^{13}C NMR (75 MHz, $CDCl_3$, 298 K)

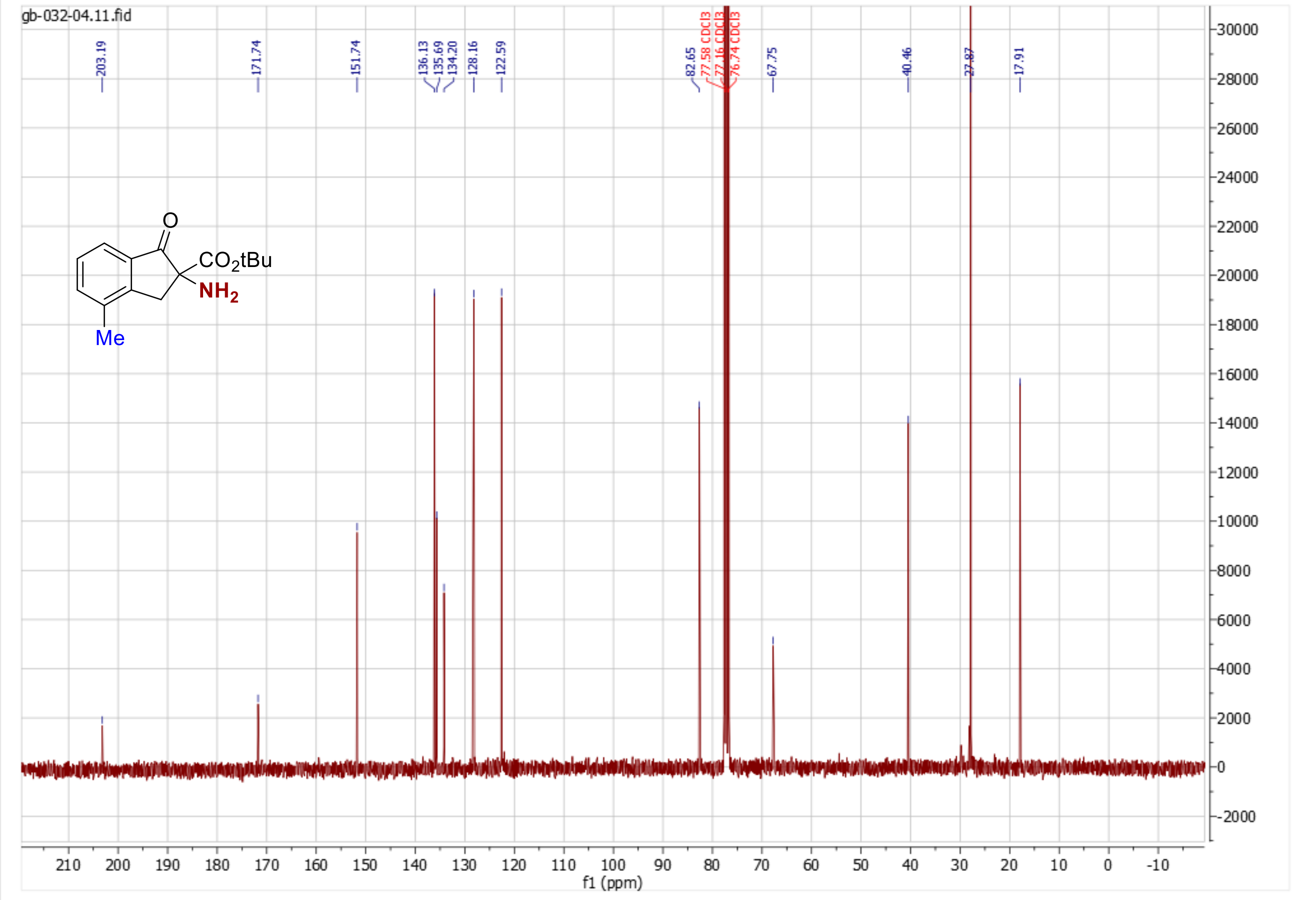

2i, ^{1}H NMR (300 MHz, $CDCl_3$, 298 K)

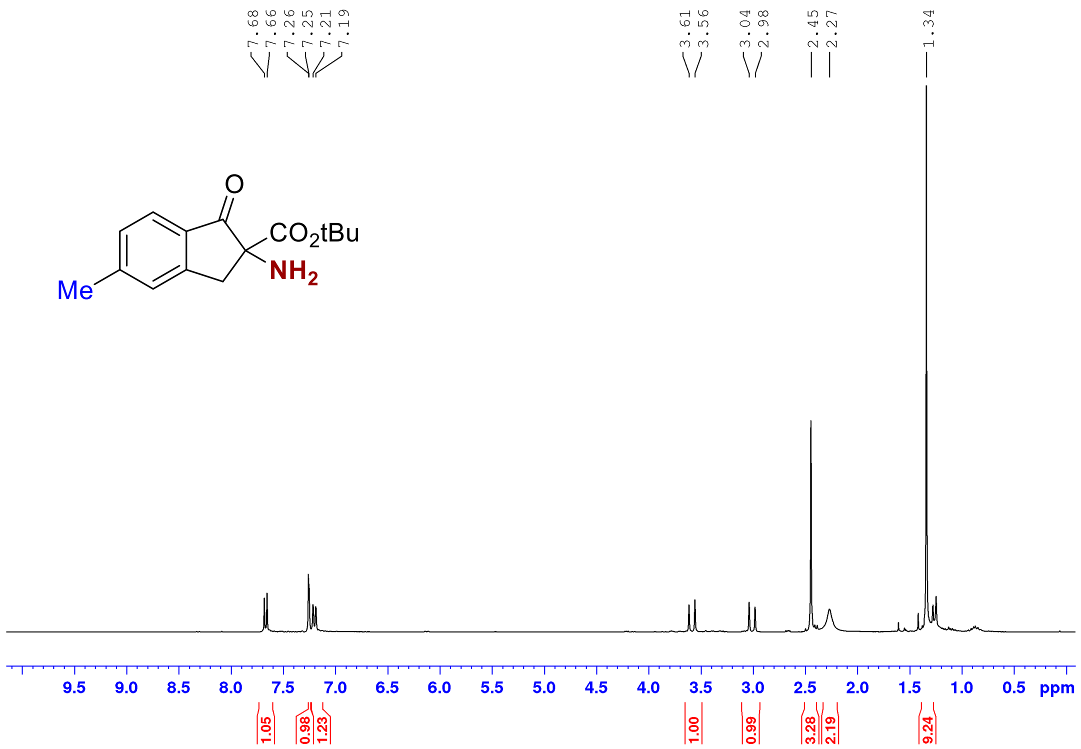

2i, ^{13}C NMR (75 MHz, $CDCl_3$, 298 K)

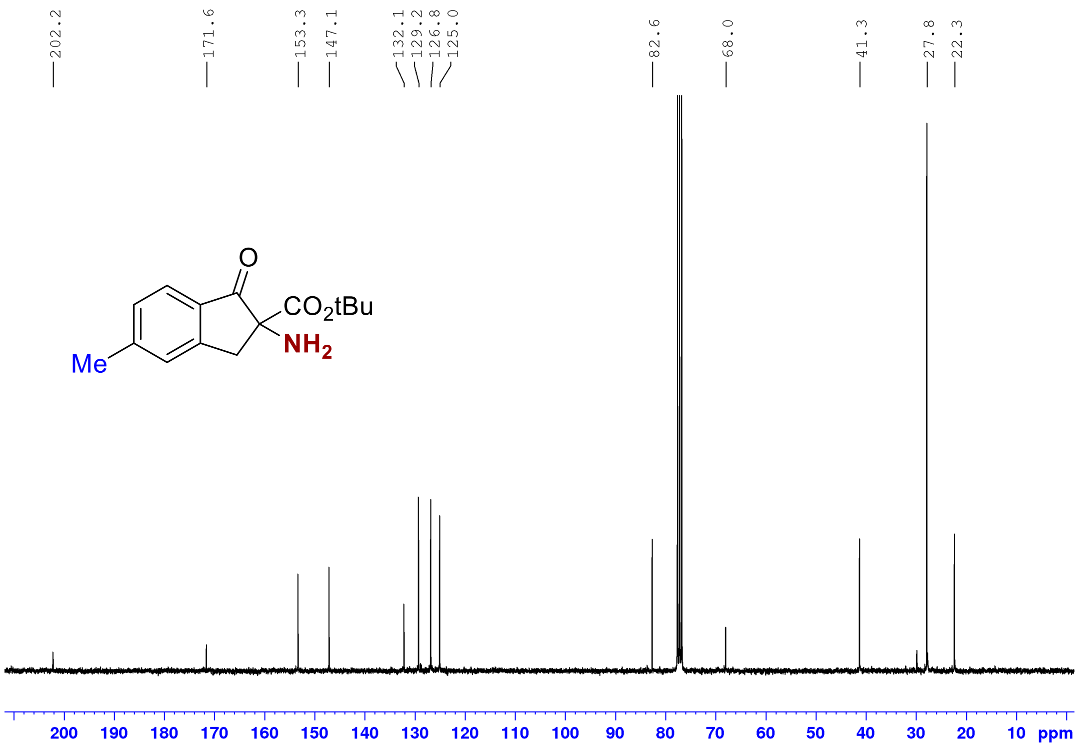

2j, ^{1}H NMR (300 MHz, $CDCl_3$, 298 K)

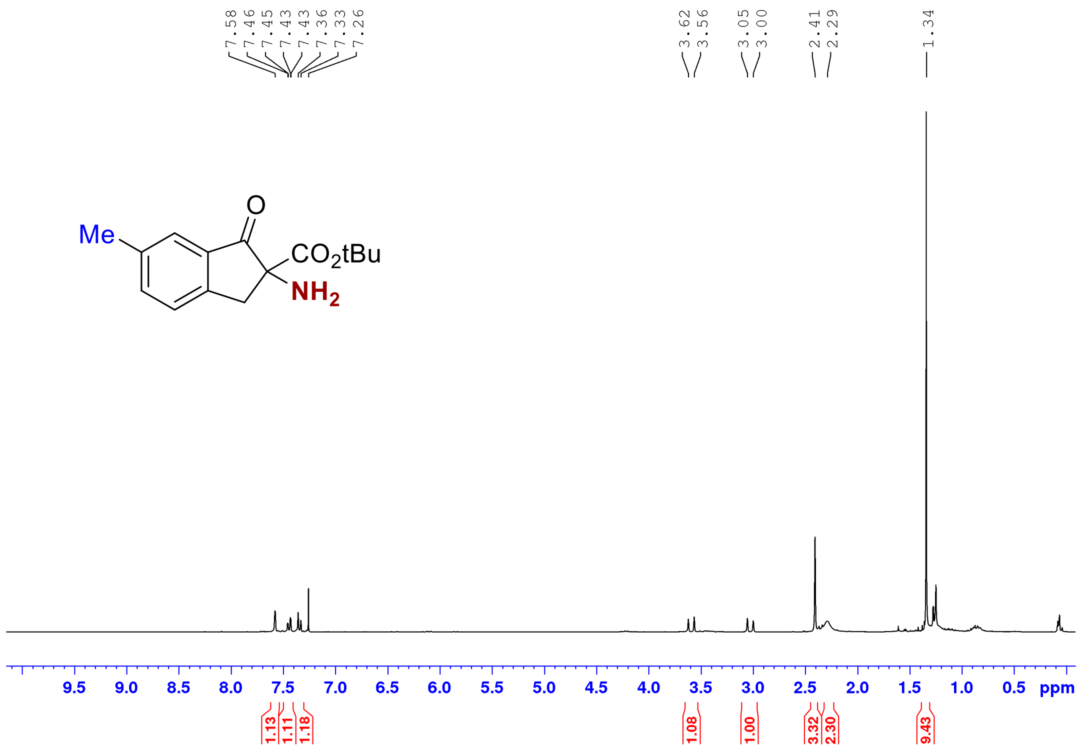

2j, ^{13}C NMR (75 MHz, $CDCl_3$, 298 K)

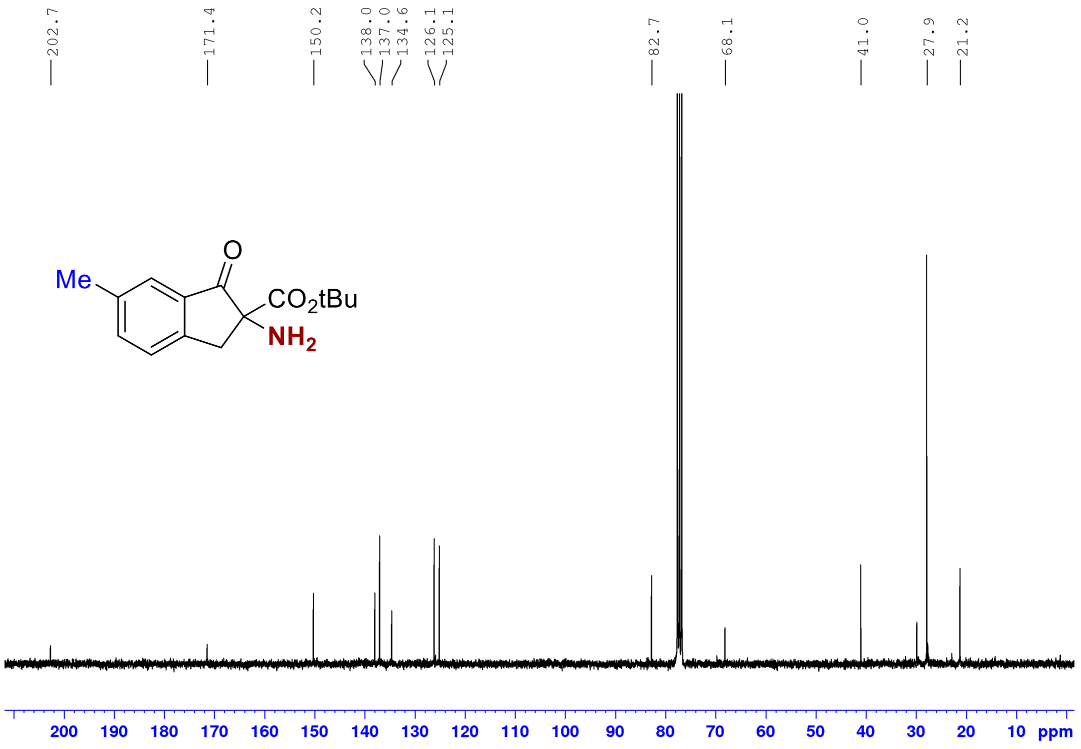

2k, ^{1}H NMR (300 MHz, CDCl$_3$, 298 K)

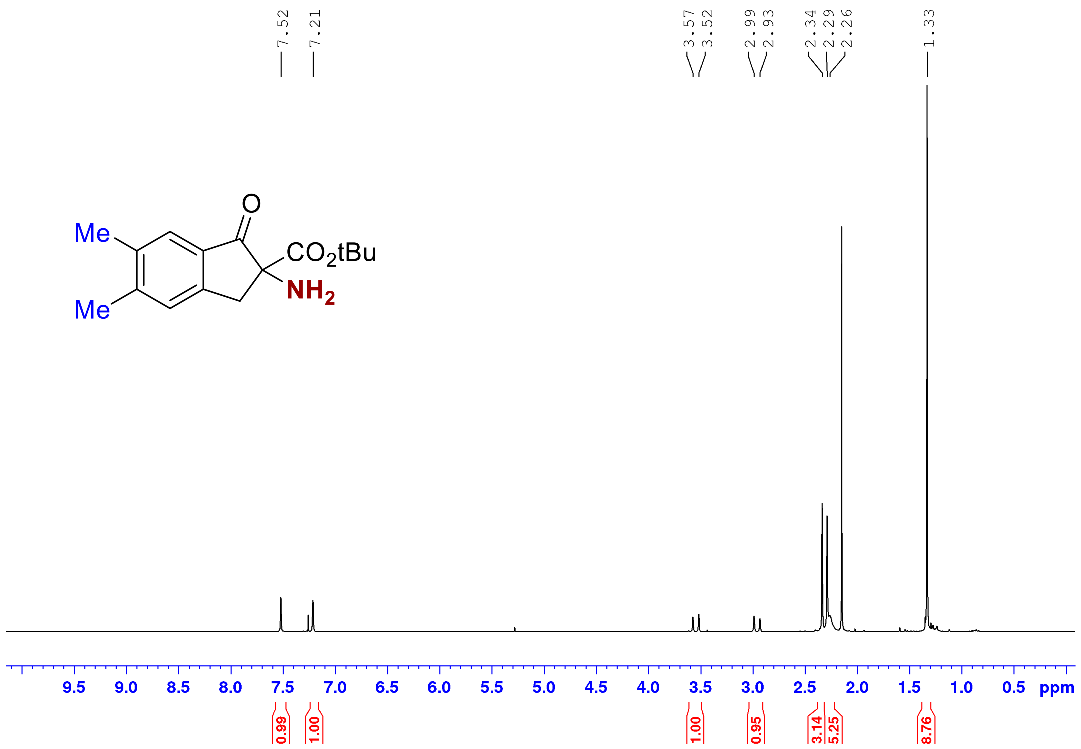

2k, ^{13}C NMR (75 MHz, CDCl$_3$, 298 K)

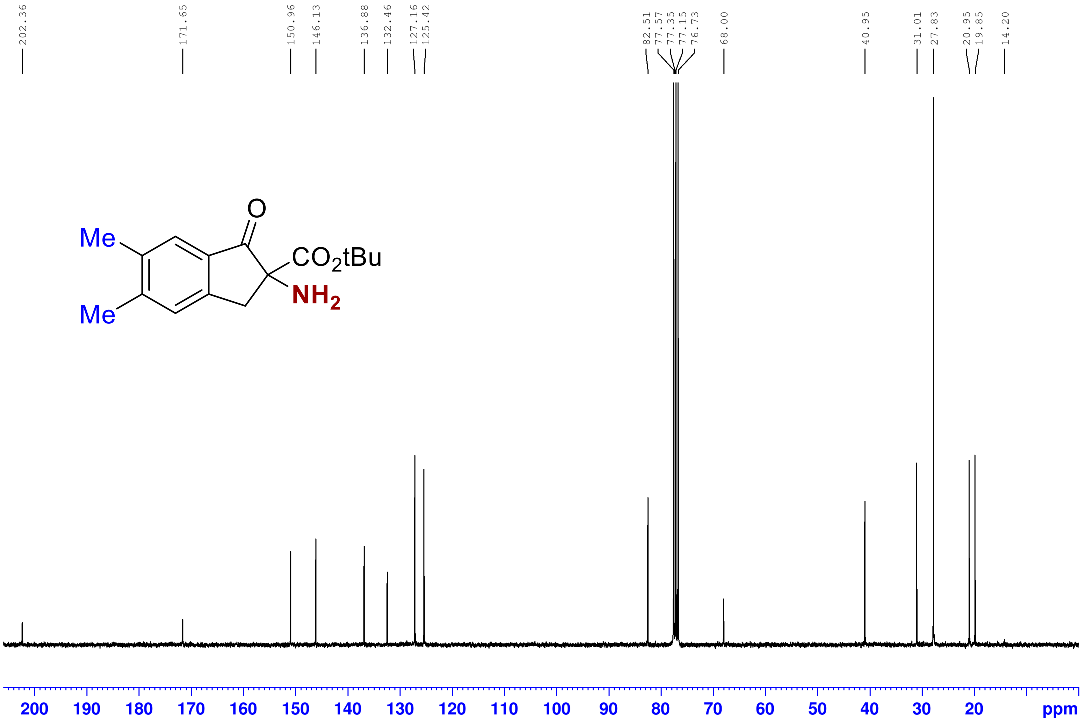

2l, ^{1}H NMR (300 MHz, $CDCl_3$, 298 K)

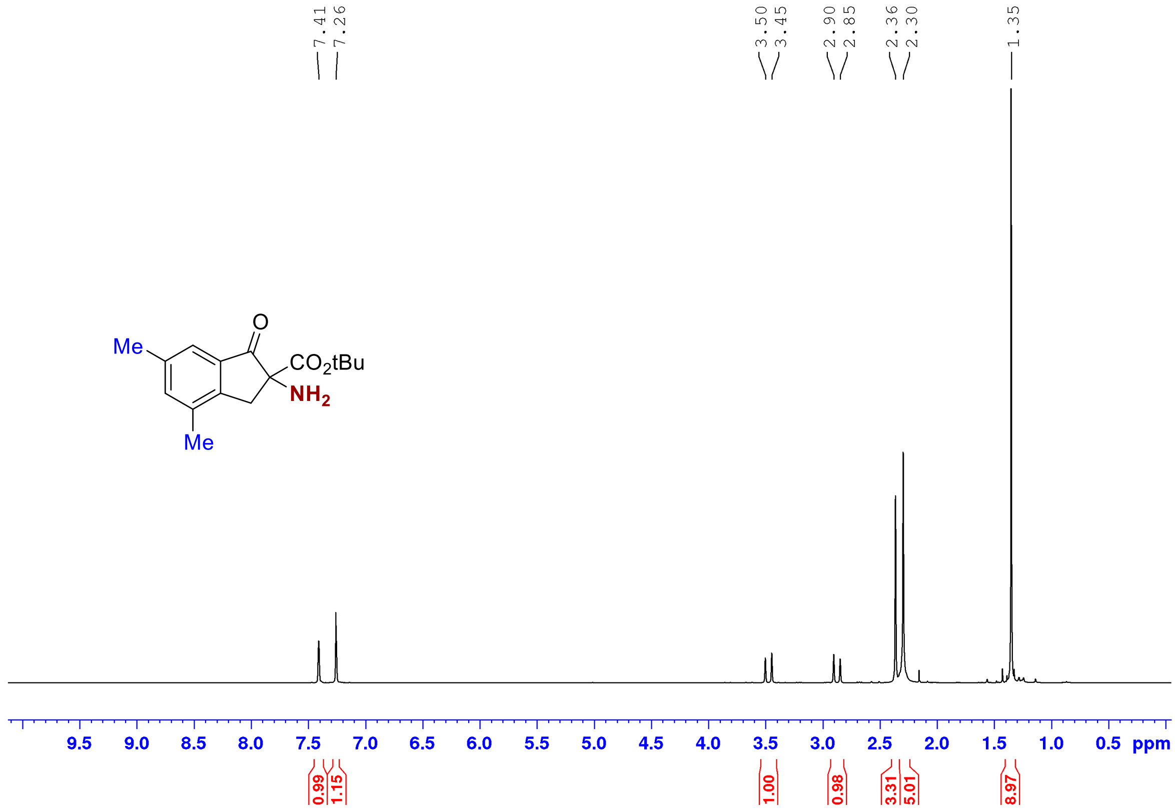

2l, ^{13}C NMR (75 MHz, $CDCl_3$, 298 K)

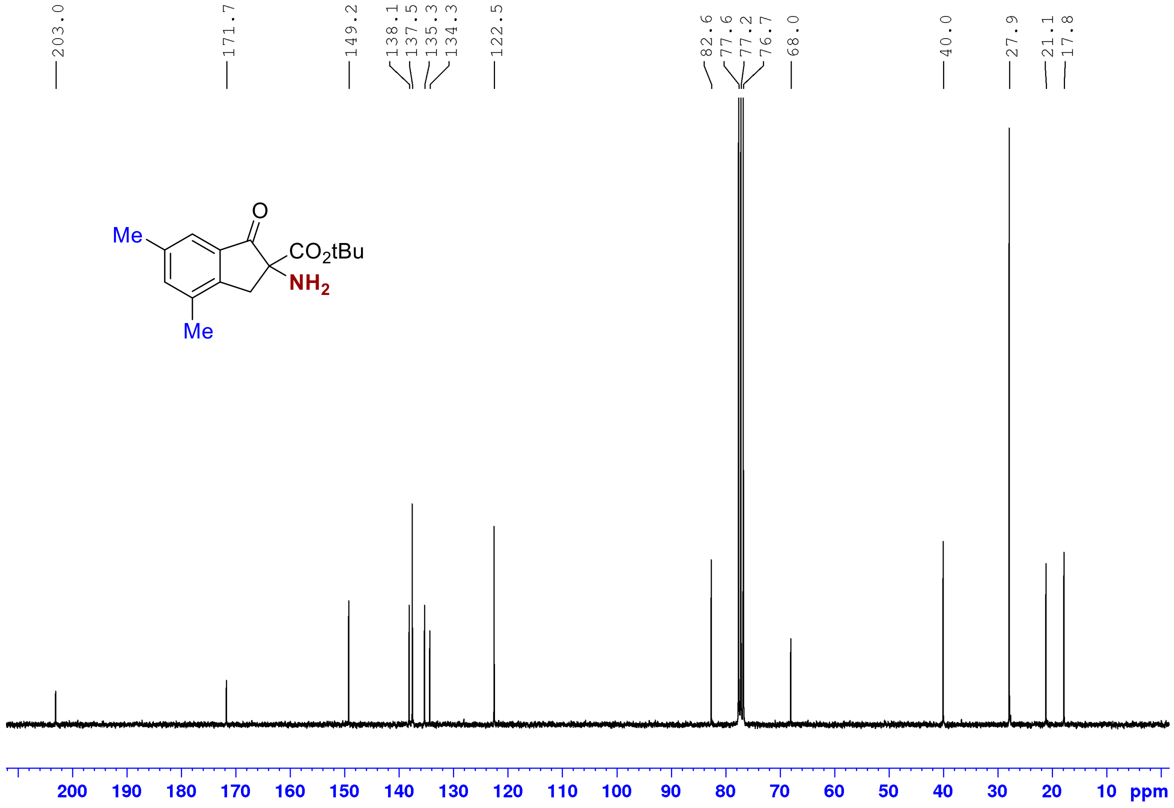

2m, ^{1}H NMR (300 MHz, $CDCl_3$, 298 K)

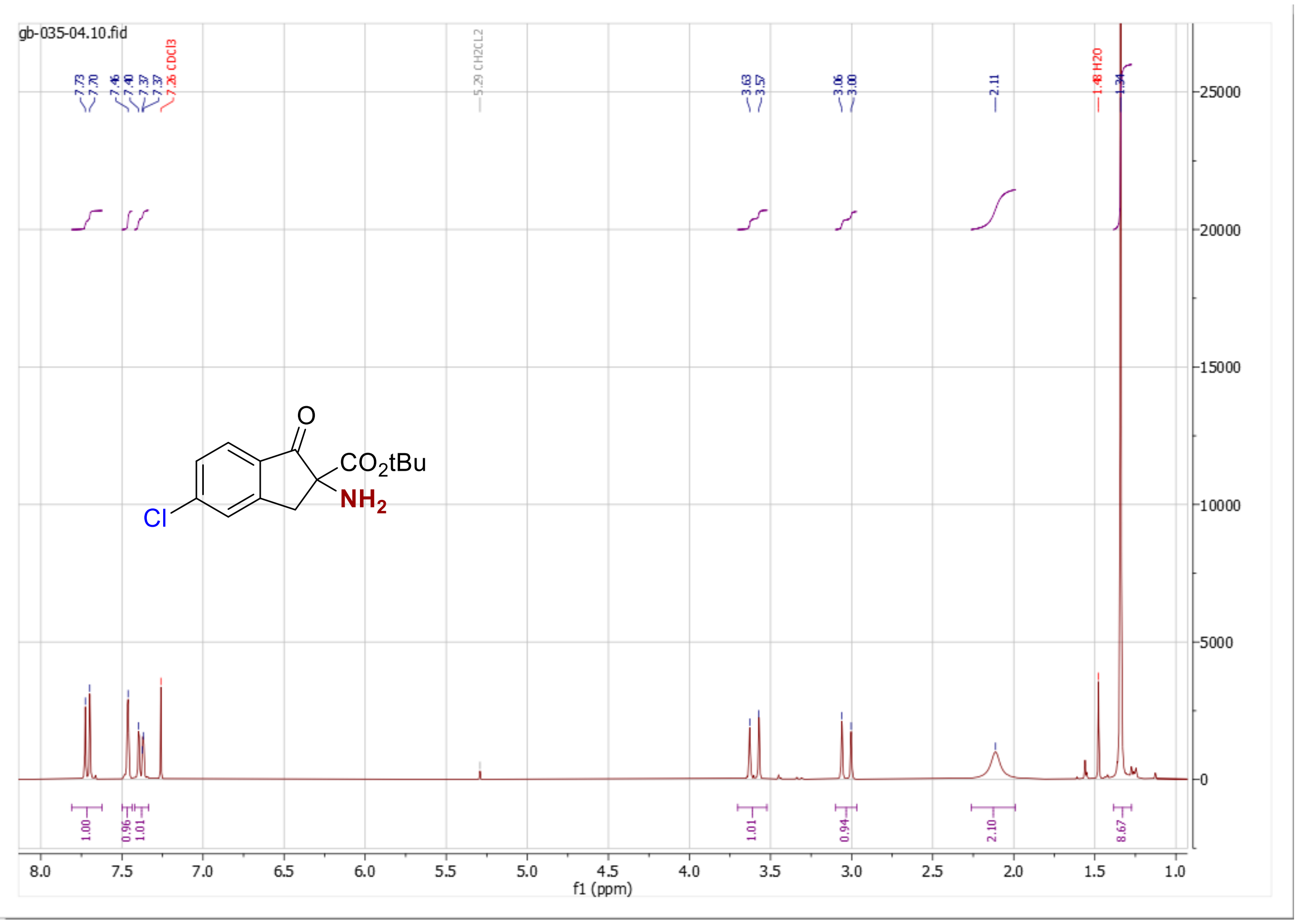

2m, ^{13}C NMR (75 MHz, $CDCl_3$, 298 K)

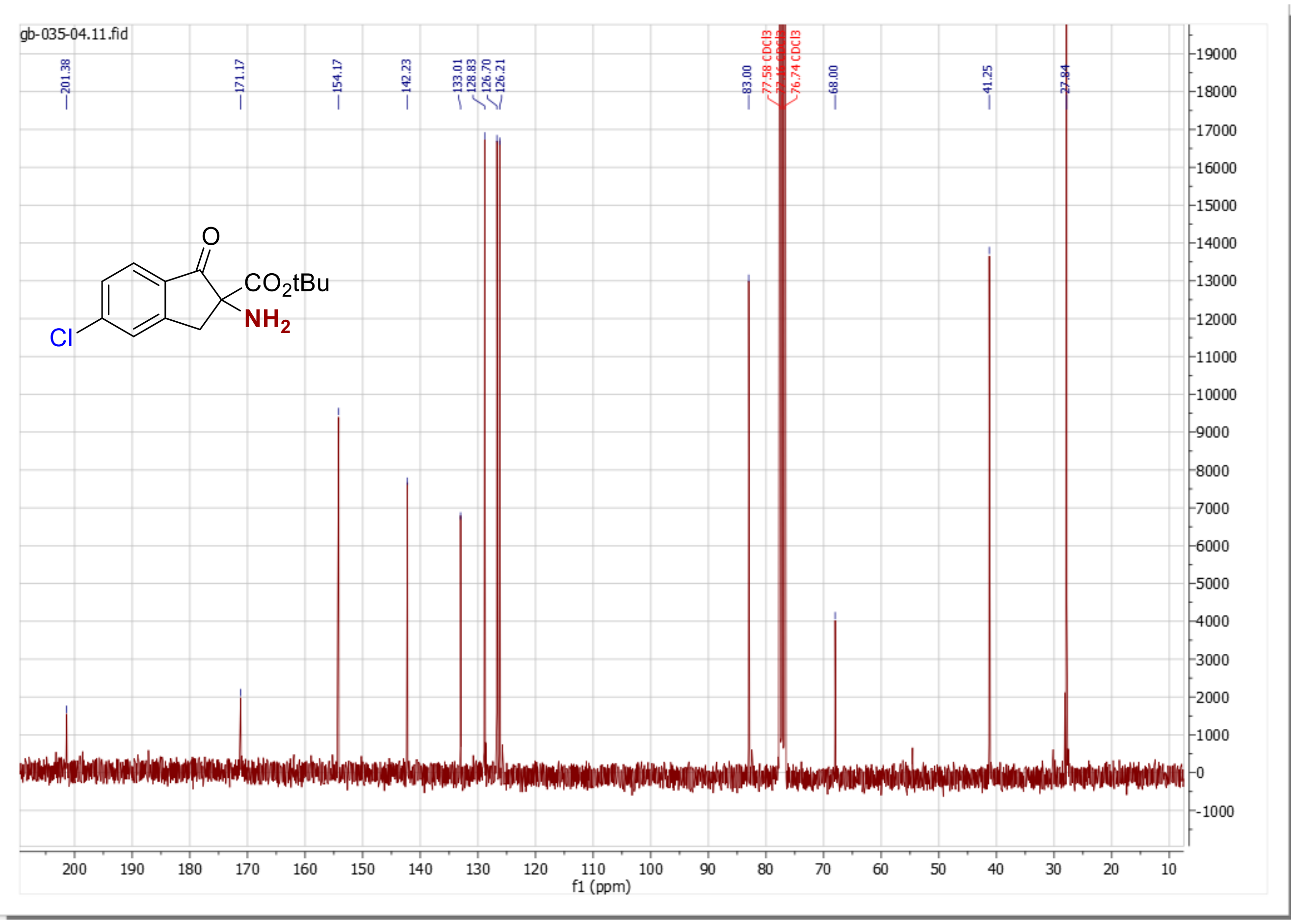

2n, ^{1}H NMR (300 MHz, $CDCl_3$, 298 K)

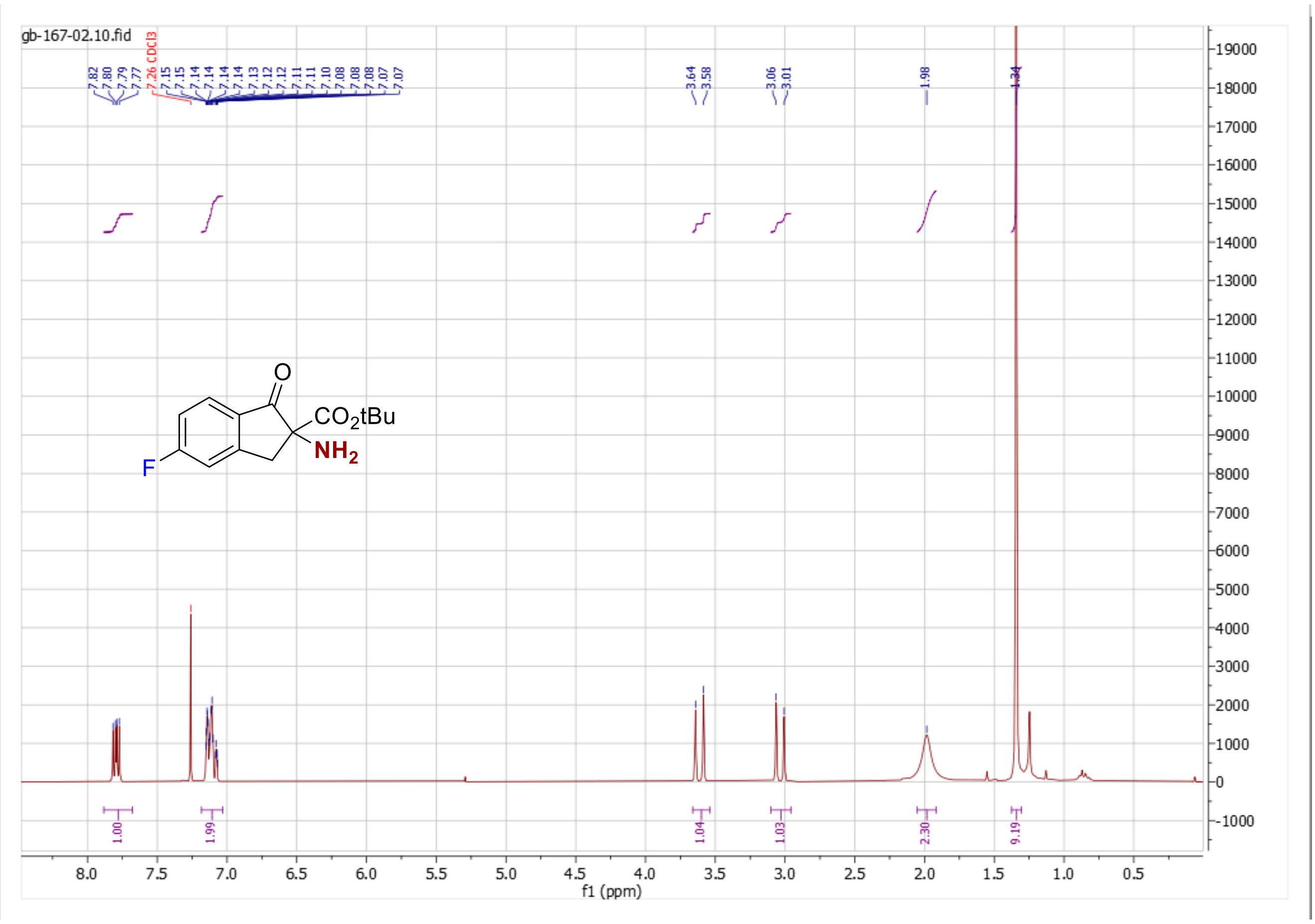

2n, ^{13}C NMR (75 MHz, $CDCl_3$, 298 K)

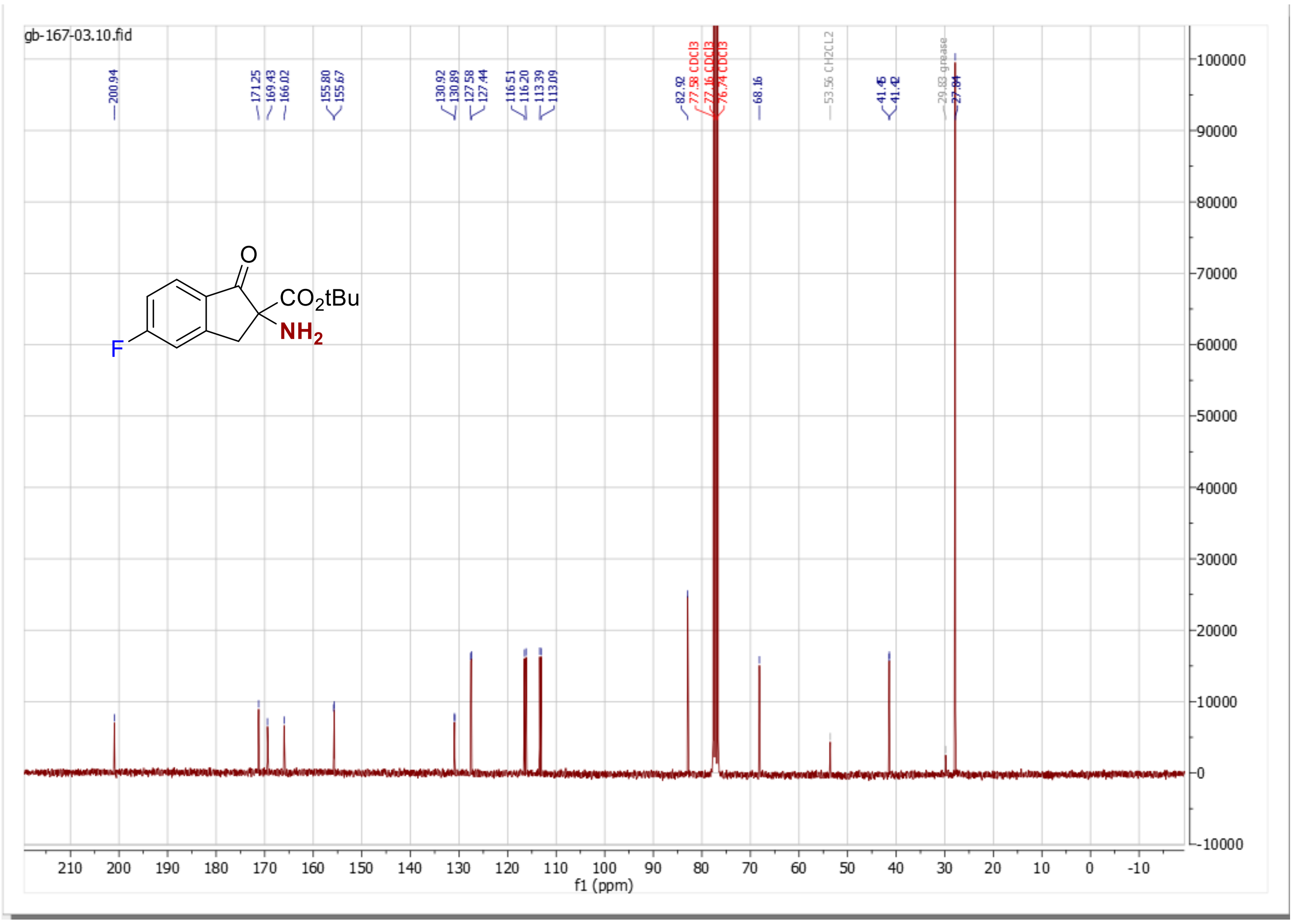

2n, ^{19}F NMR (282 MHz, $CDCl_3$, 298 K)

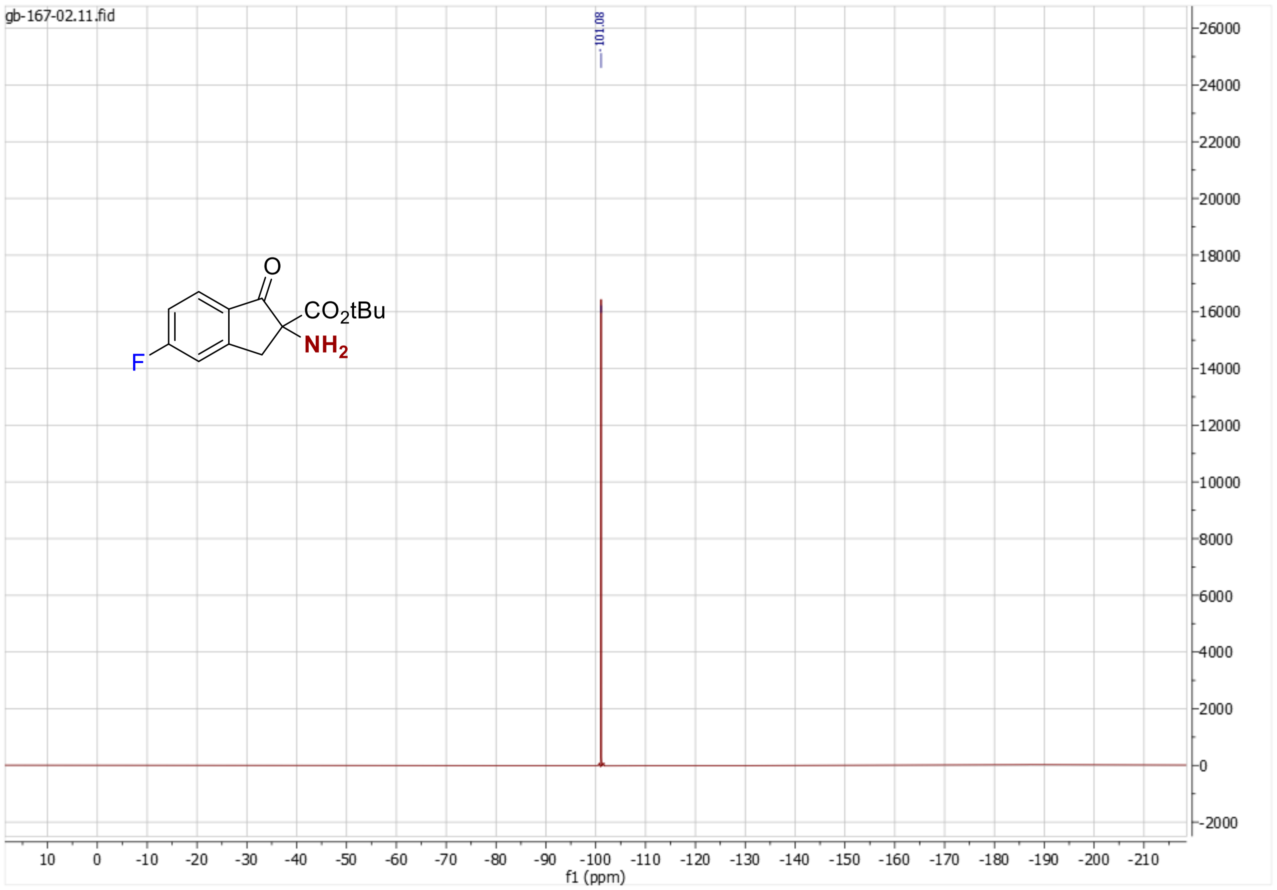

2o, ^{1}H NMR (300 MHz, $CDCl_3$, 298 K)

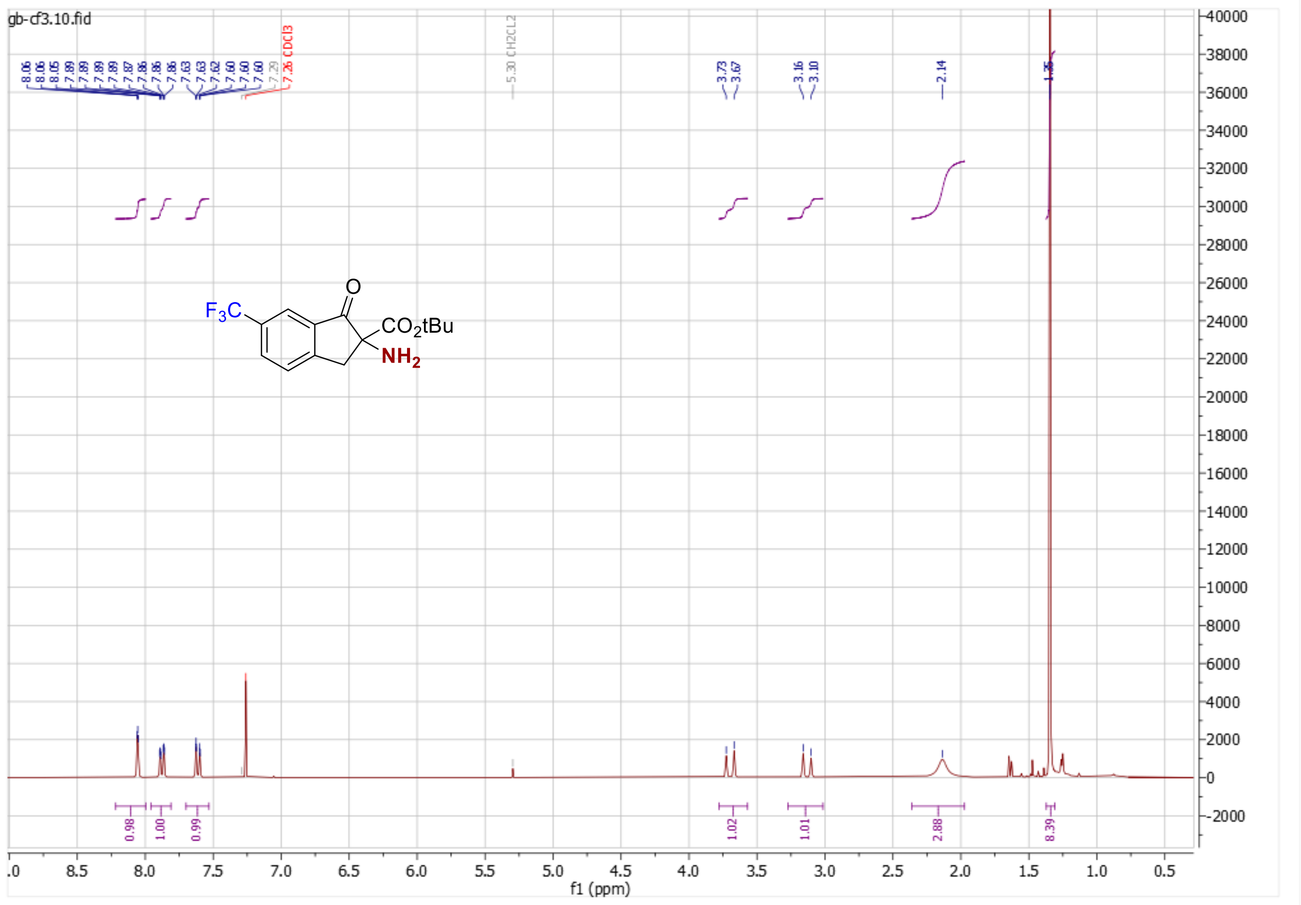

2o, ^{13}C NMR (75 MHz, $CDCl_3$, 298 K)

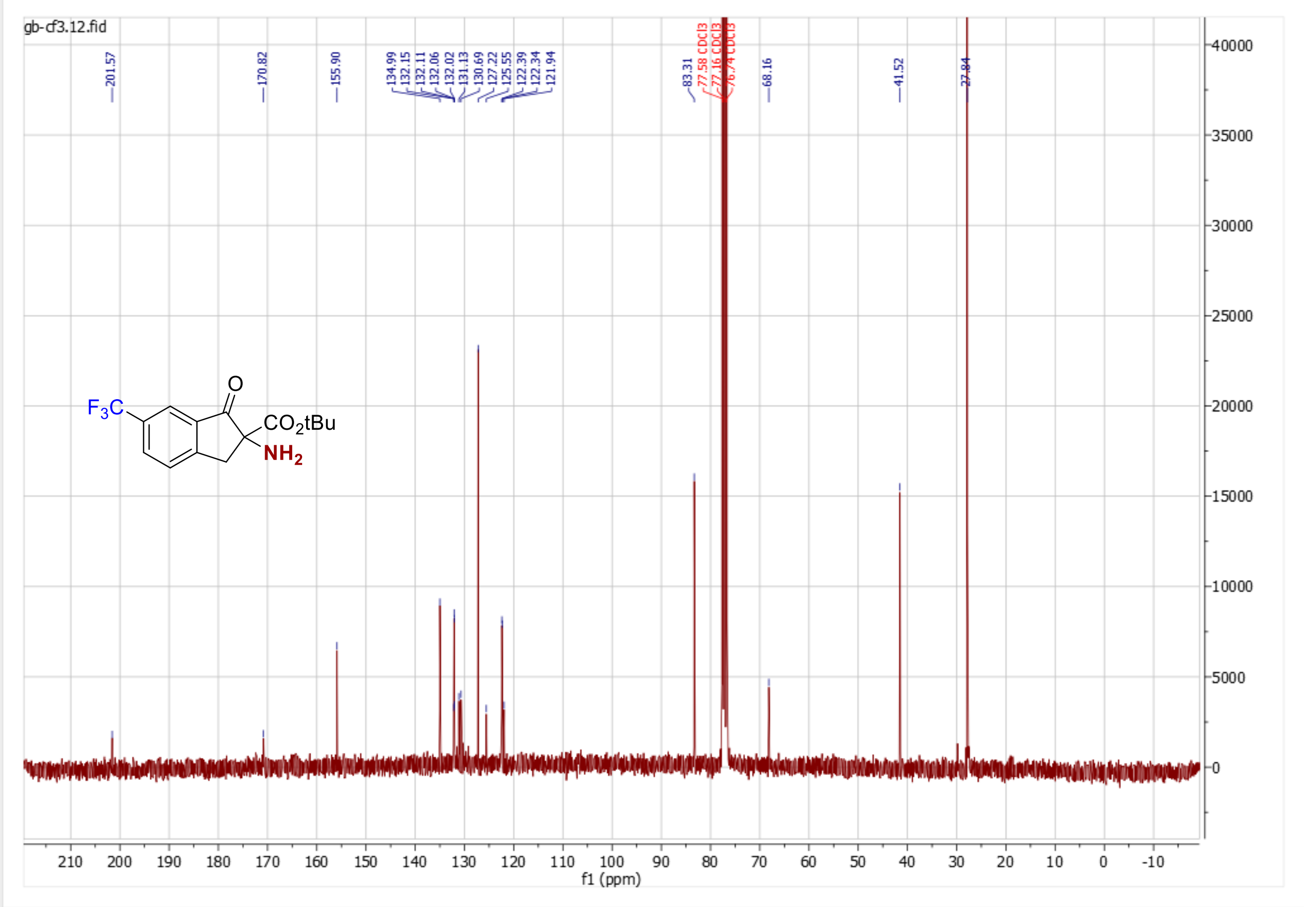

2o, ^{19}F NMR (282 MHz, $CDCl_3$, 298 K)

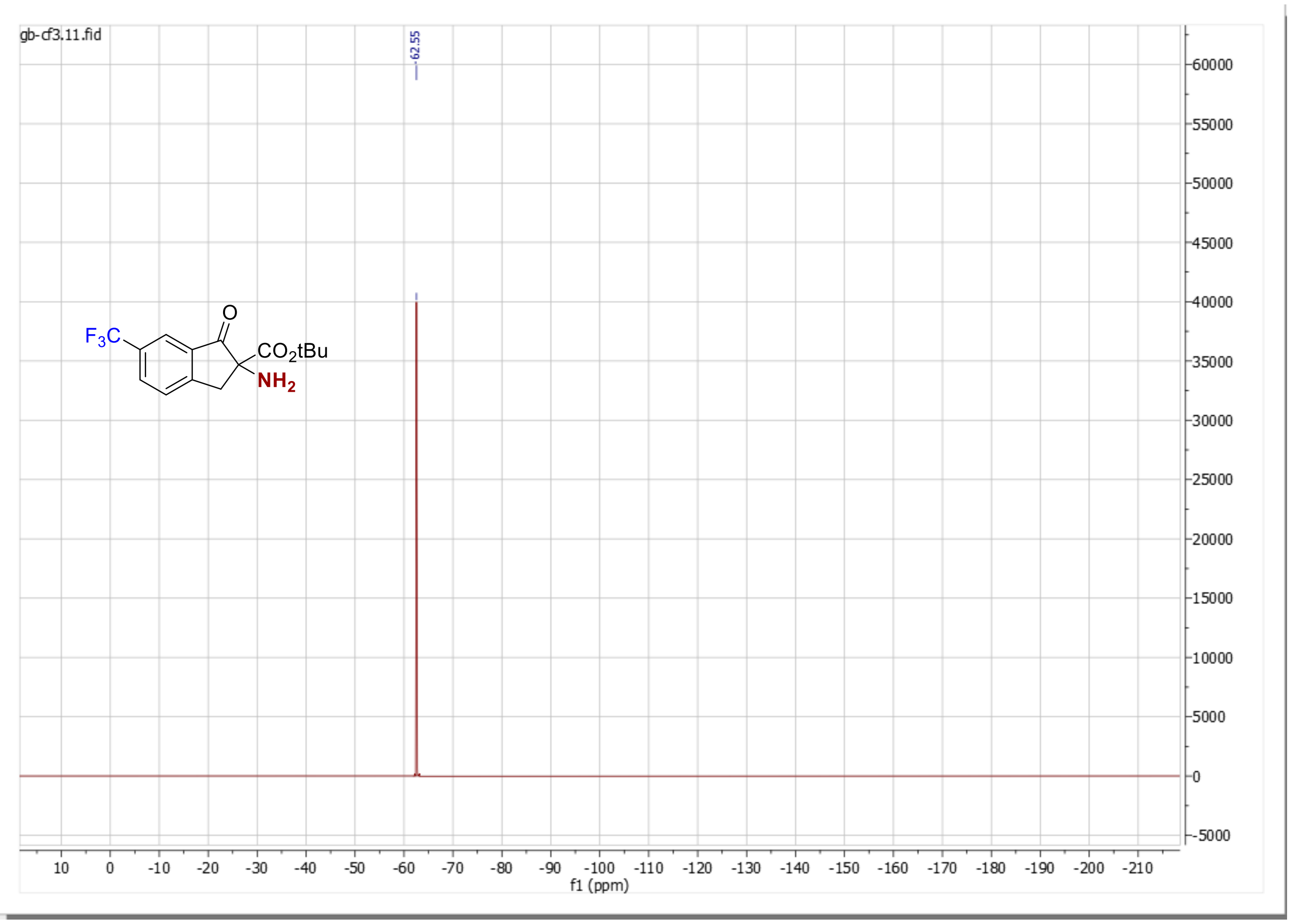

2p, ^{1}H NMR (300 MHz, $CDCl_3$, 298 K)

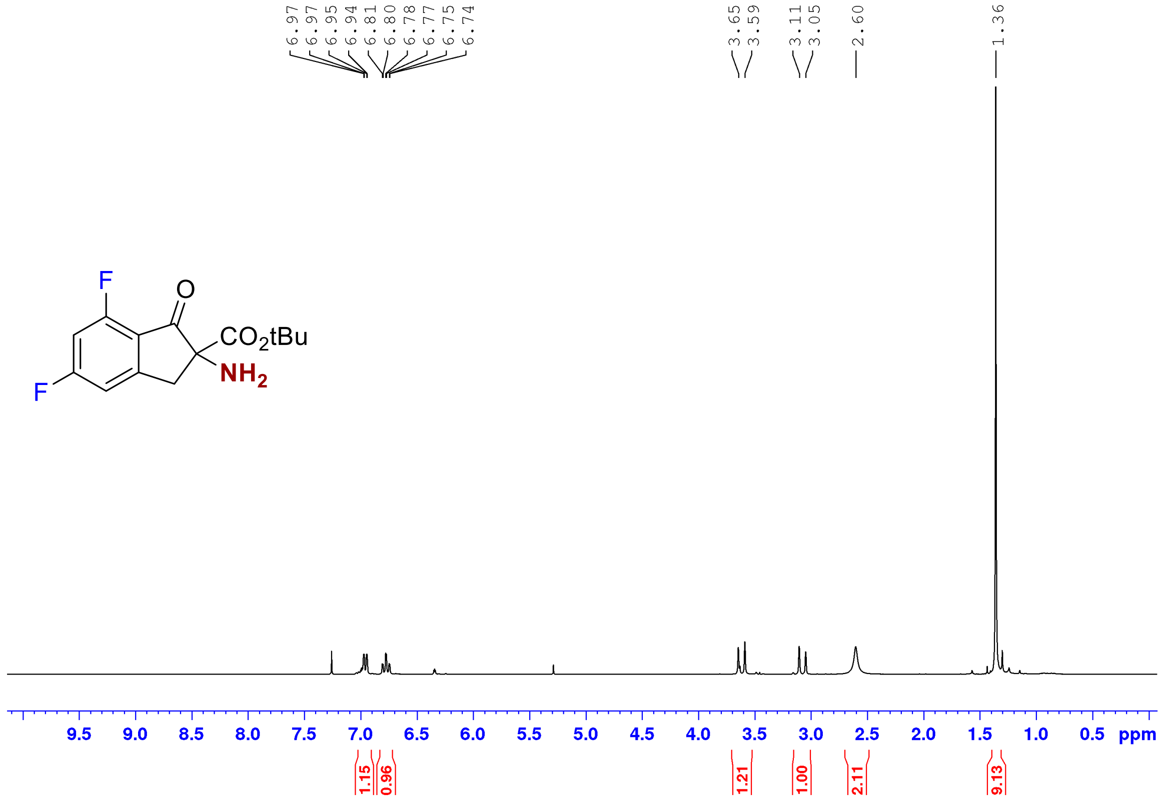

2p, ^{13}C NMR (75 MHz, $CDCl_3$, 298 K)

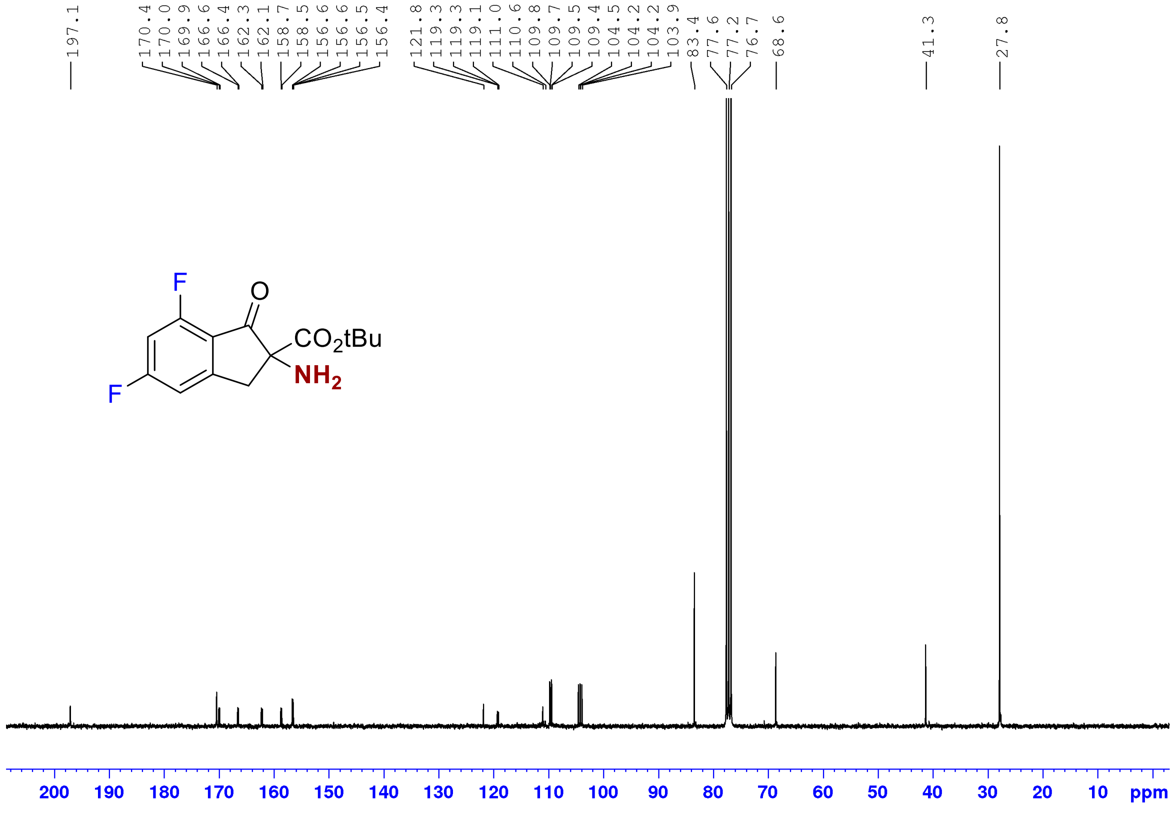

2p, ^{19}F NMR (282 MHz, $CDCl_3$, 298 K)

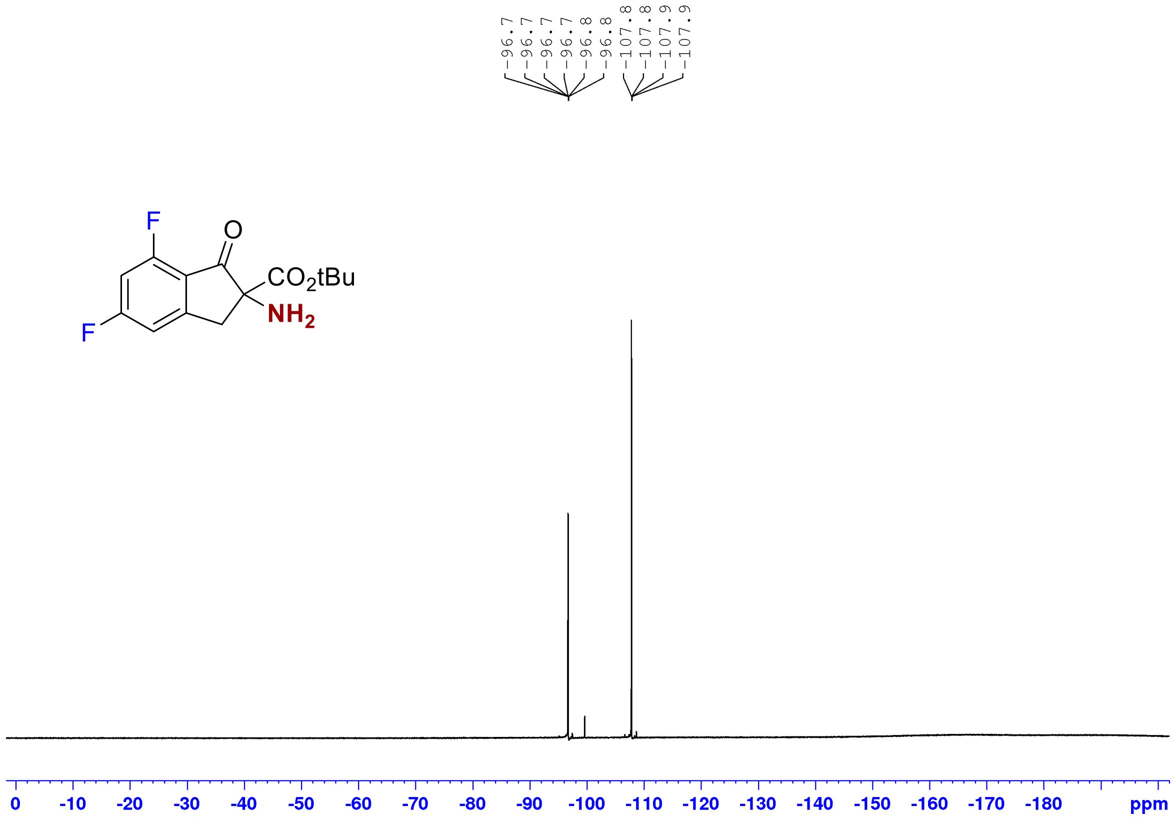

2q, ^{1}H NMR (300 MHz, $CDCl_3$, 298 K)

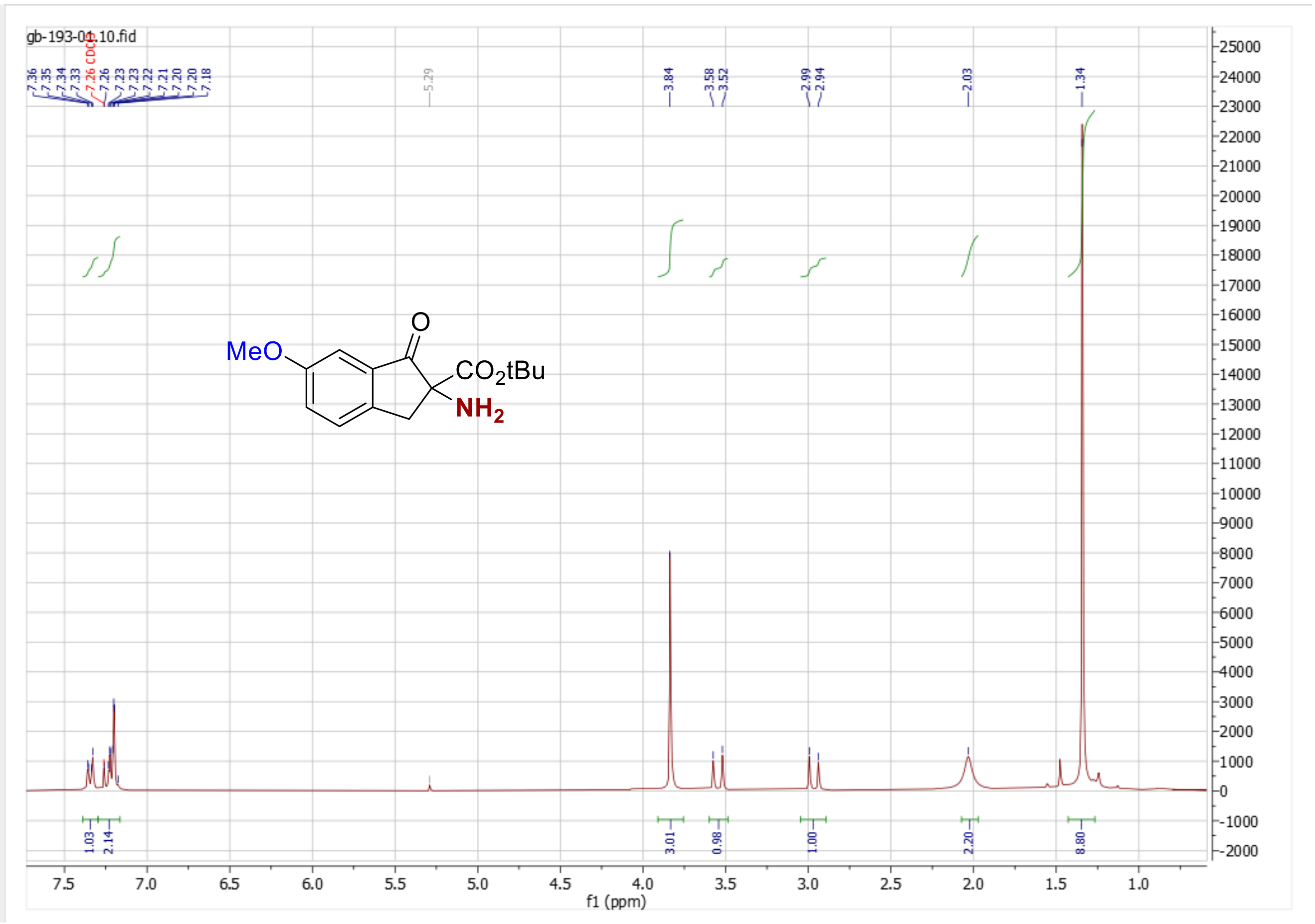

2q, ^{13}C NMR (75 MHz, $CDCl_3$, 298 K)

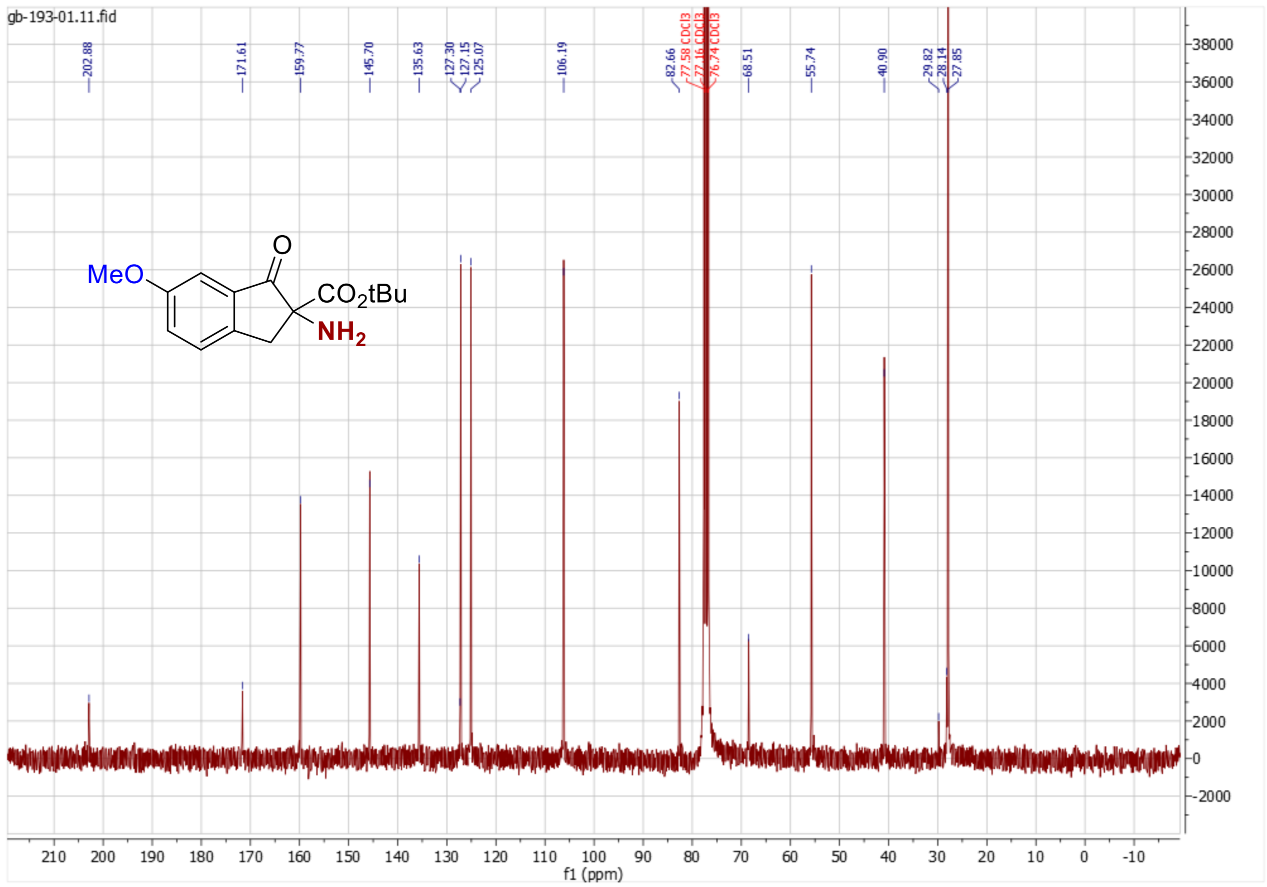

2r, ^{1}H NMR (300 MHz, $CDCl_3$, 298 K)

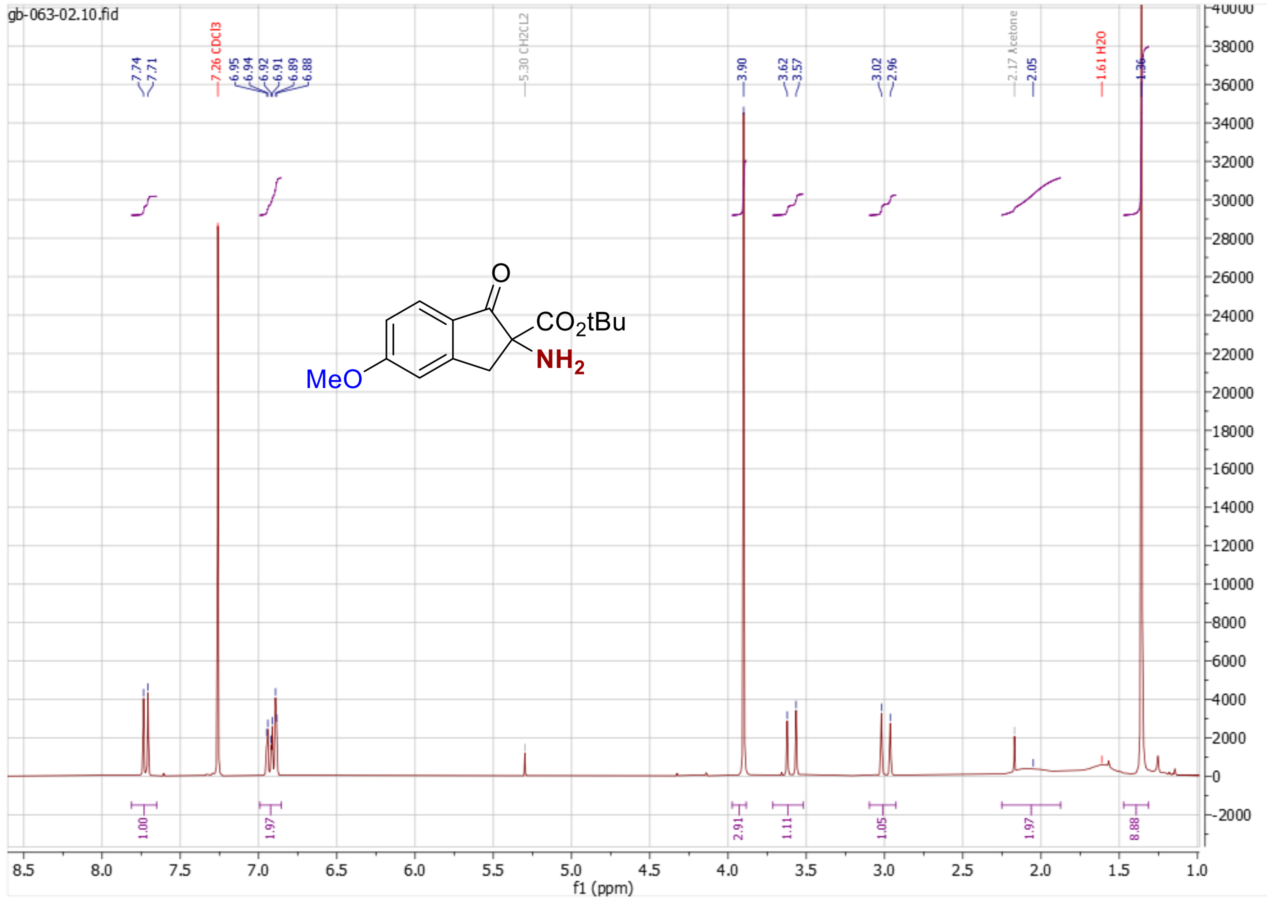

2r, ^{13}C NMR (75 MHz, $CDCl_3$, 298 K)

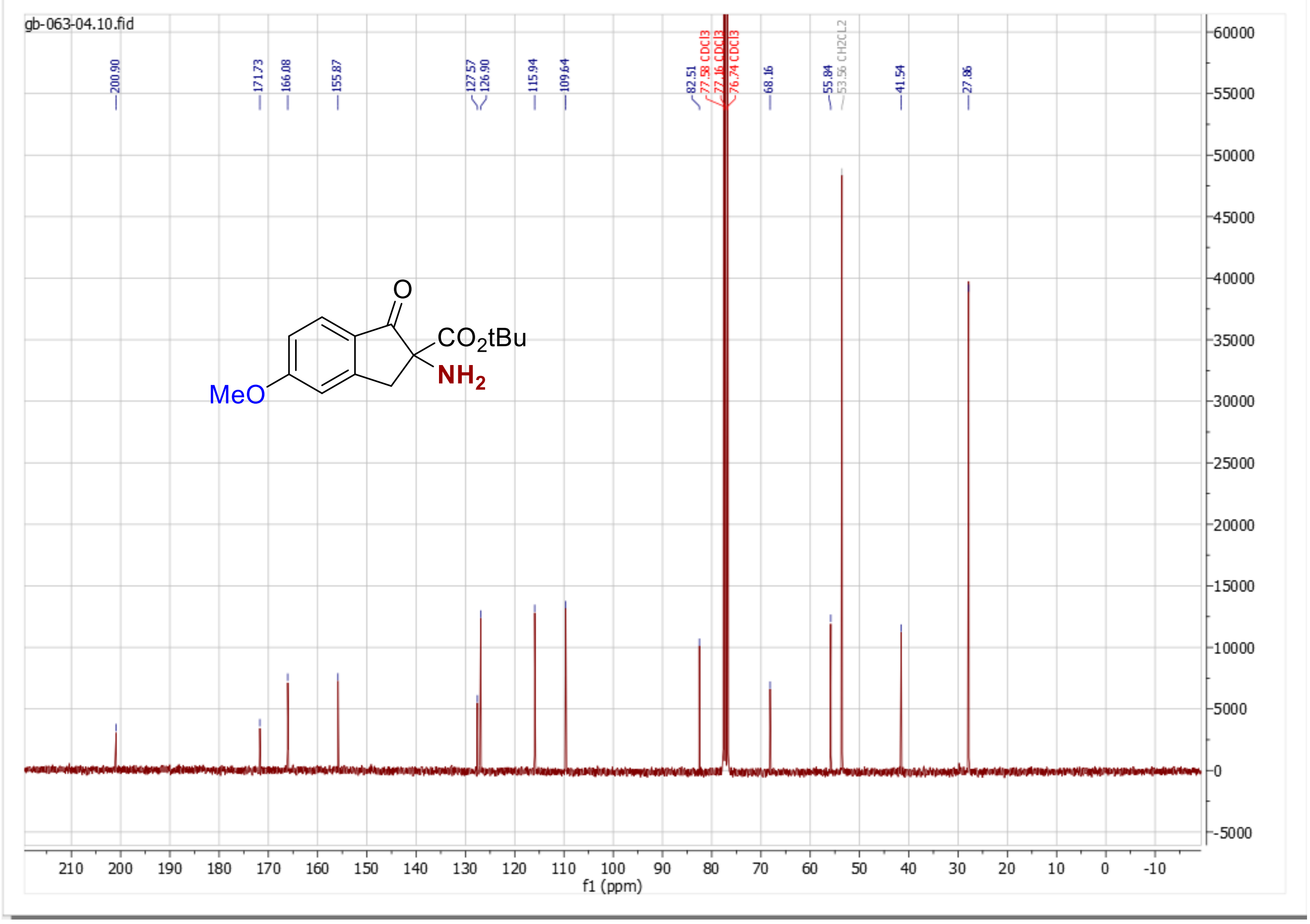

2s, ^{1}H NMR (300 MHz, $CDCl_3$, 298 K)

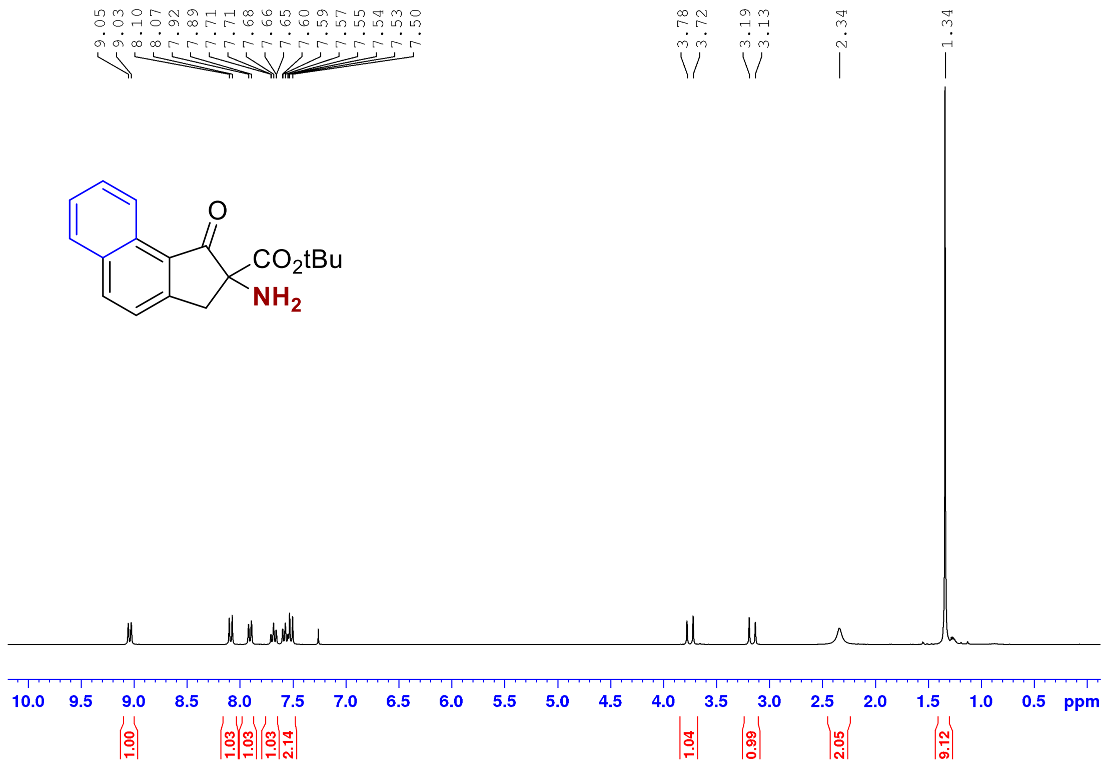

2s, ^{13}C NMR (75 MHz, $CDCl_3$, 298 K)

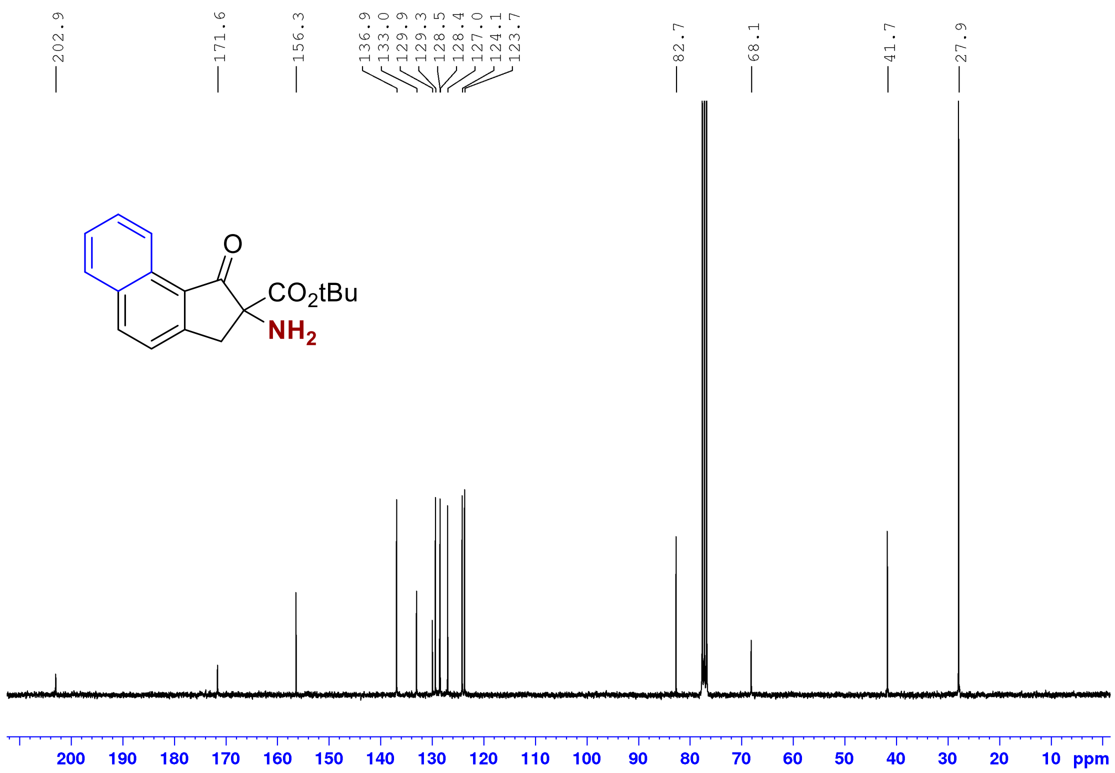

4a, ^{1}H NMR (300 MHz, $CDCl_3$, 298 K)

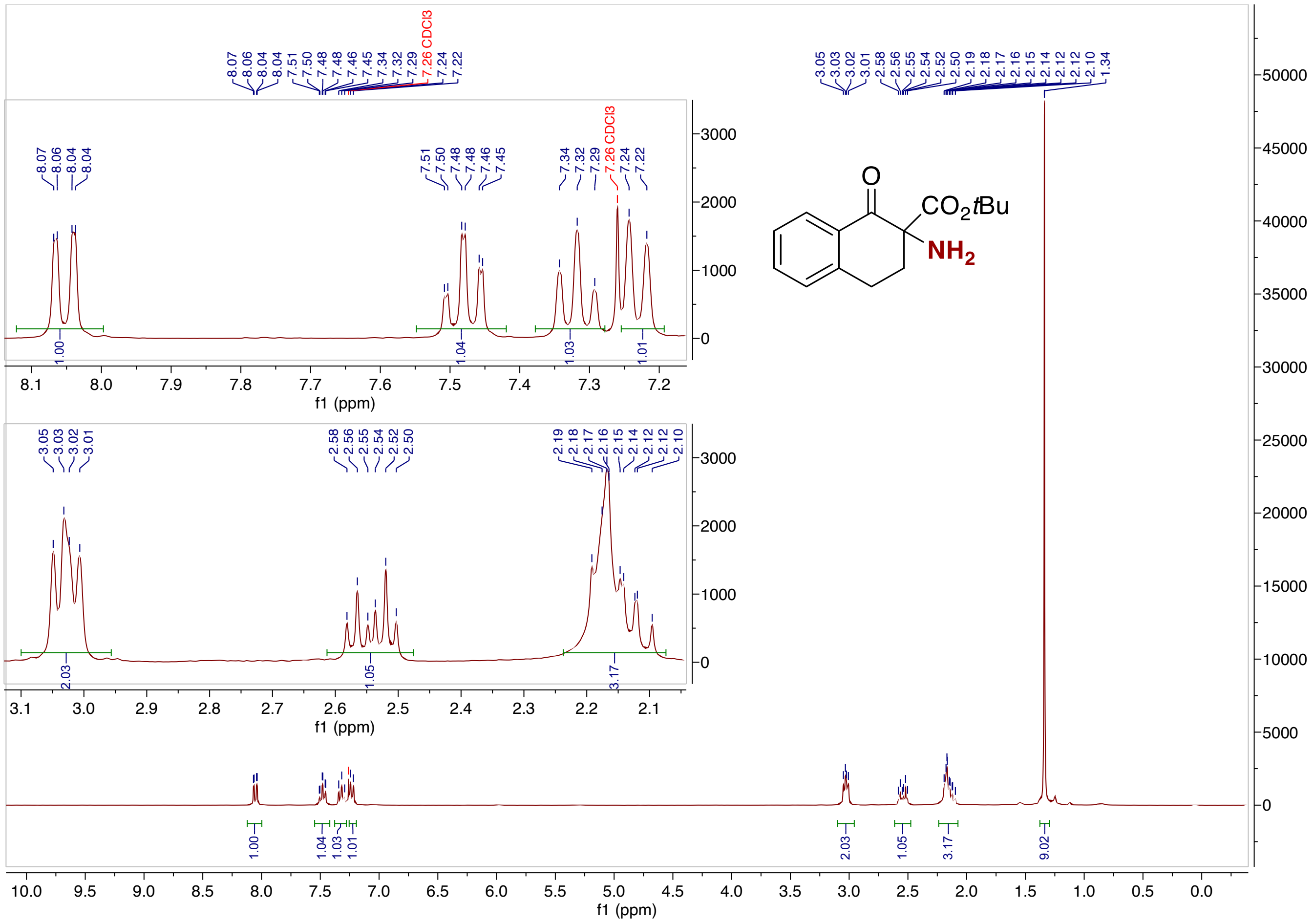

4a, ^{13}C NMR (75 MHz, CDCl$_3$, 298 K)

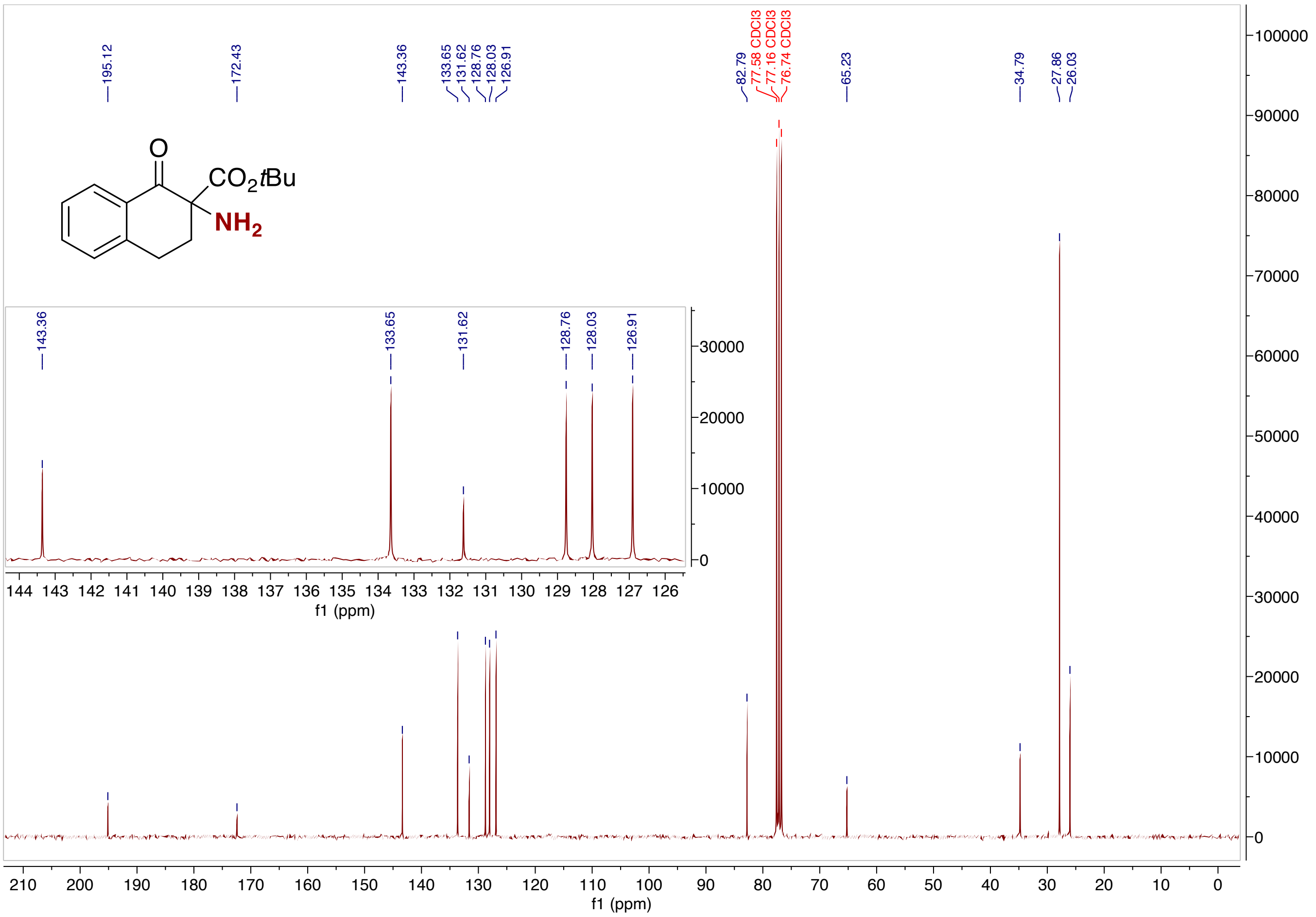

4b, ^{1}H NMR (300 MHz, CDCl$_3$, 298 K)

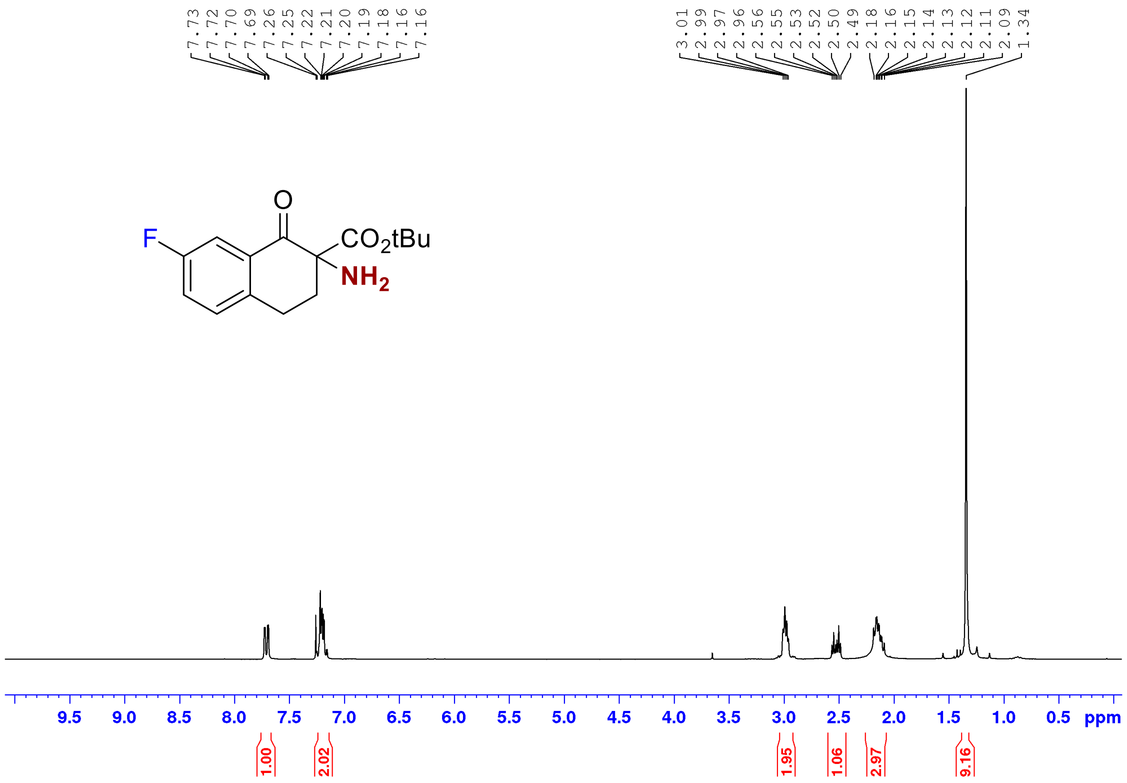

4b, ^{13}C NMR (75 MHz, $CDCl_3$, 298 K)

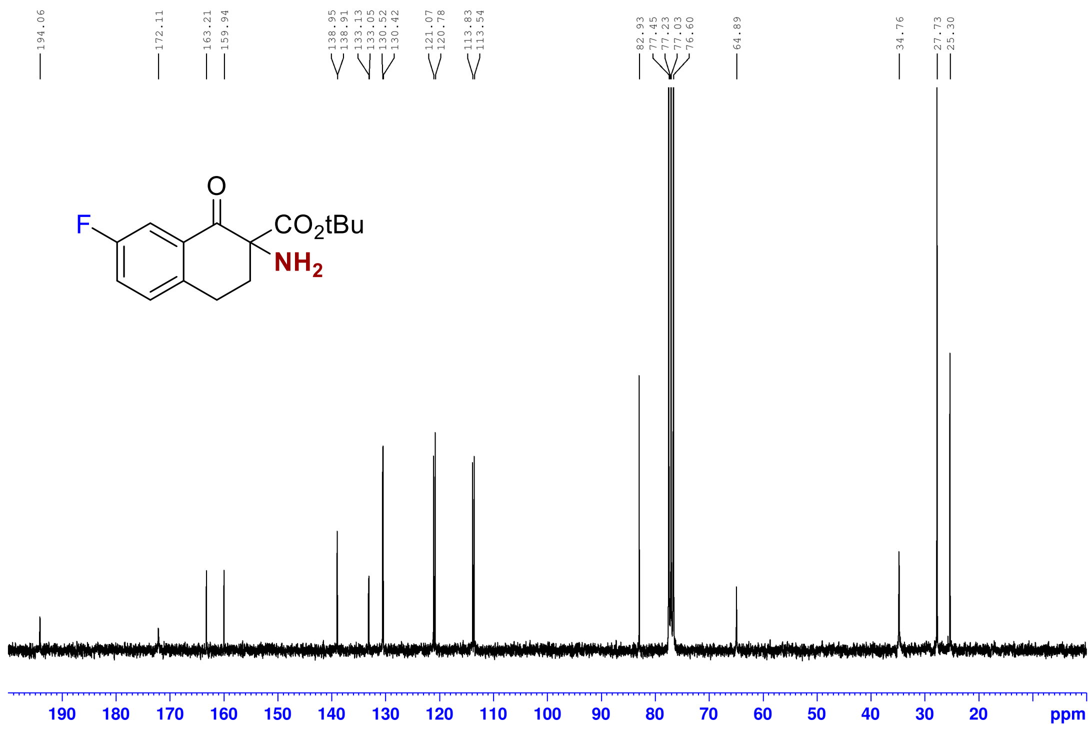

4b, ^{19}F NMR (282 MHz, $CDCl_3$, 298 K)

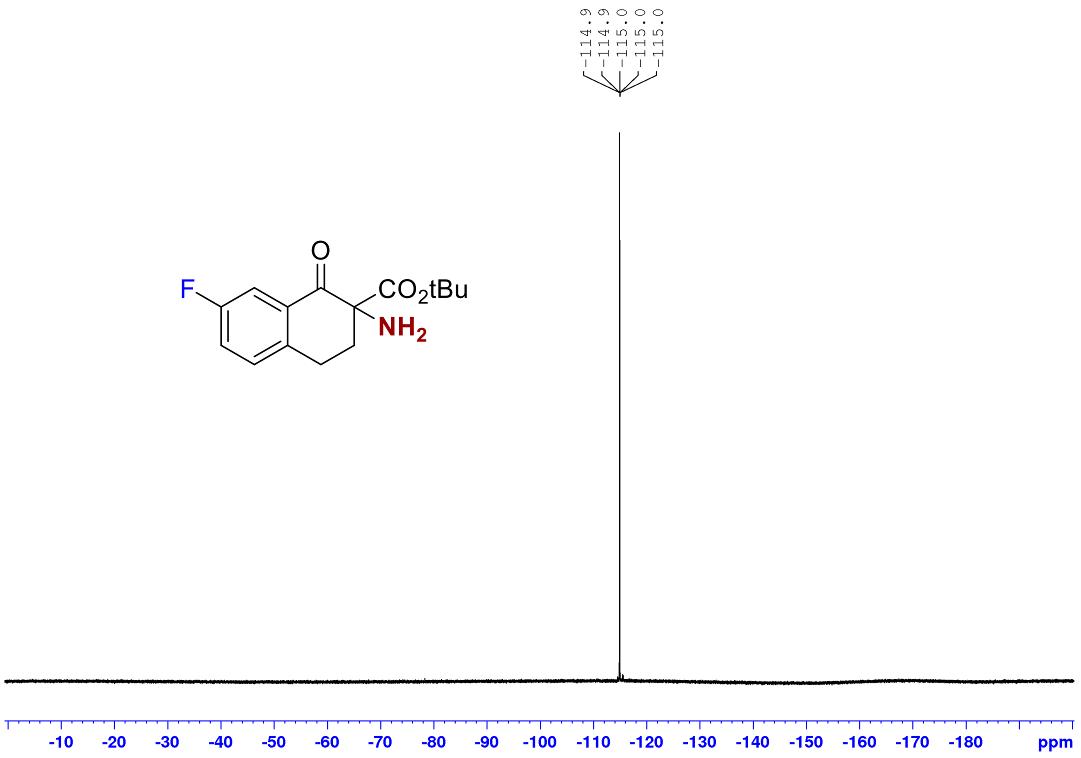

4c, ¹H NMR (300 MHz, $CDCl_3$, 298 K)

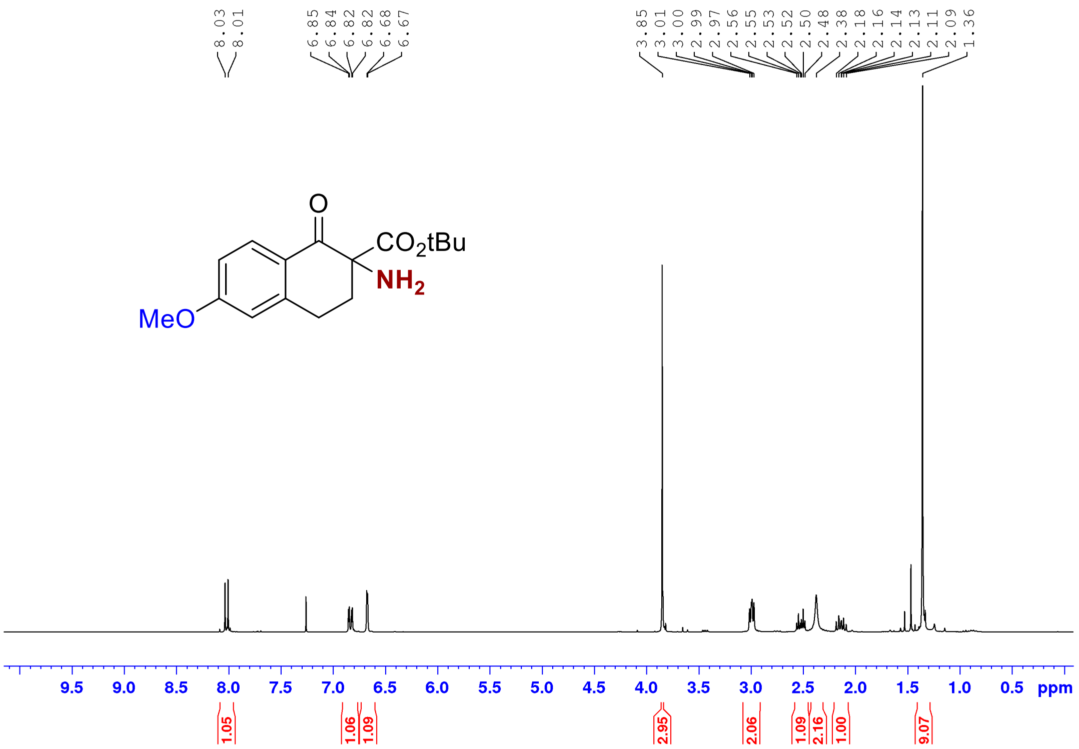

4c, ¹³C NMR (75 MHz, $CDCl_3$, 298 K)

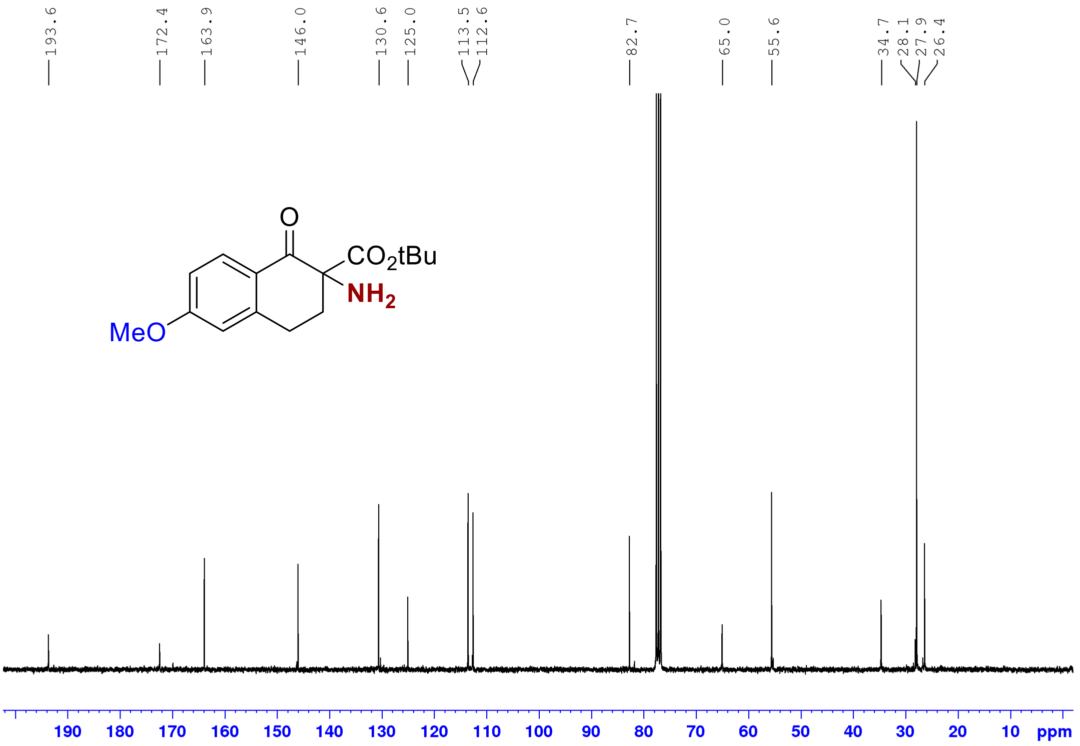

4d, ^{1}H NMR (300 MHz, $CDCl_3$, 298 K)

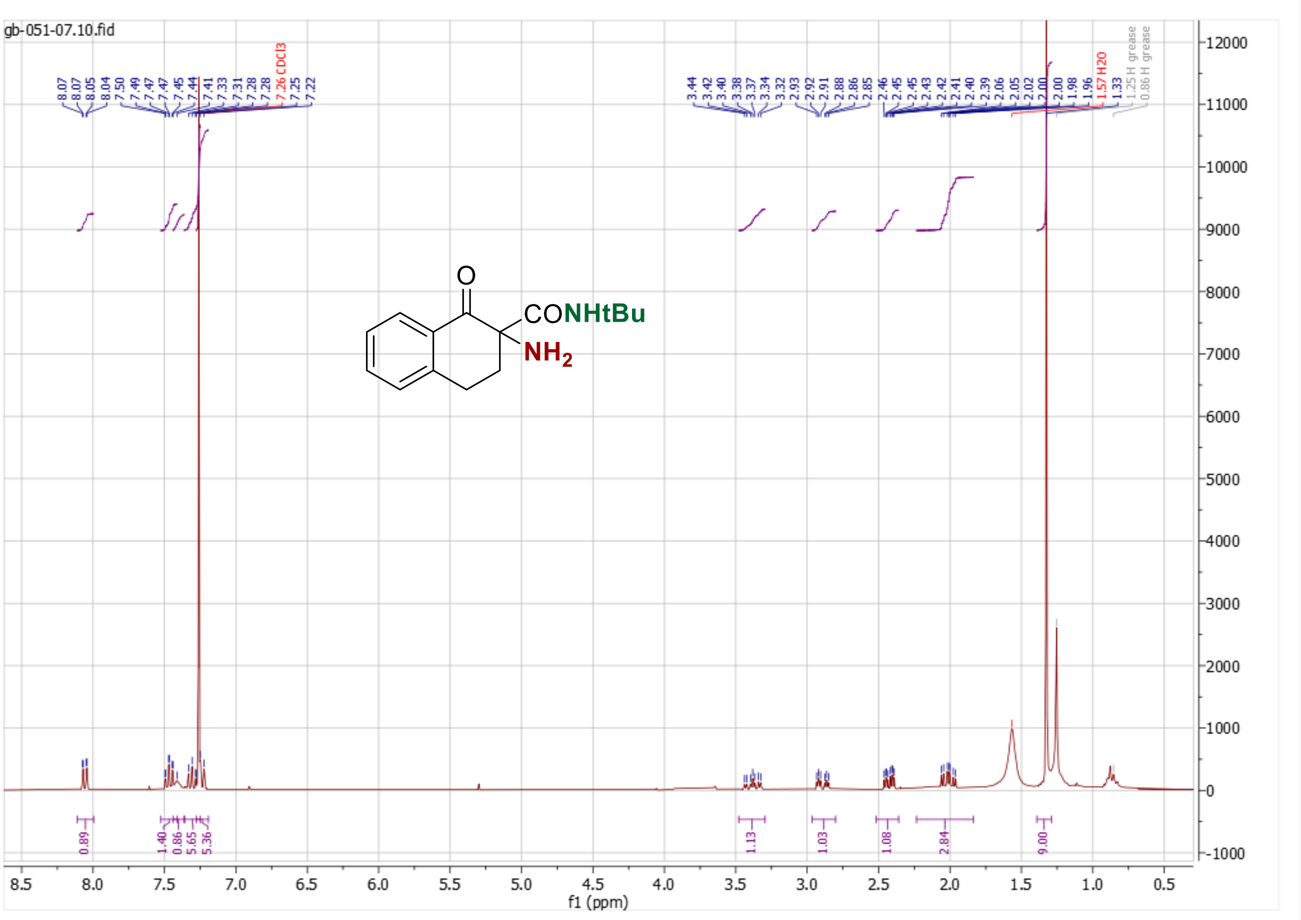

4d, ^{13}C NMR (75 MHz, $CDCl_3$, 298 K)

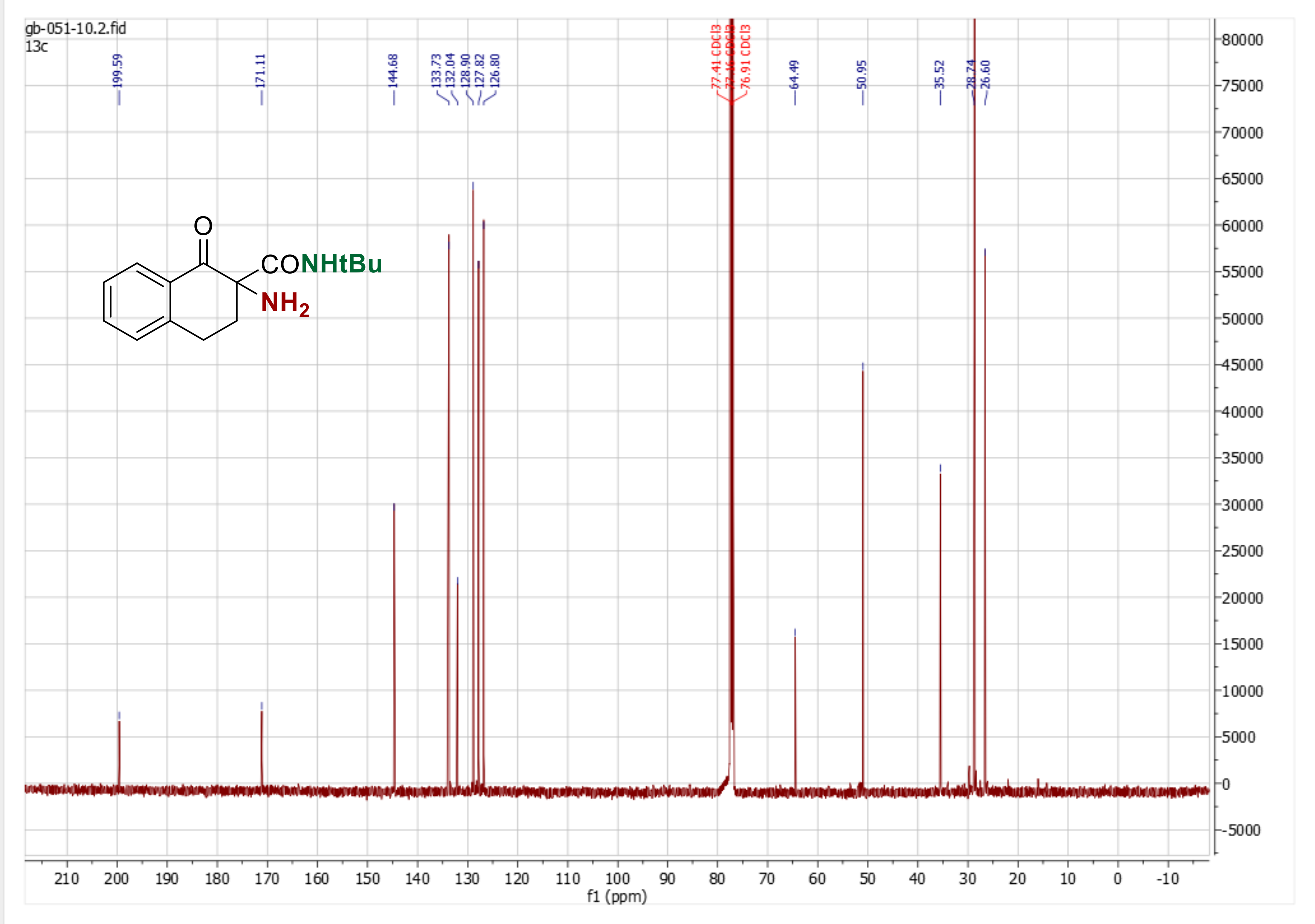

5a, ^{1}H NMR (300 MHz, $CDCl_3$, 298 K)

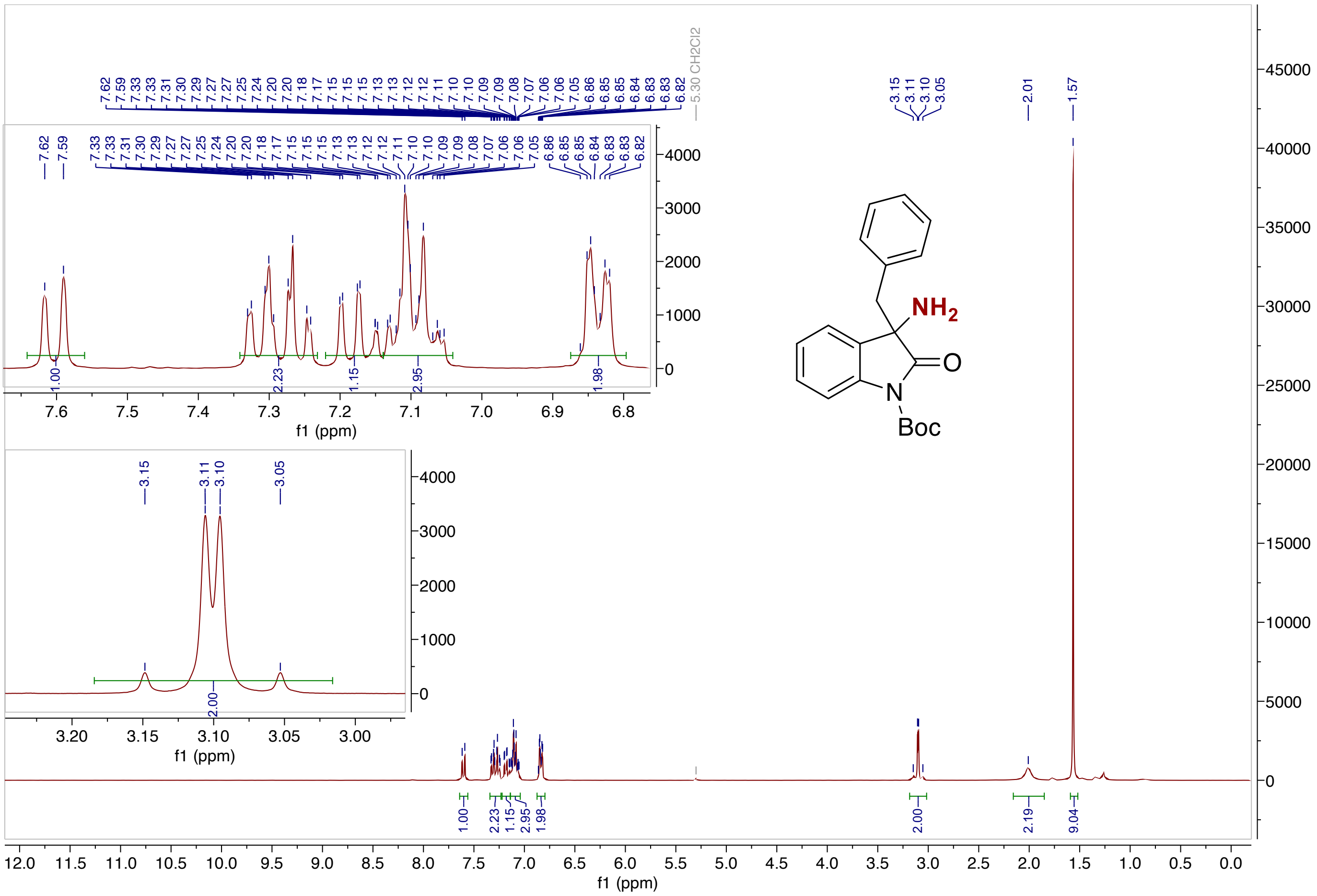

5a, ^{13}C NMR (75 MHz, $CDCl_3$, 298 K)

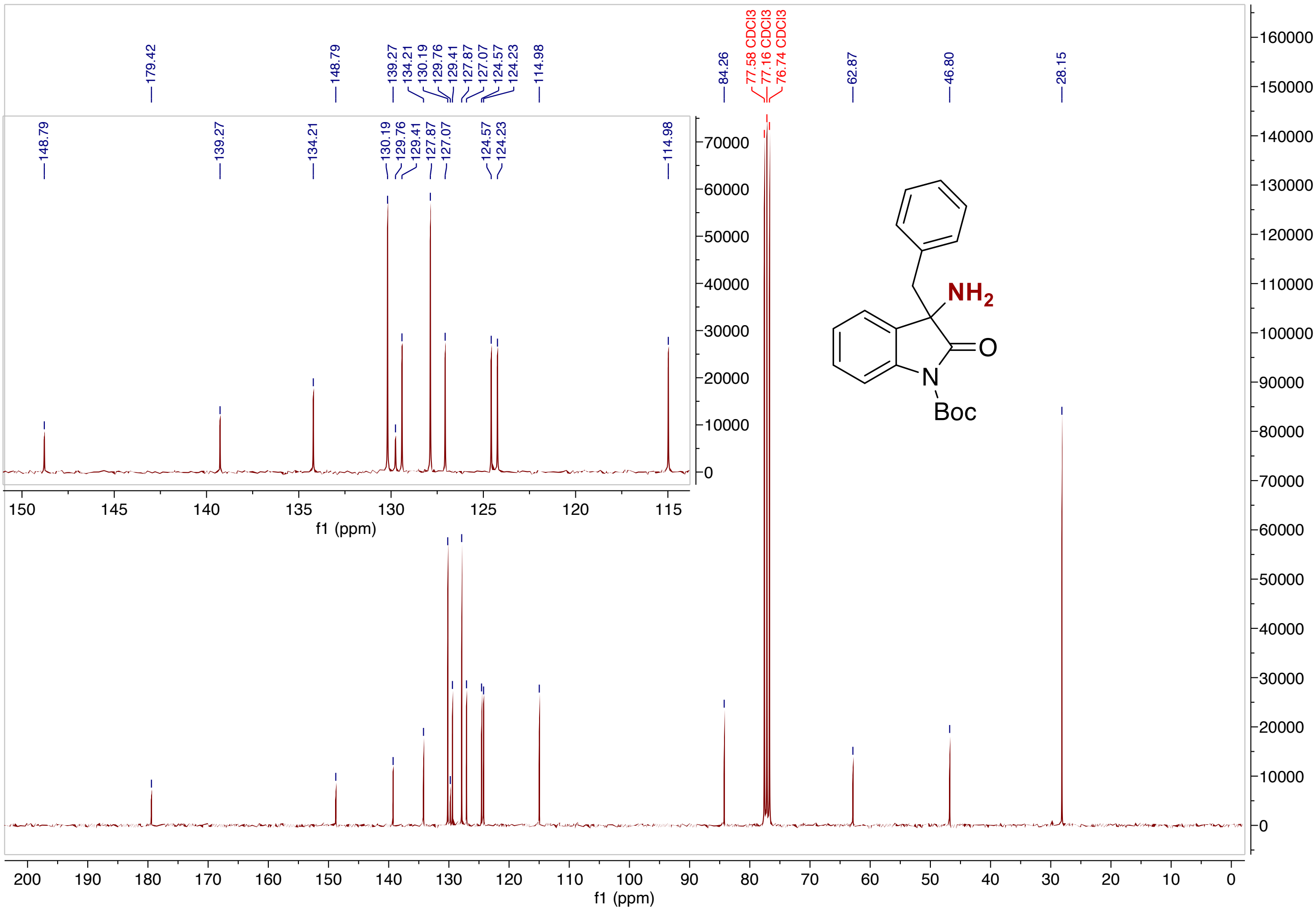

5b, ^{1}H NMR (300 MHz, $CDCl_3$, 298 K)

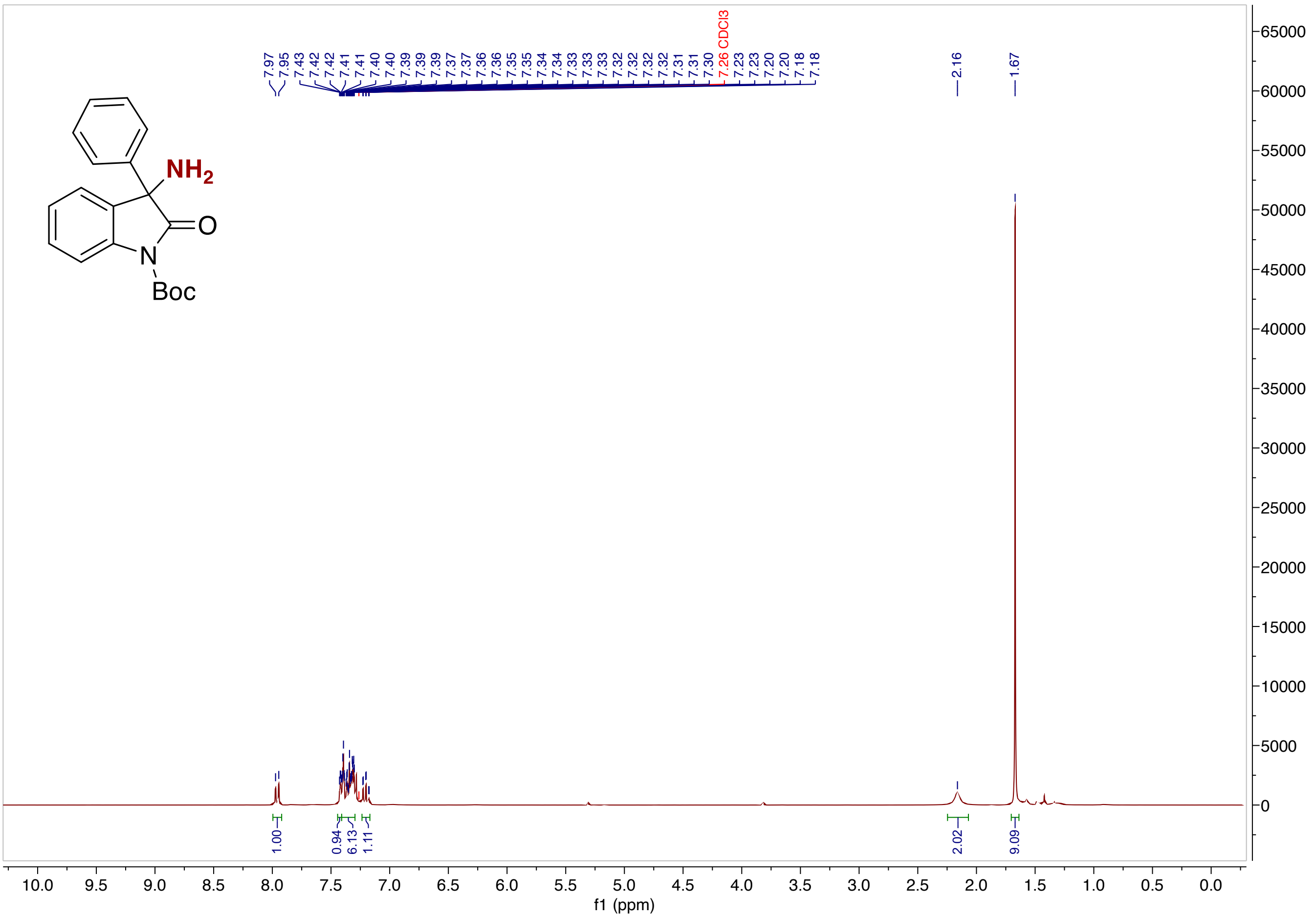

5b, ^{13}C NMR (75 MHz, $CDCl_3$, 298 K)

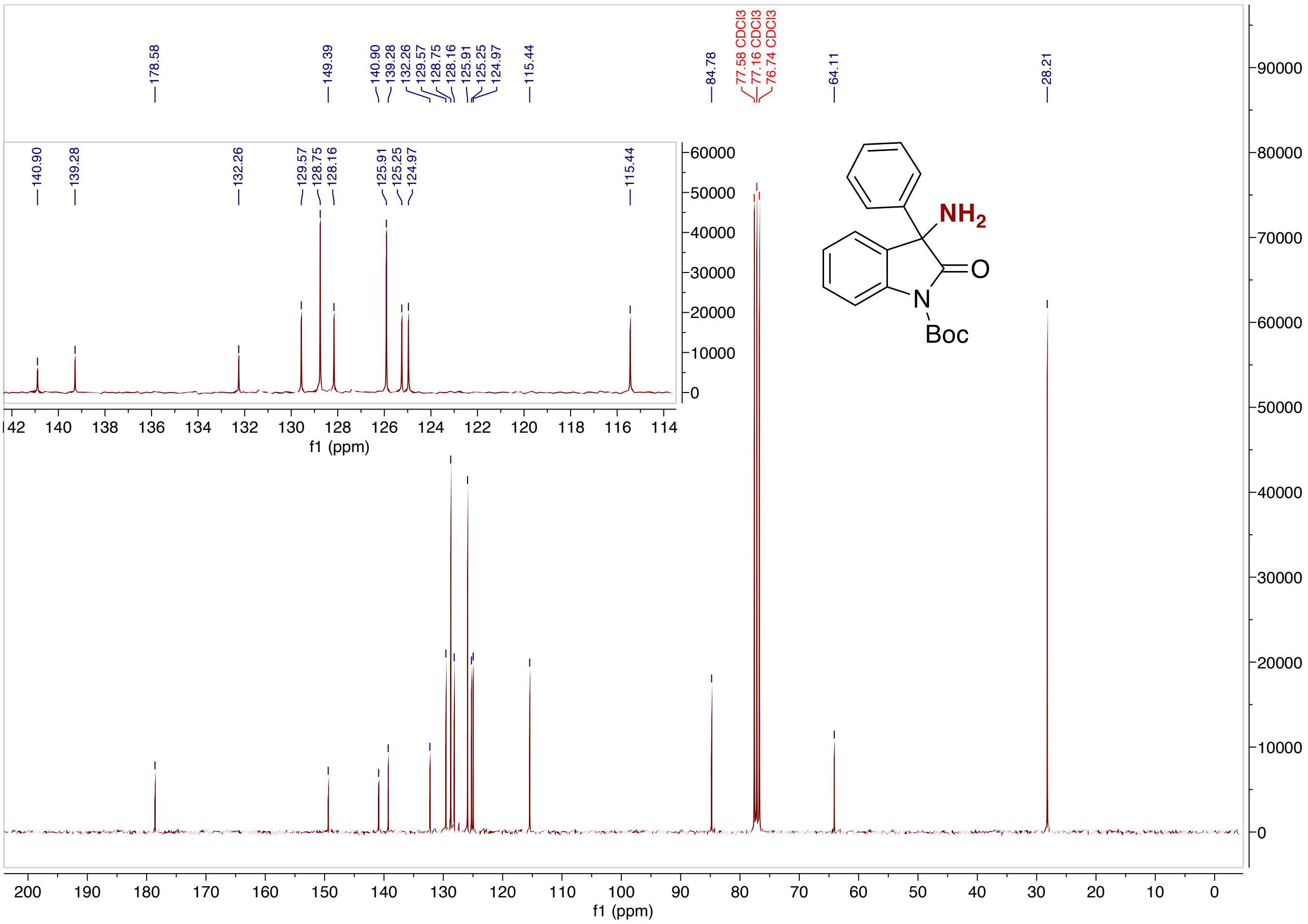

5c, ^{1}H NMR (300 MHz, $CDCl_3$, 298 K)

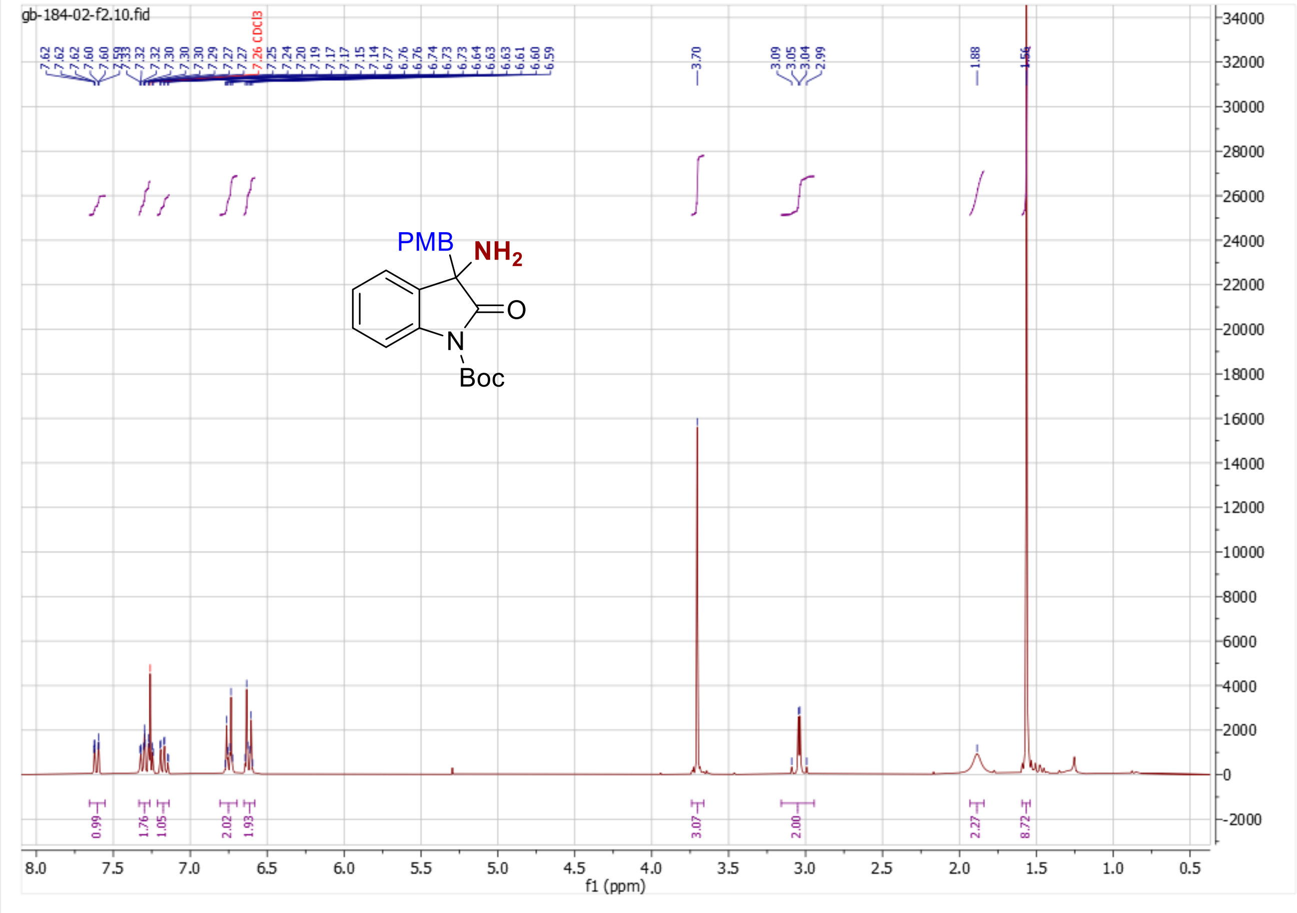

5c, ^{13}C NMR (75 MHz, $CDCl_3$, 298 K)

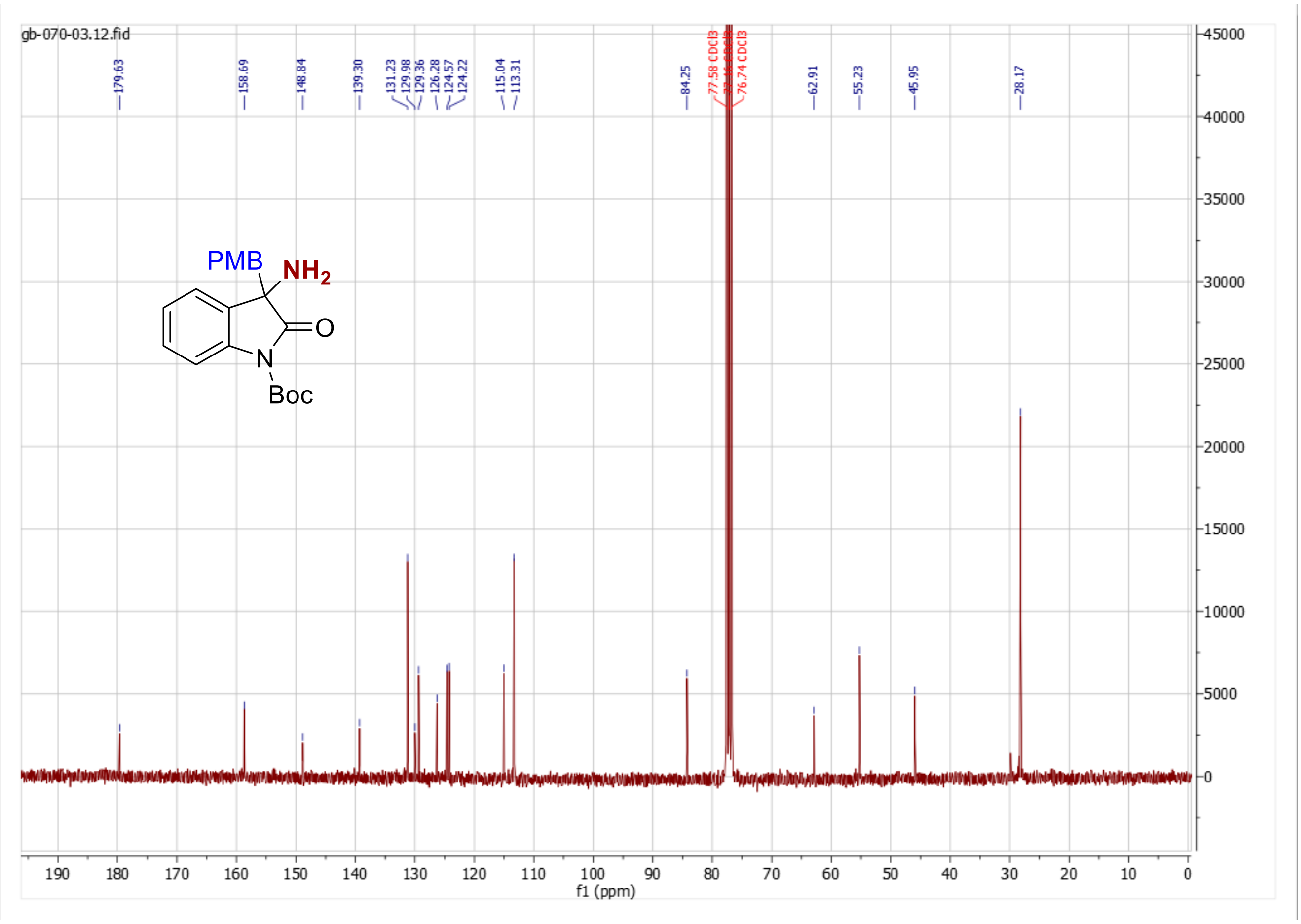

6a, ^{1}H NMR (300 MHz, $CDCl_3$, 298 K)

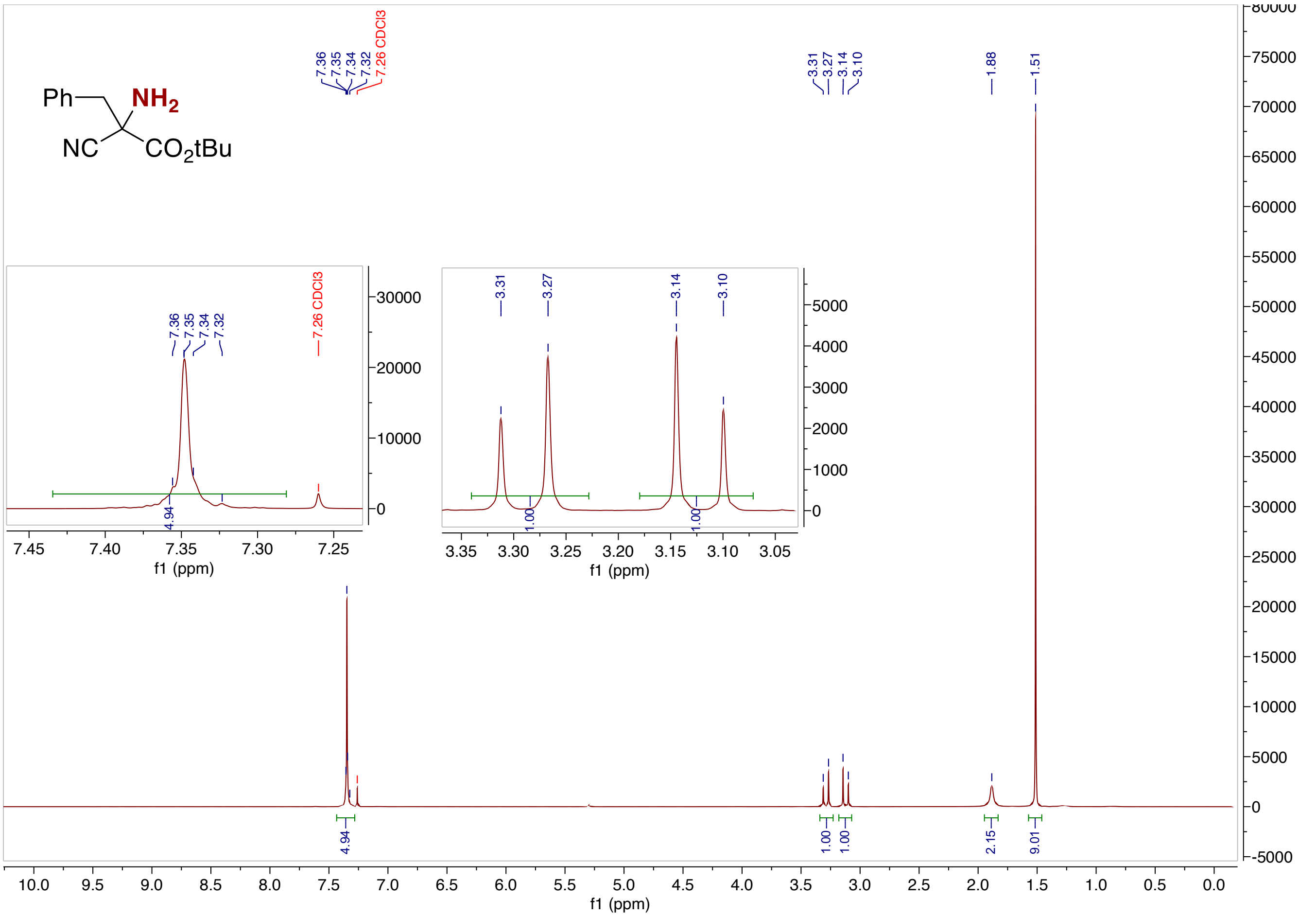

6a, ^{13}C NMR (75 MHz, $CDCl_3$, 298 K)

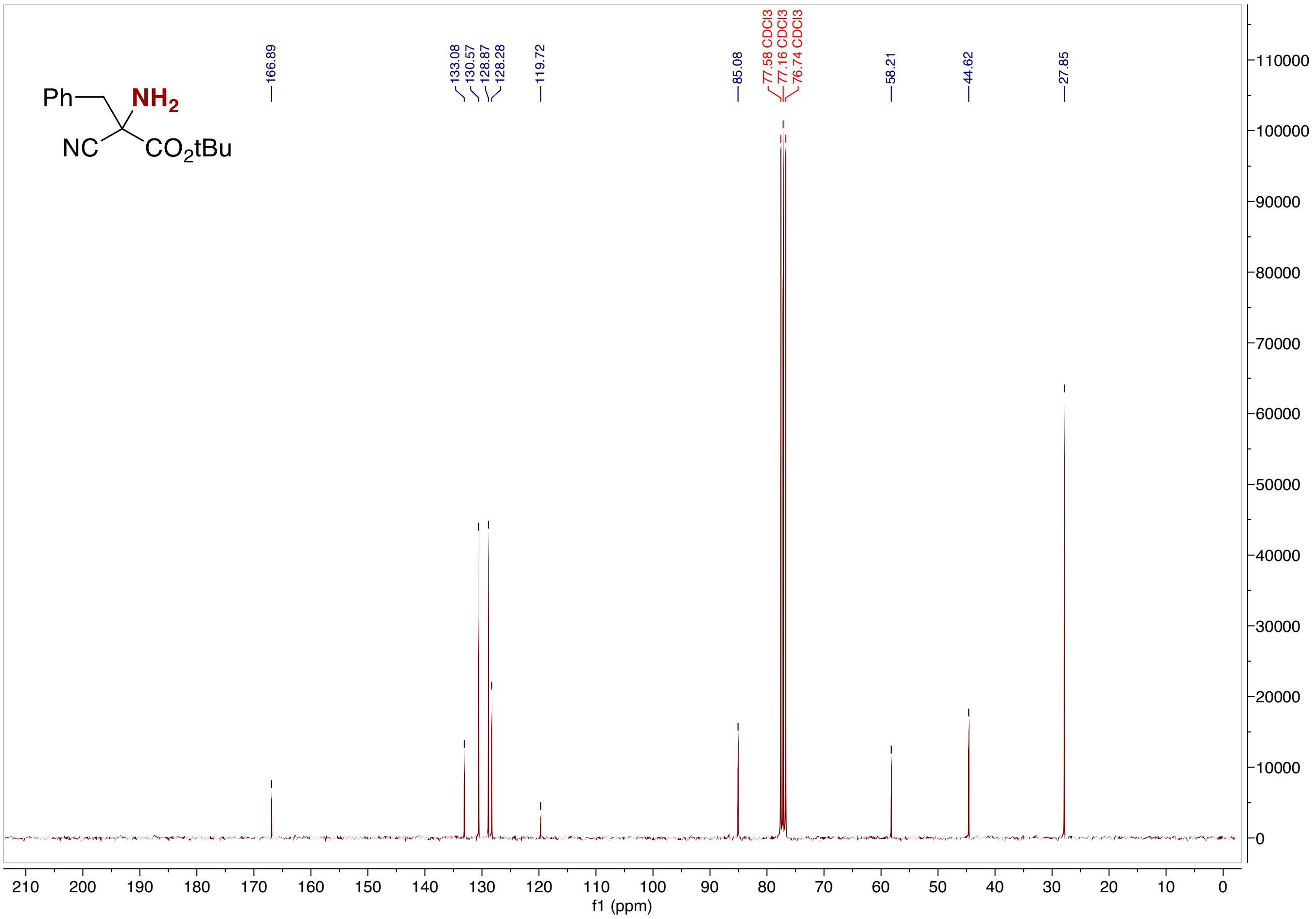

6b, ^{1}H NMR (300 MHz, $CDCl_3$, 298 K)

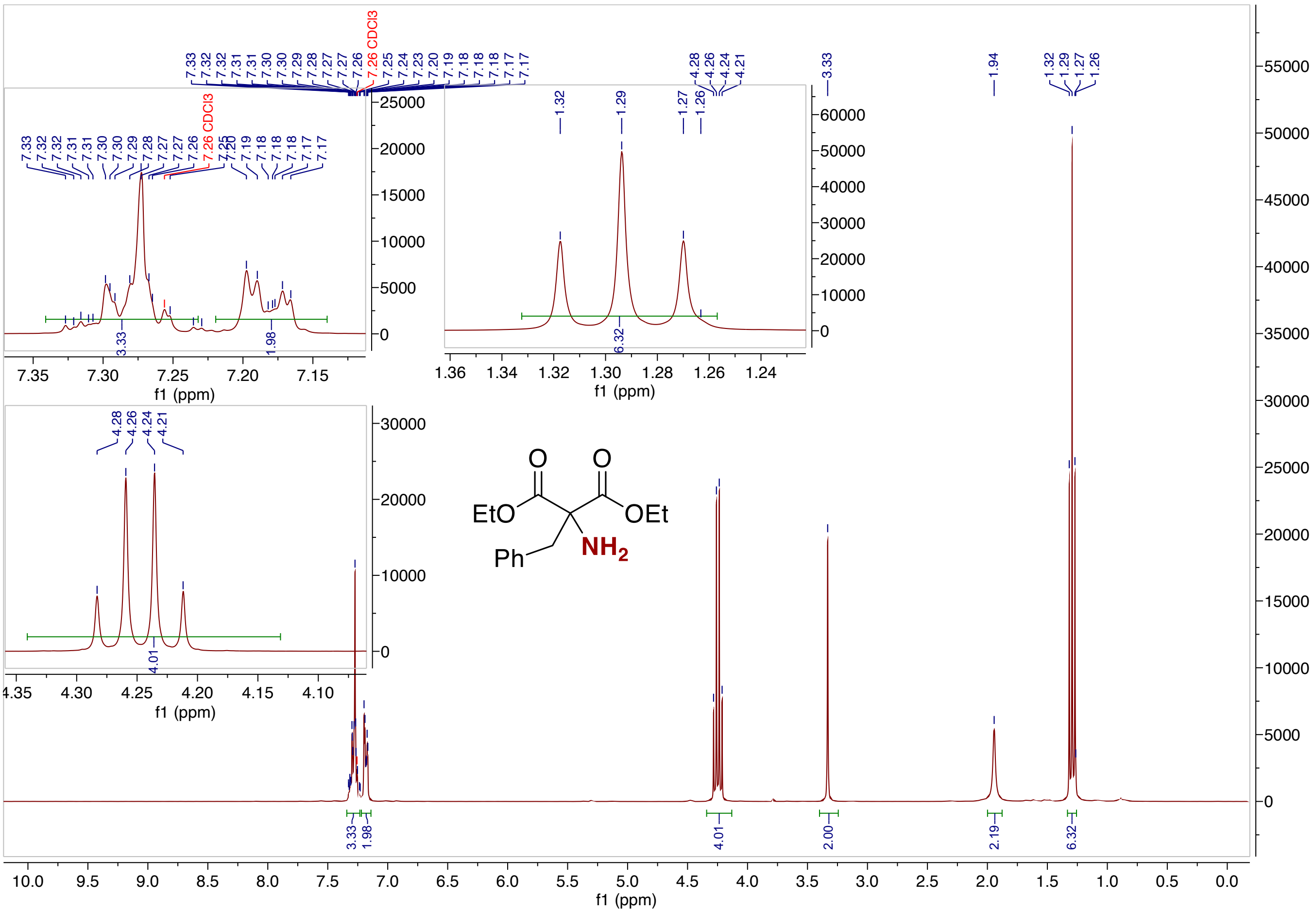

6b, ^{13}C NMR (75 MHz, $CDCl_3$, 298 K)

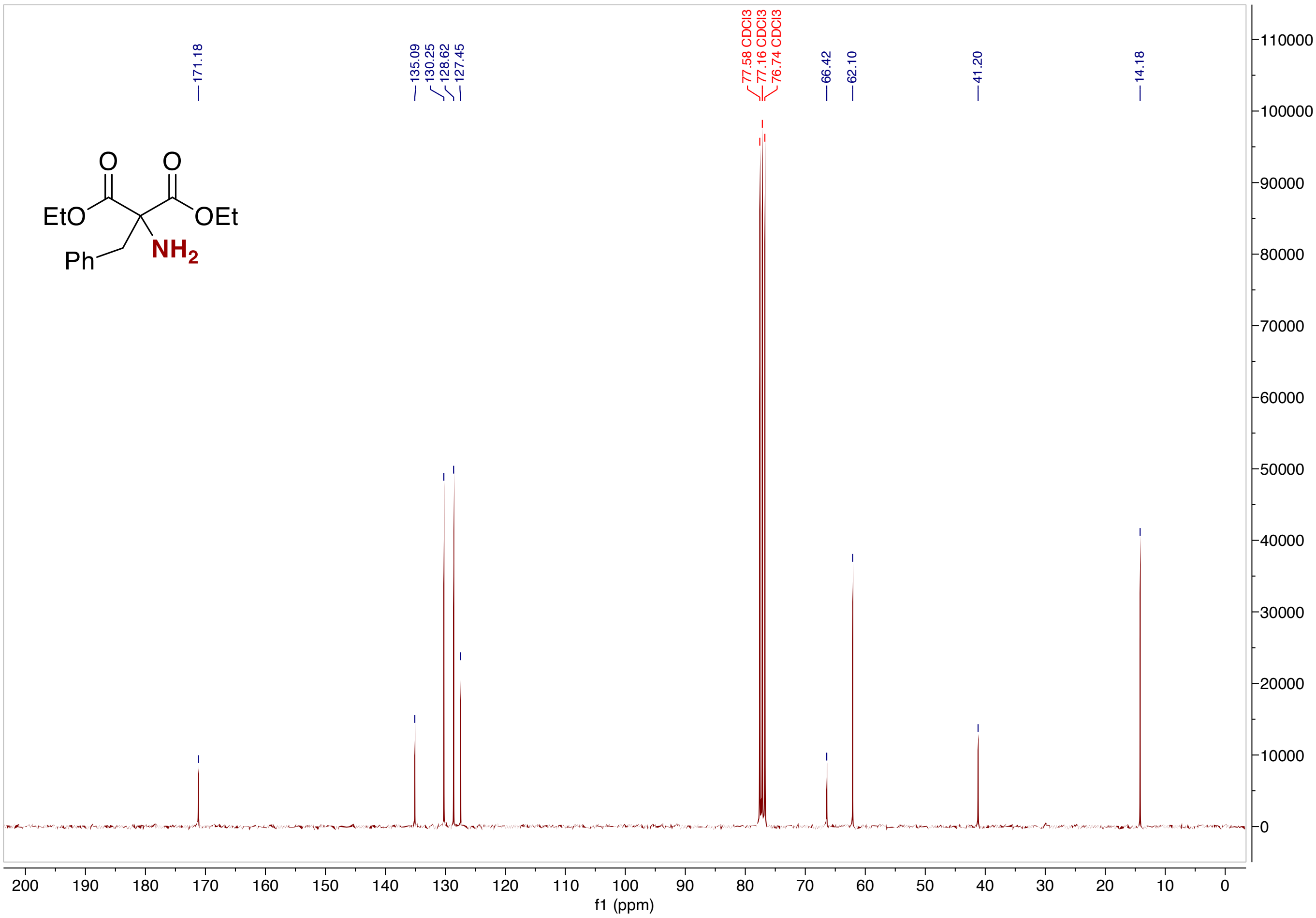

6c, ^{1}H NMR (300 MHz, $CDCl_3$, 298 K)

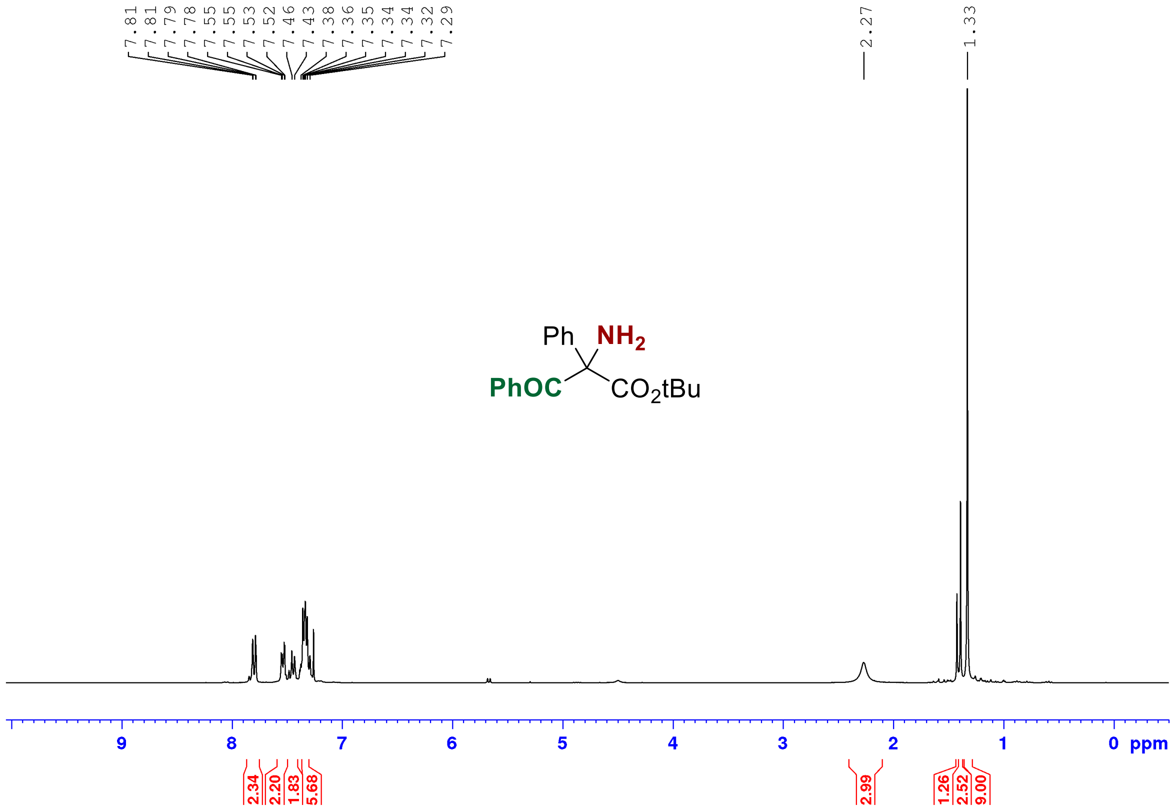

6c, ^{13}C NMR (75 MHz, $CDCl_3$, 298 K)

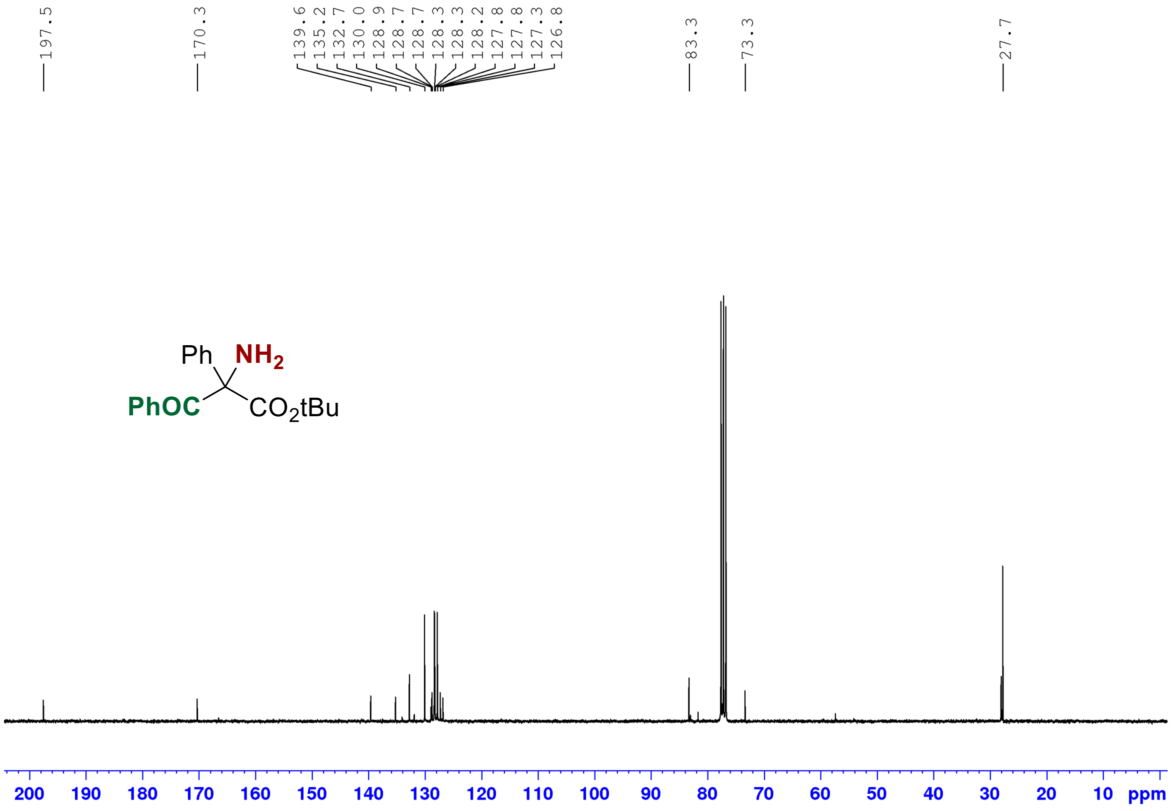

5. IR Spectra

2a, ATR FT-IR (neat)

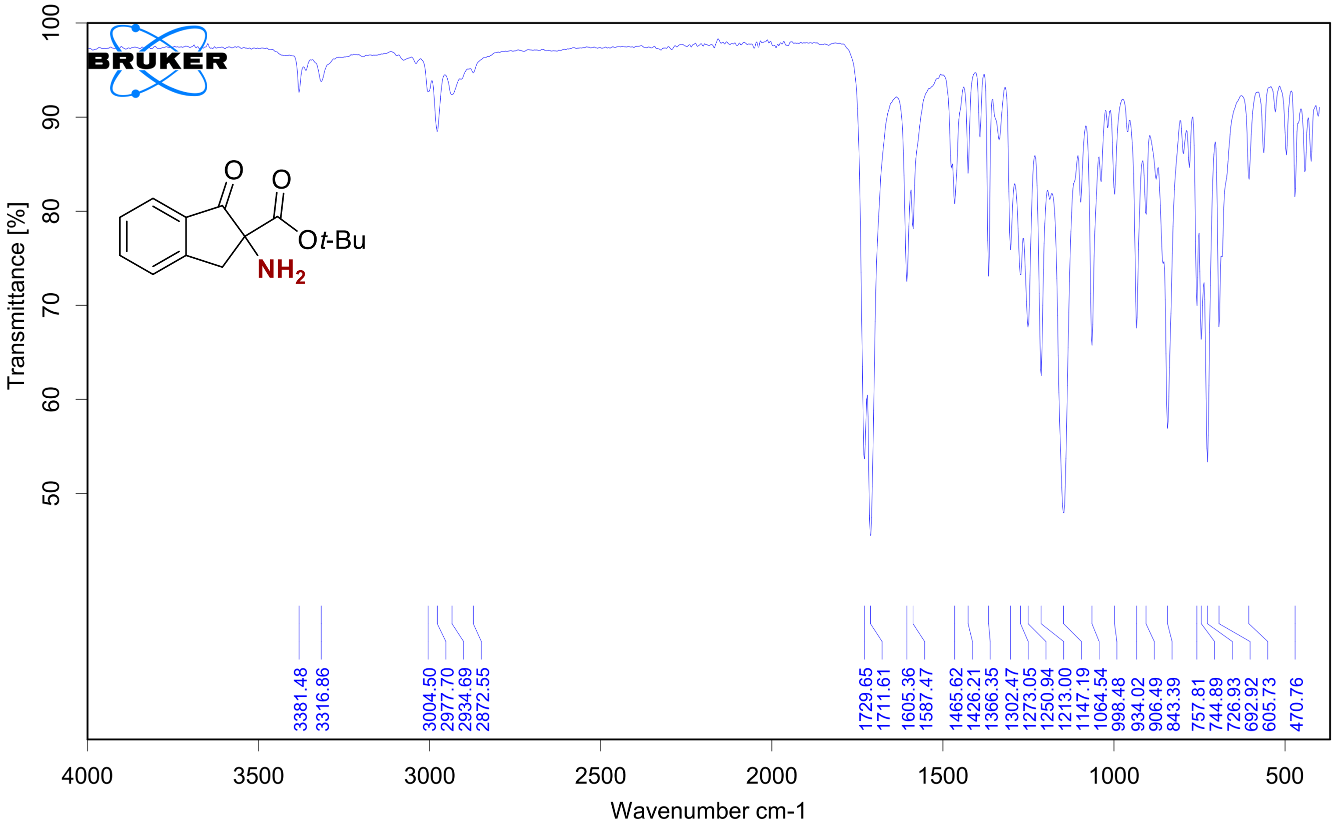

2b, ATR FT-IR (neat)

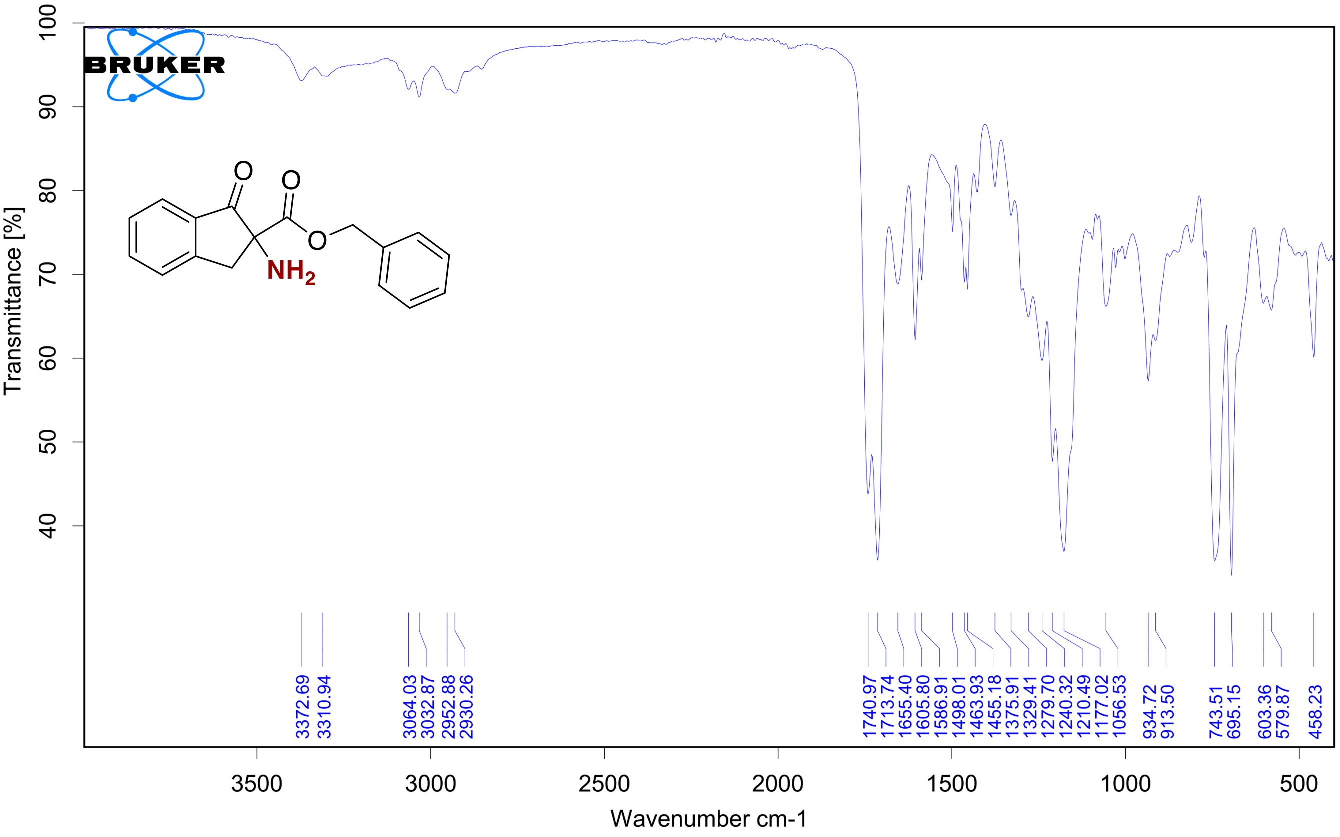

2c, ATR FT-IR (neat)

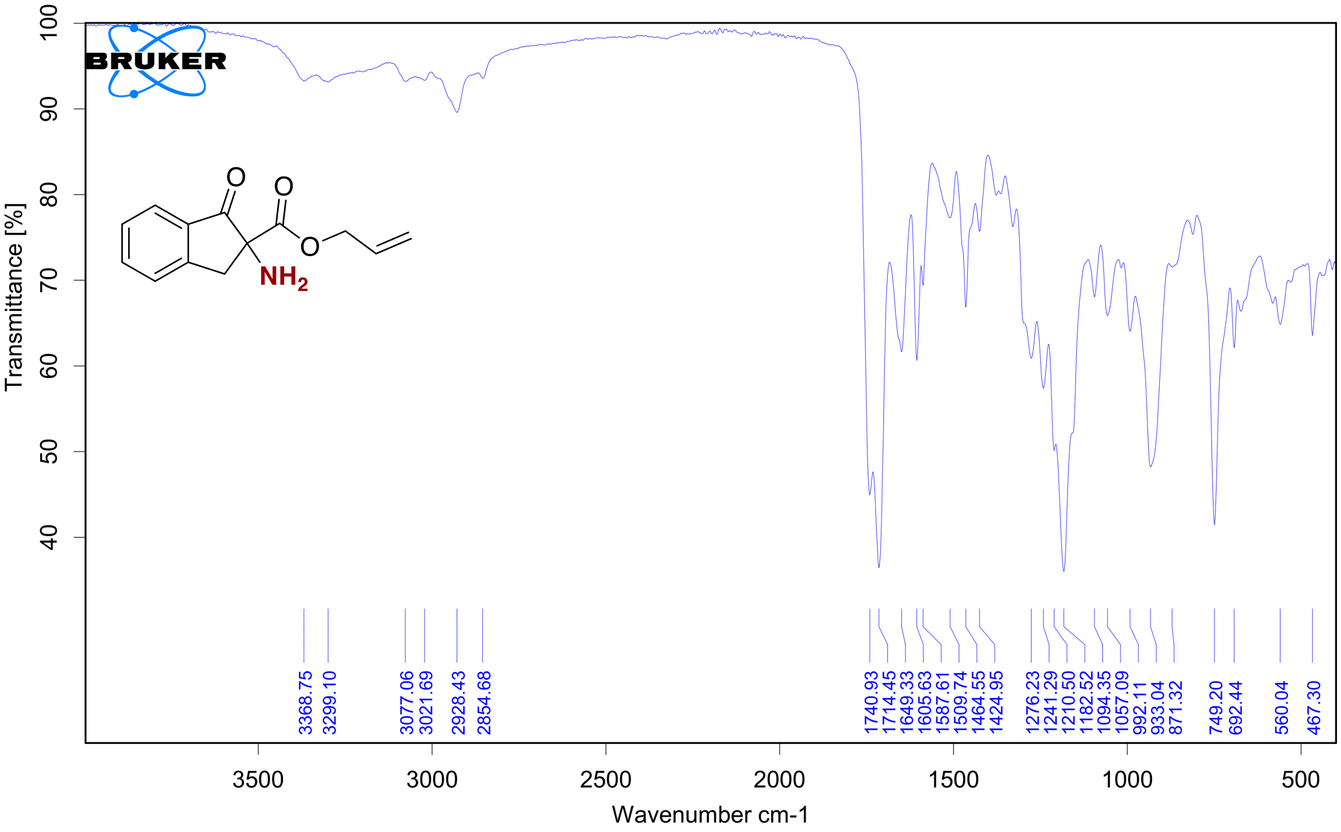

2d, ATR FT-IR (neat)

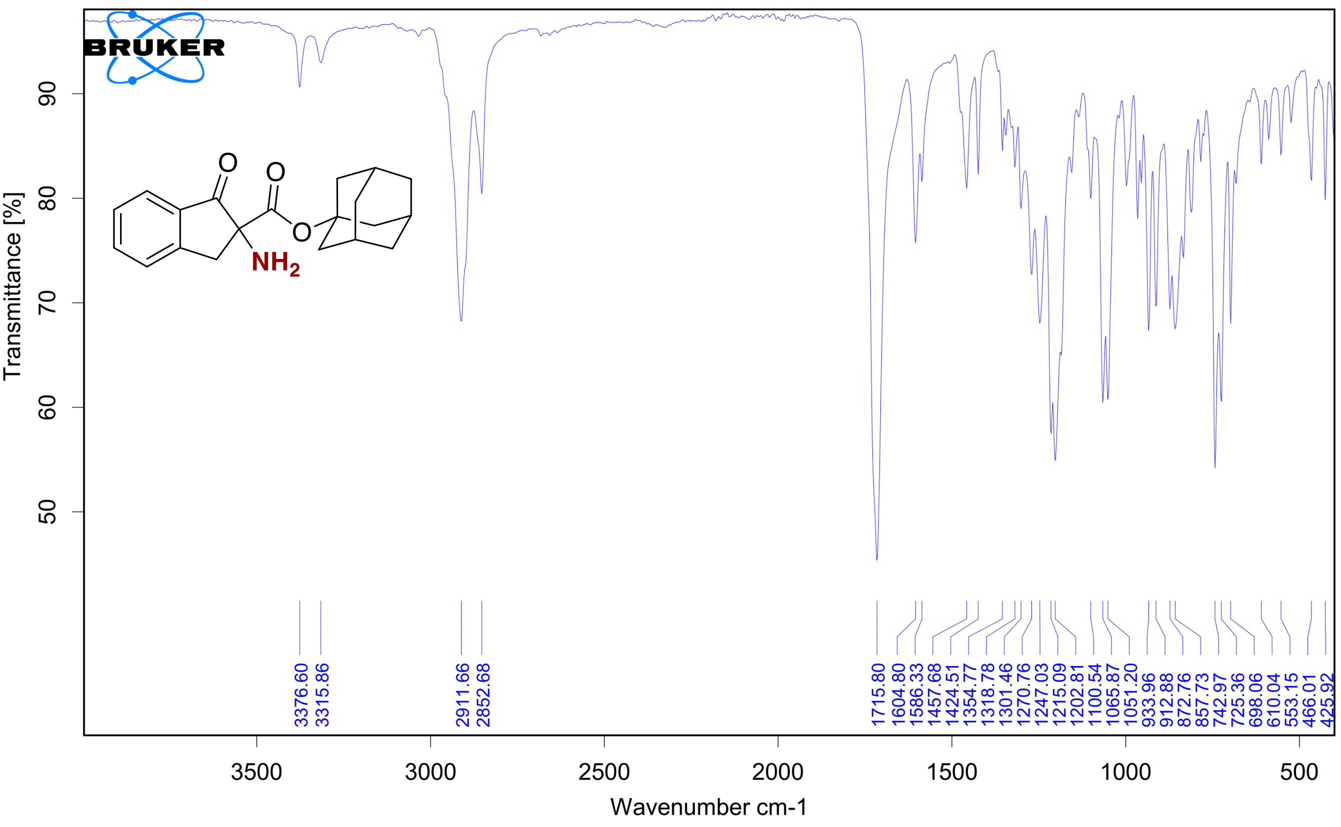

2e, ATR FT-IR (neat)

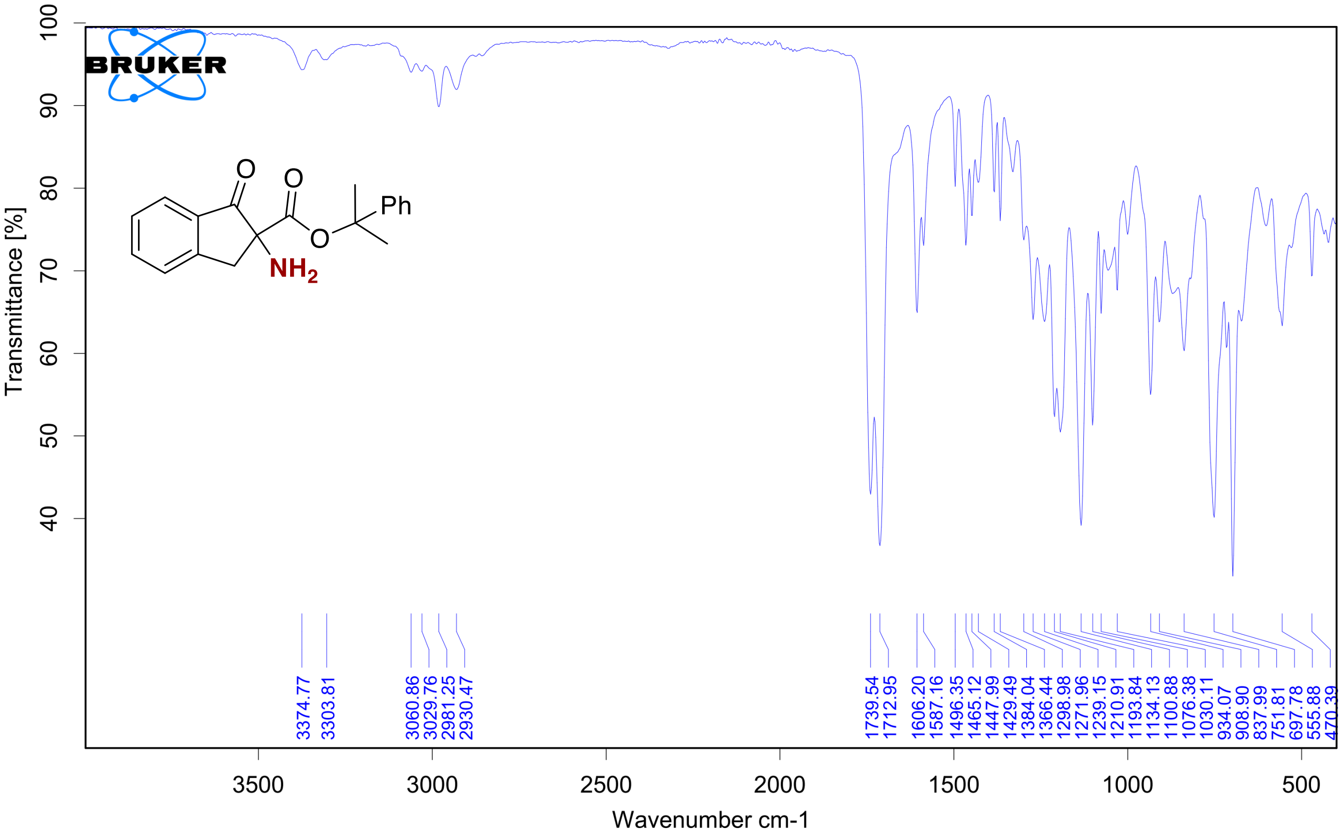

2f, ATR FT-IR (neat)

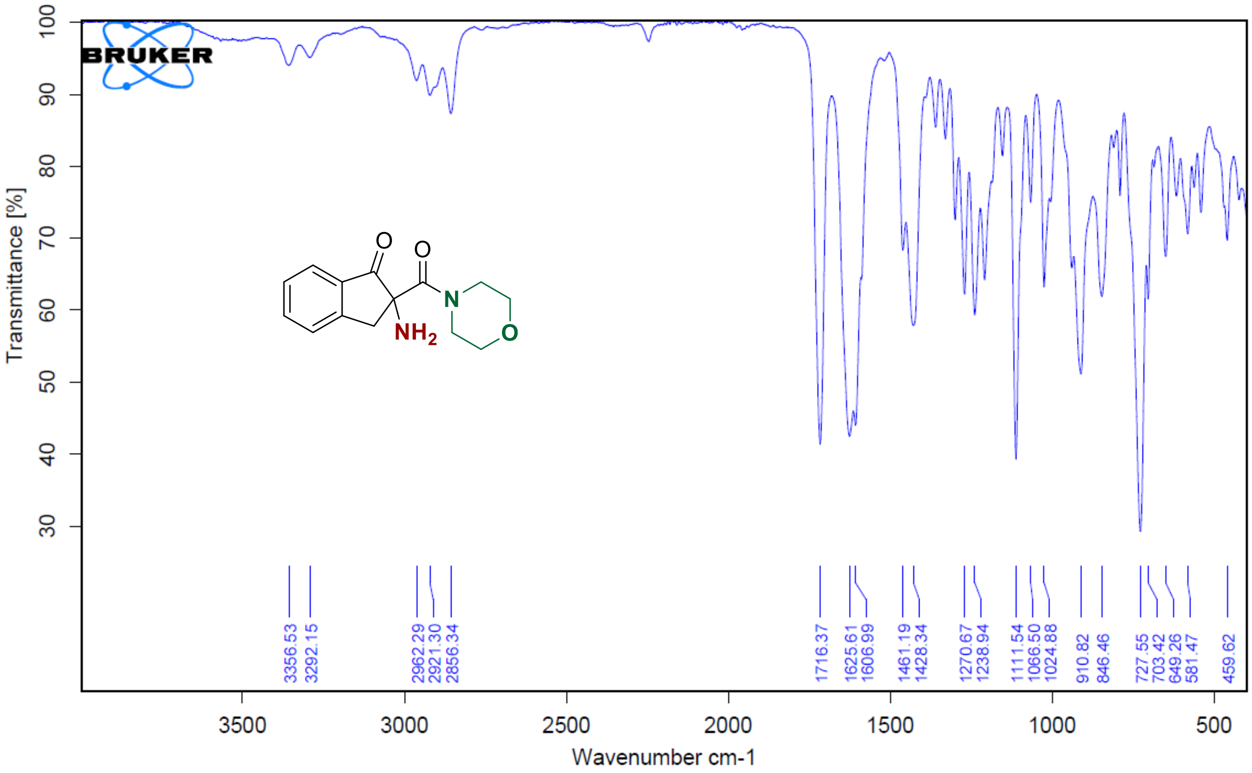

2g, ATR FT-IR (neat)

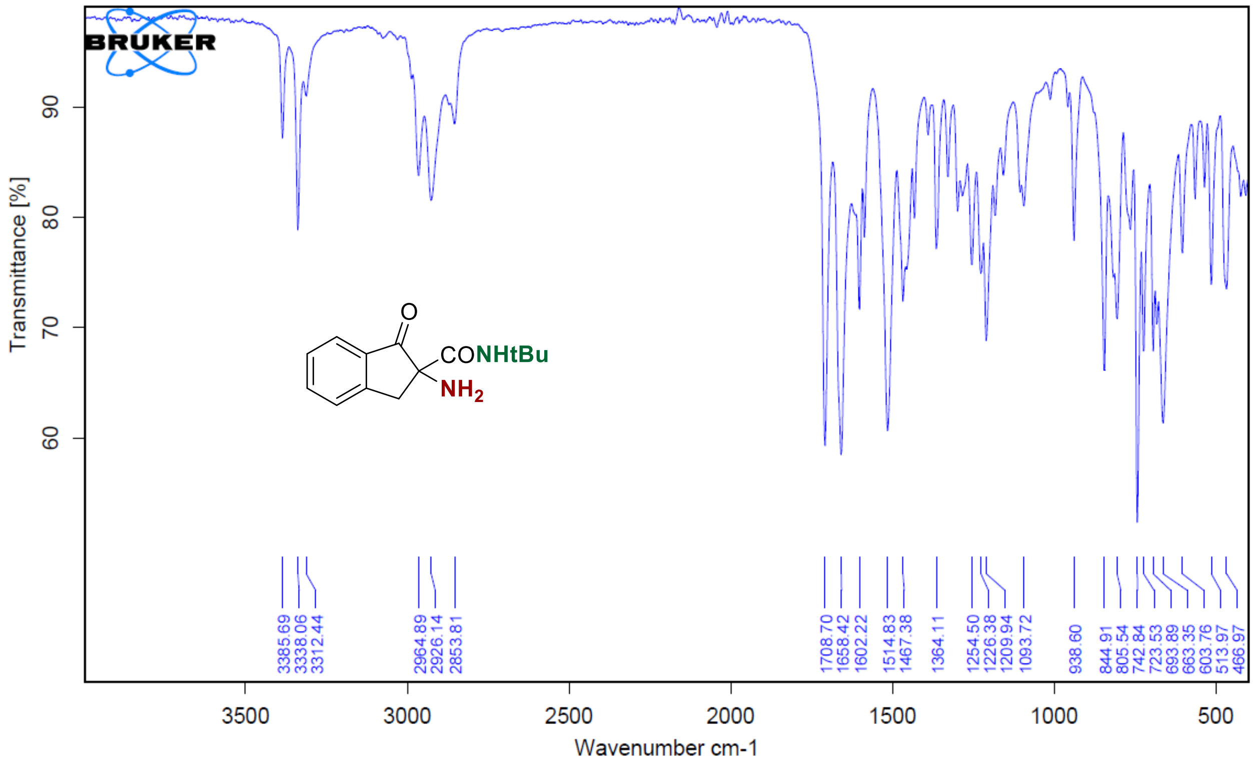

2h, ATR FT-IR (neat)

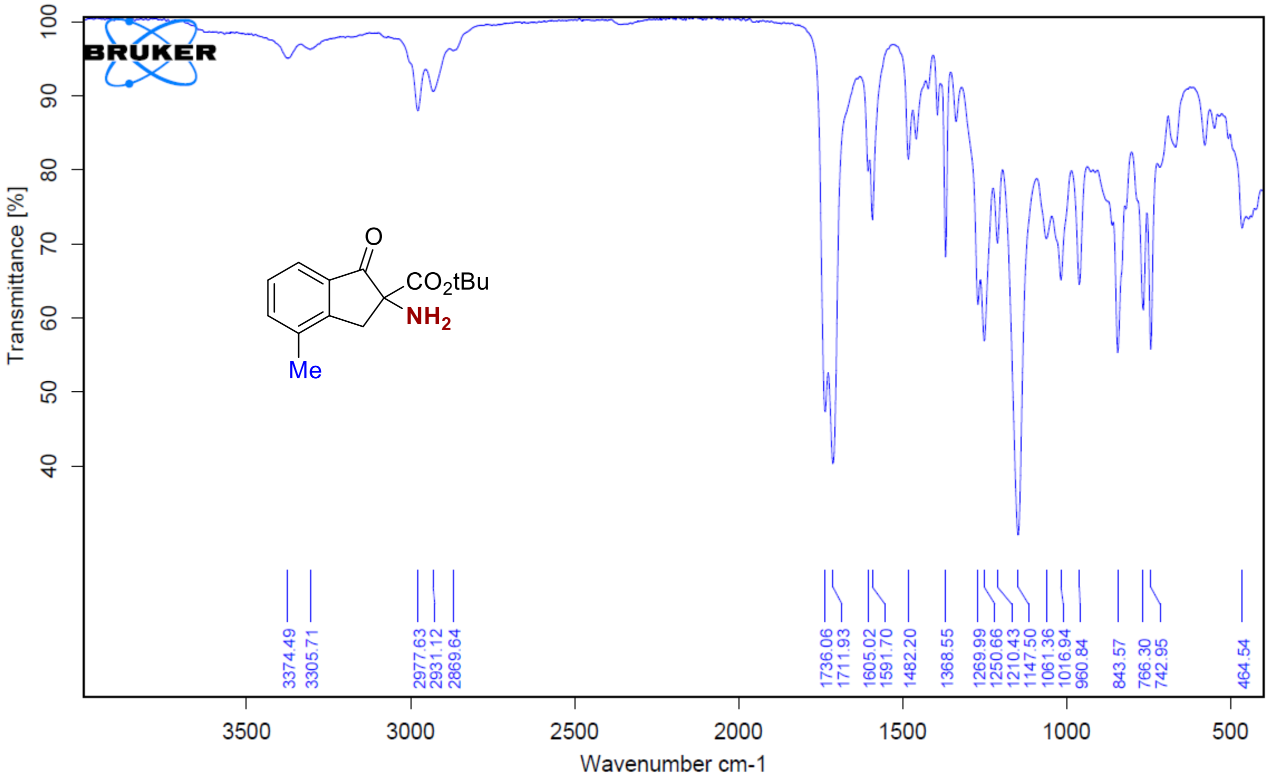

2i, ATR FT-IR (neat)

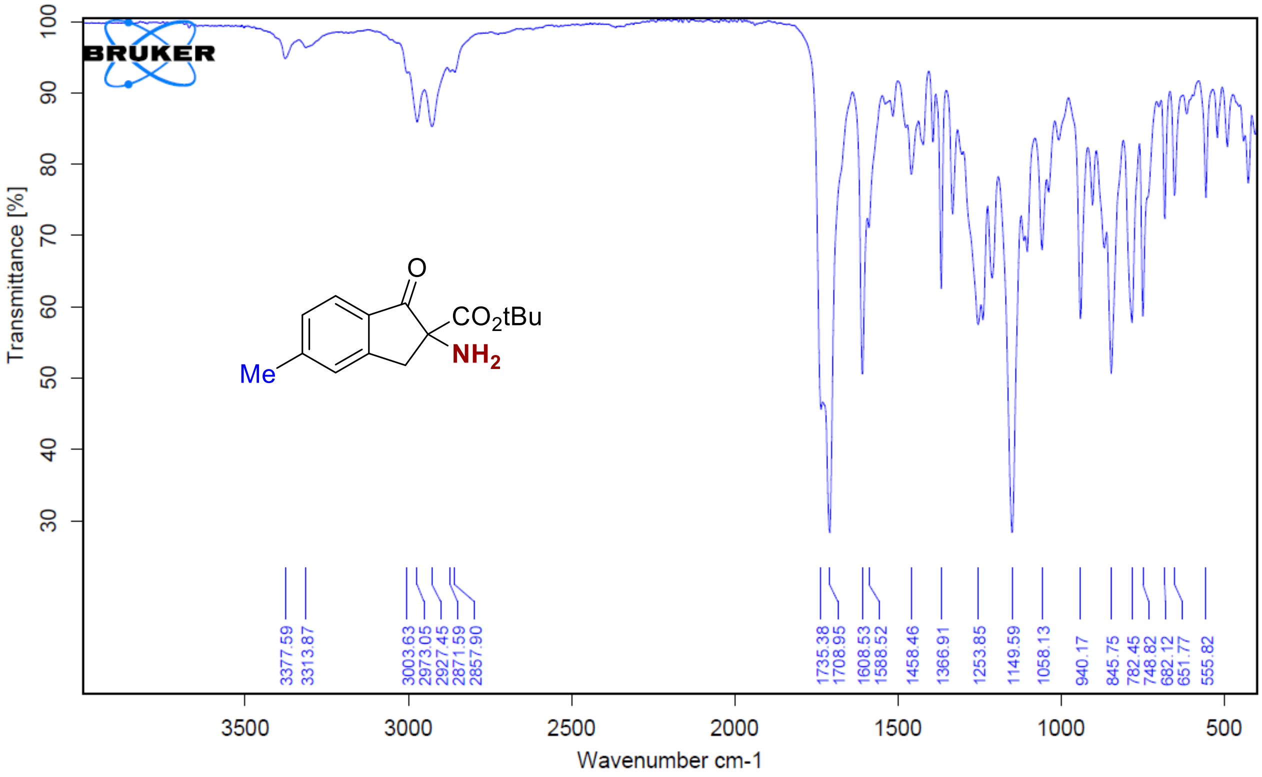

2j, ATR FT-IR (neat)

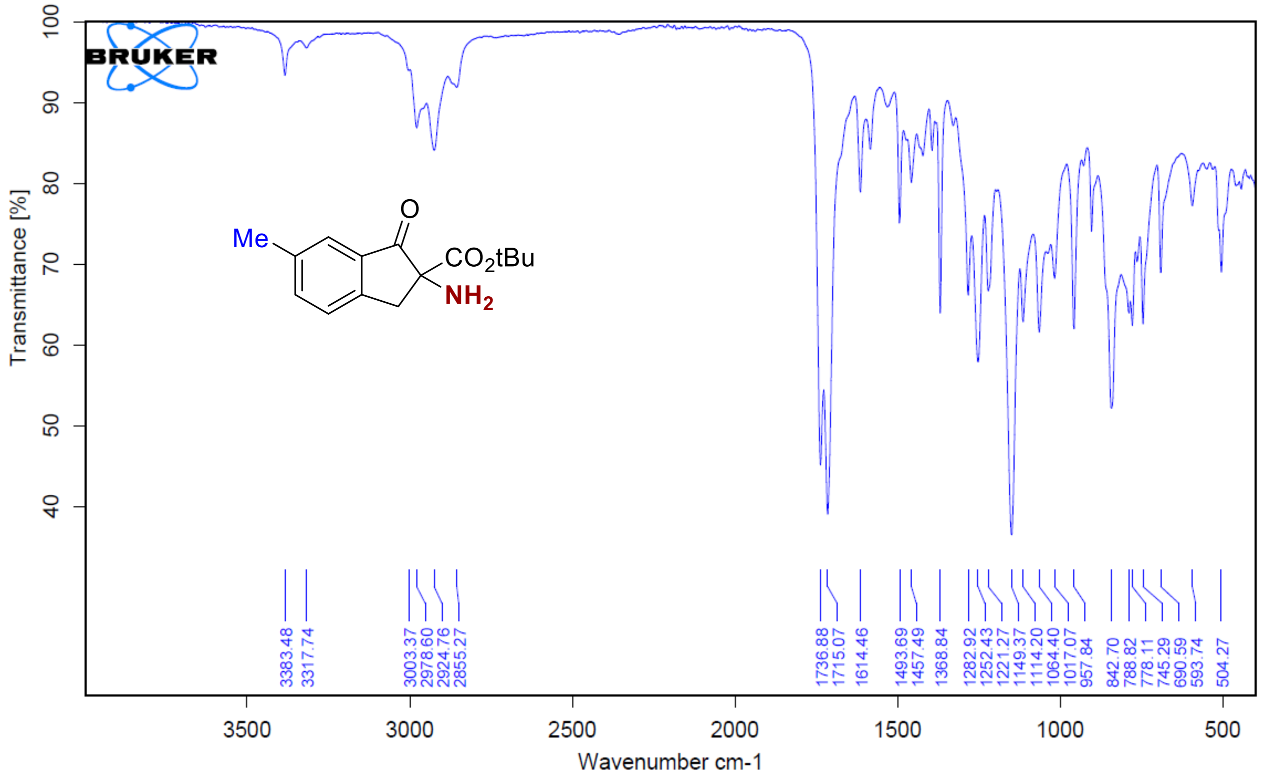

2k, ATR FT-IR (neat)

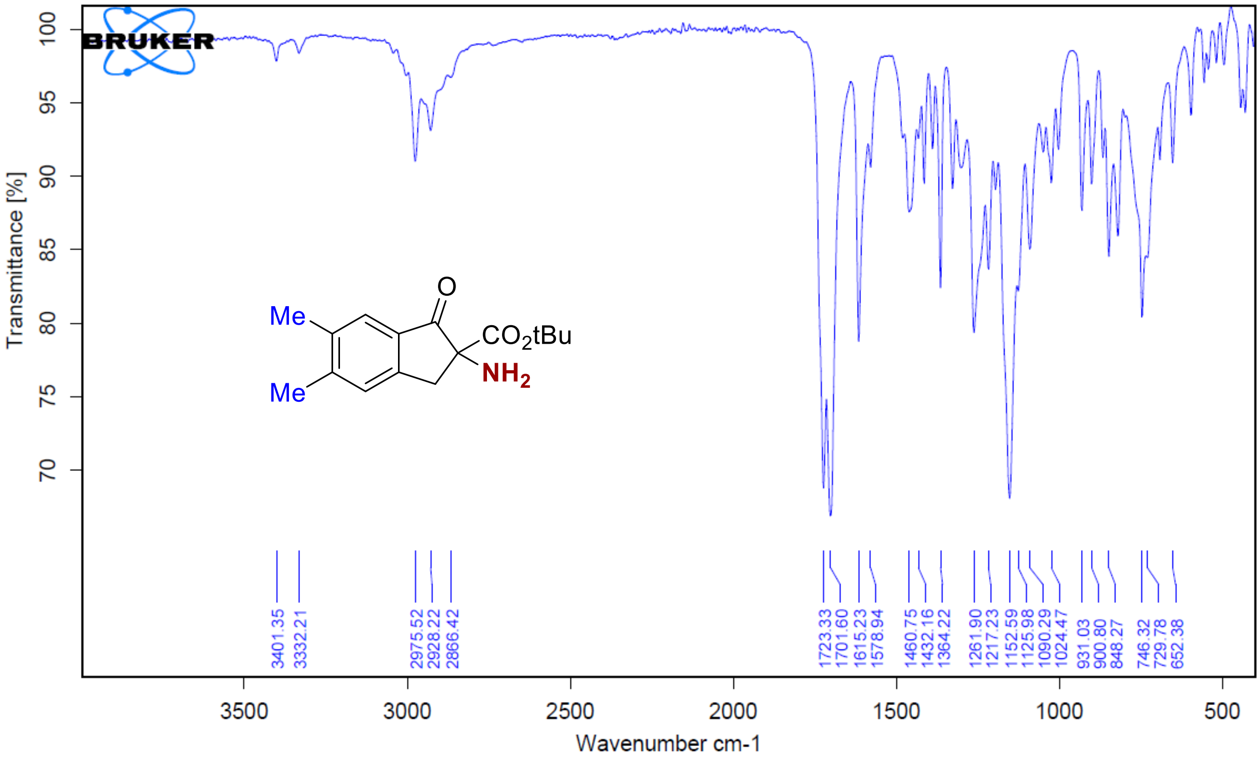

2l, ATR FT-IR (neat)

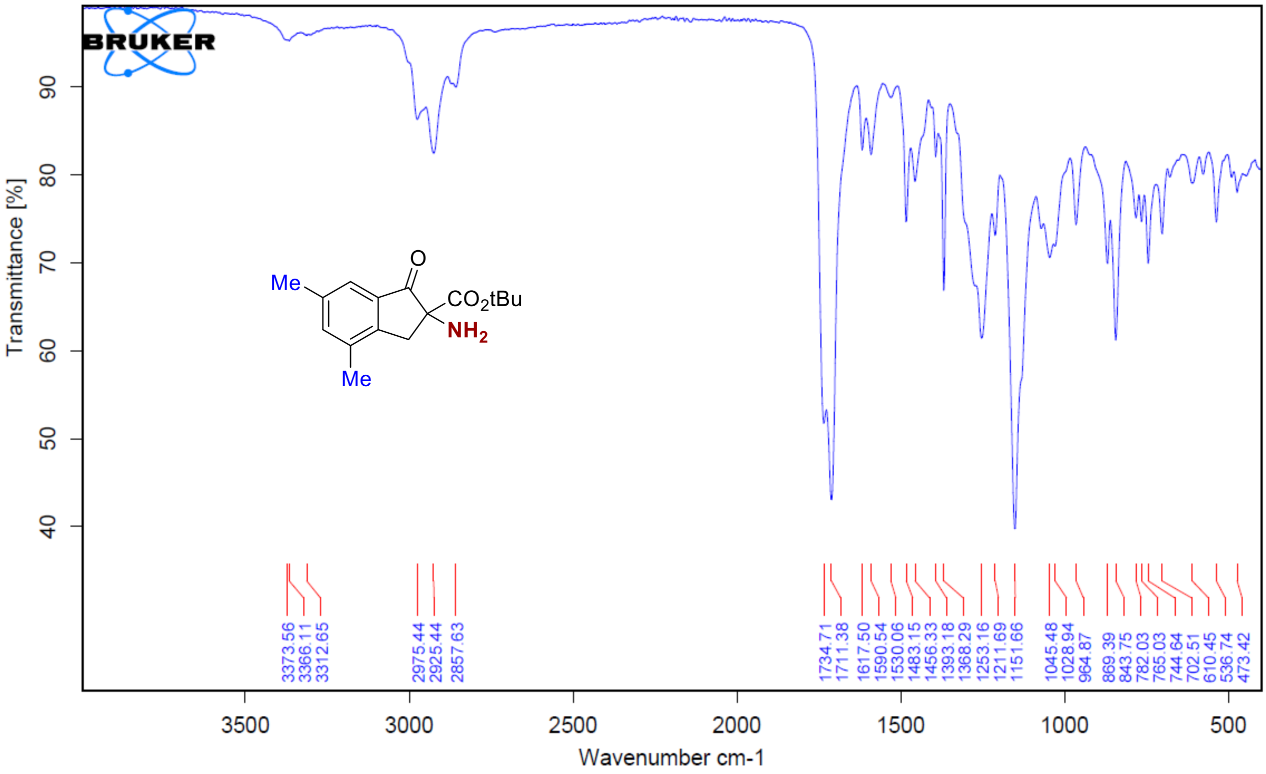

2m, ATR FT-IR (neat)

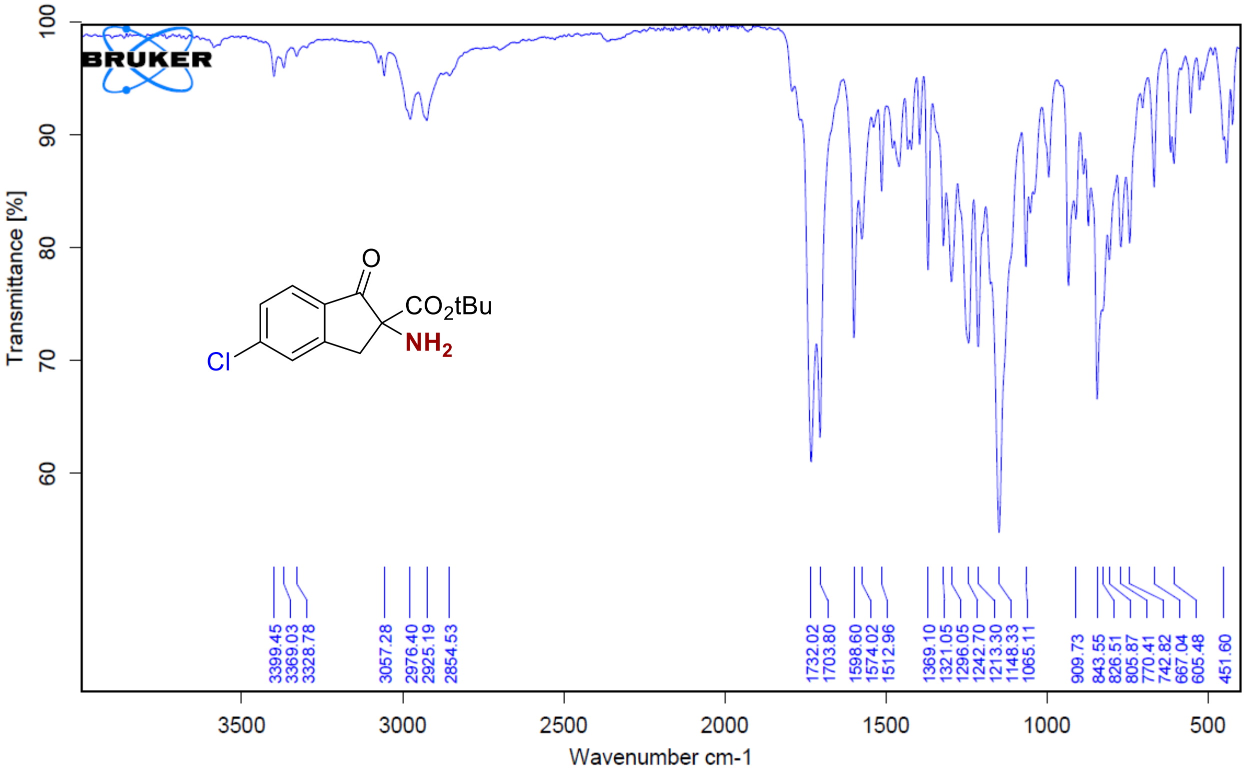

2n, ATR FT-IR (neat)

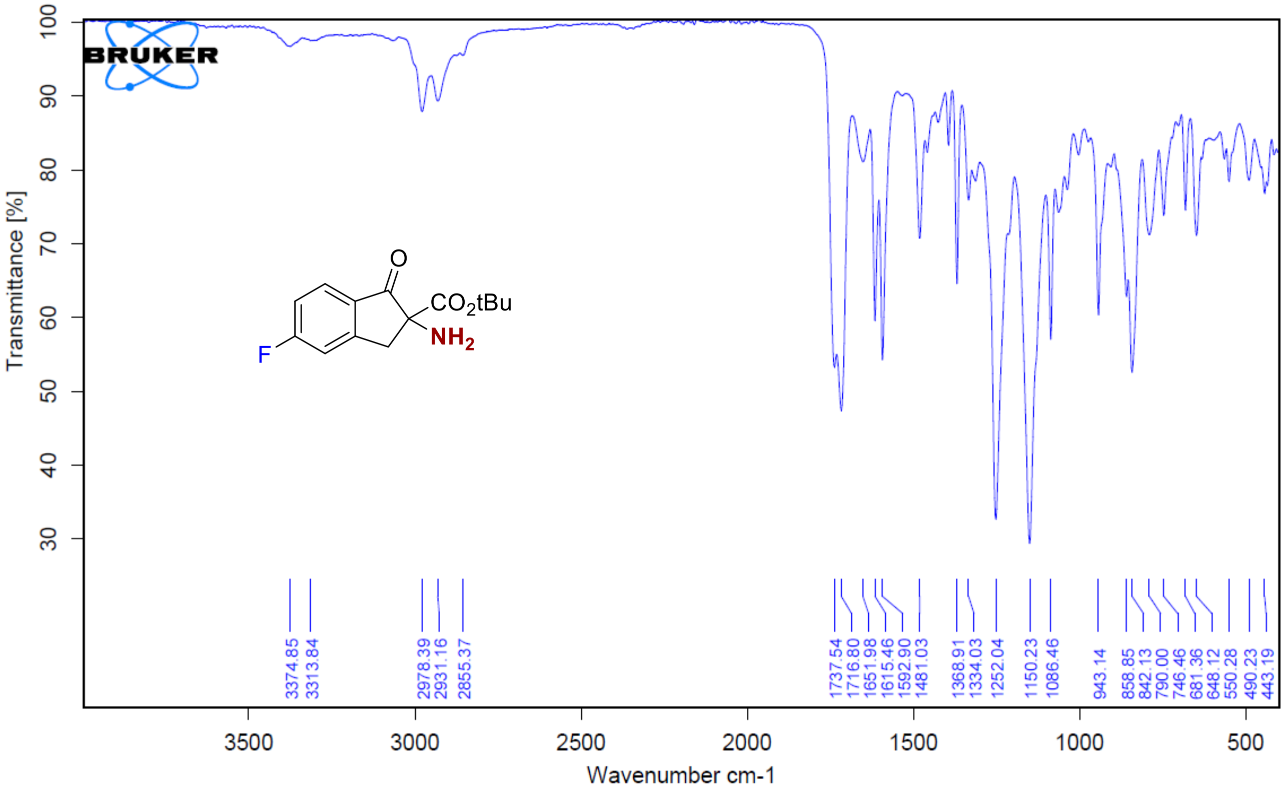

2o, ATR FT-IR (neat)

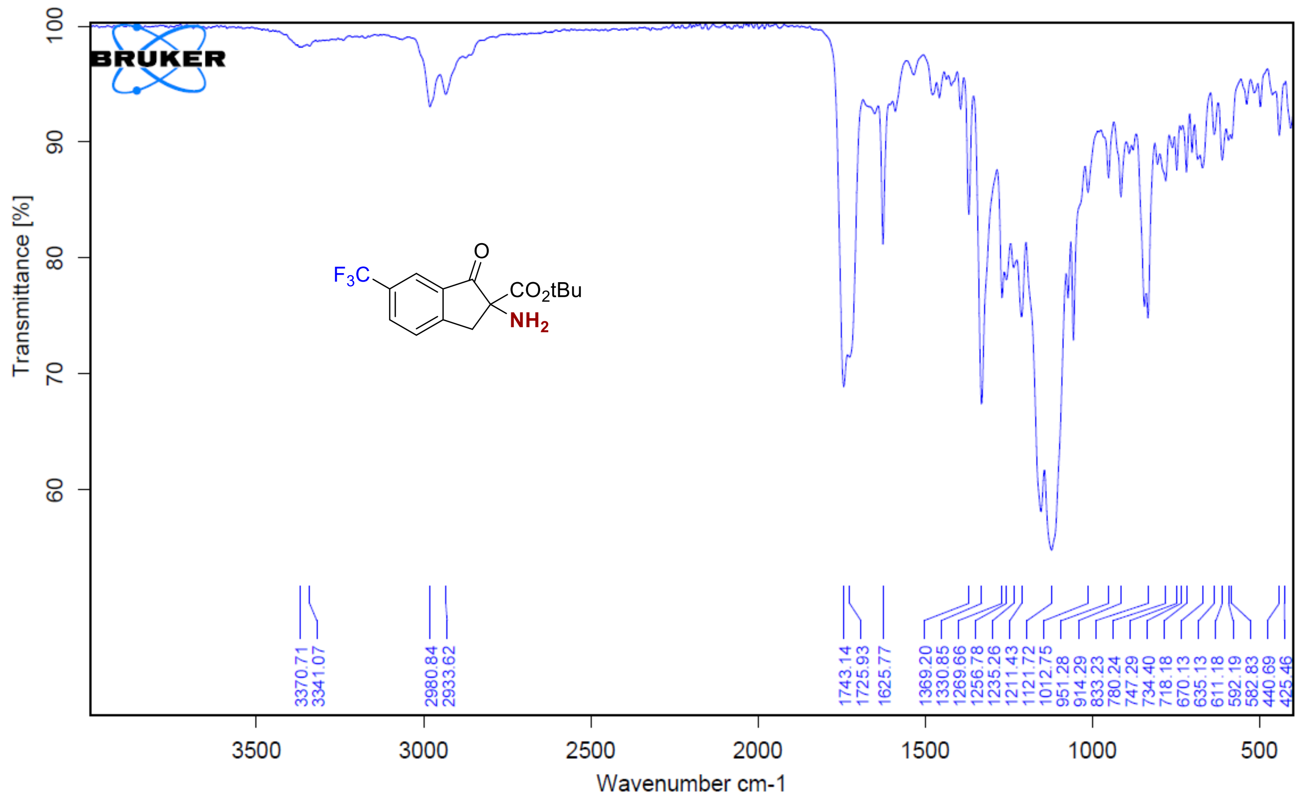

2p, ATR FT-IR (neat)

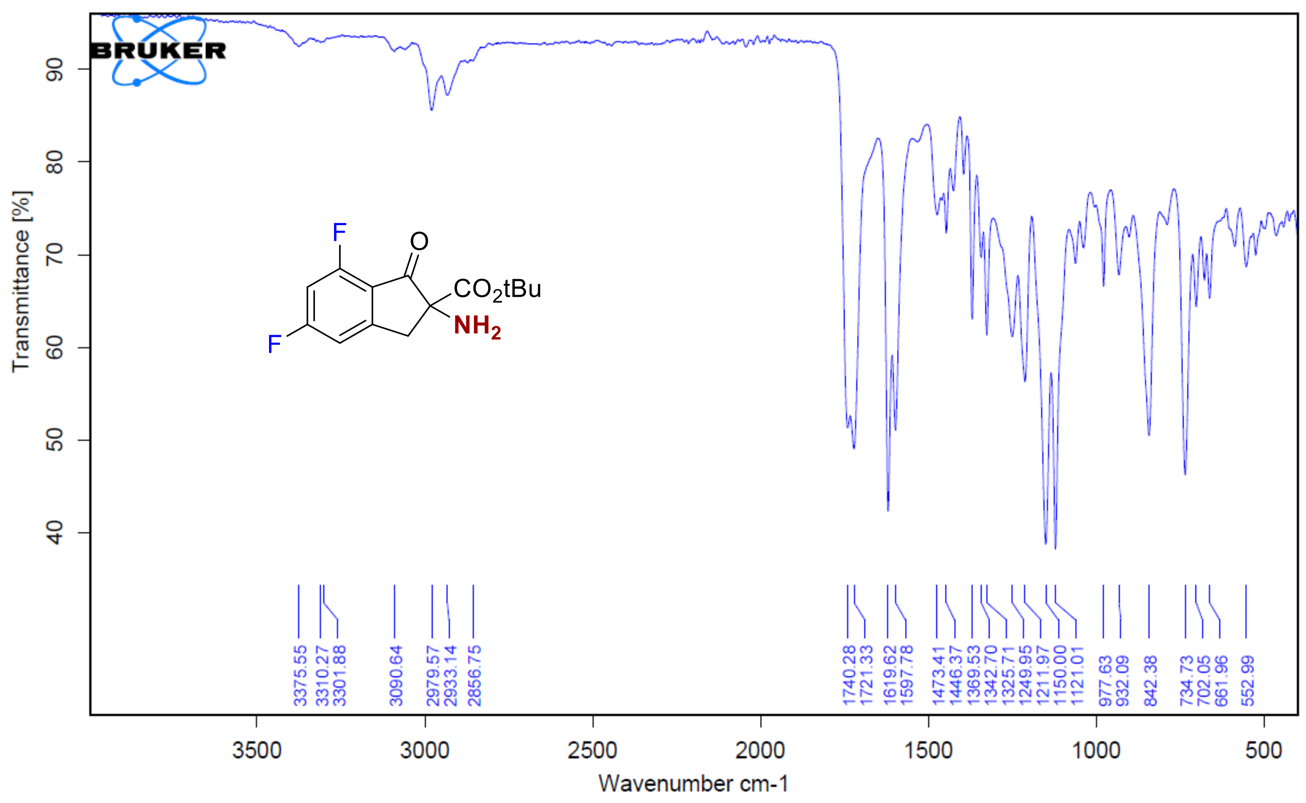

2q, ATR FT-IR (neat)

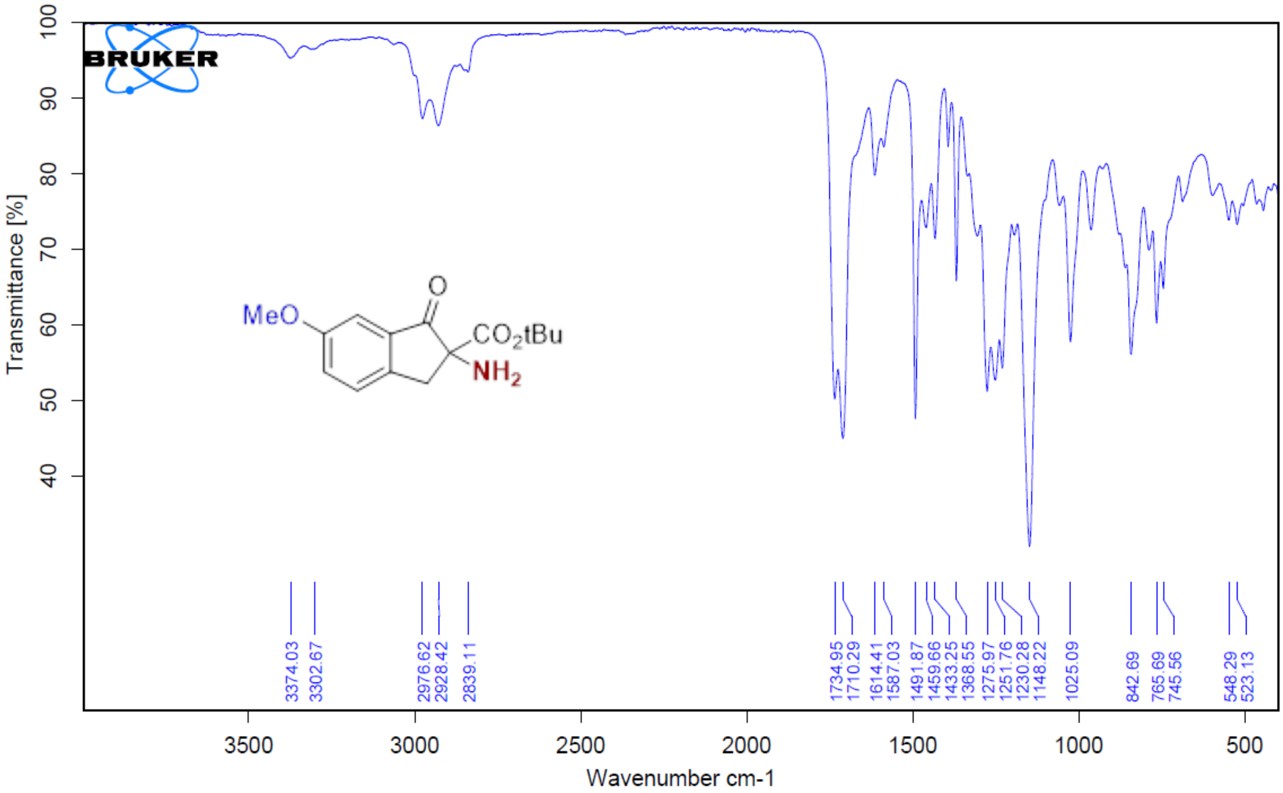

2r, ATR FT-IR (neat)

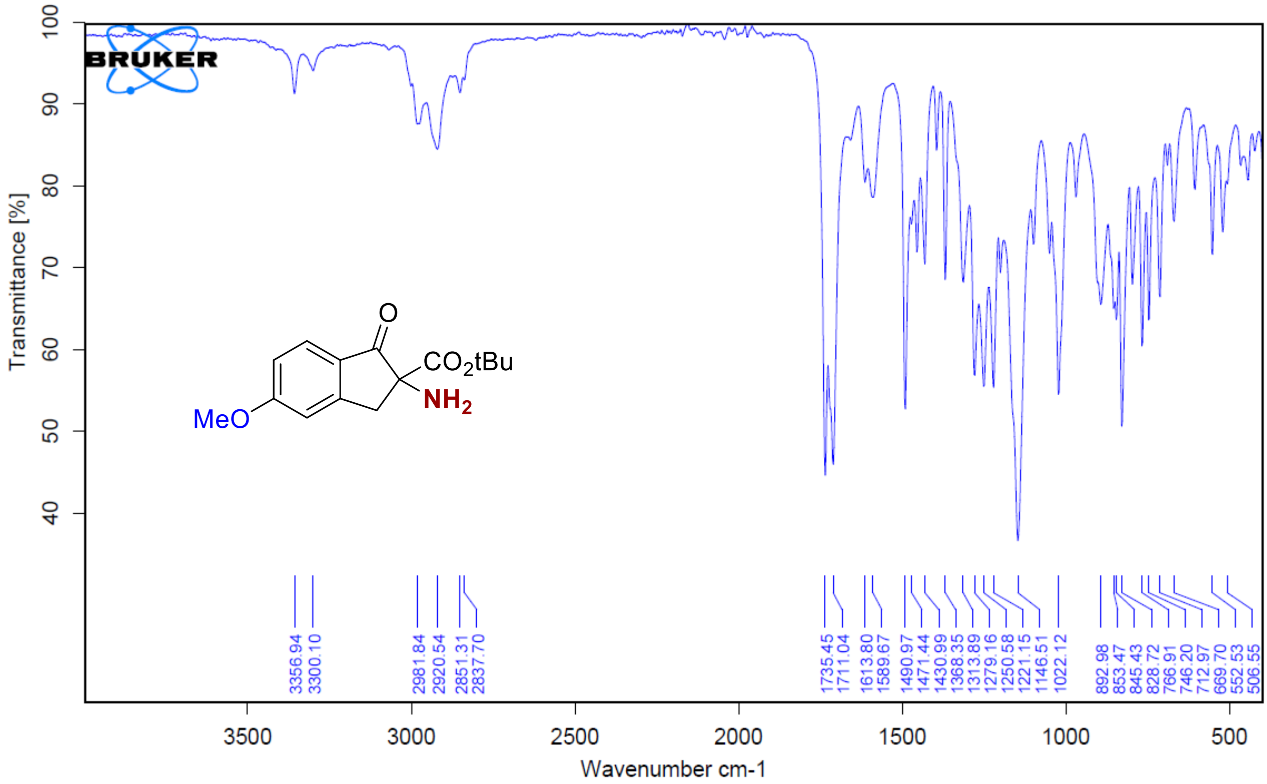

2s, ATR FT-IR (neat)

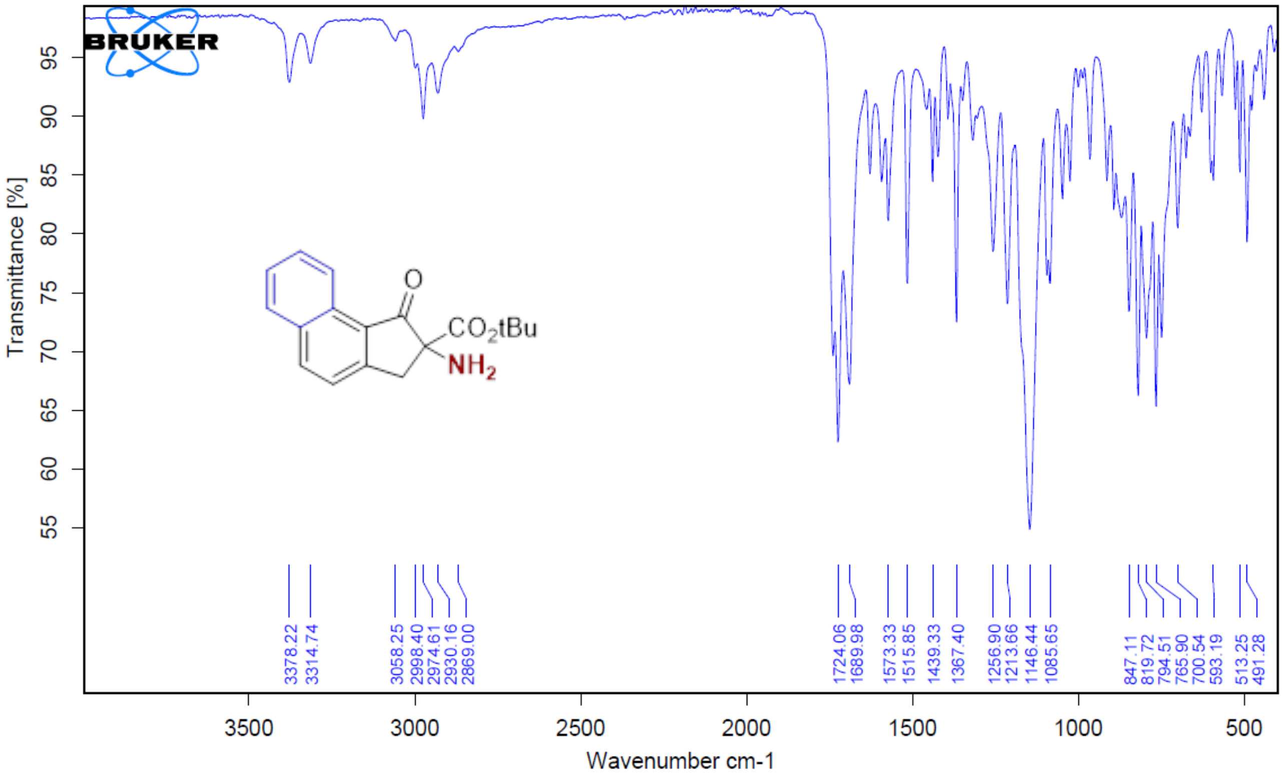

4a, ATR FT-IR (neat)

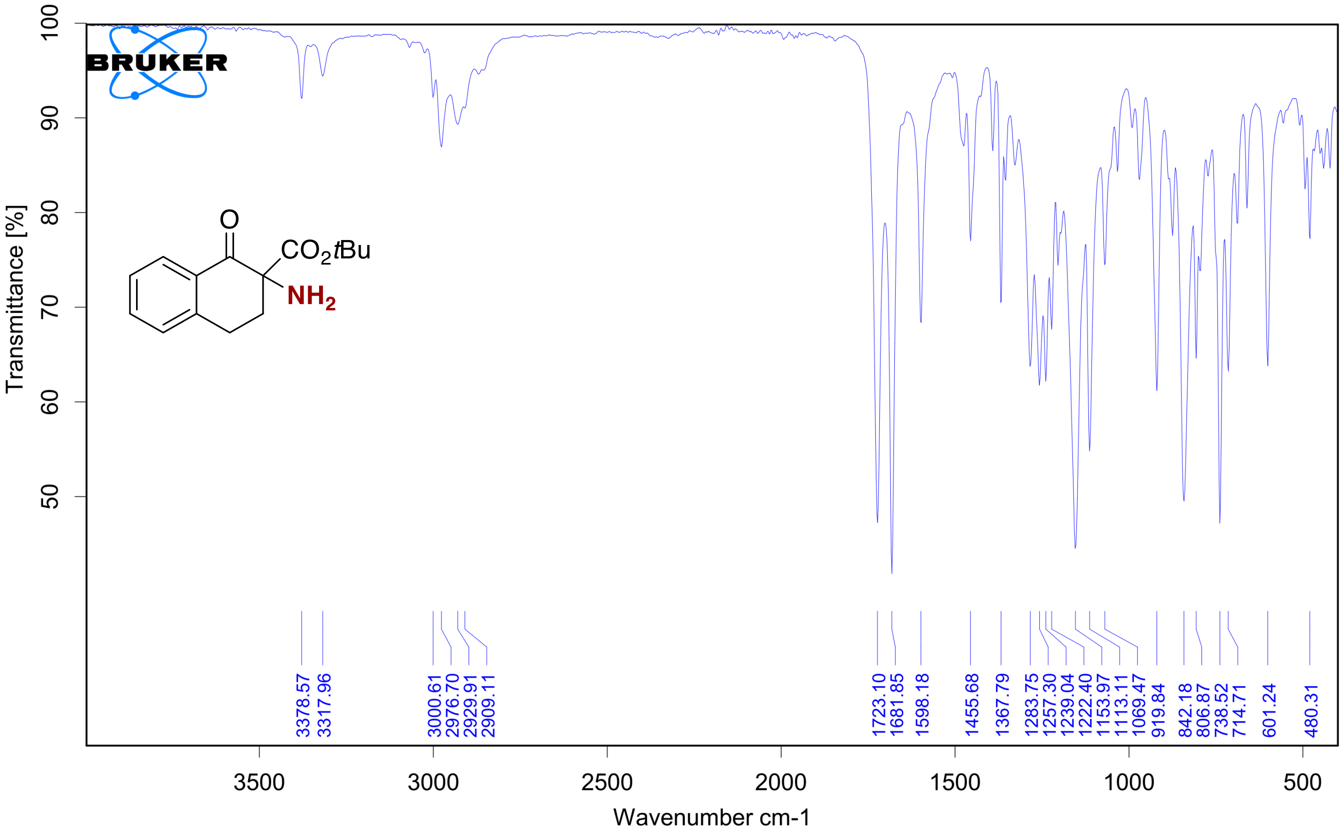

4b, ATR FT-IR (neat)

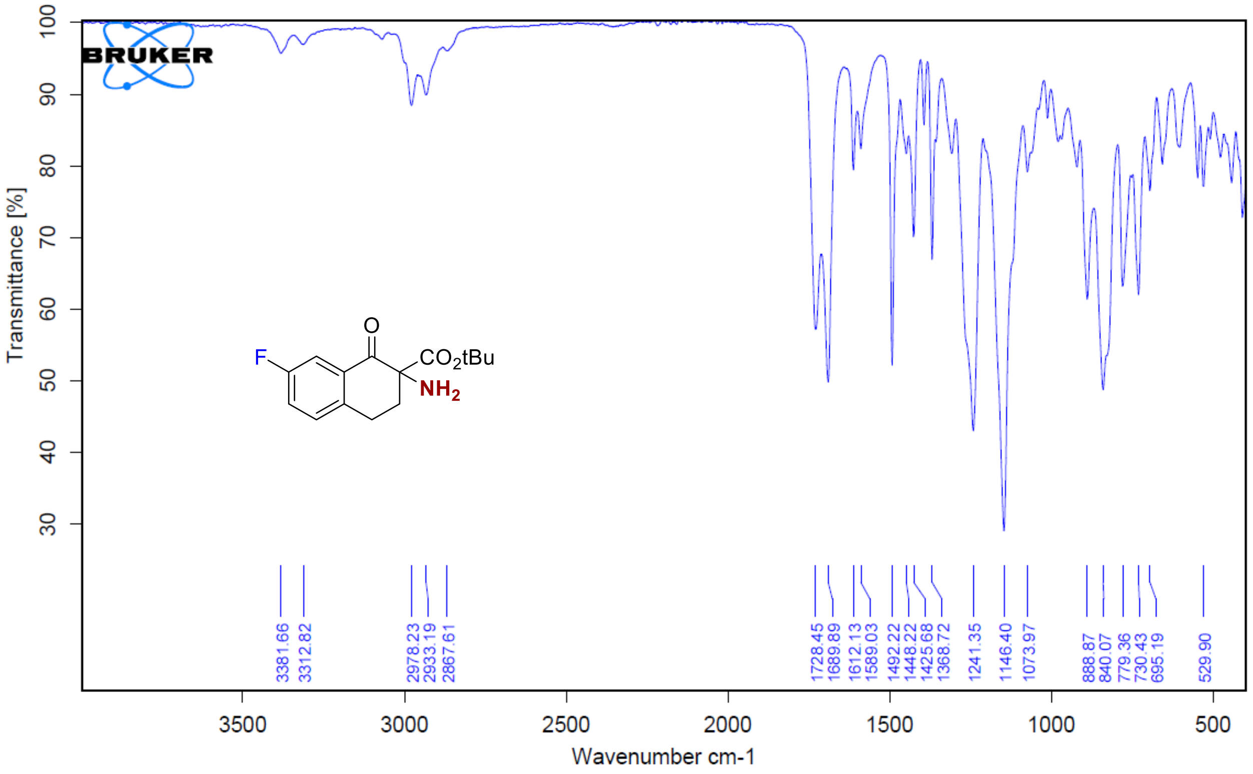

4c, ATR FT-IR (neat)

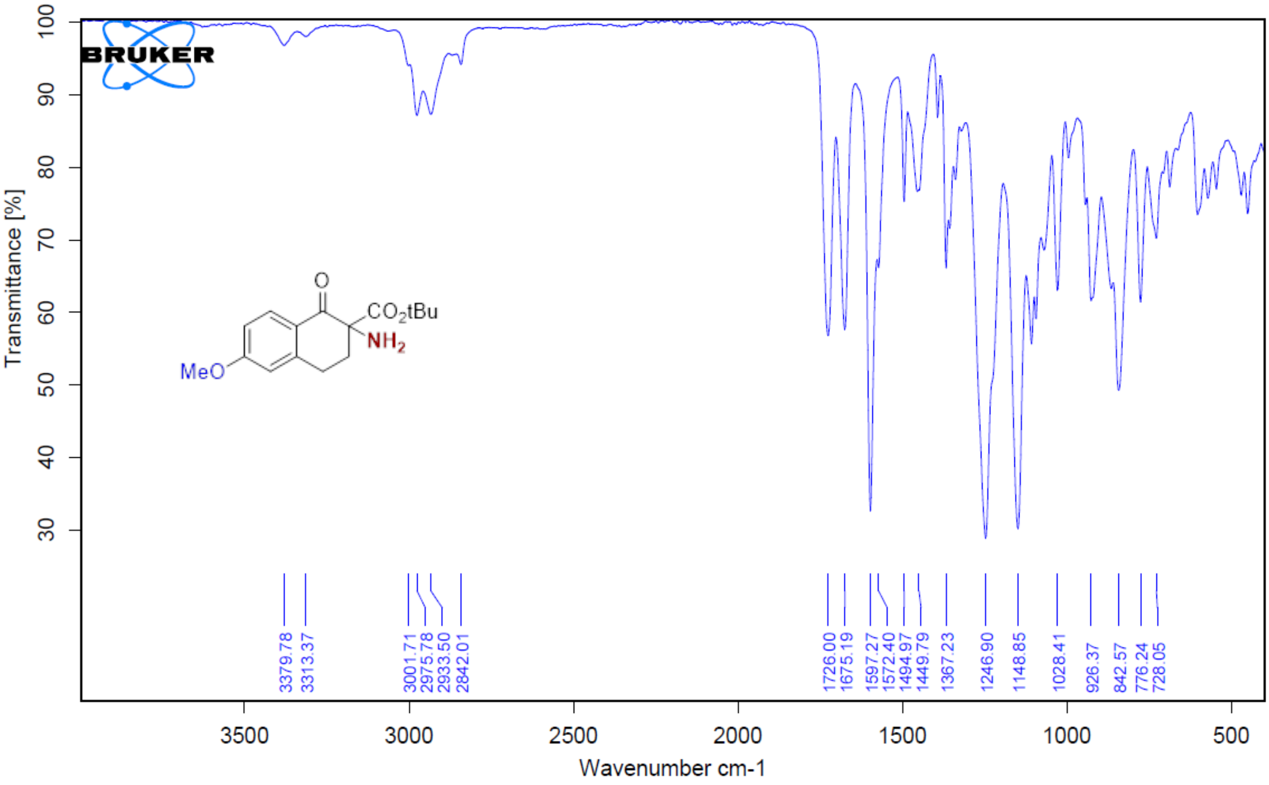

5a, ATR FT-IR (neat)

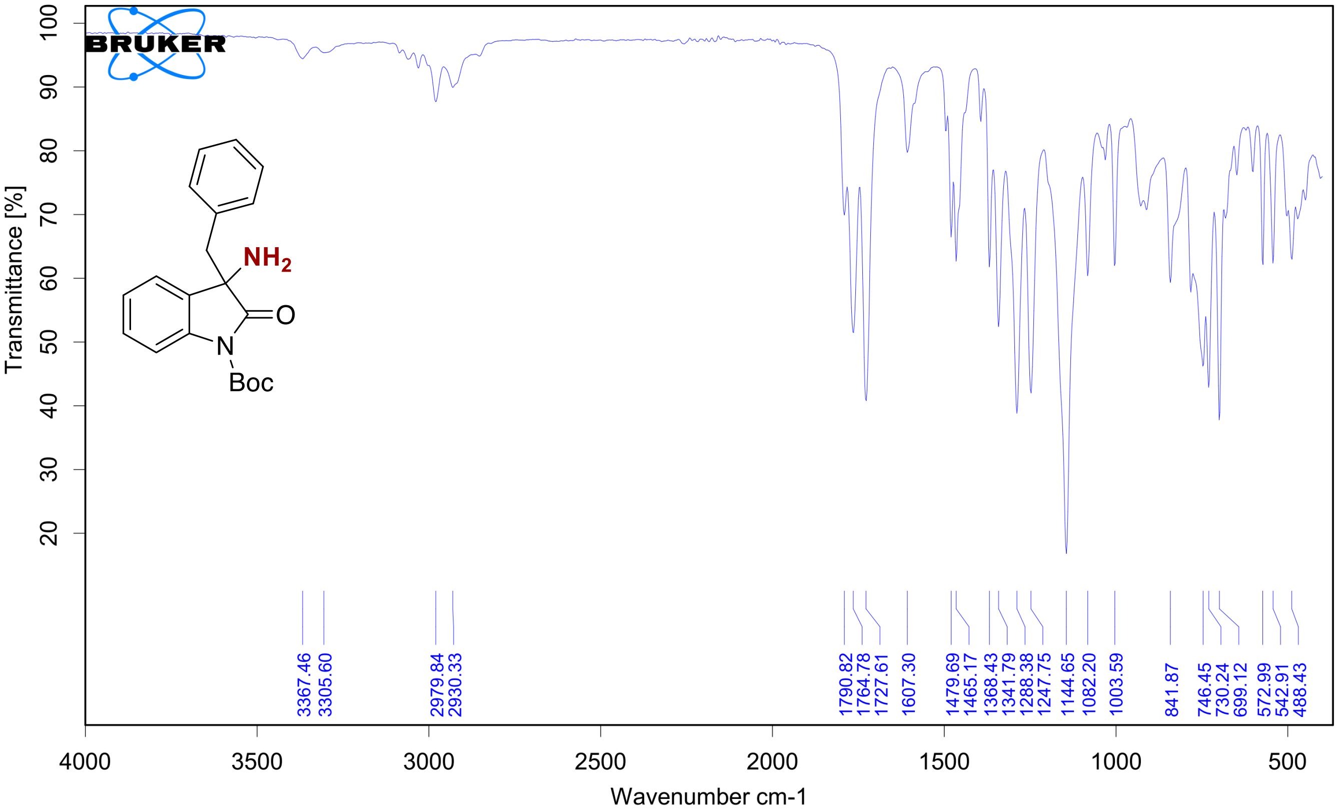

5b, ATR FT-IR (neat)

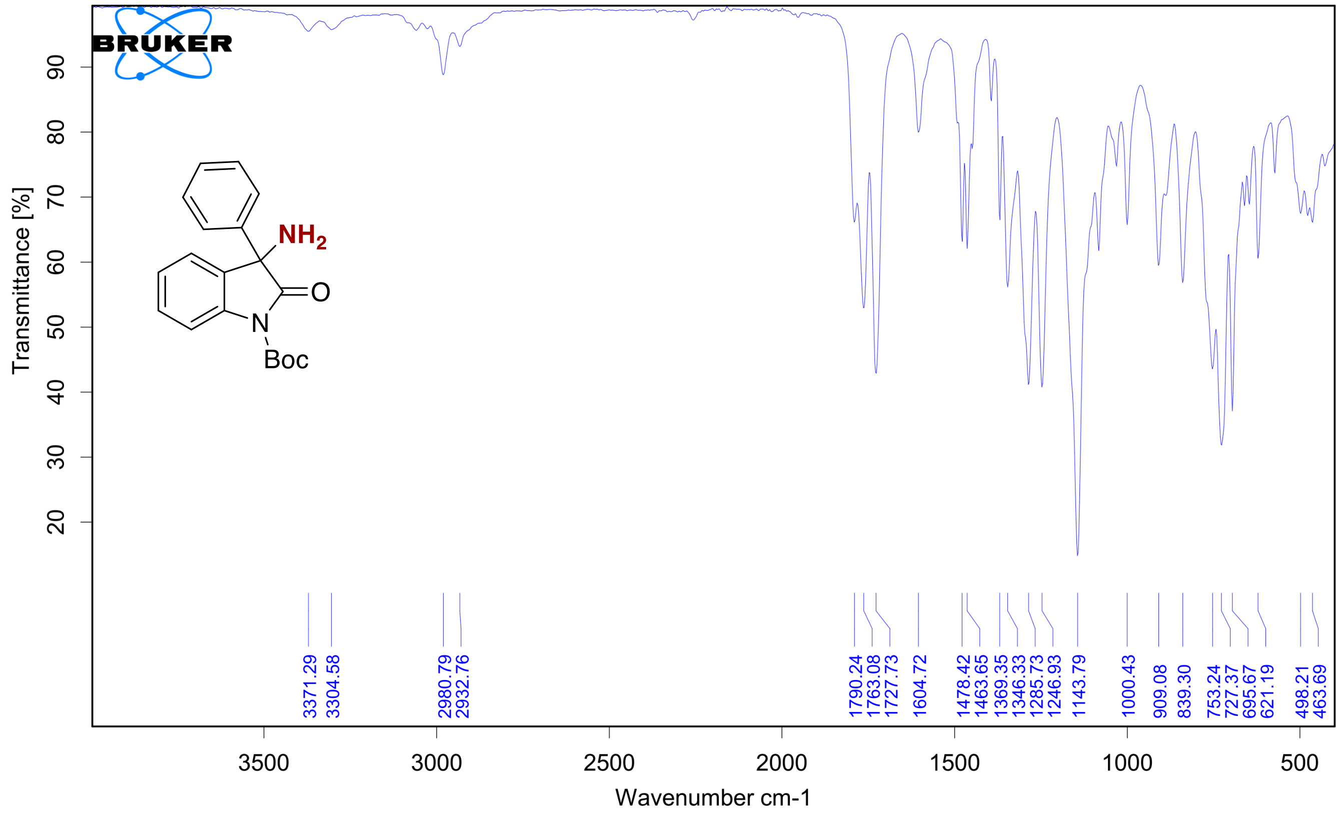

5c, ATR FT-IR (neat)

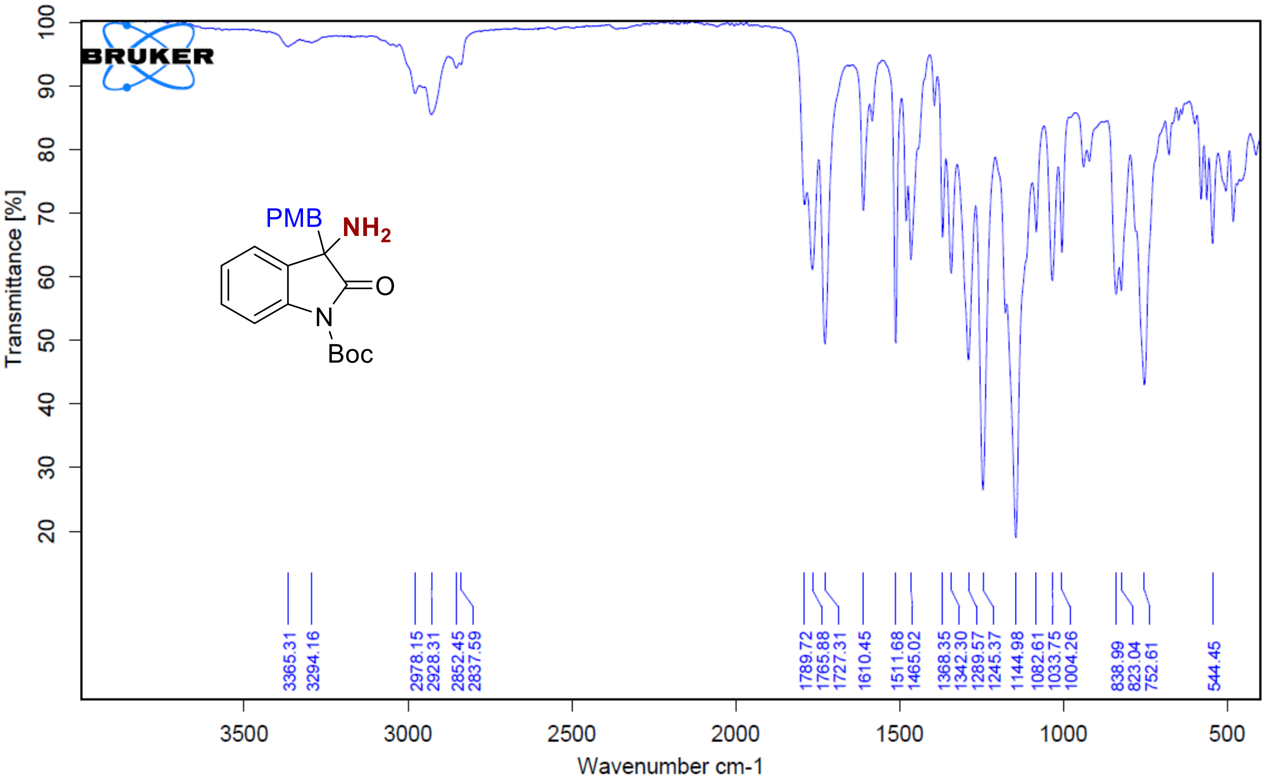

6a, ATR FT-IR (neat)

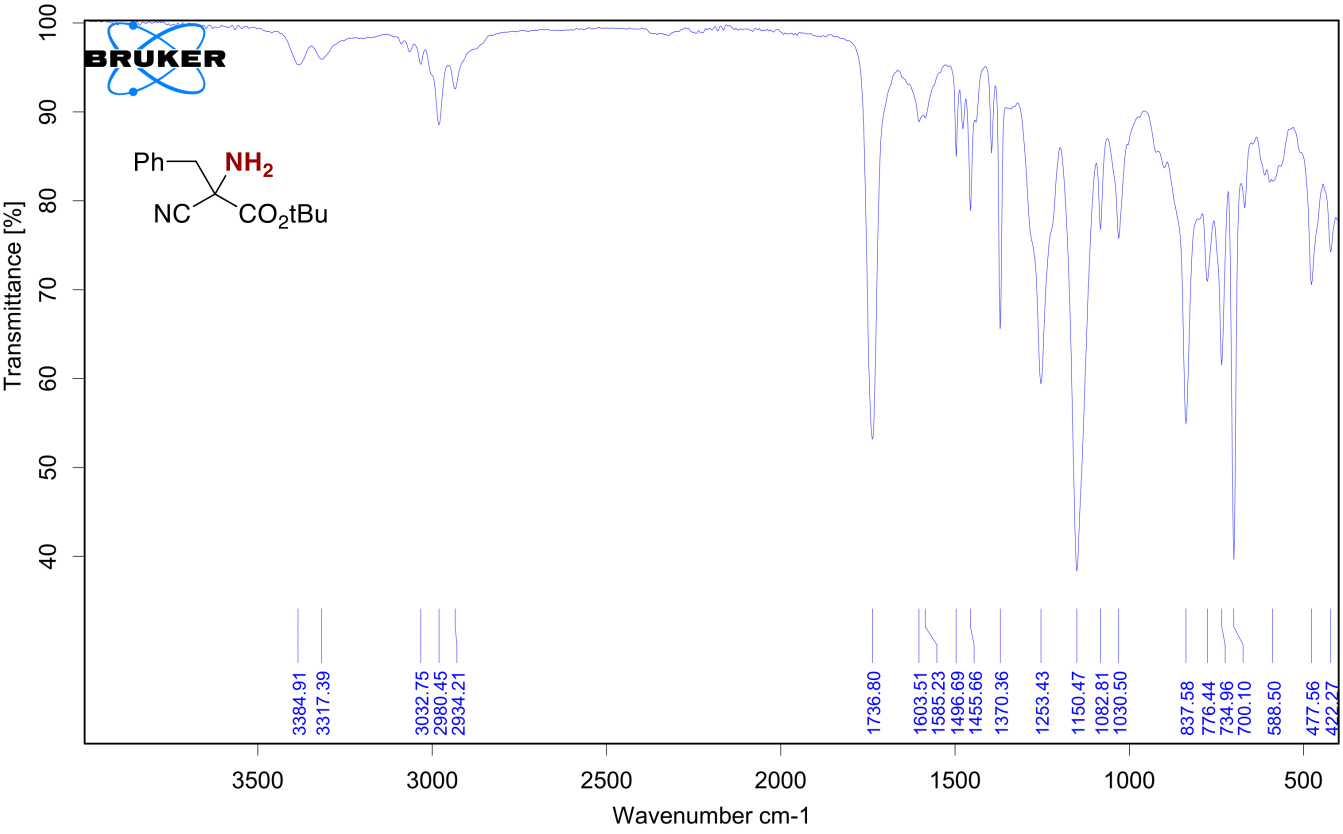

6b, ATR FT-IR (neat)

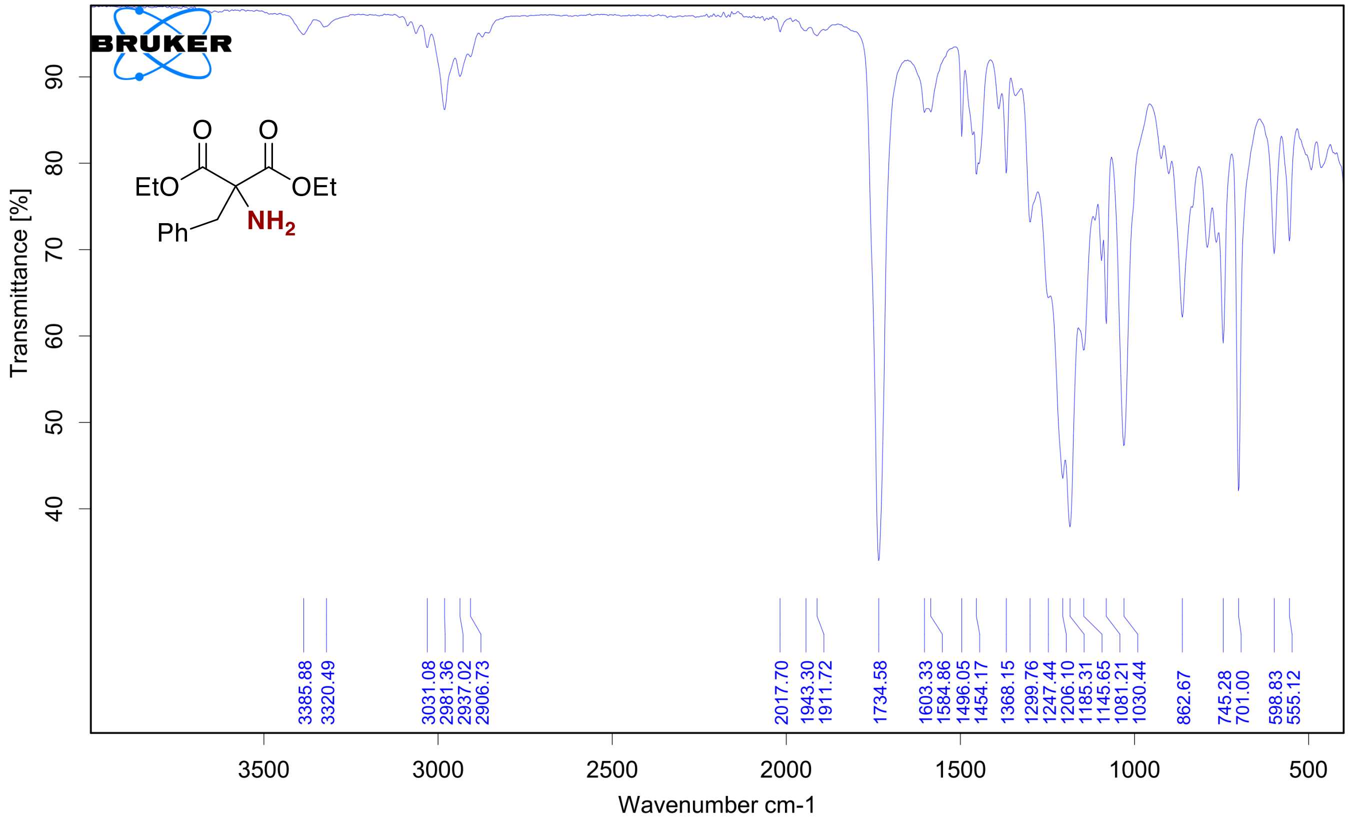

6c, ATR FT-IR (neat)

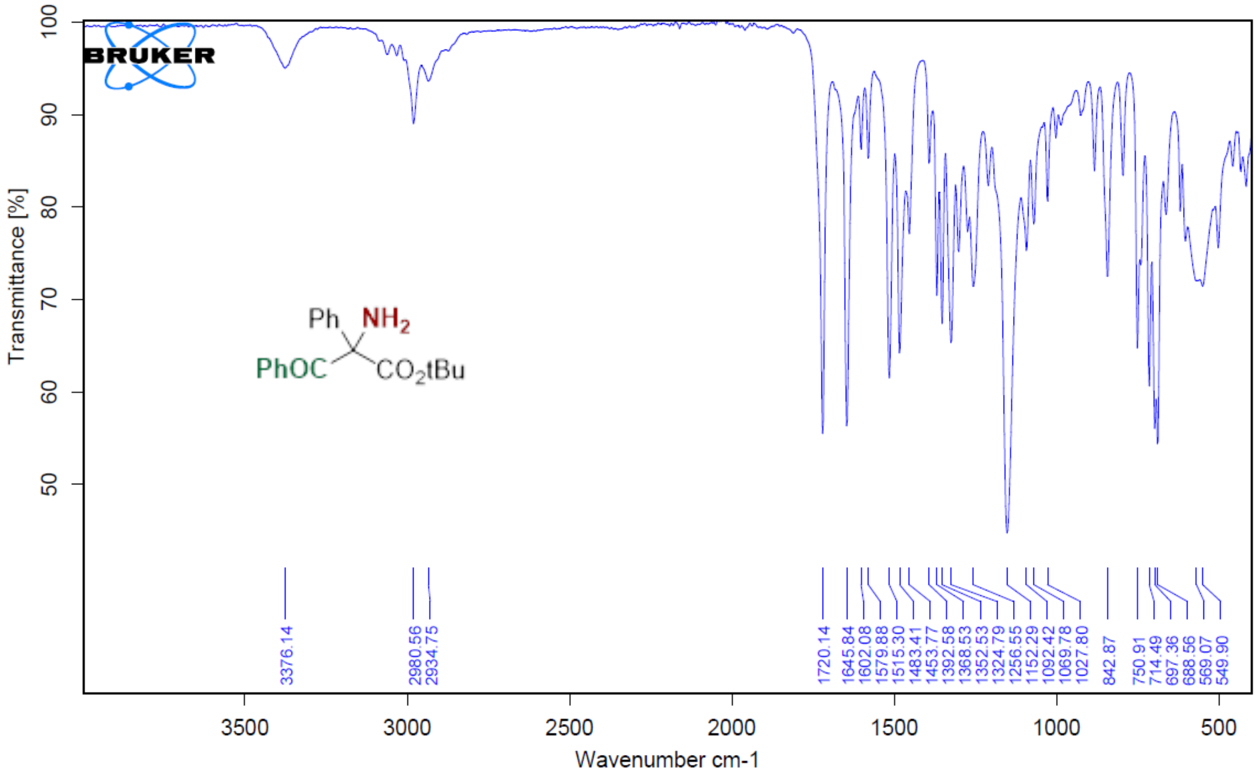

6. HRMS Data

2a, HRMS (ESI+) *m/z* calcd for $[C_{14}H_{17}NO_3+H]^+$ 248.1281, found 248.1281; also detected *m/z* calcd for $[C_{14}H_{17}NO_3+Na]^+$ 270.1101, found 270.1104 (+1.3 ppm).

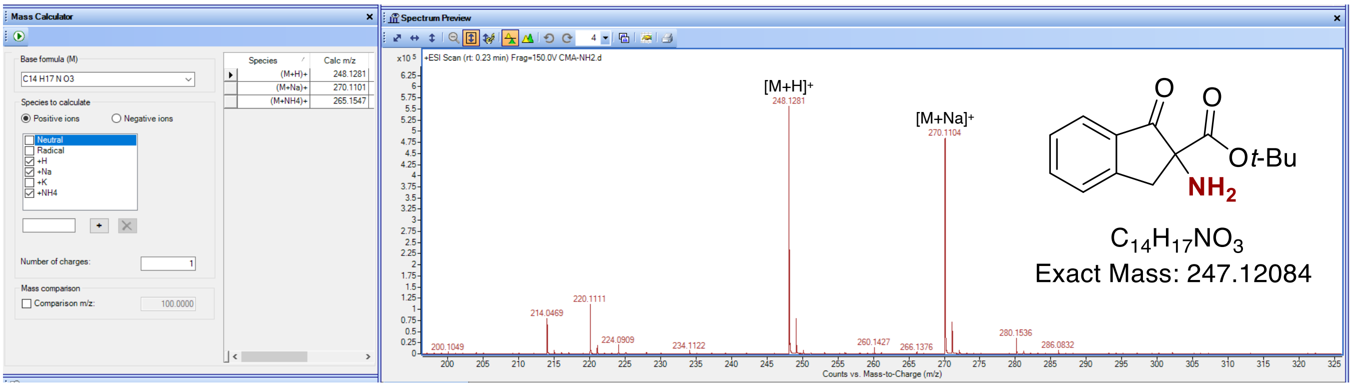

2b, HRMS (ESI+) *m/z* calcd for $[C_{17}H_{15}NO_3+H]^+$ 282.1125, found 282.1124 (−0.2 ppm).

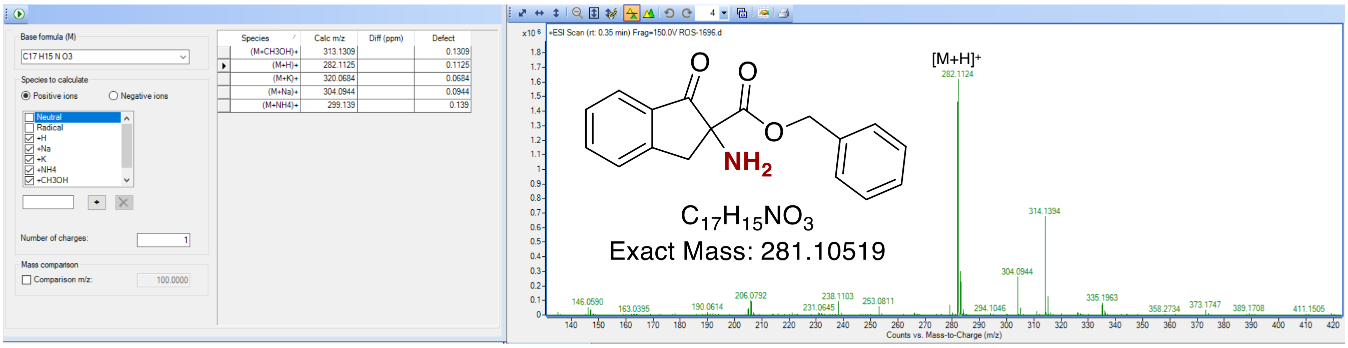

2c, HRMS (ESI+) *m/z* calcd for $[C_{13}H_{13}NO_3+H]^+$ 232.0968, found 232.0969 (+0.4 ppm).

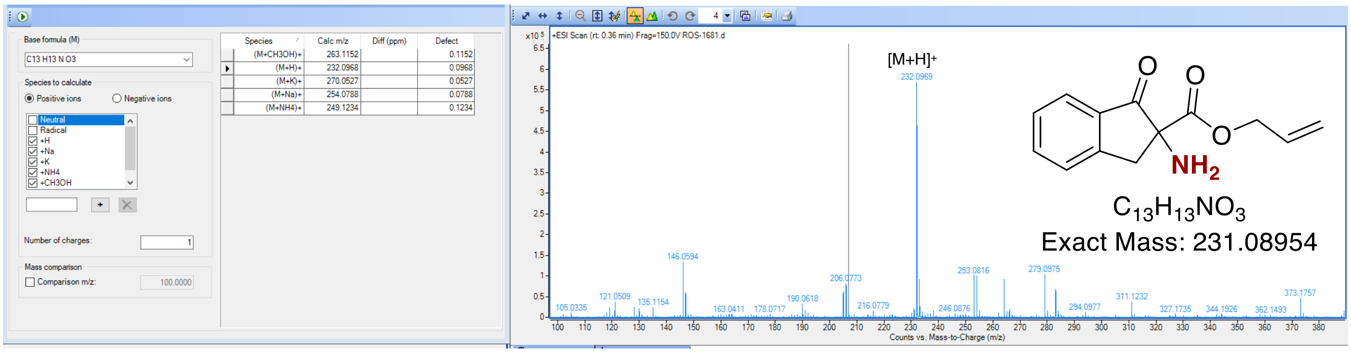

2d, HRMS (ESI+) *m/z* calcd for $[C_{20}H_{23}NO_3+H]^+$ 326.1751, found 326.1750 (−0.3 ppm).

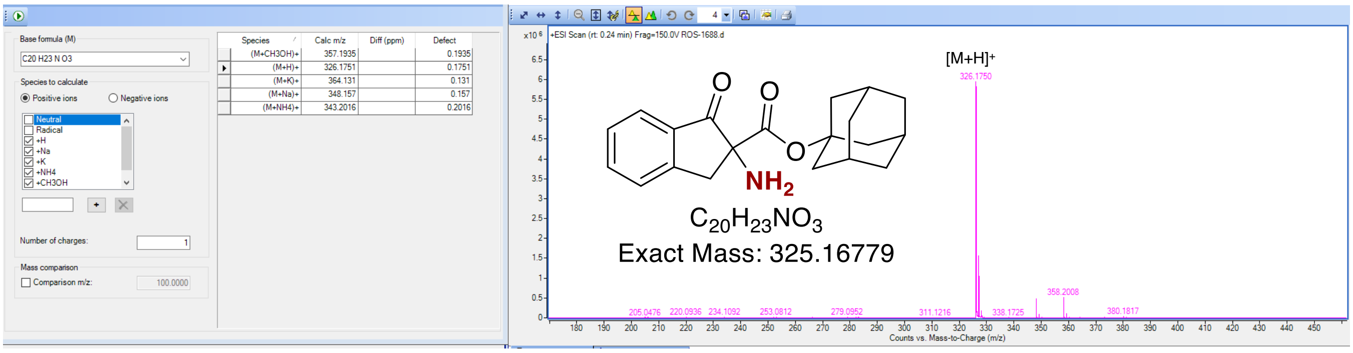

2e, HRMS (ESI+) *m/z* calcd for $[C_{19}H_{19}NO_3+Na]^+$ 332.1257, found 332.1255 (−0.6 ppm).

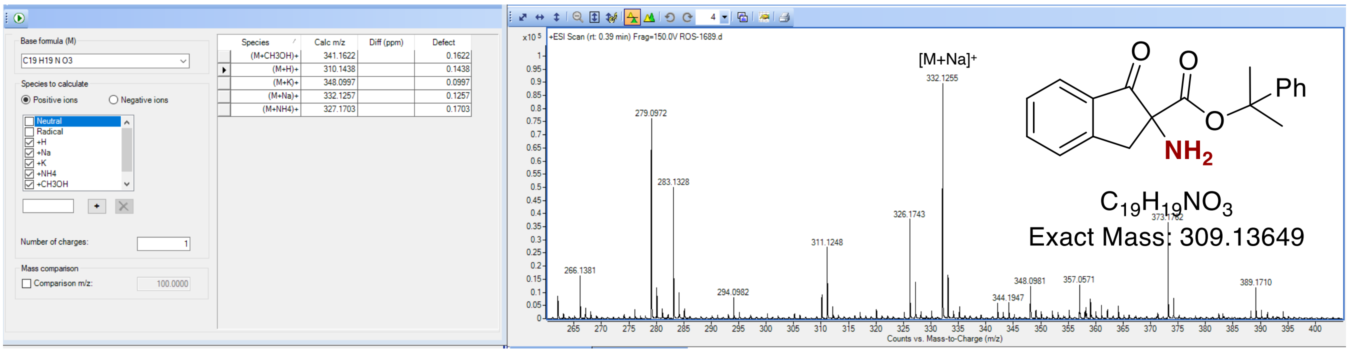

2f, HRMS (ESI+) *m/z* calcd for $[C_{14}H_{16}N_2O_3+H]^+$ 261.1234, found 261.1235 (+0.4 ppm).

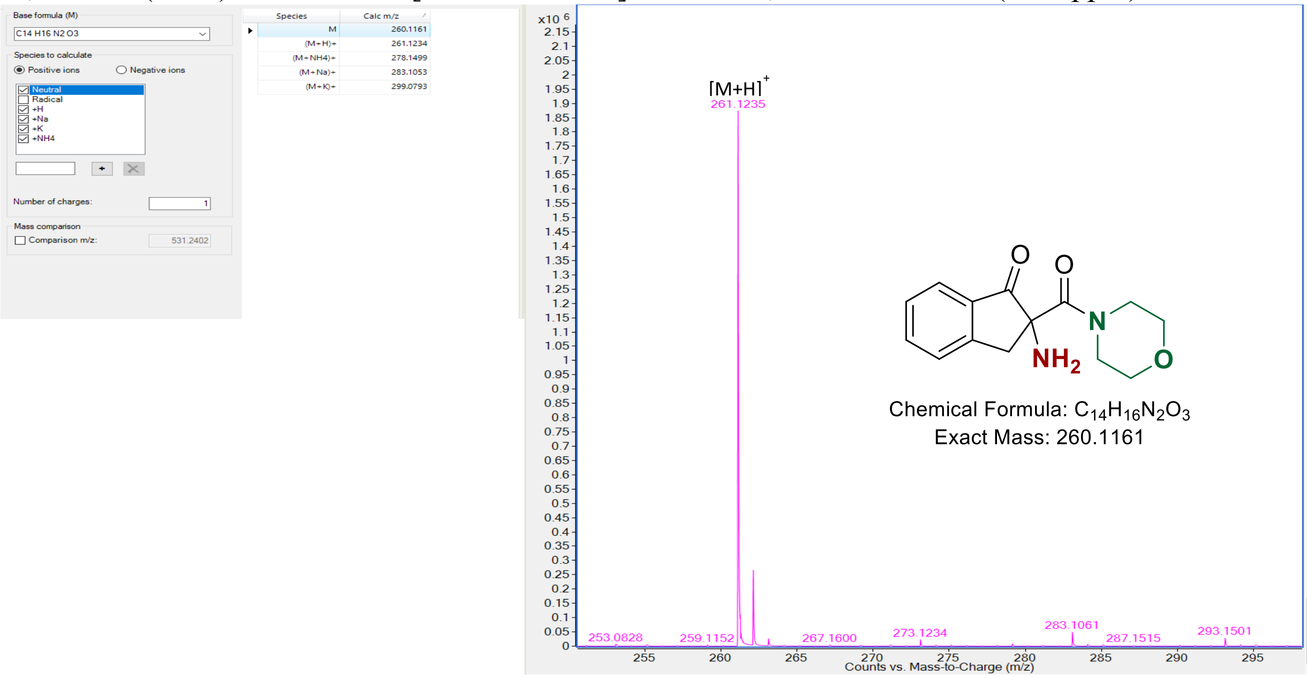

2g, HRMS (ESI+) *m/z* calcd for $[C_{14}H_{18}N_2O_2+H]^+$ 247.1441, found 247.1434 (−2.8 ppm).

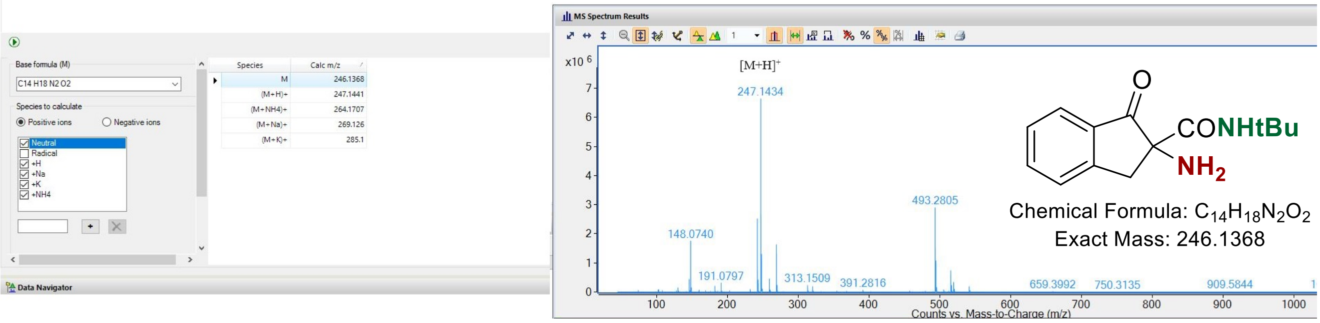

2h, HRMS (ESI+) *m/z* calcd for $[C_{18}H_{19}NO_3+Na]^+$ 284.1257, found 284.1243 (−4.9 ppm).

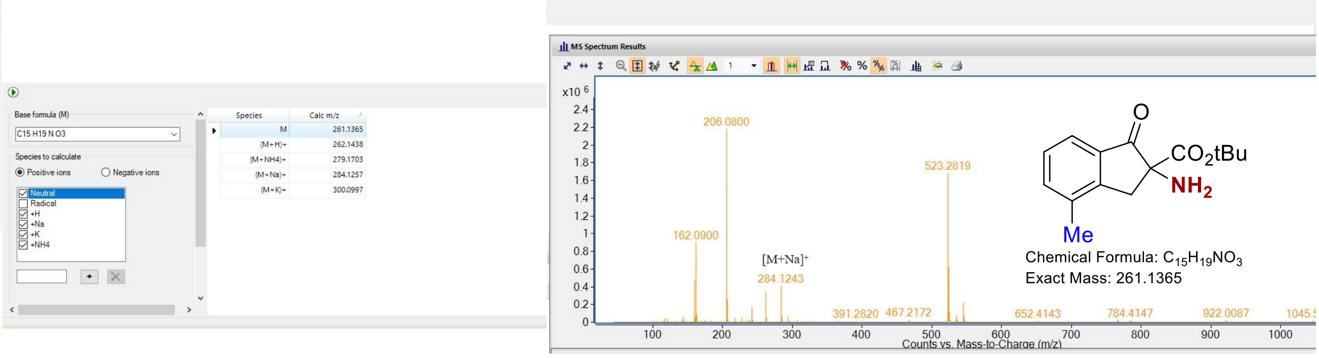

2i, H RMS (ESI+) *m/z* calcd for $[C_{15}H_{19}NO_3+H]^+$ 262.1438, found 262.1440 (+0.7 ppm).

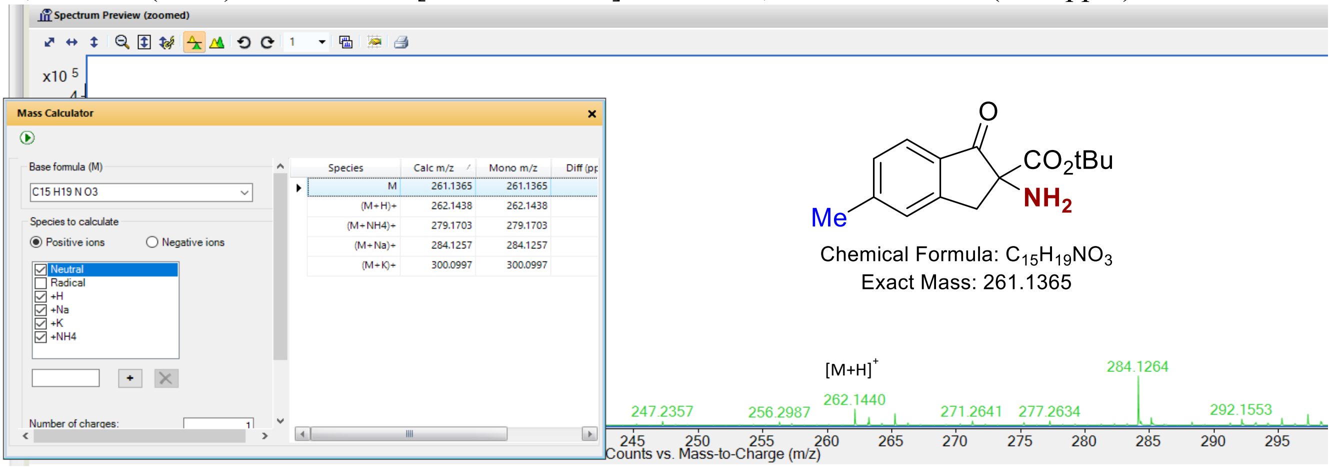

2j, HRMS (ESI+) *m/z* calcd for $[C_{15}H_{19}NO_3+H]^+$ 262.1438, found 262.1435 (-1.1 ppm).

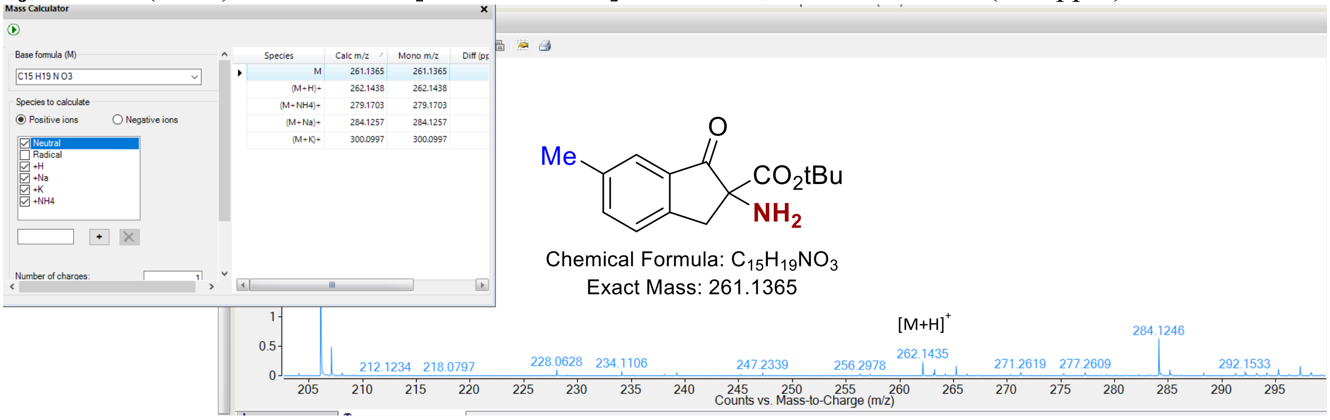

2k, HRMS (ESI+) *m/z* calcd for $[C_{16}H_{21}NO_3+H]^+$ 276.1594, found 276.1593 (-0.4 ppm).

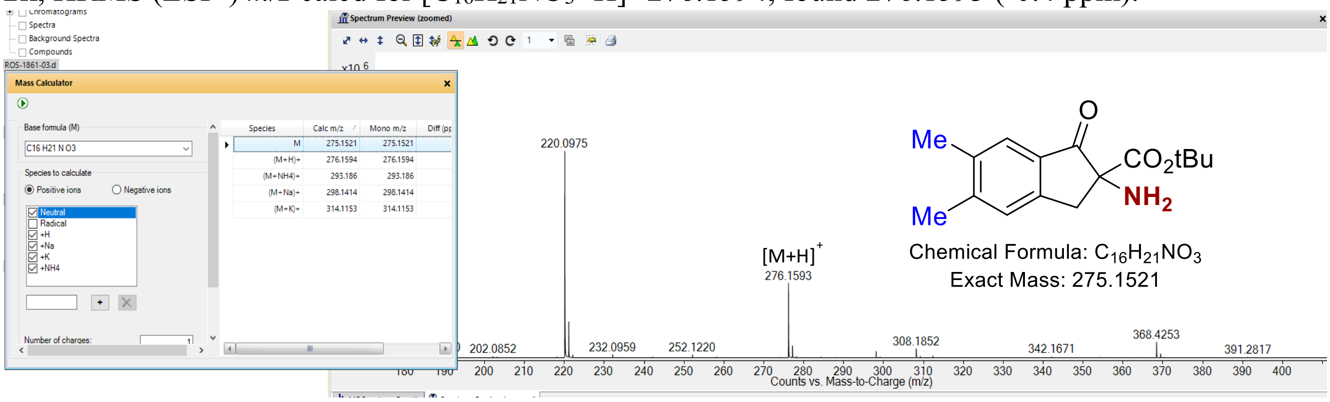

2l, HRMS (ESI+) *m/z* calcd for [$C_{16}H_{21}NO_3$+H]$^+$ 276.1594, found 276.1595 (+0.4 ppm).

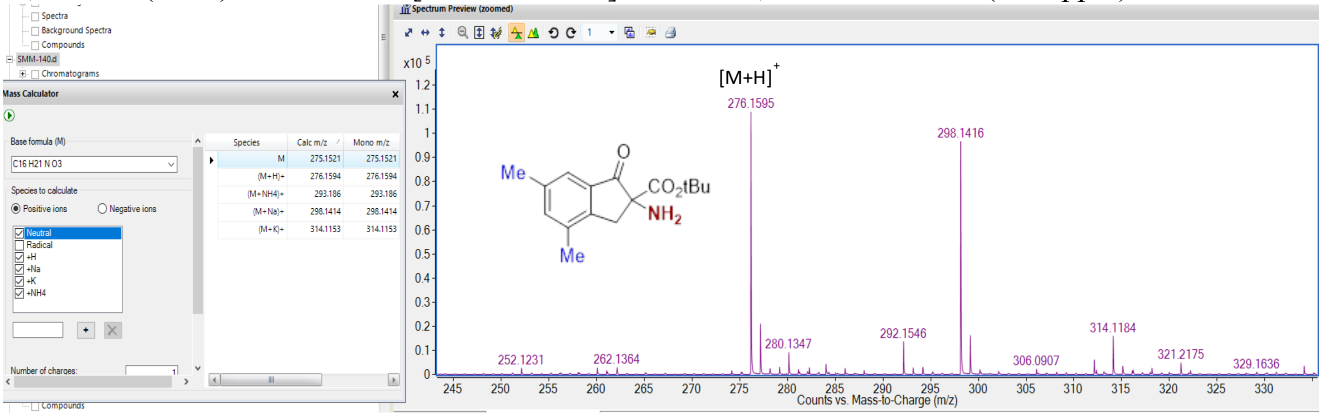

2m, HRMS (ESI+) *m/z* calcd for [$C_{14}H_{16}ClNO_3$+Na]$^+$ 304.0711, found 304.0707 (−1.3 ppm).

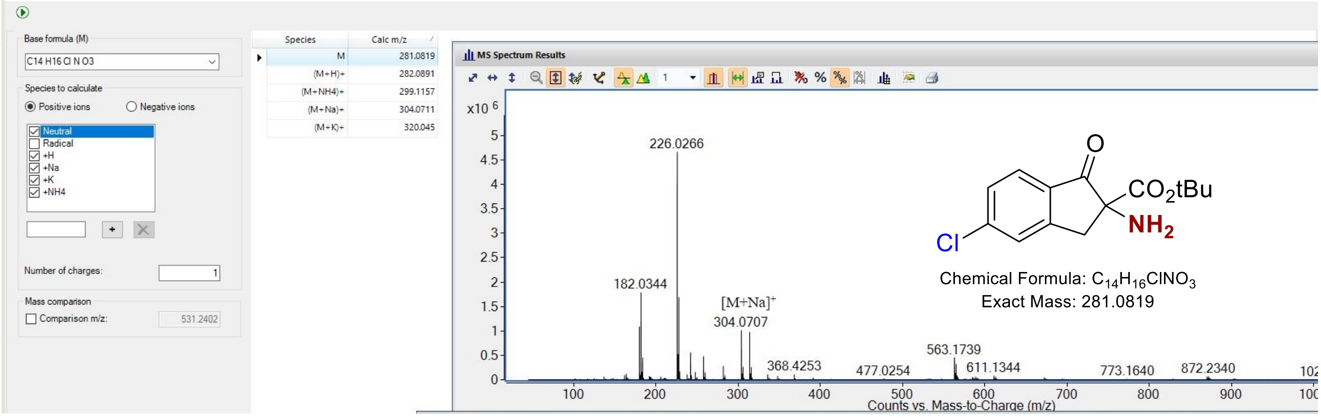

2n, HRMS (ESI+) *m/z* calcd for [$C_{14}H_{16}FNO_3$+H]$^+$ 266.1187, found 266.1191 (−1.5 ppm).

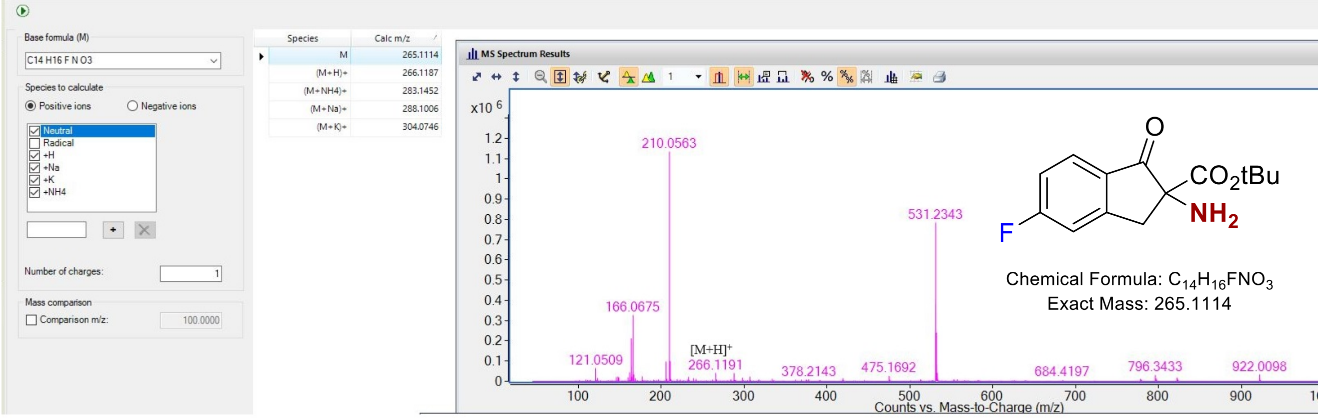

2o, HRMS (ESI+) *m/z* calcd for [$C_{15}H_{16}F_3NO_3$+H]$^+$ 316.1155, found 316.1155 (+/- 0 ppm).

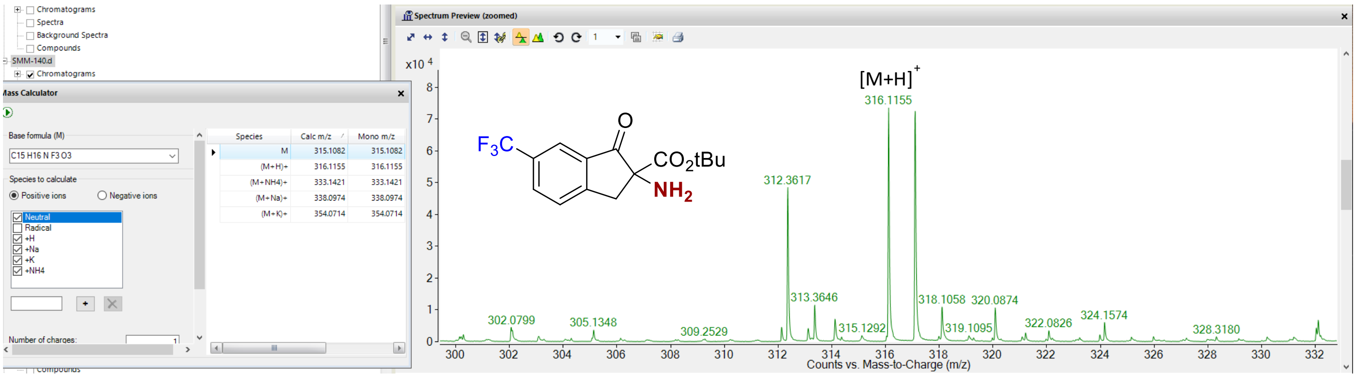

2p, HRMS (ESI+) *m/z* calcd for $[C_{14}H_{15}NF_2O_3+H]^+$ 284.1093, found 284.1094 (+0.3 ppm).

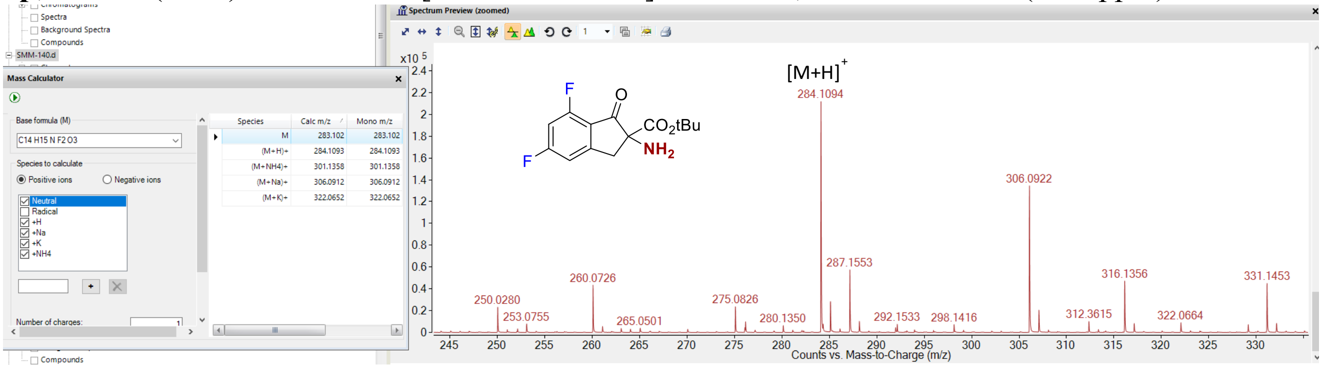

2q, HRMS (ESI+) *m/z* calcd for $[C_{15}H_{19}NO_4+Na]^+$ 300.1206, found 300.1197 (−3.0 ppm).

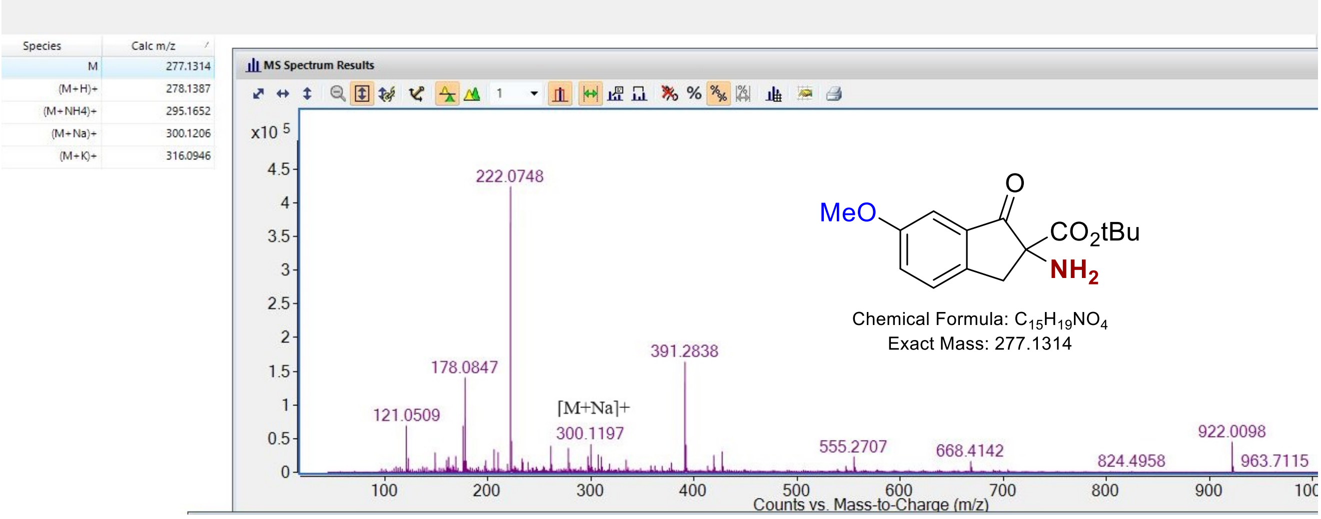

2r, HRMS (ESI+) *m/z* calcd for $[C_{15}H_{19}NO_4+H]^+$ 278.1387, found 278.1384 (−1.1 ppm).

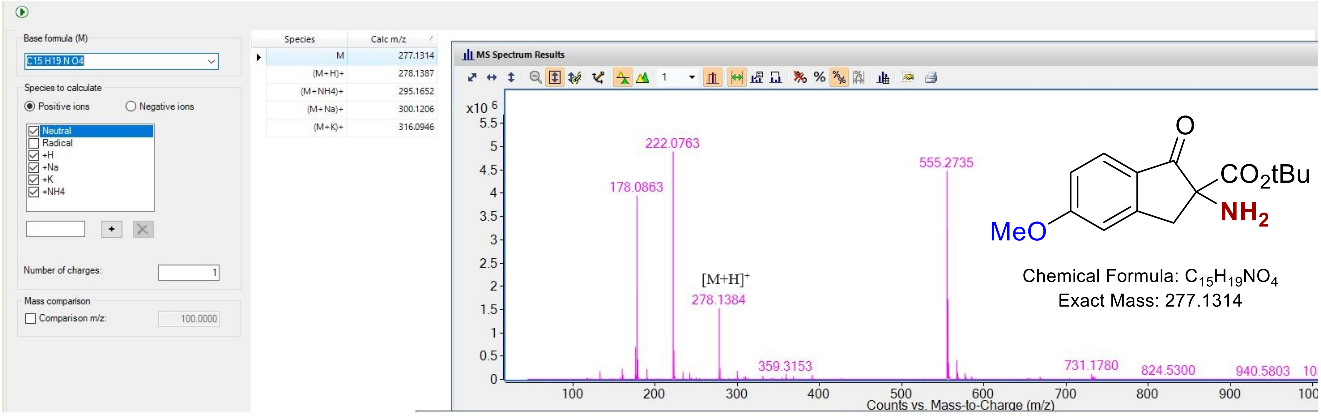

2s, HRMS (ESI+) *m/z* calcd for $[C_{18}H_{19}NO_3+H]^+$ 298.1438, found 298.1435 (-1.0 ppm).

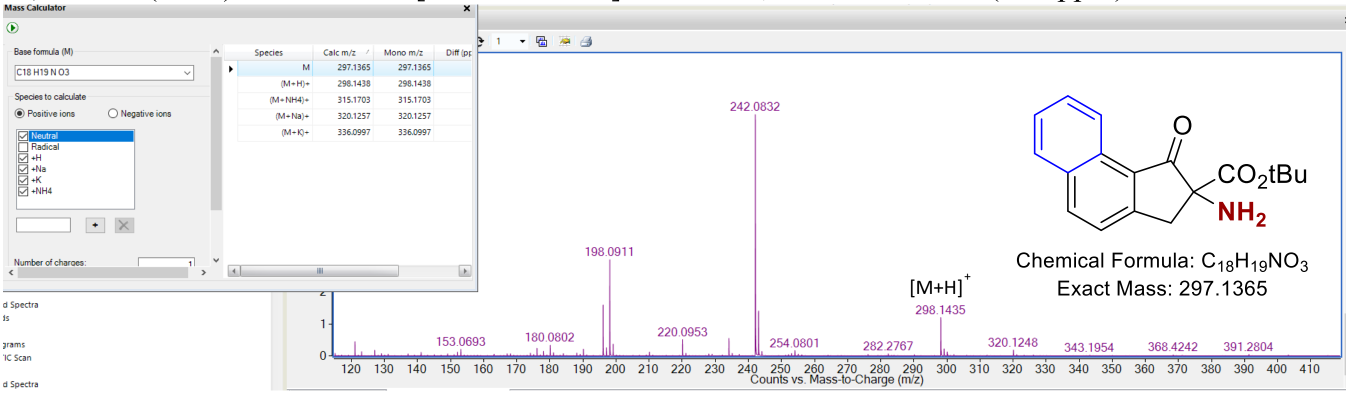

4a, HRMS (ESI+) m/z calcd for $[C_{15}H_{19}NO_3+H]^+$ 262.1438, found 262.1437 (−0.3 ppm).

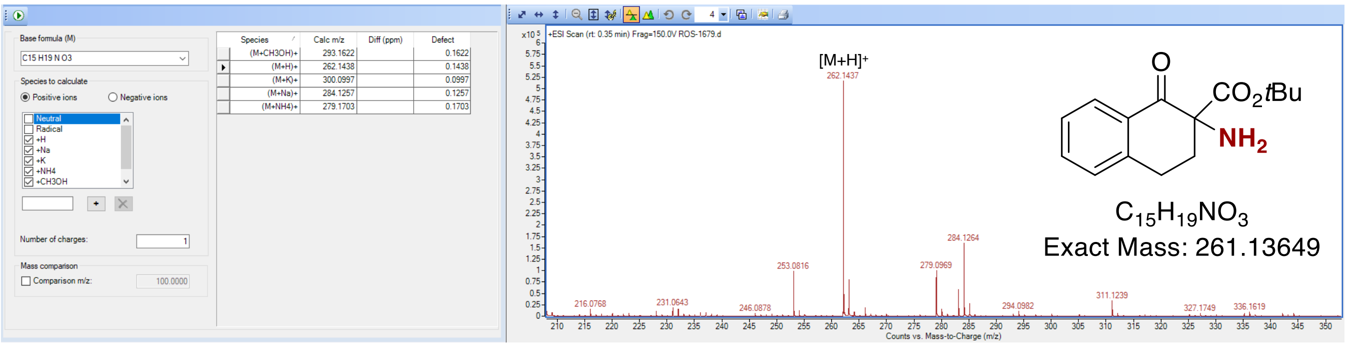

4b, HRMS (ESI+) m/z calcd for $[C_{15}H_{18}FNO_3+H]^+$ 280.1343, found 280.1344 (+0.4 ppm).

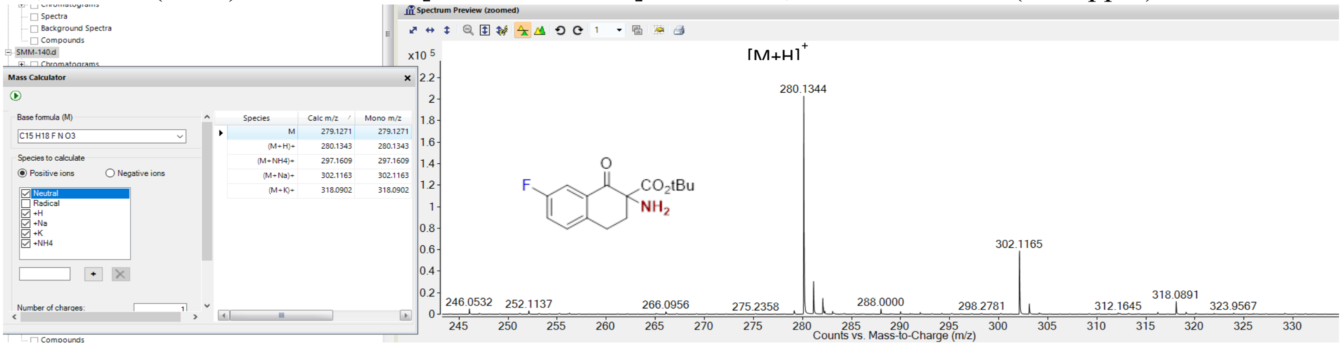

4c, HRMS (ESI+) m/z calcd for $[C_{16}H_{21}NO_4+H]^+$ 292.1543, found 292.1545 (+0.7 ppm).

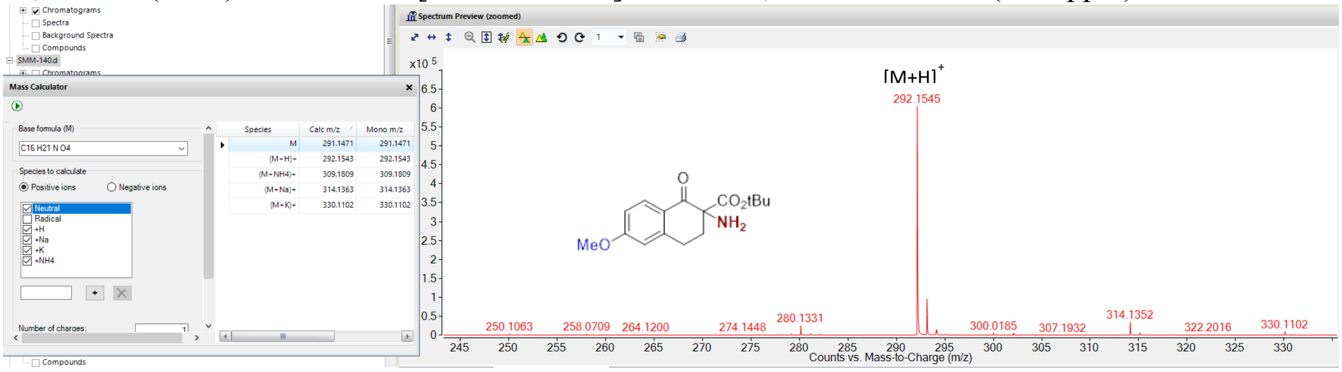

4d, HRMS (ESI+) m/z calcd for $[C_{15}H_{20}N_2O_2+H]^+$ 261.1598, found 261.1602 (−1.5 ppm).

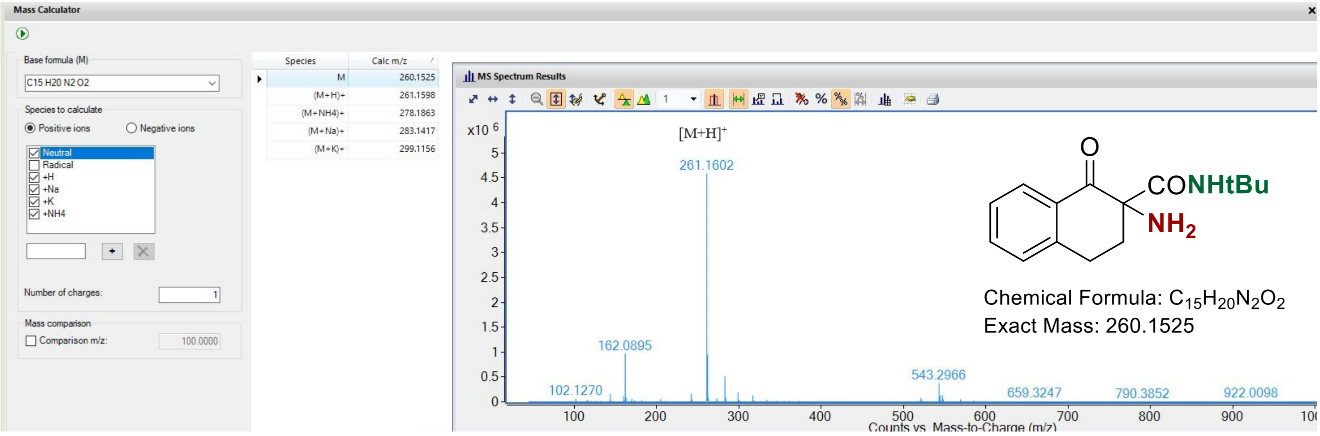

5a, HRMS (ESI+) *m/z* calcd for $[C_{20}H_{22}N_2O_3+CH_3OH+H]^+$ 371.1965, found 371.1964 (−0.4 ppm).

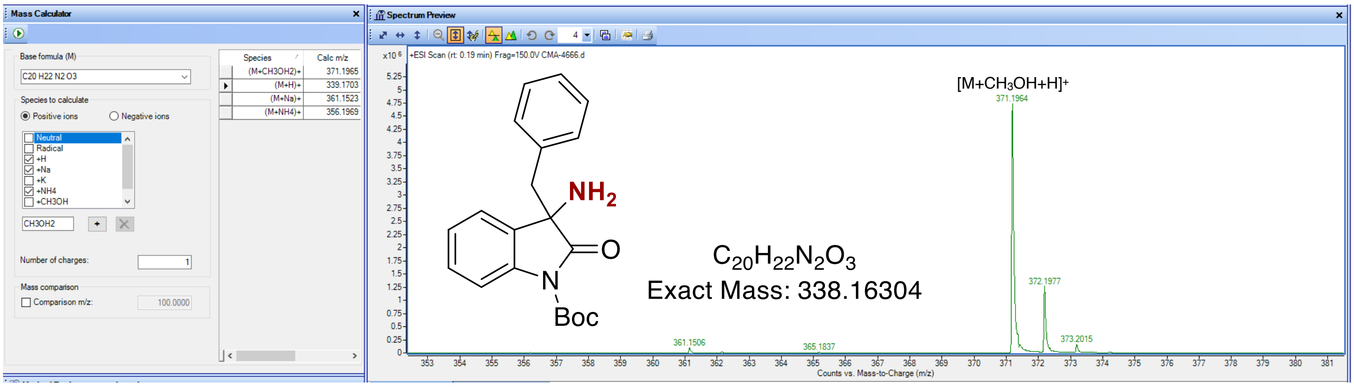

5a: Also detected *m/z* calcd for $[C_{20}H_{22}N_2O_3+Na]^+$ 361.1523, found 361.1523.

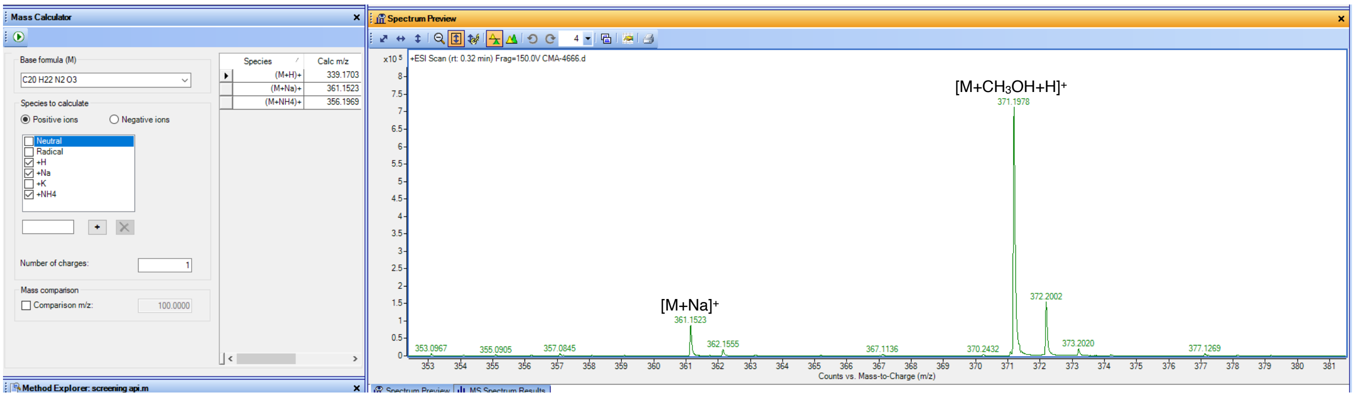

5b, HRMS (ESI+) *m/z* calcd for $[C_{19}H_{20}N_2O_3+CH_3OH+H]^+$ 357.1809, found 357.1805 (−1.1 ppm); also detected *m/z* calcd for $[C_{19}H_{20}N_2O_3+Na]^+$ 347.1366, found 347.1368 (+0.5 ppm).

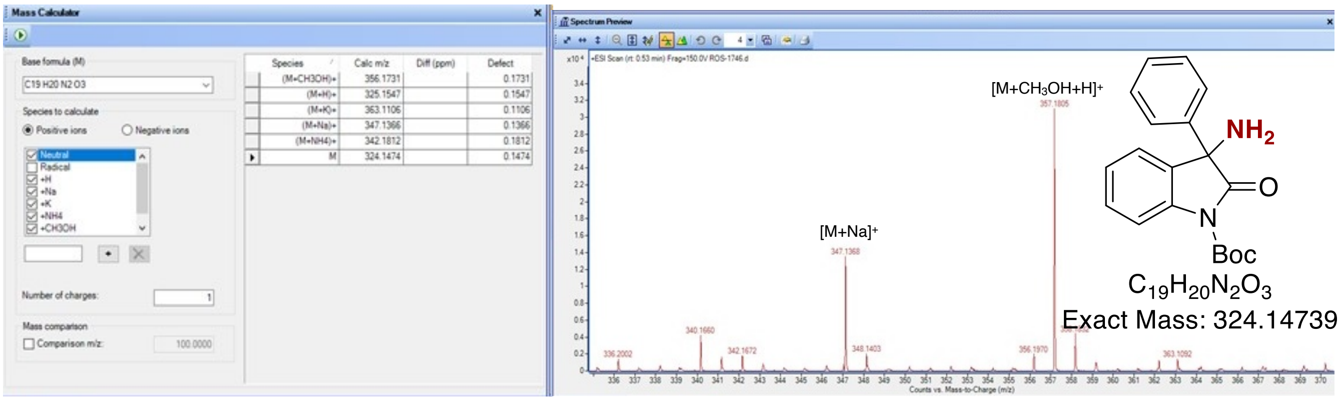

5c, HRMS (ESI+) *m/z* calcd for $[C_{21}H_{24}N_2O_4+CH_3OH+H]^+$ 401.2071, 401.2065 found (-1.5 ppm).

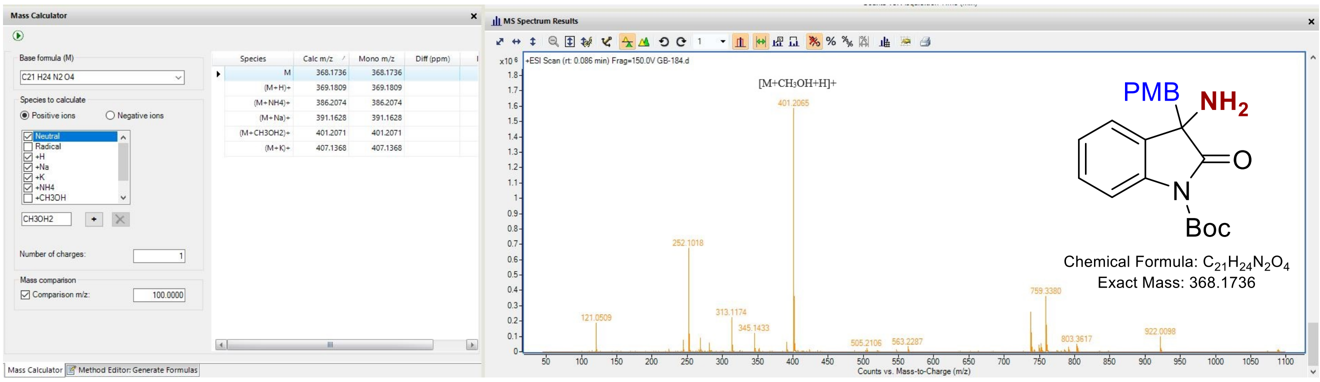

6a, HRMS (ESI+) *m/z* calcd for $[C_{14}H_{18}N_2O_2+H]^+$ 247.1441, found 247.1442 (+0.4 ppm); also detected *m/z* calcd for $[C_{14}H_{18}N_2O_2+Na]^+$ 269.1260, found 269.1259 (−0.6 ppm).

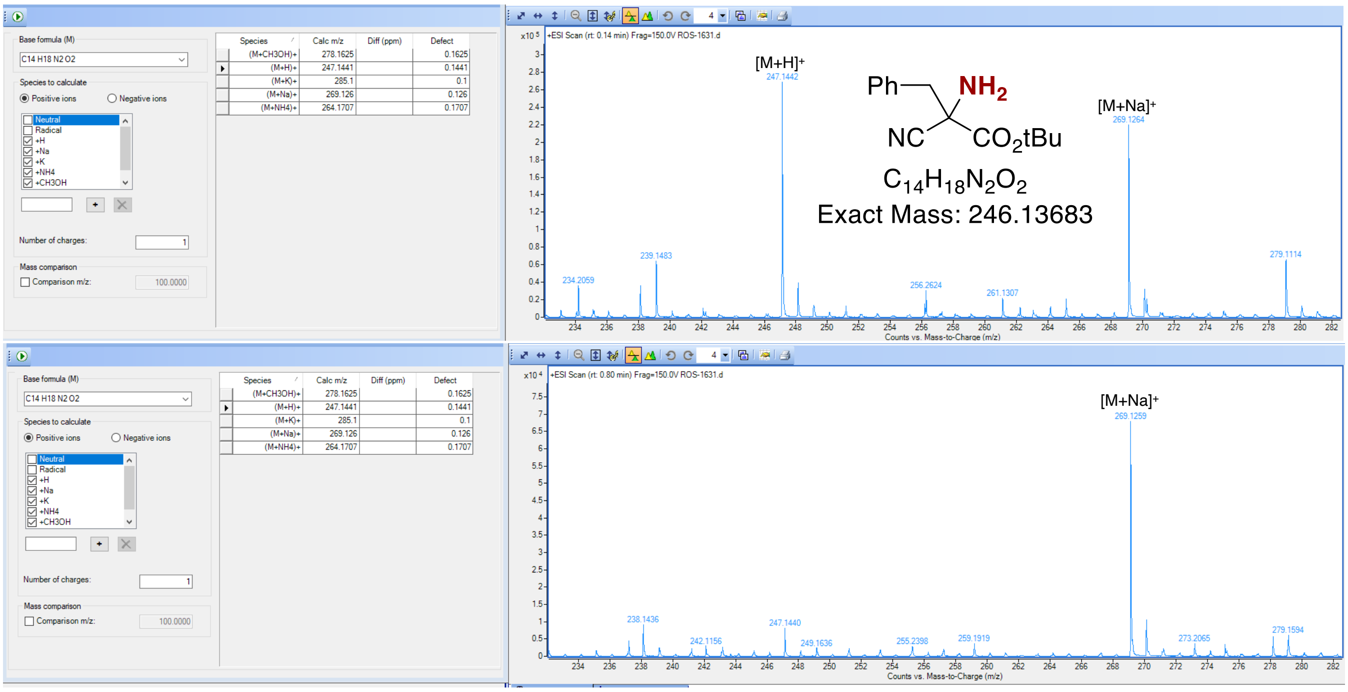

6b, HRMS (ESI+) *m/z* calcd for $[C_{14}H_{19}NO_4+H]^+$ 266.1387, found 266.1388 (+0.4 ppm).

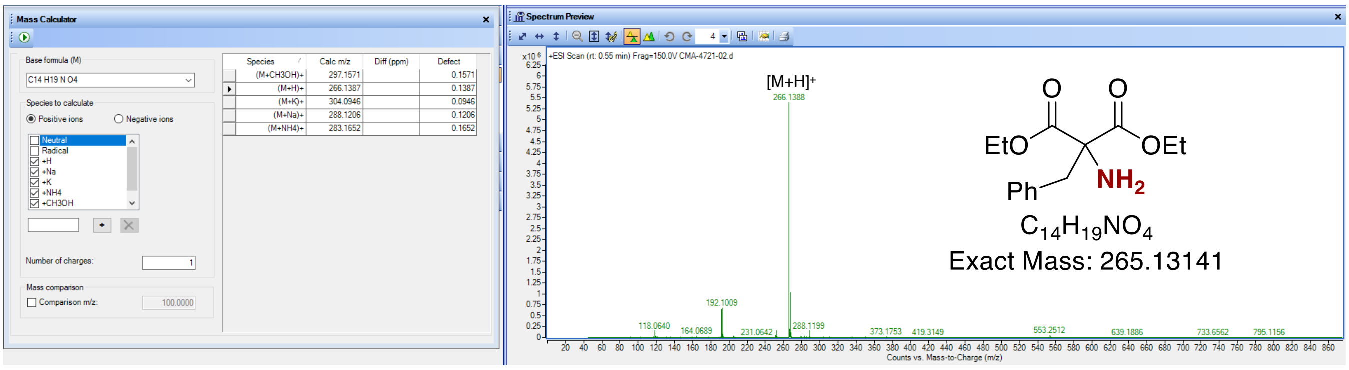

6c, HRMS (ESI+) *m/z* calcd for $[C_{19}H_{21}NO_3+H]^+$ 312.1594, found 312.1592 (-0.6 ppm).

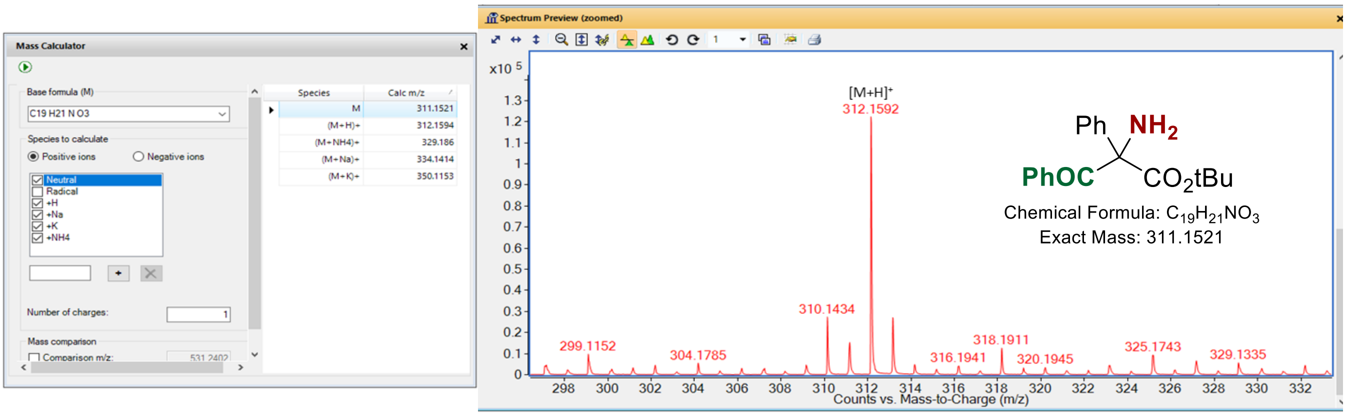

7. Differential Scanning Calorimetry

DSC of the mixture after the complete reaction:

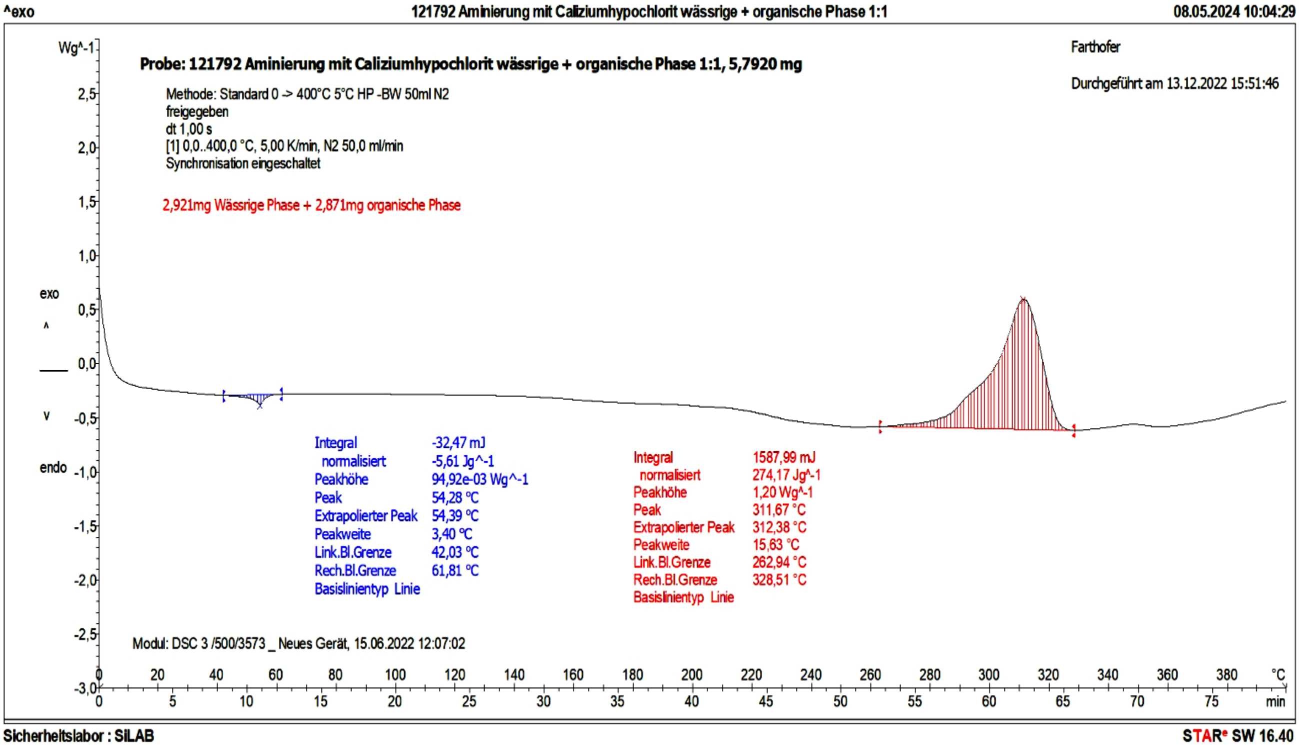

Interpretation: The sample shows a significant exothermic conversion beginning at 263°C (274 J/g). This temperature is quite above the boiling point of that mixture and therefore no thermal risk during handling of that solution is expected. Please note that this exothermic event is most likely caused by the hydrolysis of chlorobenzene by ammonia and / or water at this elevated temperature.

DSC of the upper organic layer after the reaction:

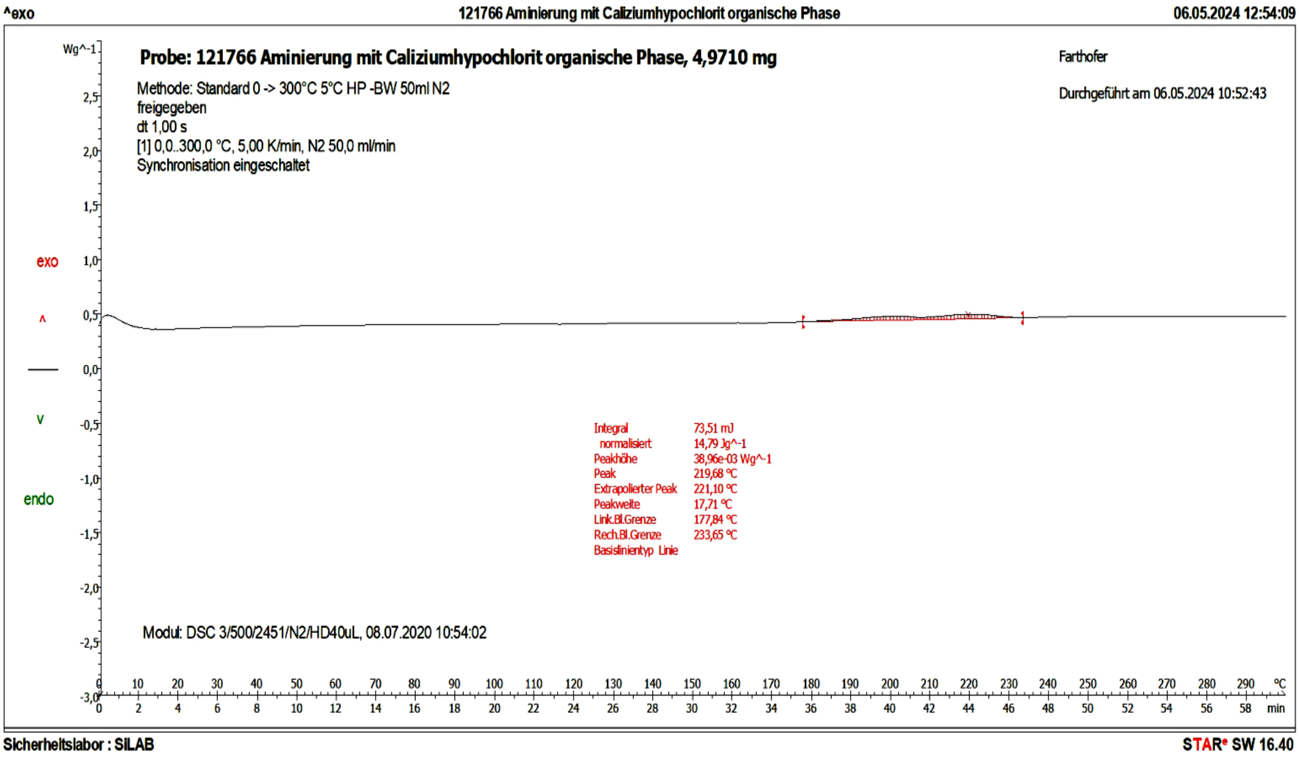

Interpretation: No significant exothermic conversion in the temperature range between 0°C and 300°C. No thermal risk during handling of that compound is expected.

DSC of the lower aqueous layer after the reaction:

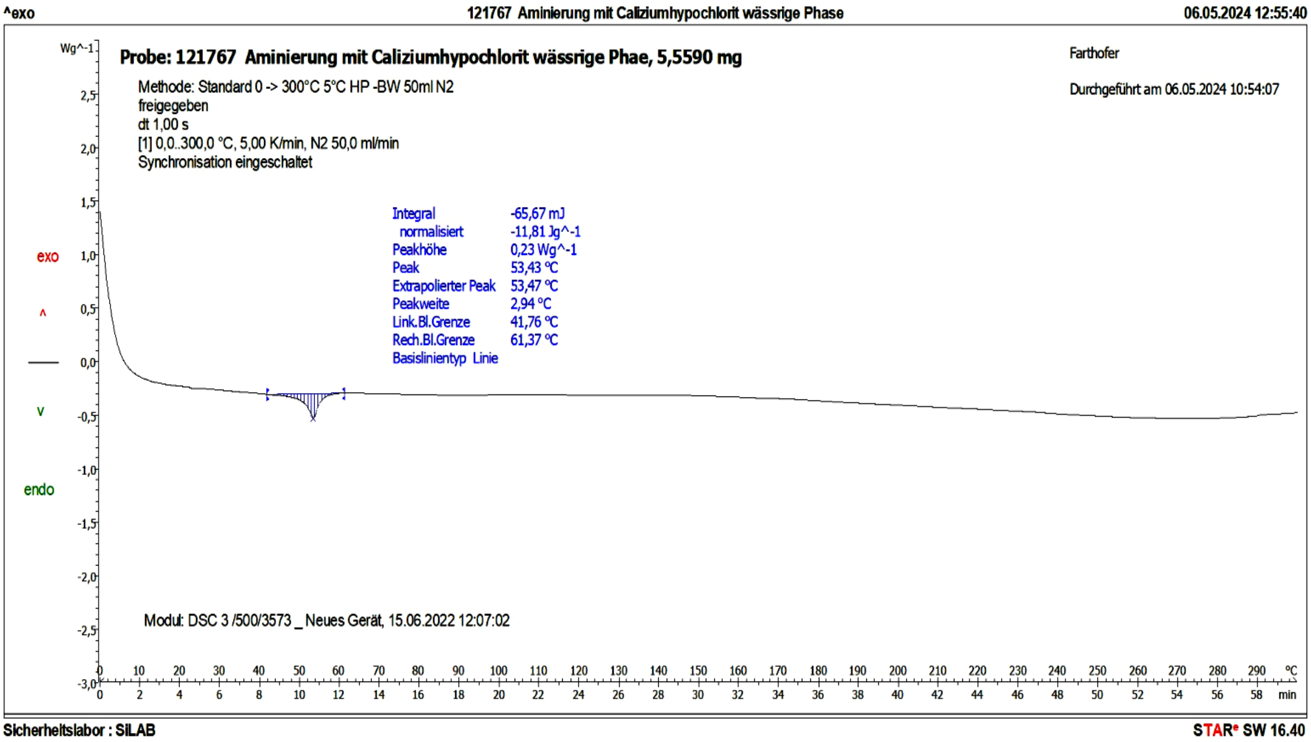

Interpretation: No significant exothermic conversion in the temperature range between 0°C and 300°C. No thermal risk during handling of that compound is expected.
